# BET inhibitor trotabresib in heavily pretreated patients with solid tumors and diffuse large B-cell lymphomas

**DOI:** 10.1038/s41467-023-36976-1

**Published:** 2023-03-13

**Authors:** Victor Moreno, Maria Vieito, Juan Manuel Sepulveda, Vladimir Galvao, Tatiana Hernández-Guerrero, Bernard Doger, Omar Saavedra, Carmelo Carlo-Stella, Jean-Marie Michot, Antoine Italiano, Massimo Magagnoli, Cecilia Carpio, Antonio Pinto, Rafael Sarmiento, Barbara Amoroso, Ida Aronchik, Ellen Filvaroff, Bishoy Hanna, Xin Wei, Zariana Nikolova, Irene Braña

**Affiliations:** 1 START Madrid-FJD, Hospital Universitario Fundación Jimenez Diaz, Madrid, Spain; 2 Vall d’Hebron University Hospital, Vall d’Hebron Institute of Oncology (VHIO), Barcelona, Spain; 3 Hospital Universitario 12 de Octubre, Madrid, Spain; 4 Department of Biological Sciences, Humanitas University, Rozzano, Milano, Italy; 5 Department of Oncology and Hematology, Humanitas Research Hospital – IRCCS, Rozzano, Milano, Italy; 6 Institut Gustave Roussy, Villejuif, France; 7 Institut Bergonie Centre Regional de Lutte Contre Le Cancer de Bordeaux et Sud Ouest, Bordeaux, France; 8 Hematology-Oncology & Stem Cell Transplantation Unit, Istituto Nazionale Tumori, Fondazione G. Pascale, IRCCS, Naples, Italy; 9 Centre for Innovation and Translational Research Europe, a Bristol Myers Squibb Company, Seville, Spain; 10 Bristol Myers Squibb, Princeton, NJ USA

**Keywords:** Cancer therapy, Cancer therapy, CNS cancer, B-cell lymphoma

## Abstract

Bromodomain and extraterminal proteins (BET) play key roles in regulation of gene expression, and may play a role in cancer-cell proliferation, survival, and oncogenic progression. CC-90010-ST-001 (NCT03220347) is an open-label phase I study of trotabresib, an oral BET inhibitor, in heavily pretreated patients with advanced solid tumors and relapsed/refractory diffuse large B-cell lymphoma (DLBCL). Primary endpoints were the safety, tolerability, maximum tolerated dose, and RP2D of trotabresib. Secondary endpoints were clinical benefit rate (complete response [CR] + partial response [PR] + stable disease [SD] of ≥4 months’ duration), objective response rate (CR + PR), duration of response or SD, progression-free survival, overall survival, and the pharmacokinetics (PK) of trotabresib. In addition, part C assessed the effects of food on the PK of trotabresib as a secondary endpoint. The dose escalation (part A) showed that trotabresib was well tolerated, had single-agent activity, and determined the recommended phase 2 dose (RP2D) and schedule for the expansion study. Here, we report long-term follow-up results from part A (N = 69) and data from patients treated with the RP2D of 45 mg/day 4 days on/24 days off or an alternate RP2D of 30 mg/day 3 days on/11 days off in the dose-expansion cohorts (parts B [N = 25] and C [N = 41]). Treatment-related adverse events (TRAEs) are reported in almost all patients. The most common severe TRAEs are hematological. Toxicities are generally manageable, allowing some patients to remain on treatment for ≥2 years, with two patients receiving ≥3 years of treatment. Trotabresib monotherapy shows antitumor activity, with an ORR of 13.0% (95% CI, 2.8–33.6) in patients with R/R DLBCL (part B) and an ORR of 0.0% (95% CI, 0.0–8.6) and a CBR of 31.7% (95% CI, 18.1–48.1) in patients with advanced solid tumors (part C). These results support further investigation of trotabresib in combination with other anticancer agents.

## Abstract (web summary)

Bromodomain and extraterminal proteins (BET) are reported as targets for anticancer therapy. Here, the authors report the final results of a phase I clinical trial of the BET inhibitor trotabresib in patients with solid tumours and diffuse large B-cell lymphoma.

## Introduction

Bromodomain and extraterminal proteins (BET) are epigenetic readers that bind to acetylated lysine residues on histones in chromatin^1–7^. The BET family comprises four proteins: BRD2, BRD3, BRD4, and BRDT^1,2^. BRD2, BRD3, and BRD4 are ubiquitously expressed, while BRDT expression in healthy tissue is restricted to the testes^2,8,9^. BET proteins play key roles in the regulation of gene expression, primarily through recruitment of the Mediator complex and positive transcription elongation factor b (p-TEFb) to acetylated histones. Aberrant acetylation of histones surrounding proto-oncogenes during oncogenesis and tumor progression suggests that BET proteins may play a role in cancer cell proliferation, survival, and oncogenic progression, providing a rationale for using BET inhibitors as anticancer drugs^5–7,10–17^. BET proteins, particularly BRD4, have been identified as having an oncogenic role in lymphomas and various solid tumors via effects on MYC and other transcription factors and signaling modulators^12,17–19^. *MYC* overexpression or rearrangement has been shown to be associated with poor prognosis in patients with diffuse large B-cell lymphoma (DLBCL)^20^.

Trotabresib (CC-90010; BMS-986378) is an orally administered, potent, reversible inhibitor of BET family members. CC-90010-ST-001 is a phase I, multicenter, open-label study to assess the safety, tolerability, pharmacokinetics (PK), and preliminary efficacy of trotabresib in patients with advanced or unresectable solid tumors or relapsed/refractory (R/R) DLBCL. The dose escalation (part A) of this trial evaluated a wide range of doses and treatment schedules, with the intention of optimizing the treatment-free interval in each treatment cycle to improve the tolerability of the dose selected for further evaluation in the expansion cohorts^18^. The recommended phase II dose (RP2D) was 45 mg/day 4 days on/24 days off, with 30 mg/day 3 days on/11 days off selected as an alternative RP2D^18^, based on the observed good tolerability, favorable PK, encouraging pharmacodynamics, and antitumor activity^18^. At the time of analysis, eight patients in part A remained on treatment with an ongoing response or stable disease (SD), of whom five subsequently remained on treatment for ≥24 cycles^18,21^.

Here, we report long-term follow-up of safety and efficacy from the dose escalation (part A) and, for the first time, present results from the dose-expansion cohorts (parts B and C), including evaluation of food effects on trotabresib safety and PK.

## Results

### Patients and treatment

Patients were enrolled at 11 study sites in France, Italy, Japan, and Spain. Patient enrollment in part A took place between July 31, 2017 and November 12, 2018, with a total of 69 patients enrolled and treated, of whom 67 had advanced or unresectable solid tumors and two had R/R DLBCL. Patient enrollment in part B began on June 18, 2019, and was ongoing at the time of the data cutoff; the last patient enrolled at the time of the data cutoff was enrolled on January 11, 2021. In total, 23 patients with R/R DLBCL were enrolled and treated in part B, of whom 19 received trotabresib 45 mg/day 4 days on/24 days off and four received an alternate RP2D dose and schedule of trotabresib 30 mg/day 3 days on/11 days off. The alternate RP2D was investigated further because it had comparable tolerability to 45 mg/day 4 days on/24 days off, delivered the same cumulative dose over a 28-day treatment cycle, and was potentially more suitable for the treatment of patients with aggressive tumors such as R/R DLBCL, due to more constant exposure and target engagement. Patient enrollment in Part C took place between October 21, 2019 and July 27, 2020, with a total of 41 patients with advanced solid tumors enrolled to evaluate the effects of food on the PK and safety profile of trotabresib 45 mg/day 4 days on/24 days off.

**Fig. 1:**
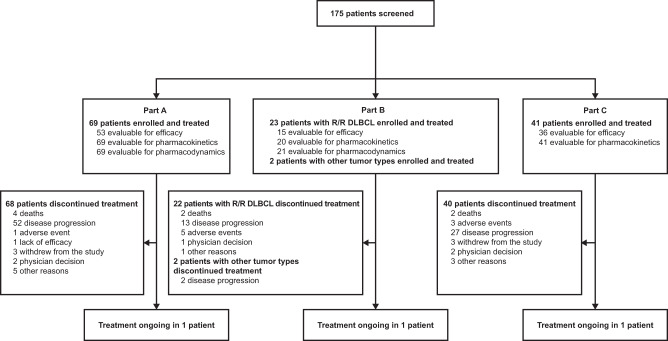
Patient disposition as of June 16, 2022. *DLBCL* diffuse large B-cell lymphoma, *R/R* relapsed/refractory.

Details of patient disposition are shown in Fig. 1. At the time of preparing this report (June 16, 2022), one patient in part A, one patient in part B, and one patient in part C remained on treatment. Analysis of overall safety and efficacy was performed using a data cutoff of July 15, 2021, for parts A, B, and C; food effect during treatment cycles 1 and 2 in part C was evaluated using data with a cutoff of January 29, 2021.

### Patient characteristics

Baseline patient demographics and characteristics are shown in Table 1. Patients in part A and patients with R/R DLBCL in part B had received a median (range) of 4 (1–9) prior treatment regimens, and patients in part C had received a median of 2 (1–8) prior treatment regimens. Compared with patients in part A and part C (median age 57 and 58 years, respectively), patients with R/R DLBCL in part B were older (median age 66 years), and a higher proportion had an Eastern Cooperative Oncology Group performance status (ECOG PS) of 1 (70% vs 52% in part A and 37% in part C). Two patients with R/R DLBCL (9%) had double-hit lymphoma, and no patients had received prior chimeric antigen receptor T-cell therapy. Detailed DLBCL-specific patient and tumor characteristics are shown in Supplementary Table 1.

**Table 1:** Baseline patient demographics and characteristics for parts A, B, and C of the CC-90010-ST-001 study

Patient characteristic	Part A (solid tumors) (*n* = 69)	Part B			Part C (solid tumors) (*n* = 41)
		BCC & NUT carcinoma^a^ (*n* = 2)	R/R DLBCL^b^ (*n* = 23)		
Age, median, y (range)	57 (21–80)	67 (59–75)	66 (35–79)		58 (20–79)
≥65 y, *n* (%)	21 (30)	1 (50)	12 (52)		11 (27)
Male, *n* (%)	38 (55)	1 (50)	12 (52)		18 (44)
Female, *n* (%)	31 (45)	1 (50)	11 (48)		23 (56)
ECOG PS, *n* (%)					
0	33 (48)	0	7 (30)		26 (63)
1	36 (52)	2 (100)	16 (70)		15 (37)
Tumor type, *n* (%)					
Glioblastoma	10 (14)	0	0		2 (5)
BCC	0	1 (50)	0		0
NUT carcinoma	0	1 (50)	0		1 (2)
Other solid tumor^c^	57 (83)	0	0		38 (93)
DLBCL	2 (3)	0	23 (100)		0
Prior systemic cancer therapies, median, *n* (range)	4 (1–9)	1^d^	4 (2–7)		2 (1–8)
Prior stem cell transplant, *n* (%)	0	0	6 (26)		0
Time from the initial diagnosis to the first dose of study drug, median, months (range)	40.6 (2.2–506.7)	222.8 (1.1–444.6)		28.5 (5.0–153.5)	35.7 (5.6–268.6)

*BCC* basal cell carcinoma, *DLBCL* diffuse large B-cell lymphoma, *ECOG* *PS* Eastern Cooperative Oncology Group performance status, *NUT* nuclear protein in testis, *R/R* relapsed/refractory.

^a^Two patients, one with advanced BCC and one with NUT midline carcinoma, were enrolled in this cohort before enrollment was stopped due to slow recruitment.

^b^Nineteen patients received trotabresib 45 mg/day 4 days on/24 days off, and four patients received trotabresib 30 mg/day 3 days on/11 days off.

^c^Includes salivary gland tumors, colorectal cancer, prostate cancer, lung cancer, astrocytoma other than grade 4, endometrial cancer, thymic cancer, medulloblastoma, and others.

^d^Data presented for one patient.

### Safety

**Fig. 2:**
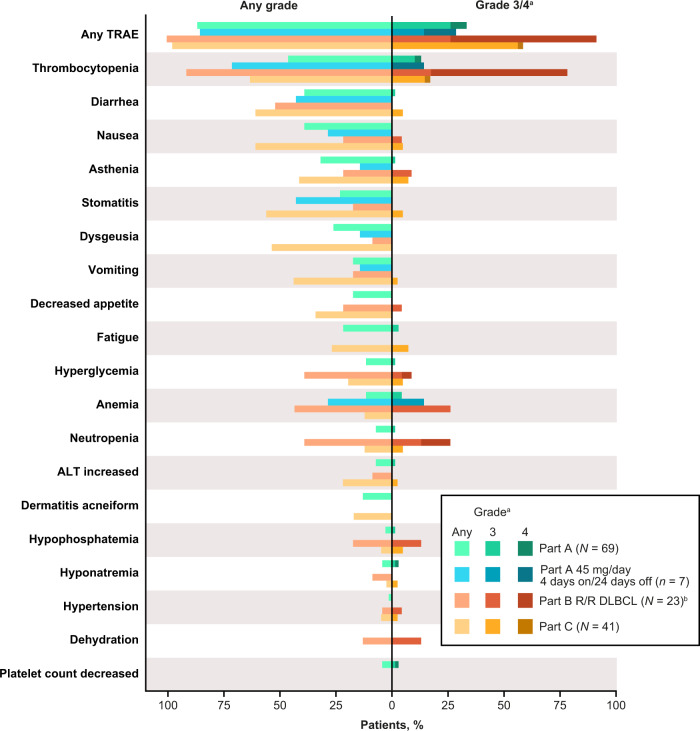
Treatment-related adverse events reported in ≥10% of patients in the overall study population, or in ≥2 patients at grade ≥3 severity. Grade 3 essential hypertension, hypokalemia, lipase increased, liver function test abnormal, presyncope, skin hemorrhage, and grade 4 blood creatinine phosphokinase increased, diabetes mellitus, and inappropriate antidiuretic hormone secretion were reported in one patient each in the overall part A population. Grade 3 abdominal infection, blood bilirubin increased, pneumonia and grade 4 blood creatinine increased, febrile neutropenia, leukopenia, and lymphopenia were reported in one patient each in the part B R/R DLBCL population. Grade 3 gamma-glutamyl transferase increased, hyperamylasemia, and syncope were reported in one patient each in the part C population. Source data are provided as a source data file. ^a^No grade 5 TRAEs were reported during the study. ^b^Nineteen patients received trotabresib 45 mg/day 4 days on/24 days off and four patients received trotabresib 30 mg/day 3 days on/11 days off. *ALT* alanine aminotransferase, *DLBCL* diffuse large B-cell lymphoma; *R/R* relapsed/refractory, *TRAE* treatment-related adverse event.

Safety and tolerability were the primary endpoints of the study. Safety results for part A at the most recent follow-up were consistent with the preliminary analysis reported previously^18^. In patients with R/R DLBCL in part B, any-grade and grade 3/4 TRAEs were reported in 23 (100%) and 21 (91%) patients, respectively (Fig. 2). The most common grade 3/4 TRAEs were hematologic, including thrombocytopenia (18 patients [78%]), anemia (six patients [26%]), and neutropenia (six patients [26%]). Serious TRAEs were reported in seven (30%) patients. Two (9%) patients with R/R DLBCL in part B died due to TEAEs that were not considered to be related to the study treatment. In part C, any-grade TRAEs were reported in 40 (98%) patients, and grade 3/4 TRAEs were reported in 24 (59%) patients. The most common grade 3/4 TRAE was thrombocytopenia, reported in seven (17%) patients. A TEAE leading to death was reported in one patient in part C, but was not considered to be related to the study treatment.

Details﻿ of TRAEs and all-cause TEAEs occurring at any-grade and grade 3–5 severity are shown in Supplementary Tables 2 and 3, respectively. A summary of dose modifications and treatment discontinuation due to adverse events is shown in Supplementary Table 4.

### Efficacy

Preliminary efficacy was a secondary endpoint of the study. Response rates in part A were unchanged from those previously reported^18^. One patient with grade 2 diffuse astrocytoma had a durable complete response (CR) lasting 19 cycles before disease progression, and one patient with endometrial cancer had a confirmed partial response (PR) for 8 cycles. The objective response rate (ORR; CR or PR) was 2.9% (95% CI, 0.4–10.1) and the clinical benefit rate (CBR; CR, PR, or SD ≥4 months) was 17.4% (95% CI, 9.3–28.4). A total of five patients with various tumor types remained on treatment for ~2 years or longer (≥24 cycles) with sustained SD. One patient with thymic cancer remained on treatment for 3.1 years (38 cycles) before discontinuing due to disease progression, and treatment was ongoing in one patient with salivary gland cancer at cycle 53 as of June 16, 2022, with a total treatment duration of 4.0 years (Supplementary Fig. 1).

**Fig. 3:**
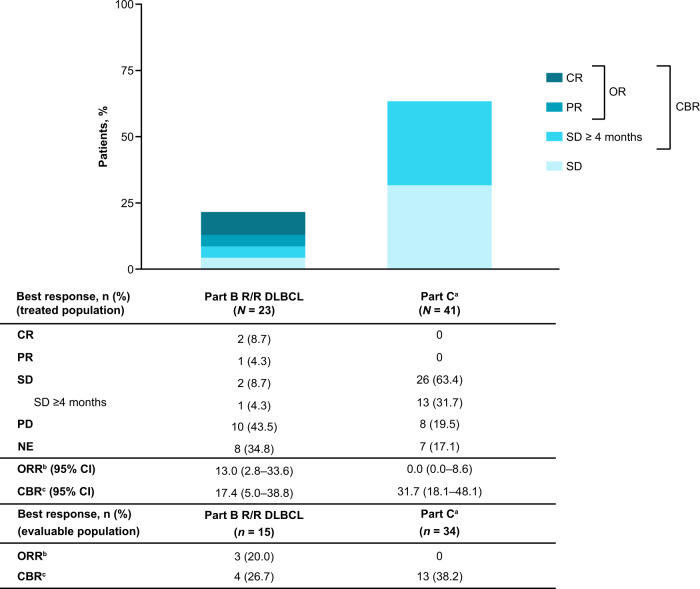
Summary of antitumoral activity. Source data are provided as a source data file. ^a^Confirmed best response is presented for part C, defined as a consecutive response of the same or better that is at least 4 weeks apart, regardless of tumor type. ^b^ORR = CR + PR. ^c^CBR = CR + PR + SD ≥4 months. *CBR* clinical benefit rate, *CR* complete response, *DLBCL* diffuse large B-cell lymphoma, *ORR* objective response rate, *PR* partial response, *R/R* relapsed/refractory, *SD* stable disease.

**Fig. 4:**
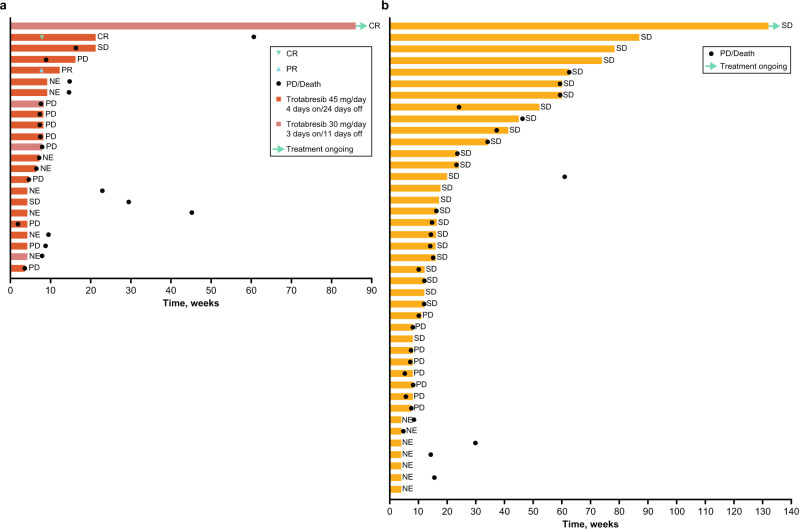
Duration of treatment and best response. **a** Patients with R/R DLBCL in part B (*n* = 23) and **b** part C (*n* = 41). A swim plot showing the duration of treatment for patients in part A is shown in Supplementary Fig. 1. Source data are provided as a source data file. *CR* complete response, *DLBCL* diffuse large B-cell lymphoma, *NE* not evaluable, *R/R* relapsed/refractory, *SD* stable disease, *PD* progressive disease, *PR* partial response.

Of the 23 patients with R/R DLBCL treated in part B, 15 (65.2%) were evaluable for response (Fig. 3). Two patients (8.7%) had CR, one patient had PR (4.3%), and two patients had SD (8.7%), with one patient (4.3%) having SD ≥4 months, resulting in an ORR of 13.0% (95% CI, 2.8–33.6) and a CBR of 17.4% (95% CI, 5.0–38.8). PD was reported in 10 (43.5%) patients. One patient with Ann Arbor stage IVa transformed DLBCL with germinal center B-cell molecular subtype, positive *BCL2* translocation, and negative *MYC* and *BCL6* translocation remained on treatment at cycle 17 as of June 16, 2022, with a total treatment duration of 1.6 years. Of note, the patient had an initial PR at cycle 2 that subsequently deepened to CR at cycle 4 (Fig. 4b).

Of the 41 patients treated in part C, a total of 34 (82.9%) were evaluable for response (Fig. 3). No confirmed CRs or PRs were reported at the time of data cutoff (ORR 0.0% [95% CI, 0.0–8.6]). A total of 26 (45.5%) patients had a best response of SD, of whom 13 had SD of ≥4 months’ duration, resulting in a CBR of 31.7% (95%CI, 18.1–48.1). PD was reported in 8 (19.5%) patients. As of June 16, 2022, treatment was ongoing in a patient with treatment-refractory high-grade astrocytoma, with a duration of 2.5 years. A further seven patients with various tumor types remained on treatment for ≥1 year with sustained SD (Fig. 4c).

**Fig. 5:**
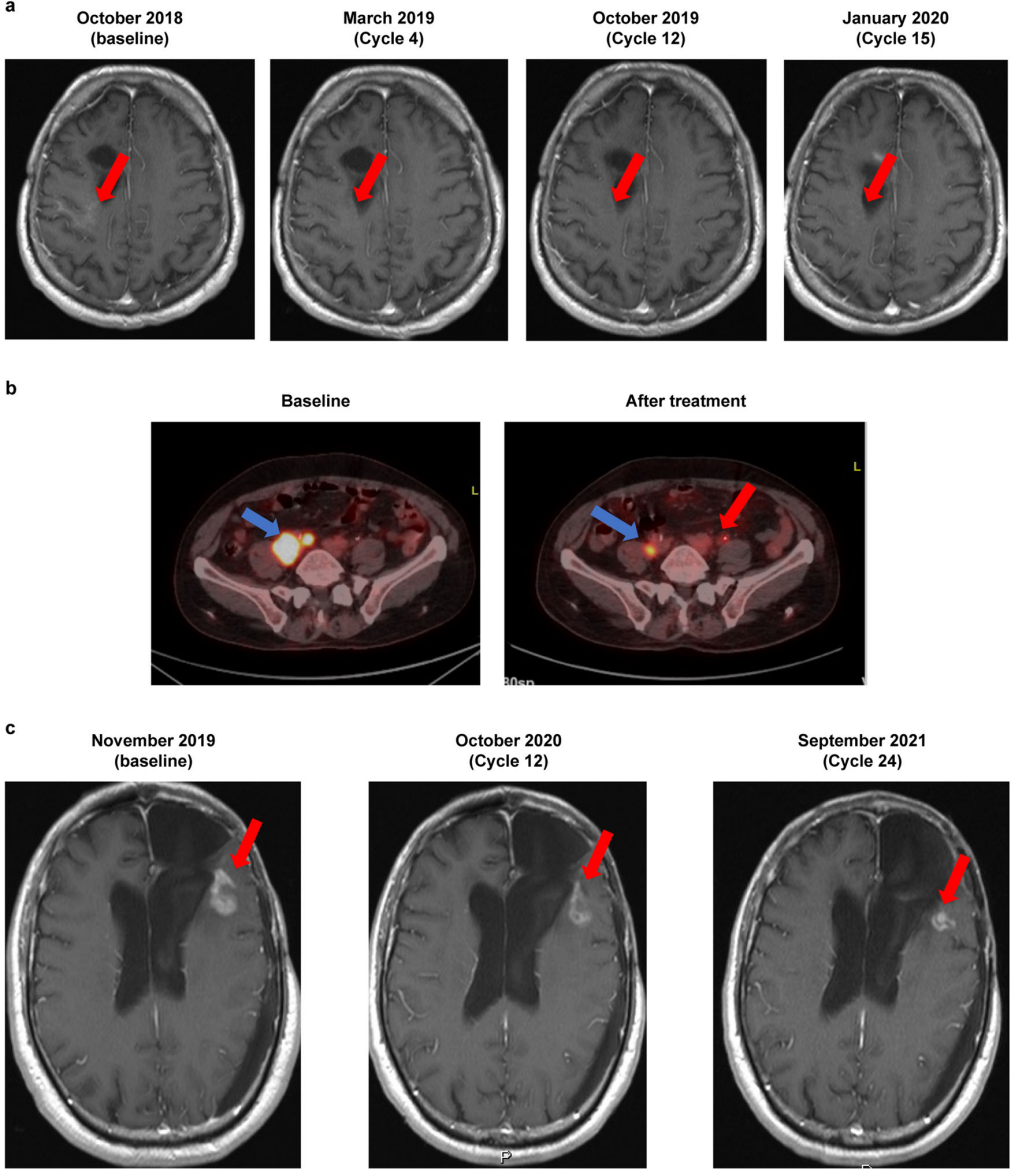
Imaging results of representative responses to trotabresib. **a** MRI evaluation of a patient with grade 2 astrocytoma. The patient had a complete response on trotabresib (red arrows), with the disappearance of both enhancing and non-enhancing areas compared with baseline. The patient discontinued after 19 cycles of treatment due to disease progression. **b** FDG-PET/CT evaluation of a patient with germinal center B-cell immunophenotype R/R DLBCL who had a 57% reduction in target lesion size after two cycles of trotabresib (blue arrow). This was accompanied by the appearance of a new metabolic lesion (red arrow), and treatment was discontinued after four cycles of trotabresib due to disease progression. **c** MRI evaluation of a patient with refractory high-grade astrocytoma. The patient had a minor response on trotabresib with a 38% reduction in tumor size (red arrows), and the patient remained on study treatment at cycle 34 with a continued response as of June 16, 2022. *CT* computed tomography, *DLBCL* diffuse large B-cell lymphoma, *FDG* fluorodeoxyglucose, *MRI* magnetic resonance imaging, *PET* positron emission tomography, *R/R* relapsed/refractory. The two left-most images in panel **a** are reprinted from *Annals of Oncology* 31(6), Moreno V, et al. Phase I study of CC-90010, a reversible, oral BET inhibitor in patients with advanced solid tumors and relapsed/refractory non-Hodgkin’s lymphoma, Page 780–788, Copyright 2020, with permission from Elsevier.

Imaging results showing representative responses to trotabresib in a patient with grade 2 astrocytoma enrolled in part A, a patient with germinal center immunophenotype R/R DLBCL enrolled in part B, and a patient with high-grade astrocytoma enrolled in part C are presented in Fig. 5a–c, respectively.

Median overall survival (OS) and median progression-free survival (PFS) in the treated patient population are shown in Table 2. In part A, median OS and PFS were 189 days (95% CI, 161–258) and 57 days (95% CI, 52–81), respectively. In part B, median OS and PFS were 159 days (95% CI, 101 to not estimable [NE]) and 54 days (95% CI, 49–65), respectively. In part C, the median OS was 508 days (95% CI, 239–NE), and the median PFS was 114 days (95% CI, 84–239).

**Table 2:** Median overall and progression-free survival

	Part A (*n* = 69)	Part B DLBCL (*n* = 23)	Part C (*n* = 41)
Median OS, days (95% CI)	189 (161–258)	159 (101–NE)	508 (239–NE)
Range	15–853	44–423	33–508
Median PFS, days (95% CI)	57 (52–81)	54 (49–65)	114 (84–239)
Range	11–945	12–423	33–438

*CI* confidence interval, *DLBCL* diffuse large B-cell lymphoma, *NE* not estimable, *OS* overall survival, *PFS* progression-free survival.

### Pharmacokinetics and food effects

Food effects on the PK and safety profile of trotabresib were secondary endpoints of the study. A total of 15 patients completed both the fasted and fed treatment periods in part C and were included in the food effect–evaluable population for analysis of the effects of food on trotabresib PK (Table 3). Trotabresib showed generally comparable PK and interindividual variability in fed and fasted patients. Although peak plasma trotabresib concentration (C_max_) was higher and time-to-peak plasma trotabresib concentration (t_max_) was shorter in fasted patients compared with fed patients, area under the plasma trotabresib concentration–time curve (AUC) from 0 to infinity and terminal half-life (t_1/2_) were similar in fasted and fed patients.

**Table 3:** Effects of food on trotabresib pharmacokinetics in patients with advanced solid tumors in part C

GM (% GCV)	Cycle 1, day 1 (fasted, *n* = 15)^a^	Overall day 4	
		Fasted (*n* = 15)	Fed (*n* = 15)
C_max_, ng/mL	560.28 (41.08)	1117.86 (21.82)	903.72 (30.93)
Median t_max_, h (range)	1.55 (0.50–24.17)	1.50 (1.00–3.00)	4.00 (1.00–6.17)
AUC_0–24_, h.ng/mL	6647.39 (33.82)^b^	17343.04 (25.73)	16820.51 (30.16)^b^
AUC_last_, h.ng/mL	n/a	65126.68 (64.00)	65556.66 (74.44)
AUC_inf_, h.ng/mL	n/a	66493.10 (66.01)	67198.78 (76.73)
Mean t_½_, h (% CV)	n/a	69.06 (39.37)	67.79 (47.52)
Cl_ss_/F, L/h	n/a	2.60 (25.89)	2.65 (28.52)
V_ss_/F, L	n/a	234.65 (40.10)	227.87 (44.88)

*AUC*_0–24_ area under the trotabresib concentration–time curve from 0 to 24 h, *AUC*_inf_ area under the trotabresib concentration–time curve from 0 to infinity, *AUC*_last_ area under the trotabresib concentration–time curve from 0 to the last quantifiable concentration, *Cl*_*ss*_/F apparent trotabresib clearance, *C*_max_ peak plasma trotabresib concentration, *CV* coefficient of variation, *GCV* geometric coefficient of variation, *GM* geometric mean, *n/a* not applicable, *t*_½_ terminal half-life; *t*_max_ time-to-peak plasma trotabresib concentration, *V*_ss_/*F* apparent trotabresib volume of distribution.

^a^Overall data summarizes both the fed and fasted treatment groups, with all patients fasted on cycle 1, day 1.

^b^*n* = 14.

Details of TRAEs reported in patients in part C following fasted (*n* = 39) or fed (*n* = 35) administration of trotabresib during cycles 1 and 2 are shown in Supplementary Table 5. The incidence of TRAEs appeared generally similar in patients treated in both the fed and fasted state. Any-grade TRAEs were reported in 37 of 39 (95%) patients treated in the fasted state and 32 of 35 (91%) patients treated in the fed state, and grade 3/4 TRAEs were reported in 13 of 39 (33%) patients treated in the fasted state and seven of 35 (20%) patients treated in the fed state.

### Pharmacodynamics

**Fig. 6:**
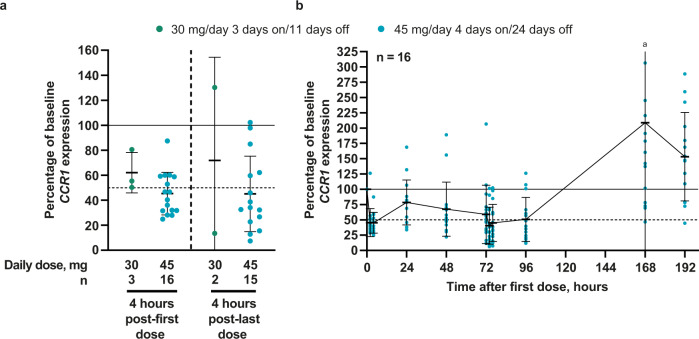
Blood *CCR1* mRNA levels in part B. **a** Four hours after the first (left) and last dose (right) in part B, and **b** from 0–192 h in patients receiving trotabresib 45 mg/day 4 days on/24 days off. For both figures, colored data points indicate individual patient values for the percentage of baseline *CCR1* expression and black lines and error bars indicate mean ± SD. Source data are provided as a source data file. ^a^Data not shown for one patient with a percentage of baseline *CCR1* expression of 1110.93; SD for this time point was 260.36. *CCR1* C-C motif chemokine receptor 1, mRNA messenger RNA.

The pharmacodynamic effects of trotabresib in peripheral blood were assessed as an exploratory endpoint. Blood C-C motif chemokine receptor 1 (*CCR1)* mRNA levels were used as a pharmacodynamic marker of BET inhibition. A minimum of 50% reduction in levels of blood *CCR1* mRNA has previously been shown to be associated with clinical response to a BET inhibitor in patients with R/R lymphoma^22^. Mean *CCR1* mRNA levels were reduced to <50% of baseline at 4 h after the first and last dose of trotabresib 45 mg/day 4 days on/24 days off in part B (Fig. 6a), and reductions were sustained during the first 96 h of treatment (Fig. 6b). *CCR1* modulation with 45 mg/day 4 days on/24 days off was similar in parts A and B^18^.

## Discussion

Effective new treatments for patients with advanced malignancies are urgently needed, particularly for those patients who have tumor types with limited treatment options or whose tumors are refractory to currently available therapies. BET inhibitors have a mechanism of action that differs from existing marketed drugs, and have demonstrated anticancer activity across a broad range of tumor types in preclinical models and patients with various solid and hematological malignancies^10,23–31^. However, clinical trials of first-generation BET inhibitors have generally shown high rates of treatment-related toxicities, particularly dose-limiting hematological and gastrointestinal adverse events, likely due to toxicities arising from the involvement of BET proteins in multiple processes required for cellular homeostasis and the specific features of these agents^10,23–31^. As first-generation BET inhibitors have typically had poor oral bioavailability and short half-lives, ranging from approximately 2 to 20 h, continuous daily dosing or schedules with relatively short treatment-free intervals (≤7 days) have been needed to produce antitumor activity^10,23–31^. Such treatment schedules may have contributed to the increased toxicity due to patients not having sufficient time off treatment to recover.

Trotabresib is a next-generation BET inhibitor with properties that provide potent and selective inhibition of BRD family proteins, as well as a PK profile that allowed evaluation of a range of dosing schedules in part A. Preliminary results of this study were encouraging, with trotabresib showing high oral bioavailability and a comparatively long t_1/2_ of ~60 h^18^. The biweekly and monthly dosing schedules selected for further evaluation showed antitumor activity and incorporated a relatively long treatment-free interval that was found to improve tolerability compared with schedules with more frequent dosing^18^.

Longer follow-up of the dose escalation (part A) and data from parts B and C confirmed that trotabresib was generally very well tolerated in heavily pretreated patients with advanced, unresectable solid tumors and R/R DLBCL. Most TRAEs were mild or moderate and were easily manageable with dose modifications and/or treatment interruption. The most frequent grade 3/4 TRAE was thrombocytopenia, which is a class effect of BET inhibitors^32^. The higher incidence of severe thrombocytopenia reported in patients with R/R DLBCL in part B compared with patients with advanced solid tumors in part C could be related to patients' underlying disease, poorer ECOG PS, older age, and extent of previous treatment. Importantly, thrombocytopenia was reversible with appropriate management.

Biomarker analysis using blood levels of *CCR1* mRNA, a pharmacodynamic marker of BET inhibition evaluated in previous clinical trials^22,33,34^, indicated target engagement and dose/schedule dependency. Reduction of *CCR1* expression to ≤50% of baseline levels has been associated with plasma BET inhibitor concentrations and selected response metrics in lymphoma in a previous study^22^. Analysis of blood *CCR1* mRNA levels during trotabresib treatment in the current study showed a mean reduction of >50% after the first and last dose with trotabresib 45 mg/day 4 days on/24 days off in part B, with sustained inhibition of *CCR1* below baseline during the first 96 h of treatment, providing further support to the observed antitumor activity and dose/schedule selection.

The preliminary efficacy of trotabresib monotherapy in patients with heavily pretreated advanced solid tumors and R/R DLBCL was supportive of antitumor activity, with one CR and one PR reported in part A (ORR 2.9%; 95% CI, 0.4–10.1), two CRs and one PR in part B (ORR 13.0; 95% CI, 2.8–33.6), and one minor response per Response Assessment in Neuro-Oncology (RANO) criteria in part C (ORR 0.0; 95% CI, 0.0–8.6). The longer follow-up of patients enrolled in part A allowed further characterization of responses in this cohort, with the patient with a CR having sustained response for 19 cycles. A total of five patients in part A remained on treatment for ≥24 cycles, including a patient who received 38 cycles of treatment and a patient who remains on treatment at cycle 53, corresponding to treatment durations of 3 and 4 years, respectively. One patient with high-grade astrocytoma in part C has a minor antitumoral response that is ongoing at cycle 34, and a further seven patients with advanced solid tumors in part C completed ≥1 year of treatment. These data suggest that trotabresib monotherapy may provide long-lasting disease control in some patients, and demonstrate the long-term efficacy and good tolerability of intermittent dosing of trotabresib even in patients with advanced, progressive disease. Importantly, the durable CR in a patient with progressive diffuse astrocytoma and the minor radiographic response in a patient with high-grade astrocytoma, a tumor type with typically poor outcomes and limited effective treatment options, illustrate the ability of trotabresib to penetrate brain tumor tissue^35^. Penetration of brain tumor tissue is a key challenge in the treatment of gliomas and other central nervous system tumors^36,37^. Preclinical studies in a wide variety of solid tumors and hematological malignancies have also shown that BET inhibition may be synergistic with a range of anticancer agents, including various targeted therapies and cytotoxic agents^16,38–48^.

Evaluation of the effects of food on trotabresib PK showed generally comparable exposure and plasma concentrations between fed and fasted patients, with a similar t_½_ to that previously reported for patients in part A^18^. Interindividual variability, characterized by the geometric coefficient of variation, was generally similar in fed and fasted patients. These data suggest that food does not markedly alter trotabresib PK, making it an attractive candidate for combination with various anticancer agents that require fed or fasted administration.

As this exploratory phase I first-in-human trial was designed to evaluate trotabresib in patients with advanced cancers for whom no standard therapy was available, as well as to guide dose selection for subsequent studies, it is subject to a number of limitations. Thus, its results should be interpreted with care in the absence of an adequately powered randomized controlled trial.

In summary, the results from this study demonstrated a tolerable safety profile during long-term treatment, a long t_½_, favorable pharmacodynamics, and antitumor activity with trotabresib monotherapy in heavily pretreated patients with advanced solid tumors and R/R DLBCL. Prolonged follow-up of part A, with treatment durations of up to 3 years in some patients, and data from parts B and C confirm the good tolerability of trotabresib when administered using monthly and biweekly intermittent dosing regimens. The extended treatment-free interval included in these regimens is enabled by the long t_½_ of trotabresib. Importantly, evaluation of the effects of food on trotabresib treatment found generally similar PK in fed and fasted patients, providing flexibility for dosing.

The results reported here demonstrate the preliminary anticancer properties of trotabresib monotherapy across a range of heavily pretreated advanced solid tumors and in R/R DLBCL, supporting further investigation of its use in combination with other anticancer agents. Based on the responses observed in this study, a clinical trial has been initiated to investigate trotabresib in combination with temozolomide in patients with newly diagnosed glioblastoma (NCT04324840)^35,49–51^.

## Methods

### Study design

**Fig. 7:**
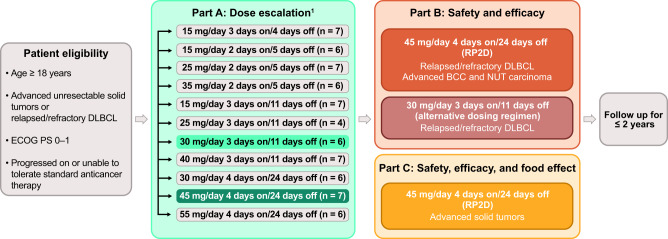
Study design. *BCC* basal cell carcinoma, *DLBCL* diffuse large B-cell lymphoma, *ECOG PS* Eastern Cooperative Oncology Group performance status, *RP2D* recommended phase II dose.

The CC-90010-ST-001 trial comprises three parts (Fig. 7). Part A was a dose-escalation study to determine the RP2D of trotabresib monotherapy. Trotabresib was administered at doses ranging from 15 to 55 mg and schedules ranging from 3 days on/4 days off to 4 days on/24 days off, as previously reported^18^. Part B is an ongoing dose expansion evaluating the safety and efficacy of trotabresib in patients with R/R DLBCL or basal cell carcinoma (BCC). Two trotabresib dosing schedules are being evaluated in patients with R/R DLBCL: the RP2D of trotabresib of 45 mg/day 4 days on/24 days off and an alternative dosing regimen of trotabresib 30 mg 3 days on/11 days off^18^. Part C is an ongoing dose expansion evaluating the safety and efficacy of trotabresib 45 mg/day 4 days on/24 days off in patients with advanced solid tumors, as well as the impact of food on trotabresib PK. The trial is registered on ClinicalTrials.gov (NCT03220347).

Dose-escalation decisions in part A were guided by a Bayesian logistic regression model with overdose control^52^. The number of patients treated at each dose level and schedule was based on empirical considerations, with a maximum of six patients typically treated in each group. For the part B R/R DLBCL expansion cohort, enrollment was based on the probability of making a no-go decision when the true efficacy was truly below the target level. For instance, if the no-go criteria is Pr (ORR <26%) >80%, enrollment of 25 patients would provide a 73% chance to make a no-go decision when the true ORR is 14% (based on the posterior probability of beta-binomial distribution with prior beta [0.35, 1]). Planned enrollment for the part C food effect cohort was also driven by empirical experience, with the enrolled sample size believed to produce sufficient precision for PK parameter assessment based on preliminary PK results from part A.

The study was conducted in accordance with the Declaration of Helsinki and in adherence to Good Clinical Practice. The protocol was reviewed and approved by the Institutional Review Board or Independent Ethics Committee of each site before initiation of the study, and all patients provided written informed consent. The protocol was approved by the Institutional Review Board or Independent Ethics Committee of Institut Gustave Roussy, Villejuif, France; Institut Bergonie Centre Regional de Lutte Contre Le Cancer de Bordeaux et Sud Ouest, Bordeaux, France; Vall d’Hebron University Hospital, Vall d’Hebron Institute of Oncology (VHIO), Barcelona, Spain; Hospital Universitario Fundación Jimenez Diaz, Madrid, Spain; Hospital Universitario 12 de Octubre, Madrid, Spain; Istituto Nazionale Tumori, Fondazione G. Pascale, IRCCS, Naples, Italy; Instituto Clinico Humanitas, Rozzano, Milano, Italy; Instituto Scientifico Romagnolo per lo Studio e la Cura dei Tumori, Meldola, Italy; National Cancer Center Hospital East, Tokyo, Japan; Aichi Cancer Center Hospital, Nagoya, Japan; and The Cancer Institute Hospital of Japanese Foundation for Cancer Research, Tokyo, Japan.

### Patient selection

Patients were enrolled from July 31, 2017 to January 11, 2021. The study included patients aged ≥18 years with an ECOG PS of 0 or 1. Part A included patients with histologically or cytologically confirmed advanced or unresectable solid tumors or R/R advanced NHL that had progressed on standard anticancer therapy or for which no conventional therapy was available.

Part B included patients with histologically or cytologically confirmed R/R DLBCL that had progressed following two or more previous lines of therapy, including autologous stem cell transplant, or that had progressed after at least one previous line of therapy and where the patient was not eligible for or had declined autologous stem cell transplant. Patients with transformed lymphoma following chemotherapy for follicular lymphoma who had received at least two standard treatment regimens for DLBCL were also eligible. Patients must have had a lack of response after chimeric antigen receptor (CAR) T-cell therapy (if available), been ineligible for CAR T-cell therapy, or declined CAR T-cell therapy. Part B also included a cohort of patients with histologically or cytologically confirmed advanced BCC, nuclear protein in testis (NUT) midline carcinoma, advanced salivary gland carcinoma, or advanced endometrial carcinoma and disease progression on, or inability to tolerate, standard therapy, or for whom no standard therapy exists. Patient enrollment in this cohort was subsequently restricted to patients with advanced BCC only in a protocol amendment.

Part C included patients with histologically or cytologically confirmed advanced solid tumors and disease progression on, or inability to tolerate standard therapy, or for whom no standard therapy exists.

Patients with solid tumors had at least one site of measurable disease; patients with NHL had bidimensionally measurable disease on cross-sectional imaging, with at least one lesion >1.5 cm in diameter.

### Endpoints

The primary endpoints of the study were the safety and tolerability of trotabresib, as well as the MTD and/or RP2D of trotabresib. Safety was assessed using the National Cancer Institute Common Terminology Criteria for Adverse Events v4.03^53^.

Secondary objectives were the preliminary efficacy of trotabresib in terms of clinical benefit rate (CR + PR + SD of ≥4 months’ duration), ORR (CR + PR), duration of response or SD, PFS, OS, and the PK of trotabresib. In addition, part C assessed the effects of food on the PK and the safety of trotabresib as a secondary objective. The pharmacodynamics of trotabresib were assessed as an exploratory objective.

Response was assessed using Response Evaluation Criteria in Solid Tumors (RECIST) v1.1^54^ in patients with solid tumors, International Working Group criteria in patients with DLBCL^55^, and Response Assessment in Neuro-Oncology (RANO) criteria^56^ in patients with CNS tumors (astrocytoma and glioblastoma). For patients with BCC, the response was assessed using a combination of radiological assessment of target lesions per RECIST v1.1, digital clinical photography assessed per World Health Organization (WHO) criteria^57^, and punch biopsies to confirm CR. PK parameters assessed included C_max_, t_max_, AUC, t_½_, apparent clearance (CL/F), and apparent volume of distribution (Vz/F).

### Pharmacokinetic and pharmacodynamic assays

Serial blood samples for PK and pharmacodynamic analyses were collected for each dosing schedule after the first and last doses in cycle 1. Additional blood samples for assessment of the food effect on trotabresib PK in part C were collected during cycle 2. The food effect–evaluable population comprised all patients with adequate PK data to allow calculation of trotabresib AUC from time 0 (day 1) to day 22 from both the fed and fasted treatment periods and who had completed a 4-h post-dose fast during the fasted cycle or consumed the entire high-fat, high-calorie meal during the fed cycle and had received all trotabresib doses during both treatment periods.

Changes in peripheral *CCR1* mRNA levels, expression of which is modulated following BET inhibition^33^, were assessed using QuantiGene technology (Thermo Fisher Scientific) and normalized to the expression of a control gene, peptidylprolyl isomerase B (*PPIB*). The data were presented as a percentage of pre-dose mRNA levels.

### Statistical analyses

Bayesian logistic regression model based on dose-limiting toxicity was used to provide initial guidance for dose recommendations in the dose-escalation phase. The final decision was reached by considering the totality of safety, PK, and PD data. For binary efficacy endpoints, two-sided 95% Clopper–Pearson exact confidence intervals were provided. For time-to-event endpoints (PFS and OS), the median and 95% CI were estimated using the Kaplan–Meier method.

### Reporting summary

Further information on research design is available in the Nature Portfolio Reporting Summary linked to this article.

## Supplementary information

Supplementary Information

Reporting Summary

## Data Availability

Anonymized participant-level data cannot be shared due to an increased risk of patient reidentification. Bristol Myers Squibb will consider requests to share anonymized clinical trial data from interventional trials in patients that have completed on or after January 1, 2008. In addition, primary results from these trials must have been published in peer-reviewed journals and the medicines or indications approved in the United States, EU, and other designated markets. Requests to access clinical trial data will be considered case by case and may be submitted to Celgene, a Bristol Myers Squibb Company, using the inquiry form at https://vivli.org/ourmember/bristol-myers-squibb/. Requests for clinical data are initially reviewed by internal Bristol Myers Squibb personnel to ensure alignment with the scope of the data-sharing policy and to check the current or expected availability of the data sets and are then evaluated by an Independent Review Committee to ensure that qualifying requests have a consistent, complete, and fair assessment. Sharing is also subject to the protection of patient privacy and respect for the patient’s informed consent. and must include a description of the research proposal. Information from eligible trials that may be considered for disclosure upon request includes deidentified study-level clinical data, Clinical Study Reports, Statistical Analysis Plans, and Protocols. The study protocol is available as Supplementary Note 1 in the Supplementary Information file. The remaining data were available within the Article, Supplementary Information, or Source Data file. Source data are provided with this paper.

## Source data

Source Data
