# Supplementary material for: BET inhibitor trotabresib in heavily pretreated patients with solid tumors and diffuse large B-cell lymphomas
Source: Nat Commun. 2023 Mar 13;14:1359. doi: 10.1038/s41467-023-36976-1 (PMC10011554; doi:10.1038/s41467-023-36976-1)
Supplement: Supplementary file 1 — Supplementary Information [file 41467_2023_36976_MOESM1_ESM.pdf]

BET inhibitor trotabresib in heavily pretreated patients with solid tumors and diffuse large B-cell lymphomas

Supplementary Information

Supplementary Table 1 DLBCL-specific patient and disease characteristics for the R/R DBLCL population in part B.

Characteristics, *n* (%)	Part B R/R DLBCL (*N* = 23)
Ann Arbor stage at enrollment	
IA	1 (4)
IIA	1 (4)
IIIA	3 (13)
IVA	15 (65)
IVB	3 (13)
Bulky disease	
Yes	4 (17)
No	19 (83)
IPI score	
0	1 (4)
1	5 (22)
2	8 (35)
3	5 (22)
4	3 (13)
Not reported	1 (4)
Transformed DLBCL	
Yes	9 (39)
FL	6 (26)
CLL	1 (4)
Indolent NHL	1 (4)
Lymphoplasmacytic lymphoma	1 (4)
No	14 (61)
Cell of origin	
GCB	7 (30)
Unclassified	1 (4)
Unknown	15 (65)

**Characteristics, *n* (%)**	**Part B R/R DLBCL (*N* = 23)**
MYC translocation	
Positive	5 (22)
Negative	8 (35)
Indeterminate	1 (4)
Not done	9 (39)
BCL2 translocation	
Positive	8 (35)
Negative	6 (26)
Not done	8 (35)
BCL6 translocation	
Positive	7 (30)
Negative	7 (30)
Not done	8 (35)
Double-hit lymphoma	
Yes	2 (9)
No	20 (87)
Not reported	1 (4)

CLL, chronic lymphocytic leukemia; DLBCL, diffuse large B-cell lymphoma; FL, follicular lymphoma; GCB, germinal center B-cell like; IPI, international prognostic index; NHL, non-Hodgkin lymphoma; R/R, relapsed/refractory.

Supplementary Table 2 Treatment-related adverse events reported in the treated patient population.

TRAEs, *n* (%)	Part A								Part B R/R DLBCL (*N* = 23)				Part C (*N* = 41)			
	Overall population (*N* = 69)				45 mg 4 days on/24 days off (*n* = 7)											
	Any grade	Grade 3	Grade 4	Grade 5	Any grade	Grade 3	Grade 4	Grade 5	Any grade	Grade 3	Grade 4	Grade 5	Any grade	Grade 3	Grade 4	Grade 5
Any TRAE[a]	60 (87)	18 (26)	5 (7)	0	6 (86)	1 (14)	1 (14)	0	23 (100)	6 (26)	15 (65)	0	40 (98)	23 (56)	1 (2)	0
Thrombocytopenia	32 (46)	7 (10)	2 (3)	0	5 (71)	0	1 (14)	0	21 (91)	4 (17)	14 (61)	0	26 (63)	6 (15)	1 (2)	0
Diarrhea	27 (39)	1 (1)	0	0	3 (43)	0	0	0	12 (52)	0	0	0	25 (61)	2 (5)	0	0
Nausea	27 (39)	0	0	0	2 (29)	0	0	0	5 (22)	1 (4)	0	0	25 (61)	2 (5)	0	0
Stomatitis	16 (23)	0	0	0	3 (43)	0	0	0	4 (17)	0	0	0	23 (56)	2 (5)	0	0
Dysgeusia	18 (26)	0	0	0	1 (14)	0	0	0	2 (9)	0	0	0	22 (54)	0	0	0
Vomiting	12 (17)	0	0	0	1 (14)	0	0	0	4 (17)	0	0	0	18 (44)	1 (2)	0	0
Asthenia	22 (32)	1 (1)	0	0	1 (14)	0	0	0	5 (22)	2 (9)	0	0	17 (41)	3 (7)	0	0
Decreased appetite	12 (17)	0	0	0	0	0	0	0	5 (22)	1 (4)	0	0	14 (34)	0	0	0
Fatigue	15 (22)	2 (3)	0	0	0	0	0	0	0	0	0	0	11 (27)	3 (7)	0	0
ALT increased	5 (7)	1 (1)	0	0	0	0	0	0	2 (9)	0	0	0	9 (22)	1 (2)	0	0
Hyperglycemia	8 (12)	1 (1)	0	0	0	0	0	0	9 (39)	1 (4)	1 (4)	0	8 (20)	2 (5)	0	0
Acneiform dermatitis	9 (13)	0	0	0	0	0	0	0	0	0	0	0	7 (17)	0	0	0
Blood bilirubin increased	1 (1)	0	0	0	0	0	0	0	2 (9)	1 (4)	0	0	6 (15)	0	0	0
Anemia	8 (12)	3 (4)	0	0	2 (29)	1 (14)	0	0	10 (43)	6 (26)	0	0	5 (12)	0	0	0
Neutropenia	5 (7)	1 (1)	0	0	0	0	0	0	9 (39)	3 (13)	3 (13)	0	5 (12)	2 (5)	0	0
AST increased	3 (4)	0	0	0	0	0	0	0	2 (9)	0	0	0	5 (12)	0	0	0
Headache	2 (3)	0	0	0	1 (14)	0	0	0	2 (9)	0	0	0	5 (12)	0	0	0
Dry skin	1 (1)	0	0	0	0	0	0	0	1 (4)	0	0	0	5 (12)	0	0	0
Abdominal pain upper	0	0	0	0	0	0	0	0	0	0	0	0	5 (12)	0	0	0

TRAEs, *n* (%)	Part A Overall population (*N* = 69)				Part A 45 mg 4 days on/24 days off (*n* = 7)				Part B R/R DLBCL (*N* = 23)				Part C (*N* = 41)			
	**Any grade**	**Grade 3**	**Grade 4**	**Grade 5**	**Any grade**	**Grade 3**	**Grade 4**	**Grade 5**	**Any grade**	**Grade 3**	**Grade 4**	**Grade 5**	**Any grade**	**Grade 3**	**Grade 4**	**Grade 5**
Dyspepsia	2 (3)	0	0	0	1 (14)	0	0	0	0	0	0	0	4 (10)	0	0	0
Palmar-plantar erythrodysesthesia syndrome	1 (1)	0	0	0	0	0	0	0	0	0	0	0	4 (10)	0	0	0
Dizziness	0	0	0	0	0	0	0	0	0	0	0	0	4 (10)	0	0	0
Maculopapular rash	5 (7)	0	0	0	1 (14)	0	0	0	0	0	0	0	3 (7)	0	0	0
Weight decreased	1 (1)	0	0	0	0	0	0	0	2 (9)	0	0	0	3 (7)	0	0	0
Pruritis	5 (7)	0	0	0	0	0	0	0	0	0	0	0	2 (5)	0	0	0
Abdominal pain	3 (4)	0	0	0	0	0	0	0	0	0	0	0	2 (5)	0	0	0
Herpes zoster	3 (4)	0	0	0	0	0	0	0	0	0	0	0	2 (5)	0	0	0
Constipation	2 (3)	0	0	0	0	0	0	0	0	0	0	0	2 (5)	0	0	0
Hypophosphatemia	2 (3)	1 (1)	0	0	0	0	0	0	4 (17)	3 (13)	0	0	2 (5)	2 (5)	0	0
Rash	2 (3)	0	0	0	0	0	0	0	0	0	0	0	2 (5)	0	0	0
Dry mouth	1 (1)	0	0	0	0	0	0	0	0	0	0	0	2 (5)	0	0	0
Amylase increased	1 (1)	0	0	0	0	0	0	0	2 (9)	0	0	0	2 (5)	0	0	0
Transaminases increased	1 (1)	0	0	0	0	0	0	0	0	0	0	0	2 (5)	0	0	0
Pharyngeal inflammation	1 (1)	0	0	0	0	0	0	0	0	0	0	0	2 (5)	0	0	0
Hypertension	1 (1)	0	0	0	0	0	0	0	1 (4)	1 (4)	0	0	2 (5)	1 (2)	0	0
GGT increased	0	0	0	0	0	0	0	0	0	0	0	0	2 (5)	1 (2)	0	0
Back pain	0	0	0	0	0	0	0	0	0	0	0	0	2 (5)	0	0	0
Hyperbilirubinemia	4 (6)	0	0	0	0	0	0	0	0	0	0	0	1 (2)	0	0	0
Hyponatremia	3 (4)	1 (1)	1 (1)	0	0	0	0	0	2 (9)	0	0	0	1 (2)	1 (2)	0	0
Insomnia	3 (4)	0	0	0	0	0	0	0	0	0	0	0	1 (2)	0	0	0

TRAEs, *n* (%)	Part A Overall population (*N* = 69)				Part A 45 mg 4 days on/24 days off (*n* = 7)				Part B R/R DLBCL (*N* = 23)				Part C (*N* = 41)			
	**Any grade**	**Grade 3**	**Grade 4**	**Grade 5**	**Any grade**	**Grade 3**	**Grade 4**	**Grade 5**	**Any grade**	**Grade 3**	**Grade 4**	**Grade 5**	**Any grade**	**Grade 3**	**Grade 4**	**Grade 5**
Blood creatinine increased	2 (3)	0	0	0	0	0	0	0	3 (13)	0	1 (4)	0	1 (2)	0	0	0
Arthralgia	1 (1)	0	0	0	0	0	0	0	0	0	0	0	1 (2)	0	0	0
Cough	1 (1)	0	0	0	0	0	0	0	0	0	0	0	1 (2)	0	0	0
Epistaxis	1 (1)	0	0	0	0	0	0	0	0	0	0	0	1 (2)	0	0	0
Abdominal pain lower	0	0	0	0	0	0	0	0	0	0	0	0	1 (2)	0	0	0
Lip dry	0	0	0	0	0	0	0	0	0	0	0	0	1 (2)	0	0	0
Lip ulceration	0	0	0	0	0	0	0	0	0	0	0	0	1 (2)	0	0	0
Odynophagia	0	0	0	0	0	0	0	0	0	0	0	0	1 (2)	0	0	0
Esophagitis	0	0	0	0	0	0	0	0	0	0	0	0	1 (2)	0	0	0
Pyrexia	0	0	0	0	0	0	0	0	1 (4)	0	0	0	1 (2)	0	0	0
Edema peripheral	0	0	0	0	0	0	0	0	0	0	0	0	1 (2)	0	0	0
Xerosis	0	0	0	0	0	0	0	0	0	0	0	0	1 (2)	0	0	0
Hyperamylasemia	0	0	0	0	0	0	0	0	0	0	0	0	1 (2)	1 (2)	0	0
Butterfly rash	0	0	0	0	0	0	0	0	0	0	0	0	1 (2)	0	0	0
Rash erythematous	0	0	0	0	0	0	0	0	0	0	0	0	1 (2)	0	0	0
Skin fissures	0	0	0	0	0	0	0	0	0	0	0	0	1 (2)	0	0	0
Skin fragility	0	0	0	0	0	0	0	0	0	0	0	0	1 (2)	0	0	0
Blood ALP increased	0	0	0	0	0	0	0	0	1 (4)	0	0	0	1 (2)	0	0	0
Syncope	0	0	0	0	0	0	0	0	0	0	0	0	1 (2)	1 (2)	0	0
Eyelid infection	0	0	0	0	0	0	0	0	0	0	0	0	1 (2)	0	0	0
Mucosal infection	0	0	0	0	0	0	0	0	0	0	0	0	1 (2)	0	0	0
Oral herpes	0	0	0	0	0	0	0	0	0	0	0	0	1 (2)	0	0	0

TRAEs, *n* (%)	Part A Overall population (*N* = 69) Any grade	Grade 3	Grade 4	Grade 5	Part A 45 mg 4 days on/24 days off (*n* = 7) Any grade	Grade 3	Grade 4	Grade 5	Part B R/R DLBCL (*N* = 23) Any grade	Grade 3	Grade 4	Grade 5	Part C (*N* = 41) Any grade	Grade 3	Grade 4	Grade 5
Pain in extremity	0	0	0	0	0	0	0	0	1 (4)	0	0	0	1 (2)	0	0	0
Dyspnea	0	0	0	0	0	0	0	0	1 (4)	0	0	0	1 (2)	0	0	0
Dyspnea exertional	0	0	0	0	0	0	0	0	0	0	0	0	1 (2)	0	0	0
Nasal inflammation	0	0	0	0	0	0	0	0	0	0	0	0	1 (2)	0	0	0
Productive cough	0	0	0	0	0	0	0	0	0	0	0	0	1 (2)	0	0	0
Erectile dysfunction	0	0	0	0	0	0	0	0	0	0	0	0	1 (2)	0	0	0
Conjunctival hyperemia	0	0	0	0	0	0	0	0	0	0	0	0	1 (2)	0	0	0
Eyelid edema	0	0	0	0	0	0	0	0	0	0	0	0	1 (2)	0	0	0
Lacrimation increased	0	0	0	0	0	0	0	0	0	0	0	0	1 (2)	0	0	0
Periorbital edema	0	0	0	0	0	0	0	0	0	0	0	0	1 (2)	0	0	0
Photophobia	0	0	0	0	0	0	0	0	0	0	0	0	1 (2)	0	0	0
Vision blurred	0	0	0	0	0	0	0	0	0	0	0	0	1 (2)	0	0	0
Gilbert's syndrome	0	0	0	0	0	0	0	0	0	0	0	0	1 (2)	0	0	0
Cheilitis	1 (1)	0	0	0	0	0	0	0	2 (9)	0	0	0	0	0	0	0
Lymphopenia	1 (1)	0	0	0	0	0	0	0	1 (4)	0	1 (4)	0	0	0	0	0
Hypokalemia	1 (1)	1 (1)	0	0	0	0	0	0	1 (4)	0	0	0	0	0	0	0
Pneumonia	1 (1)	0	0	0	0	0	0	0	1 (4)	1 (4)	0	0	0	0	0	0
Acute kidney injury	1 (1)	0	0	0	0	0	0	0	1 (4)	0	0	0	0	0	0	0
Enterocolitis	0	0	0	0	0	0	0	0	1 (4)	0	0	0	0	0	0	0
Leukopenia	0	0	0	0	0	0	0	0	2 (9)	0	1 (4)	0	0	0	0	0
Febrile neutropenia	0	0	0	0	0	0	0	0	1 (4)	0	1 (4)	0	0	0	0	0
Malaise	0	0	0	0	0	0	0	0	2 (9)	0	0	0	0	0	0	0

TRAEs, *n* (%)	Part A								Part B R/R DLBCL (*N* = 23)				Part C (*N* = 41)			
	Overall population (*N* = 69)				45 mg 4 days on/24 days off (*n* = 7)											
	Any grade	Grade 3	Grade 4	Grade 5	Any grade	Grade 3	Grade 4	Grade 5	Any grade	Grade 3	Grade 4	Grade 5	Any grade	Grade 3	Grade 4	Grade 5
Mucosal inflammation	0	0	0	0	0	0	0	0	0	0	0	0	0	0	0	0
Dehydration	0	0	0	0	0	0	0	0	3 (13)	3 (13)	0	0	0	0	0	0
Hypomagnesemia	0	0	0	0	0	0	0	0	3 (13)	0	0	0	0	0	0	0
ECG QT prolonged	0	0	0	0	0	0	0	0	3 (13)	0	0	0	0	0	0	0
INR increased	0	0	0	0	0	0	0	0	2 (9)	0	0	0	0	0	0	0
Lymphocyte count decreased	0	0	0	0	0	0	0	0	1 (4)	0	0	0	0	0	0	0
Abdominal infection	0	0	0	0	0	0	0	0	1 (4)	1 (4)	0	0	0	0	0	0
Factor VII deficiency	0	0	0	0	0	0	0	0	1 (4)	0	0	0	0	0	0	0
Cardiac failure	0	0	0	0	0	0	0	0	1 (4)	0	0	0	0	0	0	0
Platelet count decreased	3 (4)	1 (1)	1 (1)	0	0	0	0	0	0	0	0	0	0	0	0	0
Ejection fraction decreased	2 (3)	0	0	0	0	0	0	0	0	0	0	0	0	0	0	0
Conjunctivitis	2 (3)	0	0	0	0	0	0	0	0	0	0	0	0	0	0	0
Musculoskeletal chest pain	2 (3)	0	0	0	0	0	0	0	0	0	0	0	0	0	0	0
Mucosal dryness	1 (1)	0	0	0	1 (14)	0	0	0	0	0	0	0	0	0	0	0
Edema	1 (1)	0	0	0	0	0	0	0	0	0	0	0	0	0	0	0
Diabetes mellitus	1 (1)	0	1 (1)	0	0	0	0	0	0	0	0	0	0	0	0	0
Onycholysis	1 (1)	0	0	0	1 (14)	0	0	0	0	0	0	0	0	0	0	0
Skin burning sensation	1 (1)	0	0	0	0	0	0	0	0	0	0	0	0	0	0	0
Skin hemorrhage	1 (1)	1 (1)	0	0	0	0	0	0	0	0	0	0	0	0	0	0
Urticaria	1 (1)	0	0	0	0	0	0	0	0	0	0	0	0	0	0	0
Blood creatine phosphokinase increased	1 (1)	0	1 (1)	0	0	0	0	0	0	0	0	0	0	0	0	0

TRAEs, *n* (%)	Part A: Overall population (*N* = 69) Any grade	Grade 3	Grade 4	Grade 5	Part A: 45 mg 4 days on/24 days off (*n* = 7) Any grade	Grade 3	Grade 4	Grade 5	Part B R/R DLBCL (*N* = 23) Any grade	Grade 3	Grade 4	Grade 5	Part C (*N* = 41) Any grade	Grade 3	Grade 4	Grade 5
Lipase increased	1 (1)	1 (1)	0	0	0	0	0	0	0	0	0	0	0	0	0	0
Liver function test abnormal	1 (1)	1 (1)	0	0	0	0	0	0	0	0	0	0	0	0	0	0
Presyncope	1 (1)	1 (1)	0	0	0	0	0	0	0	0	0	0	0	0	0	0
Bronchitis	1 (1)	0	0	0	1 (14)	0	0	0	0	0	0	0	0	0	0	0
Herpes simplex	1 (1)	0	0	0	0	0	0	0	0	0	0	0	0	0	0	0
Influenza	1 (1)	0	0	0	0	0	0	0	0	0	0	0	0	0	0	0
Lip infection	1 (1)	0	0	0	0	0	0	0	0	0	0	0	0	0	0	0
Vulvitis	1 (1)	0	0	0	0	0	0	0	0	0	0	0	0	0	0	0
Muscle spasms	1 (1)	0	0	0	0	0	0	0	0	0	0	0	0	0	0	0
Anxiety	1 (1)	0	0	0	0	0	0	0	0	0	0	0	0	0	0	0
Inappropriate antidiuretic hormone secretion	1 (1)	0	1 (1)	0	0	0	0	0	0	0	0	0	0	0	0	0
Vaginal hemorrhage	1 (1)	0	0	0	0	0	0	0	0	0	0	0	0	0	0	0
Essential hypertension	1 (1)	1 (1)	0	0	0	0	0	0	0	0	0	0	0	0	0	0

[a]Any-grade TRAEs were reported in both the patient with BCC and the patient with NUT carcinoma enrolled in part B. Neither patient had a grade 3/4 TRAE

ALT, alanine aminotransferase; AST, aspartate aminotransferase; BCC, basal cell carcinoma; DLBCL, diffuse large B-cell lymphoma; ECG, electrocardiogram; INR, international normalized ratio; NUT, nuclear protein in testis; R/R, relapsed/refractory; TRAE, treatment-related adverse event.

Supplementary Table 3 Treatment-emergent adverse events reported in the treated patient population.

	Part A (*N* = 69)				Part B R/R DLBCL (*N* = 23)				Part C (*N* = 41)			
**TEAEs, *n* (%)**	**Any grade**	**Grade 3**	**Grade 4**	**Grade 5**	**Any grade**	**Grade 3**	**Grade 4**	**Grade 5**	**Any grade**	**Grade 3**	**Grade 4**	**Grade 5**
Any TEAE[a]	69 (100)	30 (43)	10 (14)	7 (10)	23 (100)	6 (26)	16 (70)	2 (9)	41 (100)	28 (68)	4 (10)	1 (2)
Thrombocytopenia	35 (51)	7 (10)	2 (3)	0	22 (96)	4 (17)	15 (65)	0	28 (68)	7 (17)	1 (2)	0
Diarrhea	29 (42)	1 (1)	0	0	14 (61)	0	0	0	25 (61)	2 (5)	0	0
Nausea	28 (41)	0	0	0	10 (43)	1 (4)	0	0	25 (61)	2 (5)	0	0
Stomatitis	17 (25)	0	0	0	5 (22)	0	0	0	25 (61)	2 (5)	0	0
Dysgeusia	20 (29)	0	0	0	2 (9)	0	0	0	23 (56)	0	0	0
Asthenia	27 (39)	4 (6)	0	0	9 (39)	2 (9)	0	0	18 (44)	4 (10)	0	0
Vomiting	17 (25)	0	0	0	5 (22)	0	0	0	18 (44)	1 (2)	0	0
Decreased appetite	17 (25)	0	0	0	6 (26)	2 (9)	0	0	16 (39)	0	0	0
Fatigue	17 (25)	2 (3)	0	0	1 (4)	0	0	0	13 (32)	3 (7)	0	0
Hyperglycemia	15 (22)	4 (6)	0	0	10 (43)	1 (4)	1 (4)	0	10 (24)	2 (5)	0	0
ALT increased	8 (12)	2 (3)	0	0	4 (17)	0	0	0	9 (22)	1 (2)	0	0
Blood bilirubin increased	1 (1)	0	0	0	4 (17)	2 (9)	0	0	9 (22)	0	0	0
Acneiform dermatitis	9 (13)	0	0	0	0	0	0	0	8 (20)	0	0	0
Anemia	11 (16)	3 (4)	0	0	17 (74)	9 (39)	0	0	7 (17)	0	0	0
Constipation	9 (13)	0	0	0	1 (4)	0	0	0	7 (17)	0	0	0
Headache	7 (10)	1 (1)	0	0	3 (13)	0	0	0	6 (15)	0	0	0
Dyspnea	6 (9)	2 (3)	0	0	3 (13)	0	0	0	6 (15)	0	0	0
Back pain	6 (9)	2 (3)	0	0	1 (4)	0	0	0	6 (15)	0	0	0
Neutropenia	5 (7)	1 (1)	0	0	12 (52)	5 (22)	3 (13)	0	6 (15)	2 (5)	0	0
Dizziness	0	0	0	0	0	0	0	0	6 (15)	0	0	0
AST increased	5 (7)	1 (1)	0	0	3 (13)	0	0	0	5 (12)	0	0	0
Cough	4 (6)	0	0	0	3 (13)	0	0	0	5 (12)	0	0	0
Dyspepsia	2 (3)	0	0	0	0	0	0	0	5 (12)	0	0	0
Dry skin	1 (1)	0	0	0	2 (9)	0	0	0	5 (12)	0	0	0
Abdominal pain upper	1 (1)	0	0	0	0	0	0	0	5 (12)	0	0	0
Pyrexia	7 (10)	1 (1)	0	0	7 (30)	1 (4)	0	0	4 (10)	0	0	0
Weight decreased	4 (6)	0	0	0	3 (13)	0	0	0	4 (10)	0	0	0

TEAEs, *n* (%)	Part A (*N* = 69)				Part B R/R DLBCL (*N* = 23)				Part C (*N* = 41)			
	Any grade	Grade 3	Grade 4	Grade 5	Any grade	Grade 3	Grade 4	Grade 5	Any grade	Grade 3	Grade 4	Grade 5
Hypertension	2 (3)	0	0	0	4 (17)	2 (9)	0	0	4 (10)	1 (2)	0	0
Palmar-plantar erythrodysesthesia syndrome	1 (1)	0	0	0	0	0	0	0	4 (10)	0	0	0
Abdominal pain	10 (14)	1 (1)	0	0	0	0	0	0	3 (7)	0	0	0
Maculopapular rash	7 (10)	0	0	0	0	0	0	0	3 (7)	0	0	0
Upper respiratory tract infection	6 (9)	1 (1)	0	0	0	0	0	0	3 (7)	0	0	0
Blood creatinine increased	4 (6)	1 (1)	0	0	4 (17)	0	1 (4)	0	3 (7)	1 (2)	0	0
Insomnia	4 (6)	0	0	0	1 (4)	0	0	0	3 (7)	0	0	0
Hyponatremia	3 (4)	1 (1)	1 (1)	0	4 (17)	0	0	0	3 (7)	1 (2)	1 (2)	0
Herpes zoster	3 (4)	0	0	0	3 (13)	0	0	0	3 (7)	0	0	0
Tumor pain	2 (3)	0	0	0	1 (4)	0	0	0	3 (7)	0	0	0
Rash	2 (3)	0	0	0	2 (9)	0	0	0	3 (7)	0	0	0
Edema peripheral	1 (1)	0	0	0	0	0	0	0	3 (7)	0	0	0
GGT increased	0	0	0	0	1 (4)	0	0	0	3 (7)	1 (2)	0	0
Urinary tract infection	5 (7)	0	1 (1)	0	1 (4)	0	0	0	2 (5)	0	0	0
Pruritis	5 (7)	0	0	0	1 (4)	0	0	0	2 (5)	0	0	0
Transaminases increased	5 (7)	0	0	0	0	0	0	0	2 (5)	0	0	0
Arthralgia	4 (6)	3 (4)	0	0	0	0	0	0	2 (5)	1 (2)	0	0
Performance status decreased	3 (4)	2 (3)	0	1 (1)	0	0	0	0	2 (5)	1 (2)	1 (2)	0
Hypophosphatemia	2 (3)	1 (1)	0	0	6 (26)	4 (17)	0	0	2 (5)	2 (5)	0	0
Dry mouth	2 (3)	0	0	0	0	0	0	0	2 (5)	0	0	0
Conjunctivitis	2 (3)	0	0	0	0	0	0	0	2 (5)	0	0	0
Diabetes mellitus	1 (1)	0	1 (1)	0	0	0	0	0	2 (5)	0	1 (2)	0
Dehydration	1 (1)	0	0	0	4 (17)	4 (17)	0	0	2 (5)	0	0	0
Amylase increased	1 (1)	0	0	0	4 (17)	0	1 (4)	0	2 (5)	0	0	0
Epistaxis	1 (1)	0	0	0	1 (4)	0	0	0	2 (5)	0	0	0
Gait disturbance	1 (1)	0	0	0	0	0	0	0	2 (5)	0	0	0
Hemiparesis	1 (1)	0	0	0	0	0	0	0	2 (5)	0	0	0
Respiratory tract infection	1 (1)	0	0	0	0	0	0	0	2 (5)	0	0	0
Pharyngeal inflammation	1 (1)	0	0	0	0	0	0	0	2 (5)	0	0	0

TEAEs, *n* (%)	Part A (*N* = 69) Any grade	Grade 3	Grade 4	Grade 5	Part B R/R DLBCL (*N* = 23) Any grade	Grade 3	Grade 4	Grade 5	Part C (*N* = 41) Any grade	Grade 3	Grade 4	Grade 5
Anxiety	1 (1)	0	0	0	0	0	0	0	2 (5)	0	0	0
Hypotension	0	0	0	0	1 (4)	0	1 (4)	0	2 (5)	0	0	0
Chest pain	0	0	0	0	1 (4)	0	0	0	2 (5)	0	0	0
Skin lesion	0	0	0	0	1 (4)	0	0	0	2 (5)	0	0	0
Esophagitis	0	0	0	0	0	0	0	0	2 (5)	0	0	0
COVID-19	0	0	0	0	0	0	0	0	2 (5)	0	0	0
Oral herpes	0	0	0	0	0	0	0	0	2 (5)	0	0	0
Pharyngitis	0	0	0	0	0	0	0	0	2 (5)	0	0	0
Hyperbilirubinemia	6 (9)	1 (1)	0	0	1 (4)	0	0	0	1 (2)	0	0	0
Pneumonia	4 (6)	1 (1)	0	0	1 (4)	1 (4)	0	0	1 (2)	0	0	0
General physical health deterioration	3 (4)	1 (1)	0	2 (3)	0	0	0	0	1 (2)	1 (2)	0	0
Vision blurred	3 (4)	0	0	0	0	0	0	0	1 (2)	0	0	0
Somnolence	2 (3)	0	0	0	0	0	0	0	1 (2)	0	0	0
Influenza	2 (3)	0	0	0	0	0	0	0	1 (2)	0	0	0
Musculoskeletal chest pain	2 (3)	0	0	0	0	0	0	0	1 (2)	0	0	0
Hypomagnesemia	1 (1)	0	0	0	8 (35)	2 (9)	0	0	1 (2)	0	0	0
Abdominal pain lower	1 (1)	0	0	0	1 (4)	0	0	0	1 (2)	0	0	0
Odynophagia	1 (1)	0	0	0	1 (4)	0	0	0	1 (2)	0	0	0
Nervous system disorder	1 (1)	1 (1)	0	0	0	0	0	0	1 (2)	0	0	0
Partial seizures	1 (1)	0	0	0	0	0	0	0	1 (2)	0	0	0
Peripheral sensory neuropathy	1 (1)	0	0	0	0	0	0	0	1 (2)	0	0	0
Presyncope	1 (1)	1 (1)	0	0	0	0	0	0	1 (2)	0	0	0
Mucosal infection	1 (1)	0	0	0	0	0	0	0	1 (2)	0	0	0
Exertional dyspnea	1 (1)	0	0	0	0	0	0	0	1 (2)	0	0	0
Pleuritic pain	1 (1)	0	0	0	0	0	0	0	1 (2)	0	0	0
Diplopia	1 (1)	0	0	0	0	0	0	0	1 (2)	0	0	0
Inappropriate antidiuretic hormone secretion	1 (1)	0	1 (1)	0	0	0	0	0	1 (2)	0	0	0
Atrial fibrillation	0	0	0	0	2 (9)	0	0	0	1 (2)	1 (2)	0	0
Hyperamylasemia	0	0	0	0	0	0	0	0	1 (2)	1 (2)	0	0
Cerebrovascular accident	0	0	0	0	0	0	0	0	1 (2)	1 (2)	0	1 (2)

TEAEs, *n* (%)	Part A (*N* = 69) Any grade	Grade 3	Grade 4	Grade 5	Part B R/R DLBCL (*N* = 23) Any grade	Grade 3	Grade 4	Grade 5	Part C (*N* = 41) Any grade	Grade 3	Grade 4	Grade 5
Syncope	0	0	0	0	0	0	0	0	1 (2)	1 (2)	0	0
Diverticulitis	0	0	0	0	0	0	0	0	1 (2)	1 (2)	0	0
Skin pain	0	0	0	0	0	0	0	0	1 (2)	1 (2)	0	0
Glioblastoma	0	0	0	0	0	0	0	0	1 (2)	1 (2)	0	0
Metastatic neoplasm	0	0	0	0	0	0	0	0	1 (2)	1 (2)	0	0
Urinary tract obstruction	0	0	0	0	0	0	0	0	1 (2)	1 (2)	0	0
Osteomyelitis	0	0	0	0	0	0	0	0	1 (2)	0	1 (2)	0
ECG QT prolonged	0	0	0	0	3 (13)	0	0	0	1 (2)	0	0	0
Blood ALP increased	0	0	0	0	1 (4)	0	0	0	1 (2)	0	0	0
Oropharyngeal pain	0	0	0	0	1 (4)	0	0	0	1 (2)	0	0	0
Productive cough	0	0	0	0	1 (4)	0	0	0	1 (2)	0	0	0
Pain in extremity	0	0	0	0	1 (4)	0	0	0	1 (2)	0	0	0
Lip dry	0	0	0	0	0	0	0	0	1 (2)	0	0	0
Lip ulceration	0	0	0	0	0	0	0	0	1 (2)	0	0	0
Salivary hypersecretion	0	0	0	0	0	0	0	0	1 (2)	0	0	0
Face edema	0	0	0	0	0	0	0	0	1 (2)	0	0	0
Xerosis	0	0	0	0	0	0	0	0	1 (2)	0	0	0
Hyperuricemia	0	0	0	0	0	0	0	0	1 (2)	0	0	0
Hypoglycemia	0	0	0	0	0	0	0	0	1 (2)	0	0	0
Alexia	0	0	0	0	0	0	0	0	1 (2)	0	0	0
Gait apraxia	0	0	0	0	0	0	0	0	1 (2)	0	0	0
Neuralgia	0	0	0	0	0	0	0	0	1 (2)	0	0	0
Trigeminal neuralgia	0	0	0	0	0	0	0	0	1 (2)	0	0	0
Eyelid infection	0	0	0	0	0	0	0	0	1 (2)	0	0	0
Furuncle	0	0	0	0	0	0	0	0	1 (2)	0	0	0
Paronychia	0	0	0	0	0	0	0	0	1 (2)	0	0	0
Blood LDH increased	0	0	0	0	0	0	0	0	1 (2)	0	0	0
Butterfly rash	0	0	0	0	0	0	0	0	1 (2)	0	0	0
Intertrigo	0	0	0	0	0	0	0	0	1 (2)	0	0	0
Rash erythematous	0	0	0	0	0	0	0	0	1 (2)	0	0	0

TEAEs, *n* (%)	Part A (*N* = 69)				Part B R/R DLBCL (*N* = 23)				Part C (*N* = 41)			
	Any grade	Grade 3	Grade 4	Grade 5	Any grade	Grade 3	Grade 4	Grade 5	Any grade	Grade 3	Grade 4	Grade 5
Seborrheic dermatitis	0	0	0	0	0	0	0	0	1 (2)	0	0	0
Skin fissures	0	0	0	0	0	0	0	0	1 (2)	0	0	0
Skin fragility	0	0	0	0	0	0	0	0	1 (2)	0	0	0
Asthma	0	0	0	0	0	0	0	0	1 (2)	0	0	0
Nasal inflammation	0	0	0	0	0	0	0	0	1 (2)	0	0	0
Bone pain	0	0	0	0	0	0	0	0	1 (2)	0	0	0
Muscle contracture	0	0	0	0	0	0	0	0	1 (2)	0	0	0
Pain in jaw	0	0	0	0	0	0	0	0	1 (2)	0	0	0
Spinal pain	0	0	0	0	0	0	0	0	1 (2)	0	0	0
Confusional state	0	0	0	0	0	0	0	0	1 (2)	0	0	0
Nervousness	0	0	0	0	0	0	0	0	1 (2)	0	0	0
Skin cancer	0	0	0	0	0	0	0	0	1 (2)	0	0	0
Bladder discomfort	0	0	0	0	0	0	0	0	1 (2)	0	0	0
Polyuria	0	0	0	0	0	0	0	0	1 (2)	0	0	0
Conjunctival hyperemia	0	0	0	0	0	0	0	0	1 (2)	0	0	0
Eyelid edema	0	0	0	0	0	0	0	0	1 (2)	0	0	0
Lacrimation increased	0	0	0	0	0	0	0	0	1 (2)	0	0	0
Periorbital edema	0	0	0	0	0	0	0	0	1 (2)	0	0	0
Photophobia	0	0	0	0	0	0	0	0	1 (2)	0	0	0
Erectile dysfunction	0	0	0	0	0	0	0	0	1 (2)	0	0	0
Gilbert’s syndrome	0	0	0	0	0	0	0	0	1 (2)	0	0	0
Agitation	3 (4)	0	0	0	1 (4)	0	0	0	0	0	0	0
Hypokalemia	2 (3)	2 (3)	0	0	3 (13)	0	0	0	0	0	0	0
Acute kidney injury	1 (1)	0	0	0	2 (9)	1 (4)	0	0	0	0	0	0
Lymphopenia	1 (1)	0	0	0	1 (4)	0	1 (4)	0	0	0	0	0
Cerebral hemorrhage	1 (1)	0	0	1 (1)	1 (4)	0	1 (4)	1 (4)	0	0	0	0
Cheilitis	1 (1)	0	0	0	2 (9)	0	0	0	0	0	0	0
Dysphagia	1 (1)	0	0	0	1 (4)	0	0	0	0	0	0	0
Bronchitis	1 (1)	0	0	0	1 (4)	0	0	0	0	0	0	0
Gingivitis	1 (1)	0	0	0	1 (4)	0	0	0	0	0	0	0

TEAEs, *n* (%)	Part A (*N* = 69) Any grade	Grade 3	Grade 4	Grade 5	Part B R/R DLBCL (*N* = 23) Any grade	Grade 3	Grade 4	Grade 5	Part C (*N* = 41) Any grade	Grade 3	Grade 4	Grade 5
Herpes simplex	1 (1)	0	0	0	1 (4)	0	0	0	0	0	0	0
Laryngitis	1 (1)	0	0	0	1 (4)	0	0	0	0	0	0	0
Lipase increased	1 (1)	1 (1)	0	0	1 (4)	0	0	0	0	0	0	0
Atrial flutter	1 (1)	0	0	0	1 (4)	0	0	0	0	0	0	0
Supraventricular extrasystoles	1 (1)	0	0	0	1 (4)	0	0	0	0	0	0	0
Hyperthyroidism	1 (1)	0	0	0	1 (4)	0	0	0	0	0	0	0
Leukopenia	0	0	0	0	2 (9)	0	1 (4)	0	0	0	0	0
Gastrointestinal hemorrhage	0	0	0	0	1 (4)	1 (4)	0	0	0	0	0	0
Febrile neutropenia	0	0	0	0	1 (4)	0	1 (4)	0	0	0	0	0
Abdominal infection	0	0	0	0	1 (4)	1 (4)	0	0	0	0	0	0
Infection	0	0	0	0	1 (4)	1 (4)	0	0	0	0	0	0
Ophthalmic herpes zoster	0	0	0	0	1 (4)	1 (4)	0	0	0	0	0	0
Post procedural hemorrhage	0	0	0	0	1 (4)	1 (4)	0	0	0	0	0	0
Hypocalcemia	0	0	0	0	4 (17)	0	0	0	0	0	0	0
Malaise	0	0	0	0	3 (13)	0	0	0	0	0	0	0
INR increased	0	0	0	0	2 (9)	0	0	0	0	0	0	0
Aphthous ulcer	0	0	0	0	1 (4)	0	0	0	0	0	0	0
Enterocolitis	0	0	0	0	1 (4)	0	0	0	0	0	0	0
Axillary pain	0	0	0	0	1 (4)	0	0	0	0	0	0	0
Hypercalcemia	0	0	0	0	1 (4)	0	0	0	0	0	0	0
Hyperchloremia	0	0	0	0	1 (4)	0	0	0	0	0	0	0
Hypoalbuminemia	0	0	0	0	1 (4)	0	0	0	0	0	0	0
Hypochloremia	0	0	0	0	1 (4)	0	0	0	0	0	0	0
Apraxia	0	0	0	0	1 (4)	0	0	0	0	0	0	0
Post herpetic neuralgia	0	0	0	0	1 (4)	0	0	0	0	0	0	0
Oral fungal infection	0	0	0	0	1 (4)	0	0	0	0	0	0	0
Lymphocyte count decreased	0	0	0	0	1 (4)	0	0	0	0	0	0	0
Allergic dermatitis	0	0	0	0	1 (4)	0	0	0	0	0	0	0
Petechiae	0	0	0	0	1 (4)	0	0	0	0	0	0	0
Psoriasis	0	0	0	0	1 (4)	0	0	0	0	0	0	0

TEAEs, *n* (%)	Part A (*N* = 69)				Part B R/R DLBCL (*N* = 23)				Part C (*N* = 41)			
	Any grade	Grade 3	Grade 4	Grade 5	Any grade	Grade 3	Grade 4	Grade 5	Any grade	Grade 3	Grade 4	Grade 5
Acute respiratory failure	0	0	0	0	1 (4)	0	0	1 (4)	0	0	0	0
Pleural effusion	0	0	0	0	1 (4)	0	0	0	0	0	0	0
Tachypnoea	0	0	0	0	1 (4)	0	0	0	0	0	0	0
Cholestasis	0	0	0	0	1 (4)	0	0	0	0	0	0	0
Hepatic cytolysis	0	0	0	0	1 (4)	0	0	0	0	0	0	0
Benign vulval neoplasm	0	0	0	0	1 (4)	0	0	0	0	0	0	0
Cardiac failure	0	0	0	0	1 (4)	0	0	0	0	0	0	0
Sinus tachycardia	0	0	0	0	1 (4)	0	0	0	0	0	0	0
Factor VII deficiency	0	0	0	0	1 (4)	0	0	0	0	0	0	0
Mucosal inflammation	0	0	0	0	0	0	0	0	0	0	0	0
Cognitive disorder	3 (4)	2 (3)	0	0	0	0	0	0	0	0	0	0
Platelet count decreased	3 (4)	1 (1)	1 (1)	0	0	0	0	0	0	0	0	0
Non-cardiac chest pain	2 (3)	1 (1)	0	0	0	0	0	0	0	0	0	0
Tremor	2 (3)	0	0	0	0	0	0	0	0	0	0	0
Tooth infection	2 (3)	0	0	0	0	0	0	0	0	0	0	0
Ejection fraction decreased	2 (3)	0	0	0	0	0	0	0	0	0	0	0
Hyperhidrosis	2 (3)	0	0	0	0	0	0	0	0	0	0	0
Urticaria	2 (3)	0	0	0	0	0	0	0	0	0	0	0
Dysphonia	2 (3)	0	0	0	0	0	0	0	0	0	0	0
Cancer pain	2 (3)	0	0	0	0	0	0	0	0	0	0	0
Oliguria	2 (3)	1 (1)	0	0	0	0	0	0	0	0	0	0
Ascites	1 (1)	1 (1)	0	0	0	0	0	0	0	0	0	0
Intestinal obstruction	1 (1)	1 (1)	0	0	0	0	0	0	0	0	0	0
Intestinal pseudo-obstruction	1 (1)	1 (1)	0	0	0	0	0	0	0	0	0	0
Facial pain	1 (1)	0	0	0	0	0	0	0	0	0	0	0
Influenza-like illness	1 (1)	0	0	0	0	0	0	0	0	0	0	0
Mucosal dryness	1 (1)	0	0	0	0	0	0	0	0	0	0	0
Edema	1 (1)	0	0	0	0	0	0	0	0	0	0	0
Coagulopathy	1 (1)	0	0	0	0	0	0	0	0	0	0	0
Lymph node pain	1 (1)	0	0	0	0	0	0	0	0	0	0	0

TEAEs, *n* (%)	Part A (*N* = 69)				Part B R/R DLBCL (*N* = 23)				Part C (*N* = 41)			
	Any grade	Grade 3	Grade 4	Grade 5	Any grade	Grade 3	Grade 4	Grade 5	Any grade	Grade 3	Grade 4	Grade 5
Hyperkalemia	1 (1)	1 (1)	0	0	0	0	0	0	0	0	0	0
Altered state of consciousness	1 (1)	0	0	0	0	0	0	0	0	0	0	0
Intracranial hemorrhage	1 (1)	0	1 (1)	0	0	0	0	0	0	0	0	0
Myasthenia gravis	1 (1)	1 (1)	0	0	0	0	0	0	0	0	0	0
Orthostatic intolerance	1 (1)	0	0	0	0	0	0	0	0	0	0	0
Peripheral sensorimotor neuropathy	1 (1)	0	0	0	0	0	0	0	0	0	0	0
Bacteriuria	1 (1)	0	0	0	0	0	0	0	0	0	0	0
Cellulitis	1 (1)	0	1 (1)	0	0	0	0	0	0	0	0	0
Gastroenteritis	1 (1)	0	0	0	0	0	0	0	0	0	0	0
Gastroenteritis viral	1 (1)	0	0	0	0	0	0	0	0	0	0	0
Gastrointestinal viral infection	1 (1)	0	0	0	0	0	0	0	0	0	0	0
Kidney infection	1 (1)	1 (1)	0	0	0	0	0	0	0	0	0	0
Lip infection	1 (1)	0	0	0	0	0	0	0	0	0	0	0
Nasopharyngitis	1 (1)	0	0	0	0	0	0	0	0	0	0	0
Oral candidiasis	1 (1)	1 (1)	0	0	0	0	0	0	0	0	0	0
Pleural infection	1 (1)	1 (1)	0	0	0	0	0	0	0	0	0	0
Pneumocystis jirovecii pneumonia	1 (1)	1 (1)	0	1 (1)	0	0	0	0	0	0	0	0
Skin infection	1 (1)	0	0	0	0	0	0	0	0	0	0	0
Vulvitis	1 (1)	0	0	0	0	0	0	0	0	0	0	0
Blood creatine phosphokinase increased	1 (1)	0	1 (1)	0	0	0	0	0	0	0	0	0
Liver function test abnormal	1 (1)	1 (1)	0	0	0	0	0	0	0	0	0	0
Dermatitis	1 (1)	0	0	0	0	0	0	0	0	0	0	0
Atopic dermatitis	1 (1)	0	0	0	0	0	0	0	0	0	0	0
Dermatosis	1 (1)	0	0	0	0	0	0	0	0	0	0	0
Erythema	1 (1)	0	0	0	0	0	0	0	0	0	0	0
Onycholsis	1 (1)	0	0	0	0	0	0	0	0	0	0	0
Skin burning sensation	1 (1)	0	0	0	0	0	0	0	0	0	0	0
Skin hemorrhage	1 (1)	1 (1)	0	0	0	0	0	0	0	0	0	0
Aphonia	1 (1)	1 (1)	0	0	0	0	0	0	0	0	0	0
Bronchial secretion retention	1 (1)	0	0	0	0	0	0	0	0	0	0	0

TEAEs, *n* (%)	Part A (*N* = 69) Any grade	Grade 3	Grade 4	Grade 5	Part B R/R DLBCL (*N* = 23) Any grade	Grade 3	Grade 4	Grade 5	Part C (*N* = 41) Any grade	Grade 3	Grade 4	Grade 5
Bronchospasm	1 (1)	0	0	0	0	0	0	0	0	0	0	0
Choking	1 (1)	0	0	0	0	0	0	0	0	0	0	0
Hemoptysis	1 (1)	0	0	0	0	0	0	0	0	0	0	0
Pneumonitis	1 (1)	0	0	0	0	0	0	0	0	0	0	0
Pulmonary edema	1 (1)	0	1 (1)	0	0	0	0	0	0	0	0	0
Respiratory failure	1 (1)	0	0	1 (1)	0	0	0	0	0	0	0	0
Muscle spasms	1 (1)	0	0	0	0	0	0	0	0	0	0	0
Osteonecrosis of jaw	1 (1)	0	0	0	0	0	0	0	0	0	0	0
Delirium	1 (1)	0	0	0	0	0	0	0	0	0	0	0
Depressed mood	1 (1)	0	0	0	0	0	0	0	0	0	0	0
Depression	1 (1)	0	0	0	0	0	0	0	0	0	0	0
Cholangitis	1 (1)	0	0	0	0	0	0	0	0	0	0	0
Metastases to bone	1 (1)	1 (1)	0	0	0	0	0	0	0	0	0	0
NUT midline carcinoma	1 (1)	0	0	1 (1)	0	0	0	0	0	0	0	0
Hematuria	1 (1)	0	0	0	0	0	0	0	0	0	0	0
Urinary retention	1 (1)	0	0	0	0	0	0	0	0	0	0	0
Pericardial effusion	1 (1)	0	1 (1)	0	0	0	0	0	0	0	0	0
Supraventricular tachycardia	1 (1)	0	0	0	0	0	0	0	0	0	0	0
Keratitis	1 (1)	0	0	0	0	0	0	0	0	0	0	0
Visual acuity reduced	1 (1)	0	0	0	0	0	0	0	0	0	0	0
Essential hypertension	1 (1)	1 (1)	0	0	0	0	0	0	0	0	0	0
Superior vena cava syndrome	1 (1)	0	0	0	0	0	0	0	0	0	0	0
Penile edema	1 (1)	0	0	0	0	0	0	0	0	0	0	0
Perineal pain	1 (1)	0	0	0	0	0	0	0	0	0	0	0
Vaginal hemorrhage	1 (1)	0	0	0	0	0	0	0	0	0	0	0
Motion sickness	1 (1)	0	0	0	0	0	0	0	0	0	0	0
Fall	1 (1)	0	0	0	0	0	0	0	0	0	0	0

[a]Any-grade TEAEs were reported in both the patient with BCC and the patient with NUT carcinoma enrolled in part B. One patient had a grade 3/4 TEAE.

ALT, alanine aminotransferase; AST, aspartate aminotransferase; BCC, basal cell carcinoma; DLBCL, diffuse large B-cell lymphoma; ECG, electrocardiogram; GGT, gamma-glutamyl transferase; INR, international normalized ratio; NUT, nuclear protein in testis; R/R, relapsed/refractory; TEAE, treatment-emergent adverse event.

Supplementary Table 4 Treatment interruption, dose reductions, and treatment discontinuation due to adverse events.

		Part A		
**AE, *n* (%)**	**Overall population (*N* = 69)**	**45 mg/day 4 days on/ 24 days off (*n* = 7)**	**Part B R/R DLBCL (*N* = 23)**	**Part C (*N* = 41)**
TEAE leading to treatment interruption	9 (13)	2 (29)	7 (30)	12 (29)
TEAE leading to dose reduction	9 (13)	0	4 (17)	11 (27)
TEAE leading to treatment discontinuation	1 (1)	0	6 (26)	4 (10)
TRAE leading to treatment discontinuation	0	0	5 (22)	1 (2)

AE, adverse event; DLBCL, diffuse large B-cell lymphoma; TEAE, treatment-emergent adverse event; TRAE, treatment-related adverse event.

Supplementary Table 5 Treatment-related adverse events reported during cycles 1 and 2 in the part C population, following fasted or fed administration of trotabresib.[a]

	Fasted (*n* = 39)				Fed (*n* = 35)			
**TRAEs, *n* (%)**	**Any grade**	**Grade 3**	**Grade 4**	**Grade 5**	**Any grade**	**Grade 3**	**Grade 4**	**Grade 5**
Any TRAE	37 (95)	12 (31)	1 (3)	0	32 (91)	7 (20)	0	0
Thrombocytopenia	19 (49)	3 (8)	1 (3)	0	17 (49)	2 (6)	0	0
Stomatitis	17 (44)	1 (3)	0	0	15 (43)	1 (3)	0	0
Nausea	16 (41)	1 (3)	0	0	13 (37)	0	0	0
Diarrhea	15 (38)	1 (3)	0	0	12 (34)	1 (3)	0	0
Dysgeusia	14 (36)	0	0	0	13 (37)	0	0	0
Asthenia	12 (31)	0	0	0	7 (20)	1 (3)	0	0
Vomiting	9 (23)	1 (3)	0	0	4 (11)	0	0	0
Fatigue	8 (21)	3 (8)	0	0	3 (9)	0	0	0
Decreased appetite	7 (18)	0	0	0	8 (23)	0	0	0
Neutropenia	5 (13)	0	0	0	1 (3)	1 (3)	0	0
ALT increased	4 (10)	1 (3)	0	0	6 (17)	1 (3)	0	0
Hyperglycemia	4 (10)	1 (3)	0	0	5 (14)	0	0	0
Blood bilirubin increased	4 (10)	0	0	0	4 (11)	0	0	0
Acneiform dermatitis	4 (10)	0	0	0	1 (3)	0	0	0
AST increased	3 (8)	0	0	0	3 (9)	0	0	0
Dizziness	2 (5)	0	0	0	3 (9)	0	0	0
Dyspepsia	2 (5)	0	0	0	2 (6)	0	0	0
Headache	2 (5)	0	0	0	2 (6)	0	0	0
GGT increased	2 (5)	0	0	0	2 (6)	0	0	0
Palmar-plantar erythrodysesthesia syndrome	2 (5)	0	0	0	2 (6)	0	0	0

TRAEs, *n* (%)	Fasted (*n* = 39) Any grade	Grade 3	Grade 4	Grade 5	Fed (*n* = 35) Any grade	Grade 3	Grade 4	Grade 5
Anemia	1 (3)	0	0	0	2 (6)	0	0	0
Hypophosphatemia	1 (3)	1 (3)	0	0	1 (3)	1 (3)	0	0
Hypertension	1 (3)	1 (3)	0	0	1 (3)	0	0	0
Constipation	1 (3)	0	0	0	1 (3)	0	0	0
Amylase increased	1 (3)	0	0	0	1 (3)	0	0	0
Skin fissures	1 (3)	0	0	0	1 (3)	0	0	0
Epistaxis	1 (3)	0	0	0	1 (3)	0	0	0
Vision blurred	1 (3)	0	0	0	1 (3)	0	0	0
Gilbert's syndrome	1 (3)	0	0	0	1 (3)	0	0	0
Hyperbilirubinemia	1 (3)	0	0	0	1 (3)	0	0	0
Back pain	1 (3)	0	0	0	1 (3)	0	0	0
Syncope	1 (3)	1 (3)	0	0	0	0	0	0
Abdominal pain	1 (3)	0	0	0	0	0	0	0
Abdominal pain lower	1 (3)	0	0	0	0	0	0	0
Dry mouth	1 (3)	0	0	0	0	0	0	0
Lip ulceration	1 (3)	0	0	0	0	0	0	0
Blood ALP increased	1 (3)	0	0	0	0	0	0	0
Dyspnea	1 (3)	0	0	0	0	0	0	0
Dyspnea exertional	1 (3)	0	0	0	0	0	0	0
Eyelid infection	1 (3)	0	0	0	0	0	0	0
Oral herpes	1 (3)	0	0	0	0	0	0	0
Eyelid edema	1 (3)	0	0	0	0	0	0	0
Pain in extremity	1 (3)	0	0	0	0	0	0	0
Abdominal pain upper	0	0	0	0	2 (6)	0	0	0
Weight decreased	0	0	0	0	2 (6)	0	0	0

TRAEs, *n* (%)	Fasted (*n* = 39)				Fed (*n* = 35)			
	Any grade	Grade 3	Grade 4	Grade 5	Any grade	Grade 3	Grade 4	Grade 5
Dry skin	0	0	0	0	2 (6)	0	0	0
Rash	0	0	0	0	2 (6)	0	0	0
Rash maculo-papular	0	0	0	0	2 (6)	0	0	0
Lip dry	0	0	0	0	1 (3)	0	0	0
Esophagitis	0	0	0	0	1 (3)	0	0	0
Pyrexia	0	0	0	0	1 (3)	0	0	0
Xerosis	0	0	0	0	1 (3)	0	0	0
Blood creatinine increased	0	0	0	0	1 (3)	0	0	0
Transaminases increased	0	0	0	0	1 (3)	0	0	0
Pruritis	0	0	0	0	1 (3)	0	0	0
Nasal inflammation	0	0	0	0	1 (3)	0	0	0
Pharyngeal inflammation	0	0	0	0	1 (3)	0	0	0
Productive cough	0	0	0	0	1 (3)	0	0	0
Herpes zoster	0	0	0	0	1 (3)	0	0	0
Mucosal infection	0	0	0	0	1 (3)	0	0	0
Photophobia	0	0	0	0	1 (3)	0	0	0

[a]Data cutoff January 29, 2021. ALT, alanine aminotransferase; TRAE, treatment-related adverse event.

Supplementary Fig. 1 Swim plot showing duration of treatment for patients in part A (N = 69).

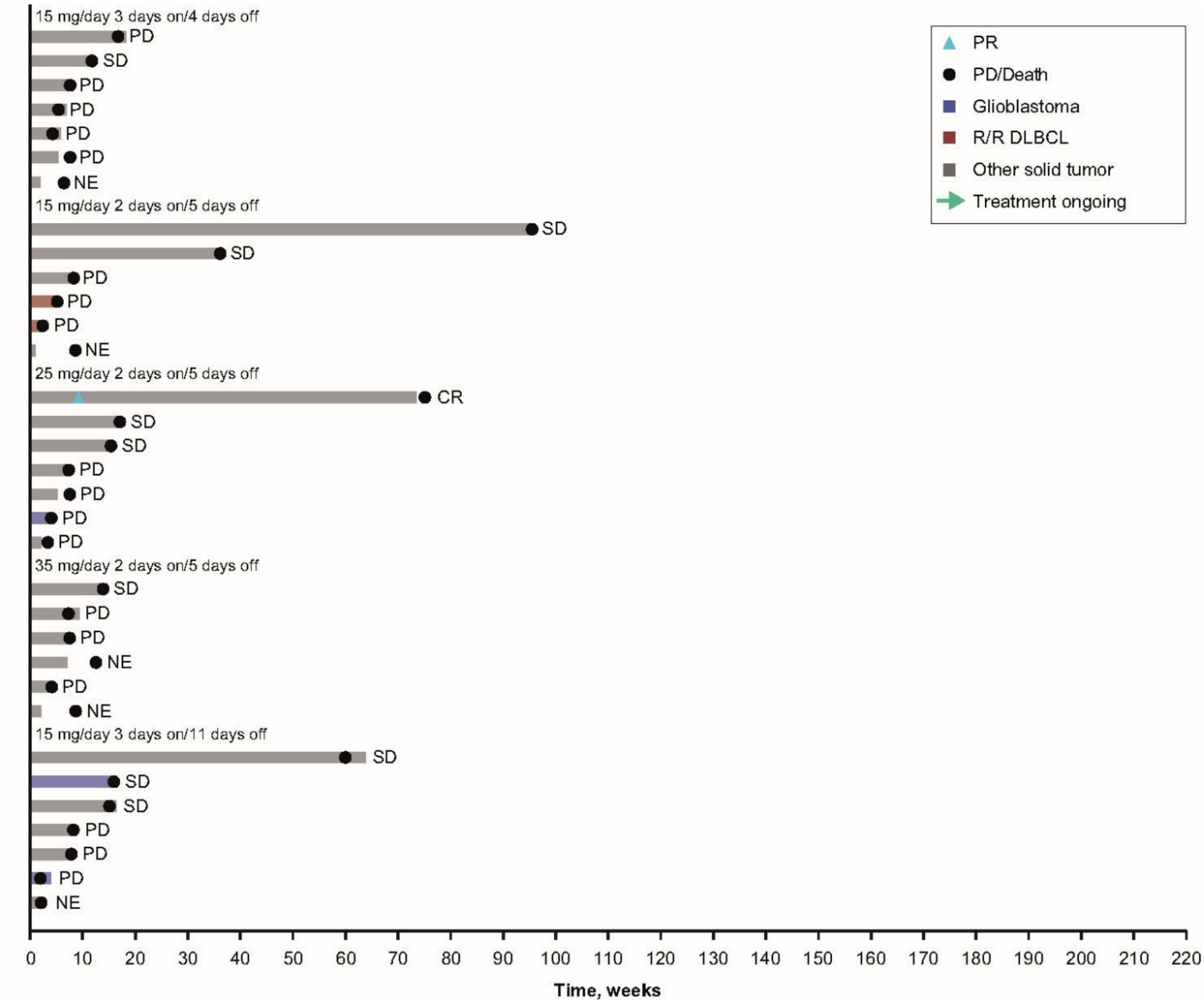

Supplementary Fig. 1 continued

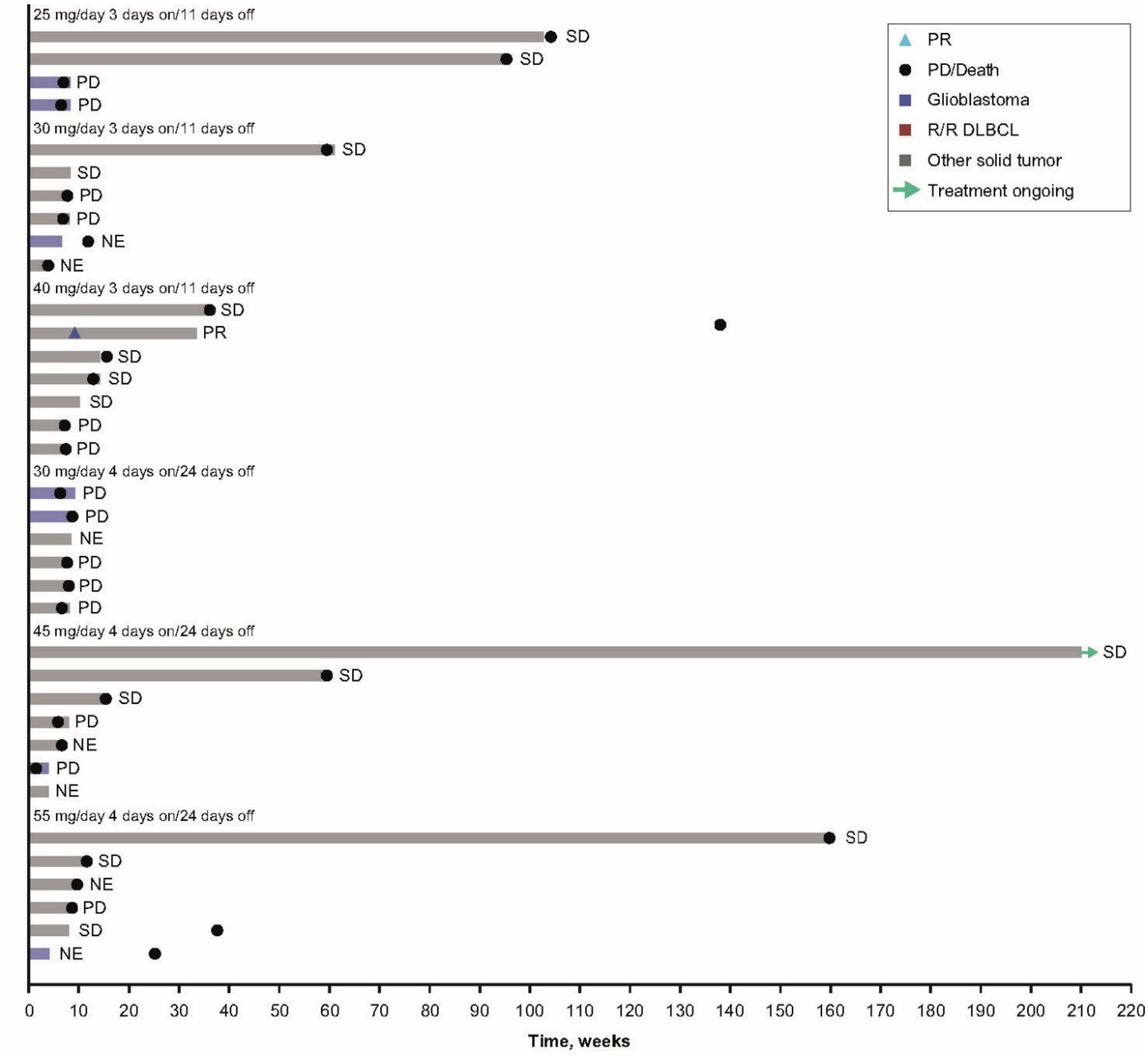

NE, not evaluable; SD, stable disease; PD, progressive disease; R/R DLBCL, relapsed/refractory diffuse large B-cell lymphoma.

A PHASE 1, OPEN-LABEL, DOSE-FINDING STUDY TO ASSESS THE SAFETY, TOLERABILITY, PHARMACOKINETICS AND PRELIMINARY EFFICACY OF CC-90010 IN SUBJECTS WITH ADVANCED SOLID TUMORS AND RELAPSED/REFRACTORY NON-HODGKIN'S LYMPHOMAS

**PROTOCOL NUMBER:**	**CC-90010-ST-001**
**DATE FINAL:**	**05 February 2016**
**AMENDMENT NO. 1:**	**21 March 2017**
**AMENDMENT NO. 2:**	**15 November 2017**
**AMENDMENT NO. 3:**	**03 December 2018**
**AMENDMENT NO. 4:**	**10 May 2019**
**AMENDMENT NO. 5:**	**15 April 2020**
**EudraCT NUMBER:**	**2015-004371-79**
**IND NUMBER:**	**Not Applicable**
**SPONSOR NAME/ ADDRESS:**	Celgene Corporation<br>86 Morris Avenue<br>Summit, NJ 07901

CONFIDENTIAL
This protocol is provided to you as an Investigator, potential Investigator, or consultant for review by you, your staff, and ethics committee/institutional review board. The information contained in this document is regarded as confidential and, except to the extent necessary to obtain informed consent, may not be disclosed to another party unless such disclosure is required by law or regulations. Persons to whom the information is disclosed must be informed that the information is confidential and may not be further disclosed by them.

MEDICAL MONITOR / EMERGENCY CONTACT INFORMATION

**Contact Information:**	
**Name:**	[redacted]
**Title:**	[redacted]
**Address:**	[redacted]
**Phone:**	[redacted]
**E-mail:**	[redacted]

**Contact Information:**	
**Name:**	[redacted]
**Title:**	[redacted]
**Address:**	[redacted]
**Phone:**	[redacted]
**E-mail:**	[redacted]

Note: The back-up 24-hour global emergency contact call center should only be used if you are not able to reach the Clinical Research Physician(s) or Medical Monitor or designee for emergency calls.

**Back-up 24-hour Global Emergency Contact Call Center:** [redacted]

CELGENE THERAPEUTIC AREA HEAD SIGNATURE PAGE

{See appended electronic signature page}
**Signature of Celgene Therapeutic Area Head** **dd mmm yyyy**
[redacted]
**Printed Name of Celgene Therapeutic Area Head and Title**
By my signature, I indicate I have reviewed this protocol and find its content to be acceptable.

SITE PRINCIPAL INVESTIGATOR SIGNATURE PAGE

**Signature of Site Principal Investigator**	**dd mmm yyyy**
**Printed Name of Site Principal Investigator**	
**Institution Name:**____________________________________	
By my signature, I agree to personally supervise the conduct of this study at my study site and to ensure its conduct is in compliance with the protocol, informed consent, Institutional Review Board (IRB)/Ethics Committee (EC) procedures, instructions from Celgene representatives, the Declaration of Helsinki, International Council for Harmonisation (ICH) Good Clinical Practices Guidelines, and local regulations governing the conduct of clinical studies.	

COORDINATING PRINCIPAL INVESTIGATOR SIGNATURE PAGE

**Signature of Coordinating Principal Investigator**	**dd mmm yyyy**
**Printed Name of Coordinating Principal Investigator**	
**Institution Name:**____________________________________	
By my signature, I agree the protocol has been written to comply with ICH Good Clinical Practices guidelines and agree to offer guidance throughout the study as needed.	

PROTOCOL SUMMARY

Study Title

A Phase 1, Open-label, Dose-Finding Study to Assess the Safety, Tolerability, Pharmacokinetics and Preliminary Efficacy of CC-90010 in Subjects with Advanced Solid Tumors and Relapsed/Refractory Non-Hodgkin's Lymphomas

Indication

Advanced or unresectable solid tumors and relapsed and/or refractory advanced non-Hodgkin's lymphomas (NHLs) [ie, diffuse large B-cell lymphoma (DLBCL) and follicular lymphoma (FL) or marginal zone lymphoma (MZL)] in Part A. Relapsed and/or refractory DLBCL and advanced basal cell carcinoma (BCC) in Part B. Advanced or unresectable solid tumors in Part C (Spain only).

Objectives

Primary Objectives:

- To determine the safety and tolerability of CC-90010.
- To define the maximum tolerated dose (MTD) and/or the recommended Phase 2 dose (RP2D) of CC-90010.

Secondary Objectives:

- To provide information on the preliminary efficacy of CC-90010.
- To characterize the pharmacokinetics (PK) of CC-90010.
- To evaluate the food effect on the PK and safety profile of CC-90010 when administered at the RP2D of 45 mg 4-days-on/24-days-off (180 mg per 28-day cycle) in a fed state (high-fat and high-calorie meal) relative to a fasted state.

Exploratory Objectives are outlined in Section 2.

Study Design

Study CC-90010-ST-001 is an open-label, Phase 1a, dose escalation and expansion, First-In-Human (FIH) clinical study of CC-90010 in subjects with advanced or unresectable solid tumors and relapsed and/or refractory advanced NHLs. The dose escalation part (Part A) of the study will explore escalating oral doses of CC-90010 to estimate the MTD of CC-90010. A Bayesian logistic regression model (BLRM) utilizing escalation with overdose control (EWOC) (Babb, 1998; Neuenschwander, 2008) will help guide CC-90010 dose escalation decisions with the final decisions made by a safety review committee (SRC). The expansion part (Part B) will further evaluate the safety and efficacy of CC-90010 administered at or below the MTD in selected expansion cohorts in order to further define the RP2D. One or more dosing regimens and/or disease subsets may be selected for cohort expansion in Part B. The food effect assessment (Part C) will evaluate the impact of food on CC-90010 by comparison of the PK parameters following fasted and fed (high-fat, high calorie meal) conditions in subjects with advanced solid tumors. Parts A, B and C will consist of 3 periods: Screening, Treatment, and Follow-up periods (refer to Figure 4).

EDMS Doc. Number: [redacted]

Screening Period

The Screening Period starts 28 days (+3 days) prior to first dose of CC-90010. The informed consent document (ICD) must be signed and dated by the subject and the administering staff prior to the start of any other study-specific procedures. All screening tests and procedures must be completed within the 28 days (+3 days) prior to the first dose of CC-90010.

Treatment Period

During the Treatment Period, CC-90010 was administered orally once daily for 3 consecutive days followed by 4 consecutive days off drug every week (3/7-days schedule) in each 28-day cycle in Part A. Alternative dosing schedules (eg, 2-days-on/5-days- off each week, 3-days-on/4-days-off every other week, 4-days on/24 days off) were evaluated one dosing schedule at a time or ≥ 2 dosing schedules given in parallel, based on the review of available safety, PK, pharmacodynamic (PD), and efficacy data by the SRC. The starting dose and schedule of each alternative dosing schedule will not exceed the dose intensity of a dose cohort that has met the criteria for dose escalation. In Part A, the window for evaluation of dose-limiting toxicity (DLT) will be 28 days (4 weeks) during Cycle 1.

Following completion of dose escalation in Part A, selected expansion cohorts will receive CC-90010 in Part B. The SRC determined the RP2D for Part B to be 45 mg CC-90010 given once daily for 4 consecutive days on followed by 24 consecutive days off (4-days-on/24-days-off) in each 28-day cycle. A cohort of up to approximately 20-25 subjects with relapsed and/or refractory DLBCL (Cohort 1) will be enrolled in Part B expansion. Enrollment in advanced basal cell carcinoma (BCC) (Cohort 2) will be stopped due to recruitment challenges. An additional cohort of approximately 15 evaluable subjects with relapsed and/or refractory DLBCL (Cohort 3) will be enrolled under an alternative dosing regimen of 30 mg CC-90010 3-days-on/11-days off in each 28-day cycle.

The food effect assessment (Part C, Spain only) will evaluate the impact of food on CC-90010 by comparison of PK parameters following fasted and fed (high-fat, high- calorie meal) conditions in approximately 24 subjects with advanced solid tumors. CC-90010 will be administered at the RP2D of 45 mg CC-90010 given once daily for 4 consecutive days on, followed by 24 consecutive days off (4-days-on/24-days-off) in each 28-day cycle.

Follow-up Period

In the Follow-up Period, all subjects will be followed for 28 days (±3 days) after the last dose of CC-90010 for safety.

Subjects who discontinue treatment for reasons other than disease progression, start of a new anticancer therapy, or withdrawal of consent from the entire study will have disease assessments performed according to the specified tumor assessment schedule until progression and/or initiation of new systemic anticancer therapies.

After the Safety Follow-up visit, all subjects will be followed every subsequent 3 months (± 2 weeks) for survival follow-up for up to 2 years or until death, lost to follow-up, or the End of Trial, whichever occurs first.

Part A Dose Escalation

A minimum of 3 subjects will be enrolled at each dose level in Part A. The initial CC-90010 dose is 15 mg. The BLRM with EWOC will incorporate available prior safety information and update the model parameters after each new cohort of subjects completes Cycle 1. The decision for the next dose will be made by the SRC based on a calculation of risk assessment using the BLRM, and available safety (eg, DLT and non-DLT safety data), PK, PD, and preliminary efficacy information. In addition, relevant non-clinical data (eg, Good Laboratory Practice [GLP] toxicity studies, in vivo pharmacology from xenograft models, etc.) may be utilized in the assessment. Details of the statistical methodology are provided in Appendix K.

At all decision time points, the BLRM permits alterations in the dose increments based on the observed DLTs; however, the dose for the next cohort will not exceed a 100% increase from the prior dose. The MTD is the highest dose that causes DLTs in not more than 33% of the subjects treated with CC-90010 in the first cycle, with at least 6 evaluable subjects treated at this dose. The SRC will make the final decision regarding the CC-90010 dose for each cohort.

During dose escalation, a CC-90010 dose can be declared the MTD after meeting the following conditions:

- at least 6 evaluable subjects have been treated at the dose,
- the posterior probability of targeted toxicity (eg, the true DLT rate lying in [0.16, 0.33]) at the dose exceeds 60% or a minimum of 6 subjects have been treated on the study, and
- the dose is recommended according to the BLRM and the SRC approves it.

The SRC will include Investigators (and/or designated representatives), the Sponsor's study physician, safety physician, study statistician, and the study manager. Ad hoc attendees may include the study pharmacokineticist, the study biomarker and study clinical scientists. Other internal and external experts may be consulted by the SRC, as necessary.

The decision to evaluate additional subjects within a dose cohort, a higher dose cohort, intermediate dose cohorts, smaller dose increments, alternative dosing schedules (eg, 2-days-on/5-days- off each week, 3-days-on/4-days-off every other week, 4-days on/24-days off) administered as one dosing schedule at a time or ≥ 2 dosing schedules given in parallel, or declare a MTD will also be determined by the SRC, based on the BLRM assessment and their review of available safety (ie, DLT and non-DLT data), PK, PD, and preliminary efficacy information. The final decision will be made by the SRC.

After the first dose is administered in any cohort during dose escalation, subjects in each cohort are observed for 28 days (Cycle 1, DLT window) before the next dose cohort can begin. No more than one subject per day will be enrolled in a given dose escalation cohort.

A subject evaluable for DLT is defined as one that:

- Has received ≥ 80% of the total planned dose amount of CC-90010 during Cycle 1 without experiencing a DLT,

or

- Experienced a DLT after receiving at least one dose of CC-90010.

Subjects non-evaluable for DLT will be replaced.

Intra-subject dose escalation will not be allowed during the DLT assessment period. However, in Cycles ≥ 2, subjects with evidence of clinical benefit (eg, symptom improvement even with concurrent initial increase in lesions) or without evidence of disease progression who are tolerating their assigned dose of CC-90010 may (at the Investigator's discretion and in consultation and agreement with the Sponsor's study physician) escalate to the highest dose level shown to be adequately tolerated by at least one cohort of subjects in this study (ie, overdose risk is less than 25% based on the BLRM assessment).

Part B Dose Expansion

Following completion of dose escalation (Part A), selected tumor cohorts may be enrolled into an expansion phase (Part B. Part B expansion cohorts will include relapsed and/or refractory DLBCL (Cohort 1) and advanced BCC (Cohort 2). Enrollment in advanced basal cell carcinoma (BCC) (Cohort 2) will be stopped due to recruitment challenges. The SRC determined the RP2D for Part B to be 45 mg CC-90010 given once daily for 4 consecutive days followed by 24 consecutive days off (4-days-on/24-days-off) in each 28-day cycle. An additional cohort of approximately 15 evaluable subjects with relapsed and/or refractory DLBCL (Cohort 3) will be enrolled under an alternative dosing regimen of 30 mg CC-90010 3-days-on/11-days off in each 28-day cycle. One or more dosing regimens may be selected for cohort expansion. The SRC will continue to review safety data regularly throughout the study and make recommendations about study continuation and dose modification, as appropriate.

Part C Food Effect Assessment

The food effect assessment (Part C, Spain only) is a randomized 2-sequence, 2-period crossover substudy to assess the impact of concomitant food intake on the PK and safety profile of CC-90010, under fasted and fed conditions. This balanced crossover design removes the inter-subject variability from the comparison between treatment (fed vs fasted), while randomly assigning subjects to one of the sequences controls bias that might otherwise influence the comparison. This design is in accordance with the European Medicines Agency (EMA) Guideline on the investigation of drug interactions (EMA CHMP, 2012) and FDA Guidance on Food-Effect Bioavailability and Fed Bioequivalence Studies (FDA, 2002). Consistent with the RP2D administered in Part B, CC-90010 will be given once daily for 4 consecutive days on followed by 24 consecutive days off (4-days-on/24-days-off) in each 28-day cycle. For specific evaluation of concomitant food intake, on Day 4 of each period (Cycle 1 and Cycle 2), subjects will either receive CC-90010 after an overnight fast lasting at least 10 hours, or within 30 minutes of a high-fat high-calorie breakfast.

Following confirmation of eligibility during screening, subjects will be randomized to one of two treatment sequences administered in 2 separate periods: Treatment A then B, or Treatment B then A, as shown in Figure 5. Period 1 and Period 2 of Part C correspond to Cycle 1 and Cycle 2, respectively. In each period in Part C, 12 subjects will be assigned to Treatment A and 12 subjects will be assigned to Treatment B, leading to a total of 24 subjects for this assessment. Treatment A and B are as described below:

- Treatment A: Single oral dose of CC-90010 (45 mg) at Hour 0 on Day 4, following a 10 hour overnight fast.
- Treatment B: Single oral dose of CC-90010 (45 mg) at Hour 0 on Day 4, 30 minutes after the start of a high-fat high-calorie breakfast.

EDMS Doc. Number: [redacted]

Blood samples will be taken prior to dosing and at several time points for up to 18 days (432 hours, corresponding to Day 22 of the cycle) following the last dose of CC-90010 in each sequence. The impact of food on CC-90010 will be characterized through PK parameters that will be calculated and compared between fasted and fed conditions.

Following Period 2 (Cycle 2), subjects will continue in the treatment period of the study at the RP2D (Figure 4).

For Part C, approximately 24 subjects who complete both treatment Periods 1 and 2 (completers) will be enrolled. Subjects may be replaced at the discretion of the sponsor to ensure 24 completers.

The study will be conducted in compliance with International Council for Harmonisation (ICH)/Good Clinical Practices (GCPs).

Study Population

Subjects to be enrolled will be men and women, 18 years or older, with histological or cytological confirmation of advanced or unresectable solid tumors or relapsed and/or refractory advanced NHL (ie, DLBCL, FL and MZL) including those who have progressed on (or not been able to tolerate due to medical comorbidities or unacceptable toxicity) standard anticancer therapy or for whom no other approved conventional therapy exists.

Following completion of Part A dose escalation, enrollment in Part B dose expansion will occur in the following cohorts:

- Cohort 1: relapsed and/or refractory DLBCL - approximately 20-25 evaluable subjects at 45 mg CC-90010 4-days-on/24-days-off in each 28-day cycle
- Cohort 2: advanced BCC - enrollment stopped due to recruitment challenges
- Cohort 3: relapsed and/or refractory DLBCL - approximately 15 evaluable subjects at 30 mg CC-90010 3-days-on/11-days-off in each 28-day cycle

Following completion of Part A (dose escalation) and in parallel to Part B (dose expansion), Part C (food effect assessment) will enroll approximately 24 subjects with advanced solid tumors (comparing fasted and fed conditions).

Length of Study

Enrollment is expected to take approximately 38 months to complete (up to 18 months for dose escalation and up to 20 months for expansion). Completion of active treatment and post-treatment follow-up is expected to take an additional 6 to 33 months. The entire study is expected to last approximately 6 years.

The End of Trial is defined as either the date of the last visit of the last subject to complete the post-treatment follow-up, or the date of receipt of the last data point from the last subject that is required for primary, secondary and/or exploratory analysis, as pre-specified in the protocol, whichever is the later date.

Study Treatments

Celgene Corporation (Celgene) will supply the investigational product (IP) labeled appropriately for investigational use as per the regulations of the relevant country health authority.

EDMS Doc. Number: [redacted]

Celgene will supply CC-90010 as formulated tablets for oral administration.

Study treatment may be discontinued if there is evidence of clinically significant disease progression, unacceptable toxicity or subject/physician decision to withdraw. Subjects may continue to receive study drug beyond initial signs of disease progression at the discretion of the Investigator after consultation with the Sponsor´s study physician.

Overview of Key Efficacy Assessments

Subjects will be evaluated for efficacy after every 2 cycles through Cycle 6, and then every 3 cycles thereafter. All subjects who discontinue treatment for reasons other than disease progression, start of a new anticancer therapy, or withdrawal of consent from the entire study will be followed until progression and/or initiation of new systemic anticancer therapies.

Tumor response will be determined by the Investigator. For solid tumors, assessment will be based on Response Evaluation Criteria in Solid Tumors (RECIST 1.1) (Eisenhauer, 2009). For NHL, assessment will be based on the International Working Group (IWG) Response Criteria for Malignant Lymphoma (Cheson, 2014) and the Deauville Criteria for fluorodeoxyglucose-positron emission tomography (FDG PET) scan interpretation (Itti, 2013; Meignan, 2014) ("Lugano criteria"). [^{18}F]fluorodeoxyglucose (FDG) positron emission tomography (PET) or FDG PET/computed tomography (CT) imaging is required to confirm a complete response (CR) in subjects with FDG-avid tumors. For subjects with glioblastoma multiforme, assessment will be based on the Response Assessment in Neuro-Oncology criteria (Wen, 2010). For locally advanced basal cell carcinoma (LaBCC) subjects, a conglomeration of radiology of target lesions assessed by RECIST 1.1, digital clinical photography assessed by World Health Organization (WHO) (bi-dimensional assessment) (Miller, 1981) and punch biopsies to confirm CR or if response confounded by lesion ulceration, cyst, or scarring/fibrosis will be used.

Overview of Key Safety Assessments

The safety variables for this study include adverse events, safety clinical laboratory variables, 12-lead electrocardiograms (ECGs), Eastern Cooperative Oncology Group (ECOG) Performance Status, left ventricular ejection fraction assessments (LVEF), physical examinations, vital signs, exposure to study treatment, assessment of concomitant medications, and pregnancy testing for females of child bearing potential.

Overview of Key Pharmacokinetic Assessments

The PK profiles of CC-90010 will be determined from serial blood collections, as well as in tumor tissue, in both Part A and Part B. The plasma PK parameters determined for CC-90010 will be peak (maximum) plasma concentration of drug (C_{max}), area under the plasma concentration time-curve (AUC), time to peak (maximum) plasma concentration (t_{max}), terminal half-life ($t_{1/2}$), apparent clearance (CL/F), apparent volume of distribution (V_z/F), and accumulation index. Additional PK parameters including CSF to plasma concentration ratio of CC-90010, may be calculated, if data permits.

In Part C, plasma PK parameters such as C_{max}, T_{max}, $AUC_{0-\infty}$, AUC_{0-Last}, AUC_{0-24}, and $t_{½}$ will be estimated following administration of CC-90010 under fasted and fed conditions.

Overview of Exploratory Pharmacodynamic Assessments

One of the exploratory objectives of this study is to evaluate the pharmacodynamic (PD) effects of CC-90010 on gene expression in peripheral blood and in tumor samples. A set of genes, including CCR1, the expression of which is changed upon ex vivo treatment with CC-90010 has been identified in peripheral blood mononuclear cells (PBMCs) and in whole blood. In the current study, changes in the expression of these genes in whole blood and/or other genes and proteins in PBMCs and in tumor biopsy tissues may provide confirmation that a dose of CC-90010 is pharmacologically active and engages its molecular target. Additional PD markers of cell viability and apoptosis, such as Poly(ADP-ribose) polymerase (PARP) cleavage, in tumor tissues may be assessed if data permits. Pharmacodynamic (PD) assessments are described in Section 6.6.

Statistical Methods

The primary objectives of this study are to evaluate the safety and tolerability of treatment with CC-90010, including the determination of the MTD and/or the RP2D. The BLRM with EWOC (Babb, 1998; Neuenschwander, 2008) will be utilized to make recommendation for dose escalation and to estimate the MTD for CC-90010, with Part A safety information incorporated.

Statistical analyses will be performed by dose level (Part A) and tumor cohort (Part B) as needed or applicable. The CC-90010 plasma concentrations and PK parameters under fasted and fed conditions in Part C will be summarized using descriptive statistics. Plasma PK parameters such as C_{max}, T_{max}, $AUC_{0-\infty}$, AUC_{0-Last}, AUC_{0-24}, and $t_{½}$ will be estimated following administration of CC-90010 under fasted and fed conditions. All analyses will be descriptive in nature. To characterize the PK of CC-90010 under fasted and fed conditions in the food effect evaluation, an analysis of variance will be performed on the natural log-transformed AUC_{0-24}, AUC_{0-Last}, $AUC_{0-\infty}$ (data permitting), and C_{max} of CC-90010 using MIXED procedure in SAS®. The MIXED model will contain terms for sequence, period, and treatment as fixed effects, and subject nested within sequence as a random effect. The geometric mean ratios (fed/fasted) and their 90% CIs will be provided. For T_{max}, a nonparametric analysis will be used to produce a median difference between treatments. Results will be presented in tabular and graphic forms, as appropriate. All summaries of safety data will be conducted using subjects receiving any CC-90010 dose (the Treated Population).

Study data will be summarized for disposition, demographic and baseline characteristics, exposure, efficacy, safety, PK, and PD. Categorical data will be summarized by frequency distributions (number and percentages of subjects) and continuous data will be summarized by descriptive statistics (mean, standard deviation, median, minimum, and maximum).

Treatment-emergent adverse events (TEAEs) will be summarized by National Cancer Institute Common Terminology Criteria for Adverse Event grades. The frequency of TEAEs will be tabulated by Medical Dictionary for Regulatory Activities system organ class and preferred term. Grade 3 or 4 TEAEs, TEAEs leading to discontinuation of CC-90010, study drug-related TEAEs, and serious adverse events will be tabulated separately. Changes from baseline in selected laboratory analytes, vital signs, 12-lead ECGs, and assessments of LVEF will be summarized. All data will be presented in by-subject listings.

The primary efficacy variable for Part A is clinical benefit rate (CBR). CBR is defined as tumor responses (as assessed by the Investigators) of complete response (CR), partial response (PR)

and stable disease (SD) (SD of ≥ 4 months duration). Point estimates and 95% confidence intervals of CBR will be reported. Objective response rate (defined as the percentage of subjects whose best response is complete response or partial response), duration of response/stable disease, progression-free survival, and overall survival will be summarized using frequency tabulations for categorical variables, or descriptive statistics for time to event variables. Efficacy analysis will be repeated for the Treated Population and Efficacy Evaluable Population (subjects who received a baseline disease assessment evaluation, at least 80% of assigned doses in Cycle 1, and one on-study disease assessment evaluation), with the result using the Treated Population considered primary.

During the Part A dose escalation, 69 evaluable subjects were enrolled. During the Part B dose expansion, at least 15 efficacy evaluable subjects for each tumor cohort will be initially enrolled. Cohort 1 will be expanded to approximately 20-25 subjects if a responder or SD of 4 months or longer is observed. Part C (food effect assessment) will enroll approximately 24 subjects with advanced solid tumors (comparing fasted and fed conditions).

TABLE OF CONTENTS

TITLE PAGE 1
PROTOCOL SUMMARY 6
1. INTRODUCTION 23
1.1. Disease Background 23
1.2. Compound Background 25
1.2.1. Nonclinical Pharmacology 25
1.2.1.1. Mechanism of Action 25
1.2.1.2. In Vitro Pharmacology 26
1.2.1.3. In Vivo Pharmacology 28
1.2.2. Nonclinical Pharmacokinetics and Drug Metabolism 31
1.2.3. Nonclinical Toxicology 32
1.3. Summary of Clinical Data with CC-90010 34
1.3.1. CC-90010-ST-001 Part A – Dose Escalation 34
1.3.2. CC-90010-ST-001 Part B - Dose Expansion 36
1.3.3. Summary of Clinical PK Data from Study CC-90010-ST-001 37
1.4. Safety Monitoring Plan 38
1.5. Rationale 39
1.5.1. Study Rationale and Purpose 39
1.5.2. Rationale for the Study Design 40
1.5.3. Rationale for the Food Effect Evaluation (Part C) 40
1.5.4. Rationale for Dose, Schedule, and Regimen Selection 41
1.5.5. Rationale for Pharmacodynamics and Potential Predictive Biomarkers 43
1.5.6. Rationale for Tumor Type Selection in Part B 43
1.5.7. Rationale for Tumor Type Selection in Part C 46
2. STUDY OBJECTIVES AND ENDPOINTS 48
3. OVERALL STUDY DESIGN 50
3.1. Study Design 50
3.1.1. Part A Dose Escalation 52
3.1.2. Part B-Cohort Expansion 53
3.1.3. Part C-Food Effect Assessment 54
3.1.4. Overview of Assessments 55

EDMS Doc. Number: [redacted]

3.2. Study Duration for Subjects 56
3.3. End of Trial 56
4. STUDY POPULATION 57
4.1. Number of Subjects 57
4.2. Inclusion Criteria 57
4.3. Exclusion Criteria 59
5. TABLE OF EVENTS 62
6. PROCEDURES 73
6.1. Screening Period 73
6.2. Treatment Period 74
6.2.1. Concomitant Medication and Procedures 74
6.2.2. Adverse Event Monitoring 74
6.2.3. Weight 75
6.2.4. Vital Signs 75
6.2.5. Physical Examination and ECOG Performance Status 75
6.2.6. B Symptom Assessment (NHL Subjects Only) 75
6.2.7. 12-Lead Electrocardiograms 75
6.2.8. Left Ventricular Ejection Fraction 76
6.2.9. Pregnancy Testing and Contraceptive Counseling 76
6.2.10. Clinical Laboratory Tests 77
6.2.11. End of Treatment (EOT) 77
6.3. Follow-up Period 78
6.3.1. Safety Follow-up 78
6.3.2. Survival Follow-up 78
6.4. Efficacy Assessment 78
6.5. Pharmacokinetics 79
6.5.1. CC-90010 Pharmacokinetic Assessments- Part A 79
6.5.2. CC-90010 Pharmacokinetic Assessments- Part B 79
6.5.2.1. Tumor Tissue for PK Assessment in Part B 81
6.5.3. CC-90010 Pharmacokinetic Assessments- Part C Food Effect Assessment 81
6.6. Biomarkers, Pharmacodynamics, Pharmacogenomics 82
6.6.1. Archival Tumor Biopsy 82
6.6.2. Pharmacodynamic and Predictive Biomarkers- Part A and Part B only 82

EDMS Doc. Number: [redacted]

6.6.2.1. Whole Blood for PD Biomarker Studies- Part A 82
6.6.2.2. Whole Blood for PD Biomarker Studies- Part B 82
6.6.2.3. Tumor Tissue for PD and Predictive Biomarker Studies 84
6.6.3. Tumor Biopsies 84
7. DESCRIPTION OF STUDY TREATMENTS 86
7.1. Description of Investigational Products (IP) 86
7.1.1. CC-90010 86
7.2. Treatment Administration and Schedule 86
7.2.1. CC-90010 Administration 86
7.2.1.1. Missed Doses of CC-90010 86
7.2.1.2. CC-90010 Administration for Food Effect Study – Part C 87
7.2.2. Dose Escalation Process 88
7.2.3. Dose Escalation Decisions 89
7.2.4. Definition of a Subject Evaluable for DLT 90
7.2.5. Definition of Maximum Tolerated Dose (MTD) 90
7.2.6. Definition of Dose-Limiting Toxicity (DLT) 90
7.2.7. Criteria for Dose Escalation in the Next Cohort of Subjects 91
7.2.8. Definition of Stopping Criteria 91
7.2.9. Permitted Study Drug Adjustments 91
7.2.9.1. Criteria for Dose Reduction 92
7.2.9.2. Criteria for Dose Increase 92
7.2.9.3. Treatment Interruption for Adverse Events 92
7.2.10. Management of Select Adverse Events 93
7.2.10.1. Neutropenia, Thrombocytopenia, and Anemia 93
7.2.10.2. Pain 93
7.2.10.3. Gastrointestinal Effects 93
7.2.10.4. Hyperglycemia 94
7.2.11. Definition of Overdose 94
7.3. Method of Treatment Assignment 94
7.4. Packaging and Labeling 94
7.5. Investigational Product Accountability and Disposal 95
7.6. Investigational Product Compliance 95
8. CONCOMITANT MEDICATIONS AND PROCEDURES 96

8.1. Permitted Concomitant Medications and Procedures 96
8.2. Prohibited Concomitant Medications and Procedures 97
9. STATISTICAL CONSIDERATIONS 99
9.1. Overview 99
9.2. Study Population Definitions 99
9.3. Sample Size and Power Considerations 99
9.4. Background and Demographic Characteristics 100
9.5. Subject Disposition 100
9.6. Efficacy Analysis 101
9.7. Safety Analysis 102
9.8. Interim Analysis 103
9.9. Other Topics 103
9.9.1. Statistical Method for Dose Escalation 103
9.9.2. Assessment of Pharmacokinetics (Part A and Part B) 104
9.9.3. Assessment of Pharmacokinetics in Food Effect Evaluation (Part C) 105
9.9.4. Assessment of Pharmacodynamics 105
10. ADVERSE EVENTS 106
10.1. Monitoring, Recording and Reporting of Adverse Events 106
10.2. Evaluation of Adverse Events 106
10.2.1. Seriousness 106
10.2.2. Severity/Intensity 108
10.2.3. Causality 108
10.2.4. Duration 109
10.2.5. Action Taken 109
10.2.6. Outcome 109
10.3. Abnormal Laboratory Values 109
10.4. Pregnancy 109
10.4.1. Females of Childbearing Potential: 110
10.4.2. Male Subjects 110
10.5. Reporting of Serious Adverse Events 110
10.5.1. Safety Queries 111
10.6. Expedited Reporting of Adverse Events 111
11. DISCONTINUATIONS 113

11.1. Treatment Discontinuation 113
11.2. Study Discontinuation 113
12. EMERGENCY PROCEDURES 115
12.1. Emergency Contact 115
12.2. Emergency Identification of Investigational Products 115
13. REGULATORY CONSIDERATIONS 116
13.1. Good Clinical Practice 116
13.2. Investigator Responsibilities 116
13.3. Subject Information and Informed Consent 117
13.4. Confidentiality 117
13.5. Protocol Amendments 117
13.6. Institutional Review Board/Independent Ethics Committee Review and Approval 117
13.7. Ongoing Information for Institutional Review Board/ Ethics Committee 118
13.8. Termination of the Study 118
14. DATA HANDLING AND RECORDKEEPING 119
14.1. Data/Documents 119
14.2. Data Management 119
14.3. Record Retention 119
15. QUALITY CONTROL AND QUALITY ASSURANCE 121
15.1. Study Monitoring and Source Data Verification 121
15.2. Audits and Inspections 121
16. PUBLICATIONS 122
17. REFERENCES 123
18. APPENDICES 131
Appendix A: Table of Abbreviations 131
Appendix B: Table of Events- Part A only 138
Appendix C: Pharmacokinetic Blood Sampling Schedules for Part A, Cycle 1 143
Appendix D: Pharmacodynamic Blood Sampling Schedules for Part A, Cycle 1 146
Appendix E: RECIST Version 1.1 148
Appendix F: The Lugano Classification Criteria for the Evaluation of Non-Hodgkin Lymphoma and the Deauville 5-Point Scale 152
Appendix G: Response Assessment for Neuro-Oncology (RANO) Working Group 157

Appendix H: Performance Status Criteria 158
Appendix I: General Guidelines for Managing Hyperglycemia 159
Appendix J: Recommendations for Management of Treatment-Induced Diarrhea 160
Appendix K: Characteristics of the Bayesian Logistic Regression Model........ 161

EDMS Doc. Number: [redacted]

LIST OF TABLES

Table 1: Absolute Fifty Percent Inhibitory Concentration Values for CC-90010 and Three Bromodomain and Extra Terminal Domain Protein Inhibitors, Experiment 1 26

Table 2: Absolute Fifty Percent Inhibitory Concentration Values for Doxorubicin-resistant Diffuse Large B-cell Lymphoma Cell Lines 27

Table 3: Proposed Clinical Starting Dose of CC-90010 Based on the Severely Toxic Dose in 10% of the Rats and the Highest Non-severely Toxic Dose in the One-Month Toxicity Study in Dogs 41

Table 4: Study Objectives 48

Table 5: Study Endpoints 49

Table 6: Table of Events - Part B (Cohorts 1 and 2) and Part C 62

Table 7: Table of Events - Part B (Cohort 3) 68

Table 8: Pharmacokinetic Blood Sampling Schedule for the 4-days-on/24-days-off Schedule, Part B 79

Table 9: Pharmacokinetic Blood Sampling Schedule for the 3-days-on/11-days-off Schedule, Part B 80

Table 10: Pharmacokinetic Blood Sampling Schedule for the 4-days-on/24-days-off Schedule for Food Effect Assessment, Part C 81

Table 11: Pharmacodynamic Blood Sampling Schedule for the 4-days-on/24-days-off Schedule, Part B 83

Table 12: Pharmacodynamic Blood Sampling Schedule for the 3-days-on/11-days-off Schedule, Part B 83

Table 13: Treatment Sequences for Food Effect Assessment 87

Table 14: Precision based on Estimates of Intra-subject Coefficient of Variance 100

Table 15: Prior Parameters for Bivariate Normal Distribution of Model Parameters 103

Table 16: Table of Abbreviations 131

Table 17: Table of Events - Part A 138

Table 18: Pharmacokinetic Blood Sampling Schedule for the 3-days-on/4-days-off every week Schedule, Part A, Cycle 1 143

Table 19: Pharmacokinetic Blood Sampling Schedule for the 3-days-on/4-days-off every other week and 2-days-on/5-days-off each week Schedule, Part A, Cycle 1 143

Table 20: Pharmacokinetic Blood Sampling Schedule for the 4-days-on/24-days-off Schedule, Part A, Cycle 1 144

Table 21: Pharmacodynamic Blood Sampling Schedule for the 3-days-on/4-days-off every other week and 2-days-on/5-days-off each week Schedule, Part A, Cycle 1 146

Table 22: Pharmacodynamic Blood Sampling for the 4-days-on/24-days-off Schedule, Part A, Cycle 1 146

Table 23: Time Point Response: Subjects With Target (± Non-target) Disease 150

Table 24: Time Point Response: Subjects With Non-target Disease Only 151

Table 25: Criteria for Involvement of Site 152

Table 26: Revised Criteria for Response Assessment 153

Table 27: Eastern Cooperative Oncology Group (ECOG) Performance Status 158

Table 28: P(DLT) for Five Simulated Scenarios with Numbers in Grey Indicating Doses with True P(DLT) within the Target Toxicity Interval [16%, 33%] under 3 different dosing schedules. 162

Table 29: Summary Metrics of Simulation for BLRM under 5 dose-toxicity curves and different dosing schedule. 164

EDMS Doc. Number: [redacted]

LIST OF FIGURES

Figure 1: Dose-Dependent Tumor Growth Inhibition as Measured by Tumor Volume in a Triple-negative Breast Cancer Patient-derived Xenograft Model, COH70, following Dosing with CC-90010 29
Figure 2: Dose-Dependent Tumor Growth Inhibition as Measured by Tumor Volume in a Glioblastoma Multiforme Patient-derived Xenograft Model, GBM15, following Dosing with CC-90010 30
Figure 3: CC-90010-mediated Tumor Growth Inhibition of GBM3 (Glioblastoma Multiforme Patient-derived Xenograft) with and without Temozolomide (TMZ) 31
Figure 4: Overall Study Design....51
Figure 5: Overall Study Design for Part C (Food-Effect Assessment) 55
Figure 6: Dose Toxicity Curves for Simulation....163

1. INTRODUCTION

CC-90010 is an oral, potent and reversible inhibitor of the epigenetic target bromodomain and extra-terminal (BET) proteins. The best studied member of the BET protein family is bromodomain-containing protein-4 (BRD4). BRD4 is a transcriptional co-regulator that binds to acetylated lysines of histones H3 and H4. It regulates gene expression through recruitment of additional proteins to its chromatin binding sites and has been shown to control expression of genes involved in cell growth and oncogenesis (Shi, 2014; Filippakopoulos, 2014). Examples of such genes are MYC, FOSL1, and GLI1.

1.1. Disease Background

The epigenetic code determines if, when and where specific genes are expressed. Hence epigenetic modifications permit the cell to turn on or off gene expression, reversibly and selectively. BET proteins play a pivotal role in this process (Chaidos 2015).

The epigenetic code is a dynamic and reversible process written, erased and read by families of enzymes. Proteins termed 'writers' covalently attach acetyl or methyl groups to produce the code. Proteins that remove these marks are termed 'erasers' while proteins that recognize and bind to the modifications are termed 'readers' of the code (Arrowsmith, 2012). Initiation and progression of cancer has increasingly been linked to misreading, miswriting or miserasing of these modifications (Chi, 2010).

BET proteins are a group of epigenetic readers known to control expression of genes involved in cell growth and oncogenesis (Wyce, 2013a). The BET family has a common structural design, featuring a tandem bromodomain at the N-terminus and includes four members: BRD2, BRD3, BRD4, and BRDT (Dawson, 2012; Junwei, 2014). BRDT is found exclusively in germ cells but the other members are ubiquitous (Chaidos, 2015). BET protein deregulation has been seen in several tumors. The rare aggressive epithelial tumor, nuclear protein in testis (NUT) midline carcinoma is driven by fusions of the NUT protein with BRD3 or BRD4 ; BET inhibitors have shown preclinical activity in this tumor (Filippakopoulos, 2010; French, 2010). BRD4 deregulation occurs in leukemia, hepatocellular carcinoma and breast cancers (Zuber, 2011; Li 2015). Also, overexpression of BRD2 and BRD4 has been demonstrated in glioblastoma cells and BET inhibition by I-BET-151 showed activity in glioblastoma multiforme (GBM) xenografts, comparable to temozolomide (Pastori, 2014). Separately, BET inhibition suppressed the oncogenic transcription factor FOSL1 and its targets in a lung adenocarcinoma cell line (Lockwood, 2012).

BET proteins bind to ε-N-lysine acetylation pockets on the tails of histones, thereby affecting chromatin structure and function (Jacobson, 2000). They bind preferentially at hyperacetylated super-enhancer promoter regions and regulate transcription of target genes (Jung, 2015; Junwei, 2014) by recruiting co-activator or co-expressor complexes (Jenuwein, 2001). BRD-containing complexes binding at super-enhancer sites often localize to promoter regions of key transcription factors, such as the oncogene c-MYC, which is activated in nearly 70% of all cancers (Nilsson, 2003; Whyte, 2013; Loven, 2013). BET inhibitors disrupt these complexes, down regulate MYC and have shown activity in human tumor xenografts of MYC-driven hematologic and solid tumors (Mertz, 2011; Puissant, 2013; Shimamura, 2013; Wyce, 2013b;

Bandopadhayay, 2014; Hu, 2015; Li, 2015; Mazur, 2015). Moreover activity has been seen in clinical trials of a BET inhibitor in refractory/resistant lymphoma and leukemia (Dombret, 2014).

In DLBCL, 60-80% of MYC gene alterations are associated with BCL2 or BCL6 translocations conferring a very aggressive behavior, but MYC protein upregulation may occur in tumors without gene alterations, and its association with BCL2 overexpression also confers a poor prognosis (Ott, 2013; Ott, 2014). The small molecule BET inhibitor JQ1 was shown to displace BRD4 from acetylated chromatin resulting in suppression of MYC transcription in multiple myeloma cell lines (Delmore, 2011), and in a variety of leukemia and lymphoma cell lines harboring MYC translocations (Mertz, 2011). There is also evidence of direct regulation of MYC transcription by BET bromodomains in multiple myeloma cell lines, with BRD4 acting as a co-activator of MYC transcription through physical interaction with distal enhancer elements influencing MYC expression and function (Delmore, 2011). Treatment with the BET inhibitor OTX015 as a single agent has resulted in clinical responses including complete remissions in patients with relapsed or refractory lymphoma (Amorim, 2016; Herait, 2015; Thieblemont, 2014).

BET proteins also appear to have a role in epithelial-mesenchymal transition (EMT) and development of cancer stem cells (CSCs). Epithelial-mesenchymal transition is associated with progression and metastasis of many carcinomas and there appears to be a correlation between EMT, chemoresistance and emergence of CSCs (Thiery, 2002; Thiery, 2003; Huber, 2005; Mani, 2008; Castellanos, 2013; Satoh, 2015). Cancer stem cells have unrestrained proliferation but also can self-renew, differentiate into other cell types, and form tumors in immunodeficient mice (Castellanos, 2013). They are felt to be responsible for tumor initiation, progression, recurrence and metastasis, as well as tumor heterogeneity and resistance to treatment (Sheridan, 2006; Campbell, 2007; Li, 2008; Zhu, 2014; Dawood, 2014). Cancer stem cells have been identified in leukemias, breast [particularly basal-like breast cancer (BLBC)], colon, GBM, head and neck, hepatic, lung, melanoma, pancreas and prostate carcinomas (Fang, 2005; Ma, 2007; Tang, 2007; Eppert, 2011; Lathia, 2015). The Twist transcription factor is a key activator of EMT (Wu, 2016). It has been shown that BRD4 binds to Twist and this Twist/BRD4 interaction invokes tumorigenicity and invasion in BLBC (Shi, 2014). However BET inhibitors have been shown to block this Twist-BRD4 interaction and inhibit growth in a BLBC xenograft model. High levels of Twist have also been demonstrated in aggressive pancreatic cancer cells with high metastatic potential and breast cancer CSCs (Mani, 2008; Von Burstin, 2009). Other work in colorectal cancer (CRC) supports BRD4 playing a key role in EMT with the BRD4 inhibitor, MS417, inhibiting colon cell proliferation, migration and invasion and impairing growth in a CRC xenograft model along with suppressing development of liver metastases (Hu, 2015). Furthermore, BET proteins are critical regulators of the Hedgehog (Hh) pathway which is activated in CSCs (Varnat, 2009; Amakye, 2013; Tang, 2014; Infante, 2015).

The Hh pathway is a key regulator of cell growth and differentiation during embryogenesis (Ingham, 2001) but is normally inactive in adult tissues (Von Hoff, 2009). However, aberrant activation of this pathway is responsible for tumorigenesis of various cancers such as medulloblastoma, rhabdomyosarcoma and almost all basal cell carcinoma (BCC) (Xie, 1998; Epstein, 2008; Teglund, 2010). Hedgehog ligand over expression has also been observed in breast, colorectal, esophageal, lung, gastric, pancreatic and prostate tumors (Teglund, 2010).

Aberrant Hh pathway signaling activates the Smoothened receptor (SMO) which up regulates glioma-associated oncogene homolog 1 (GLI1) transcriptional activity. Additionally, it has been shown that GLI1- driven transcription contributes to pancreas cancer progression and that GLI transcription is independent of Hh signaling, being driven by tumor growth factor (TGF)-beta and KRAS (Nolan-Stevaux, 2009). BRD4 and other BET proteins regulate GLI1 transcription downstream of SMO, with BRD4 directly occupying GLI1 and GLI2 promoters (Tang, 2014). This occupancy can be inhibited by BET inhibitors, thus offering potential in both Hh-driven tumors associated with SMO activation and also those independent of SMO activation. Of note, the BET inhibitor, JQ1, decreased tumor cell proliferation in vitro and in vivo in Hh-driven tumors, even those resistant to SMO inhibition (Tang, 2014). Another BET inhibitor I-BET151 suppressed Hh-dependent growth of medulloblastoma in vitro and in vivo and suppressed activation of SMO-independent activation of the Hh pathway in vitro (Long, 2014).

1.2. Compound Background

CC-90010 is an oral, potent, reversible inhibitor of BET family members, including BRD2, BRD3, BRD4 and BRDT. It shows dose- and time-dependent inhibition of GLI1 and so could be of value in the treatment of Hh-driven tumors and tumors with GLI-driven transcription. CC-90010 also reduced tumor cell growth in a BLBC model in vivo and showed more potent activity than the current clinical standard of temozolomide in the GBM3 xenograft model, as well as additive or synergistic effects in combination with temozolomide, suggesting it could be useful in tumors with CSCs and MYC-driven tumors.

The clinical investigation of the BET inhibitor CC-90010 for antineoplastic activity in a variety of malignancies is warranted. This protocol describes the first study of CC-90010 in humans, designed to evaluate drug safety and pharmacokinetic profiles with various dose levels/regimens and to detect initial signals of drug efficacy in order to advance development to Phase 2 clinical trials.

Please refer to the Investigator's Brochure for detailed information concerning the available pharmacology, toxicology, pharmacokinetics, and drug metabolism profile of the investigational product (IP).

1.2.1. Nonclinical Pharmacology

1.2.1.1. Mechanism of Action

Regulation of MYC gene expression by BRD4 has been shown in models of Burkitt's lymphoma with inhibition of BRD4, leading to growth arrest (Mertz, 2011). Similarly, in a model of lung adenocarcinoma, BRD4 inhibition was also found to be antiproliferative; however, this effect was ascribed to FOSL1 down-regulation (Lockwood, 2012). BRD4 also has been shown to regulate GLI1 gene expression, thereby modulating the Hh signaling pathway, which is known to be dysregulated in several cancer types (Tang, 2014). The effect of CC-90010 treatment on MYC, FOSL1, and GLI1 gene expression was evaluated by quantitative reverse transcription polymerase chain reaction (qRT-PCR). Treatment with CC-90010 inhibited MYC gene expression in Raji Burkitt's lymphoma cells with a mean half-maximal inhibitory concentration (IC_{50}) value of 0.06 μM, FOSL1 gene expression in U-87 glioblastoma cells with an IC_{50} value

EDMS Doc. Number: [redacted]

of 0.03 μM, and GLI1 gene expression in MIA-PaCa-2 pancreatic adenocarcinoma cells with an IC_{50} value of 0.24 μM.

1.2.1.2. In Vitro Pharmacology

Diffuse Large B-cell Lymphoma Human Cell Lines

The antiproliferative activity of CC-90010 and 3 other BET protein inhibitors was evaluated in six activated B-cell-like (ABC) DLBCL, seven germinal center B-cell-like (GCB) DLBCL, and two primary mediastinal B-cell lymphoma (PMBL) cell lines. Additionally, the antiproliferative activity of CC-90010 and the 3 other BET protein inhibitors was evaluated in two ABC and two GCB DLBCL doxorubicin-resistant cell lines (Report CC-90010-Pharm-1018).

CC-90010 exhibited broad activity against all 15 cell lines tested, inhibiting proliferation in a dose-dependent manner and independently of the DLBCL molecular subtype. CC-90010 was more potent than the 3 other BET inhibitors at inhibiting DLBCL cell viability.

The absolute IC_{50} values for CC-90010 ranged from 0.03 μM (KARPAS-1106P; PMBL) to 0.71 μM (Farage; GCB DLBCL) for Experiment 1 (Table 1) and 0.10 μM (SU DHL 2; ABC DLBCL) to 3.35 μM (Farage; GCB DLBCL) for Experiment 2 (Report CC-90010-Pharm-1018).

Table 1: Absolute Fifty Percent Inhibitory Concentration Values for CC-90010 and Three Bromodomain and Extra Terminal Domain Protein Inhibitors, Experiment 1

DLBCL Subtype	Compound Number	CC-90010 (μM)	CC0742005 (μM)	CC0994345 (μM)	CC0924104 (μM)
	**Mechanism of Action**	**Blocks recruitment of BET to chromatin**	**Blocks recruitment of BET to chromatin**	**Attenuates BET-dependent gene expression**	**Blocks recruitment of BET to chromatin**
ABC	SU-DHL-2	0.12	0.94	1.21	> 10
	RIVA	0.12	0.75	1.25	2.59
	RC-K8	0.13	0.77	1.14	1.39
	U-2932	0.15	0.86	1.62	2.69
	TMD8	0.19	1.12	2.15	> 10
	OCI-LY-3	0.51	3.32	> 10	> 10
GCB	HT	0.08	0.64	0.86	1.97
	WSU-DLCL2	0.12	1.05	1.31	> 10
	SU-DHL-4	0.16	1.07	2.97	> 10
	DB	0.18	0.94	1.39	3.65
	OCI-LY-19	0.22	1.08	3.07	>10
	KARPAS-422	0.32	1.09	1.93	9.36
	FARAGE	0.71	> 10	2.41	0.36
PMBL	KARPAS-1106P	0.03	0.41	0.47	0.39
	U-2940	0.12	0.80	1.19	3.84

ABC = activated B-cell-like DLBCL; absolute IC_{50} = inhibitory concentration corresponding to 50% of the control's response (µM); BET = bromodomain and extra terminal domain family or Family II of the bromodomain proteins (eg, BRD2-4); DLBCL = diffuse large B-cell lymphoma; GCB = germinal center B-cell-like DLBCL; PMBL = primary mediastinal B-cell lymphoma.
Source: Report CC-90010-Pharm-1018.

The absolute IC_{50} values for inhibition of cell proliferation were also determined for CC-90010 and the same 3 other BET inhibitors in two ABC and two GCB DLBCL doxorubicin resistant cell lines. Doxorubicin-resistant DLBCL cell lines exhibited an ~3-fold decrease in sensitivity to BET inhibitors. CC-90010 was more potent than the 3 other BET inhibitors at inhibiting DLBCL cell viability irrespective of the status of a cell line's sensitivity to doxorubicin (Table 2).

Table 2: Absolute Fifty Percent Inhibitory Concentration Values for Doxorubicin-resistant Diffuse Large B-cell Lymphoma Cell Lines

DLBCL Subtype	Compound Code/Number	Doxorubicin (µM)	CC-90010 (µM)	CC0742005 (µM)	CC0994345 (µM)	CC0924104 (µM)
	**Mechanism of Action**	**DNA intercalating agent**	**Blocks recruitment of BET to chromatin**	**Blocks recruitment of BET to chromatin**	**Attenuates BET-dependent gene expression**	**Blocks recruitment of BET to chromatin**
ABC	OCI-LY-10	0.041	0.418	2.377	1.993	3.321
	**OCI-LY-10 (DOX-R)**	**3.660**	**1.100**	**~4.2**	**>10**	**5.567**
	U-2932	0.020	0.167	0.340	0.688	1.429
	**U-2932 (DOX-R)**	**1.869**	**0.348**	**1.712**	**1.216**	**3.045**
GCB	SU-DHL-4	~0.01	0.304	0.631	~1.1	>10
	**SU-DHL-4 (DOX-R)**	**3.566**	**0.899**	**~3.6**	**~3.3**	**>10**
	WSU-DLCL2	0.010	0.120	~0.35	0.582	~6.9
	**WSU-DLCL2 ([1]DOX-R)**	**3.267**	**~0.36**	**~3.2**	**1.380**	**>10**

ABC = activated B-cell-like DLBCL; absolute IC_{50} = inhibitory concentration corresponding to 50% of the control's response (µM); BET = bromodomain and extra terminal domain family or Family II of the bromodomain proteins (eg, BRD2-4); DLBCL = diffuse large B-cell lymphoma; DNA = deoxyribonucleic acid; DOX-R = doxorubicin resistant; GCB = germinal center B-cell-like DLBCL; PMBL = primary mediastinal B-cell lymphoma.
Bold items represent doxorubicin-resistant cell lines.
Source: Report CC-90010-Pharm-1018.

Solid Tumor Human Cell Lines

CC-90010 demonstrated in vitro inhibition of tumor cell growth using anti-proliferative 2-dimensional (2-D) cultures with cell lines and inhibition of colony formation using 3-dimensional (3-D) organoid cultures with cells from patient-derived xenograft (PDX) GBM tumor models and PDX breast cancer models.

The effect of CC-90010 on colony formation in 14 PDX-derived GBM tumor models was assessed using an in vitro neurosphere assay. CC-90010 was tested at concentrations ranging from 0.0003 µM to 20 µM in 3-fold increments. Colony formation was assessed after 7 days of treatment by quantifying colony numbers by microscopy. CC-90010 inhibited colony formation

EDMS Doc. Number: [redacted]

in a dose-dependent manner yielding mean half-maximal inhibitory concentration (IC_{50}) values ± standard error of the mean (SEM) ranging from 0.11 ± 0.04 µM to 2.00 ± 0.40 µM and spanning an 18-fold activity range. The overall mean for the GBM models was 0.62 ± 0.13 µM.

The effect of CC-90010 on colony formation in 4 PDX-derived breast cancer models was assessed using a 3-D Matrigel-based in vitro culture system. CC-90010 was tested at concentrations ranging from either 0.008 µM to 5 µM or 0.0016 µM to 1 µM in 5-fold increments. Colony formation was assessed after 7 or 14 days of treatment by quantifying colony numbers by microscopy. CC-90010 inhibited colony formation in a dose-dependent manner yielding a mean IC_{50} value for the BR0869f estrogen receptor (ER) negative, progesterone receptor (PR) negative, and HER2/neu positive (ER-PR-Her2+) tumor model of 0.12 ± 0.01 µM and IC50 values for the COH69, COH71, and TNBR3 triple negative breast cancer (TNBC) models of 0.07 µM, 0.18 ± 0.02 µM, and 0.08 ± 0.00 µM, respectively. The overall mean for the three TNBC models was 0.11 ± 0.04 µM.

1.2.1.3. In Vivo Pharmacology

In mouse studies, CC-90010 has demonstrated dose-dependent tumor growth inhibition (TGI) in PDX of TNBC and GBM tumors. Additionally, using limiting dilution assays, a decrease in tumor initiating cell (TIC) frequency has been shown following treatment with CC-90010 (performed with a daily dosing schedule and not included in the Clinical Trial Application).

Different doses and schedules of CC-90010 were evaluated pre-clinically. CC-90010 dosed on a 3 days on /4 days off schedule showed TGI efficacy equivalent to that seen in the continuous dosing schedules as well as improved tolerability relative to continuous dosing schedules. Body weight, gastrointestinal (GI), and bone marrow (BM) toxicities appeared fully reversible by less frequent dosing schedules, and recovery was suitable for weekly repeat dosing.

Treatment of mice bearing COH70, a TNBC PDX tumor, with CC-90010 at 2 or 10 mg/kg resulted in down-regulation of MYC. CC-90010 at 2 mg/kg maximally suppressed MYC expression by 51.3% at 2 hours, with MYC expression rebounding to control levels by 8 hours postdose. CC-90010 at 10 mg/kg maximally suppressed MYC expression by 63.4% at 4 hours; however, MYC expression did not rebound to control levels by 24 hours postdose. Corresponding tumor concentrations of CC-90010 were determined in the COH70 model at 2, 4, and 8 hours postdose. Maximally-measured tumor levels of CC-90010 were at 2 hours postdose and were 1.3 ± 0.3 µM and 6.7 ± 1.7 µM at 2 mg/kg and 10 mg/kg, respectively. Modulation of MYC expression levels correlated with intra-tumor concentrations of CC-90010.

1.2.1.3.1. Tumor Growth Inhibition by CC-90010 in Xenograft Models of TNBC and GBM Tumors

A TNBC PDX subcutaneous model was noted to have significant TGI in NOD/SCID gamma (NSG) mice at CC-90010 doses of 12.5, 16, and 20 mg/kg. Dosing was orally by gavage once daily (QD) for 3 consecutive days followed by 4 days off (designated as 3x/week in Figure 1) each week for 6 weeks. CC-90010 was well tolerated up to a daily dose of 25 mg/kg. When tumor volumes were measured on Day 38, compared to vehicle control, the mean percent TGI of treated tumors was 64% for 12.5 mg/kg/dose group, 68% for the 16 mg/kg/dose group, and 72% for the 20 mg/kg/dose group. Mean body weights increased in all groups. Steady state PK parameters were determined following the final doses for the 12.5 mg/kg and 16 mg/kg dose

levels. The area under the plasma concentration-time curve between 0 and 24 hours (AUC_{0-24hr}) of CC-90010 at 12.5 mg/kg was 12,003 ng·hr/mL and at 16 mg/kg was 15,174 ng·hr/mL.

Figure 1: Dose-Dependent Tumor Growth Inhibition as Measured by Tumor Volume in a Triple-negative Breast Cancer Patient-derived Xenograft Model, COH70, following Dosing with CC-90010

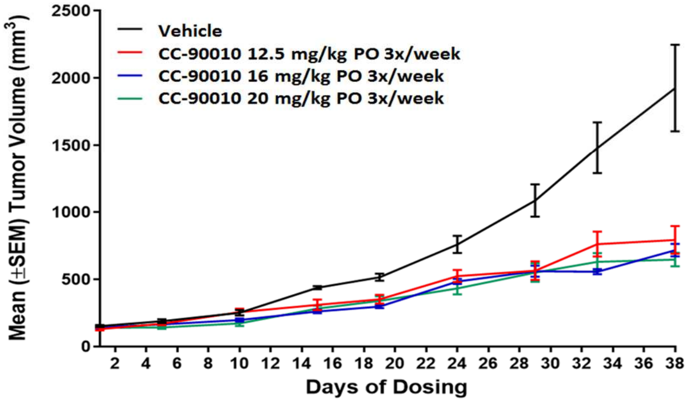

3x/week = 3 consecutive days of once daily CC-90010 dosing followed by 4 days off; PO = by mouth; SEM = standard error of the mean.
Source: Unpublished data from Celgene Corporation.

In a GBM PDX subcutaneous model, GBM15, efficacy of CC-90010 was shown on several schedules ranging from dosing 5 times QD weekly to twice weekly for 4 weeks (refer to Figure 2). Mice bearing tumors were dosed orally QD on several schedules with the cumulative weekly CC-90010 dose on each schedule equal to 75 mg/kg. Dosing schedules were:

- 15 mg/kg CC-90010 for 5 consecutive days on and 2 days off (5/2),
- 25 mg/kg CC-90010 for 3 consecutive days on and 4 days off (3/4), and
- 37.5 mg/kg CC-90010 for 2 consecutive days on and 5 days off (2/5).

When tumor volumes were measured on Day 29, compared to vehicle control, the mean percent TGI of treated tumors were 65% for the 15 mg/kg/dose (5/2) group, 65% for the 25 mg/kg/dose (3/4) group, and 70% for the 37.5 mg/kg/dose (2/5) group. Minimal weight loss was seen in all groups (vehicle group = -1.2%, 15 mg/kg/dose group = -6.6%, 25 mg/kg/dose group = -3.7%, and 37.5 mg/kg/dose group = -3.1%).

Figure 2: Dose-Dependent Tumor Growth Inhibition as Measured by Tumor Volume in a Glioblastoma Multiforme Patient-derived Xenograft Model, GBM15, following Dosing with CC-90010

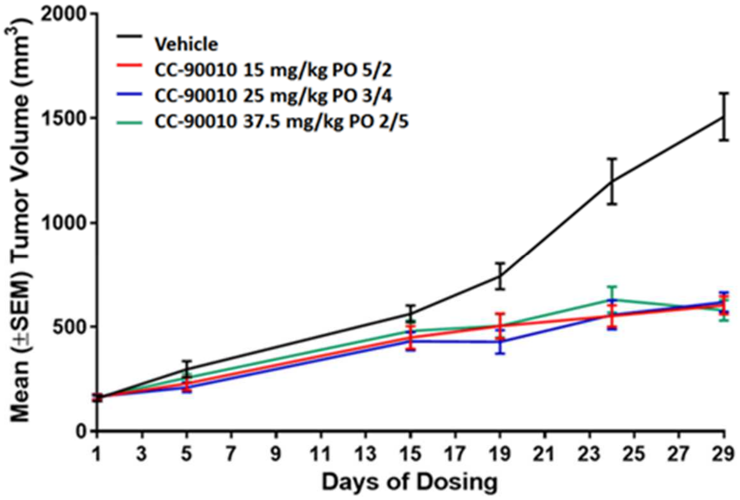

5/2 = 5 consecutive days of once daily CC-90010 dosing followed by 2 days off; 3/4 = 3 consecutive days of once daily CC-90010 dosing followed by 4 days off; 2/5 = 2 consecutive days of once daily CC-90010 dosing followed by 5 days off; PO = by mouth; SEM = standard error of the mean.
Source: Unpublished data from Celgene Corporation.

1.2.1.3.2. Synergistic Effects of CC-90010 and Temozolomide in a Temozolomide-resistant Xenograft GBM Model

O-6-methylguanylmethyltransferase (MGMT) has been implicated in GBM resistance to the alkylating DNA damage of temozolomide (TMZ). GBM3 is a GBM PDX subcutaneous model with high MGMT expression by PCR, a non-methylated MGMT promoter, and has the phenotype of being resistant to TMZ. In previous studies of neurospheres cultured from GBM3, MGMT was shown to be down-regulated by CC-90010 in a dose-responsive manner using RT-PCR. When mice bearing GBM3 were given a single dose of CC-90010 at 20 mg/kg, MGMT down-regulation was seen by qRT-PCR in the harvested tumor. This led to an efficacy experiment to understand whether CC-90010 could sensitize TMZ-resistant GBM to TMZ and have synergistic effects.

Cohorts of NSG mice bearing GBM3 were treated with TMZ 50 mg/kg intraperitoneal (IP) x 3 Q2 weeks, CC-90010 6 mg/kg orally twice daily (BID) or 12 mg/kg orally once daily, or with a combination of CC-90010 6 mg/kg orally BID and TMZ 50 mg/kg IP x 3 Q2 weeks. Significant tumor growth inhibitions, as measured by tumor volumes, were observed following dosing with CC-90010 alone or in combination with TMZ (refer to Figure 3). TMZ alone did not induce significant TGI when given alone (3%). CC-90010 alone induced significant TGIs of 63% (12 mg/kg QD) and 76% (6 mg/kg BID). The combination of CC-90010 and TMZ demonstrated synergy and was significantly superior to all other regimens in terms of TGI. Moderate weight loss was observed during part of the study course (nadir -5.1%) in the combination group. However, body weight loss recovered, and all treatment groups exhibited net gain in mean body weight at study end.

Figure 3: CC-90010-mediated Tumor Growth Inhibition of GBM3 (Glioblastoma Multiforme Patient-derived Xenograft) with and without Temozolomide (TMZ)

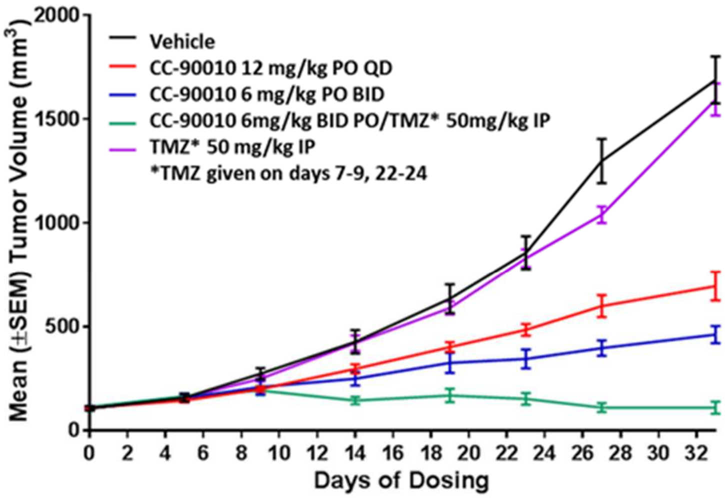

Tumor volumes plotted as mean ± standard error (SEM).

BID = twice daily; IP = investigational product (CC-90010); PO = by mouth; QD = once daily; SEM = standard error of the mean.
Source: Unpublished data from Celgene Corporation.

1.2.2. Nonclinical Pharmacokinetics and Drug Metabolism

In vitro and in vivo studies have been conducted to characterize the absorption, PK, distribution, metabolism and elimination of CC-90010. Robust and reproducible bioanalytical methods for the quantitation of CC-90010 levels were developed and used in PK and toxicokinetic studies. Human PK parameters and exposures were predicted using allometric scaling.

Pharmacokinetics and oral bioavailability of CC-90010 were evaluated in Sprague-Dawley rats and Beagle dogs. The systemic clearance was low (approximately 5% to 13% of liver blood flow). While low in both male and female rats, males showed approximately 2-fold higher clearance than females. The volume of distribution ranged from approximately 1- to 3-fold the total body water volume, suggesting distribution of CC-90010 into tissues. The mean oral bioavailability of CC-90010 was 40% in rats and 76% in dogs. Due to sex differences in systemic clearance between male and female rats and in order to obtain comparable systemic exposure in toxicology studies, CC-90010 doses administered to male rats were 3-fold higher than female rats. Toxicokinetics of CC-90010 in rats and dogs showed no sex differences in systemic exposure, dose-proportional increase in systemic exposure, no accumulation in rats and up to 3-fold accumulation in dogs after repeat dosing. CC-90010 showed limited brain distribution with brain to plasma ratios of 0.14 to 0.16 in tumor bearing NSG mice.

No notable differences in plasma protein binding of CC-90010 were observed in plasmas derived from preclinical species (89.9% to 93.3%) and human sources (90.2%).

The metabolism of CC-90010 was evaluated in vitro using hepatocytes from various species (mouse, rat, rabbit, dog, monkey, and human). Metabolism occurred via multiple pathways including N-dealkylation, O-dealkylation, oxidation, glucuronidation of oxidative metabolites

and combinations of these pathways. All metabolites identified in human hepatocytes were also formed in rat and/or dog, the two species used for pre-clinical safety testing. No unique human metabolites were identified. Studies using recombinant cytochrome P450 (CYP) enzymes, human liver microsomes, monoclonal antibodies and chemical inhibition suggest that CC-90010 metabolism is mediated primarily by CYP3A4/5 with minor involvement of CYP2C8. Hence, drugs that are known strong inducers or inhibitors of CYP3A4/5 should be avoided with CC-90010. Should use of these drugs become necessary, the risks and benefits need to be discussed with the Sponsor's clinical and safety physician prior to administration of CC-90010.

In vitro, CC-90010 had little to no direct inhibitory effect on CYP1A2, CYP2A6, CYP2B6, CYP2E1 and CYP3A4/5. CC-90010 caused direct inhibition of CYP2C8, CYP2C9, CYP2C19, and CYP2D6, with IC_{50} values ≥ 13.9 μM. CC-90010 demonstrated little to no time-dependent inhibition of any of the isozymes tested (CYP1A2, CYP2A6, CYP2B6, CYP2C8, CYP2C9, CYP2C19, CYP2D6, CYP2E1, and CYP3A4/5). Cytochrome P450 induction potential of CC-90010 (0.01 to 10 μM) was evaluated using cultures of cryopreserved human hepatocytes and following incubation up to 3 days. The ability of CC-90010 to induce messenger RNA (mRNA) expression of CYP1A2, CYP2B6 and CYP3A4 was determined. CC-90010 caused no increase in mRNA (< 2 fold over vehicle control), indicating that CC-90010 is not an inducer of CYP1A2, CYP2B6, or CYP3A4. Hence, at clinically relevant concentrations, CC-90010 is not expected to cause any drug-drug interactions due to CYP inhibition or induction. CC-90010 is a substrate of P-gp and not a substrate of BCRP, MRP2, OAT1, OAT3, OATP1B1, OATP1B3 and OCT2. CC-90010 is not an inhibitor of BCRP or MRP2. CC-90010 inhibited P-gp, OATP1B1 and OCT2 with an IC_{50} of 2 μM, 4.94 μM, and 8.24 μM, respectively. CC-90010 showed 45.6%, 53.6%, and 58.3% inhibition for OAT1, OAT3, OATP1B3 mediated transport at 30 μM concentration, respectively. At clinically relevant doses and concentrations, the potential for interactions due to inhibition of transporters is minimal. However, there is some potential for interaction with P-gp substrates at the gut level and narrow therapeutic index drugs that are substrates of P-gp (eg, digoxin and apixaban) should be avoided.

In rats, following intravenous (IV) administration of non-radiolabeled CC-90010, an average of 0.9% of the dose was excreted intact either in bile or urine, indicating that excretion of intact drug is not the primary mode of elimination and that metabolism may play a major role in disposition of CC-90010.

1.2.3. Nonclinical Toxicology

CC-90010 has been evaluated in non-GLP exploratory toxicology and genetic toxicology studies, and in GLP repeat-dose (up to 4 weeks) studies. The GLP 4-week oral toxicity studies (with a 4-week recovery period) were conducted in rats [0, 5, 10, or 20 mg base/kg/dose (females); 0, 15, 30, or 60 mg base/kg/dose (males)] and Beagle dogs (0, 1.75, 3.75, or 7.5 mg base/kg/dose). The dosing schedule was once daily administration for 3 consecutive days followed by 4 consecutive days off drug each week for a total of 4 weeks. CC-90010 dosed on a 3 days on / 4 days off schedule showed TGI efficacy equivalent to that seen in the continuous dosing schedule with improved tolerability relative to continuous dosing schedule in PDX mouse models. Body weight, gastrointestinal (GI), and bone marrow (BM) toxicities appeared fully reversible by less frequent dosing schedules, and recovery was suitable for weekly repeat dosing.

In rats, the primary target tissues of toxicity were those that make up the GI tract (erosion, ulceration, and/or hemorrhage [nonglandular stomach mucosa]; necrosis and hemorrhage [glandular stomach mucosa]; blunting/fusion of villi [small intestine], and hemorrhage [small intestine mucosa]; hemorrhage [large intestine mucosa]), bone marrow (hypocellularity), lymphoid organs (lymphocyte depletion), testes (vacuolation of Sertoli cells [seminiferous tubules]), and bone (hypertrophy [femoral physis]). In dogs, the primary target tissues of toxicity were similar to those identified in the rat and consisted of those that make up the GI tract (ulceration and/or hemorrhage [stomach]; shortened villi and/or decreased crypt depth [small and/or large intestine]), bone marrow (hypocellularity), lymphoid organs (lymphocyte depletion), and testes (vacuolation [seminiferous tubules]).

In the 4-week rat study the severely toxic dose was ≥ 20 mg base/kg/dose. These doses resulted in the death or moribund sacrifice of animals as early as Day 6, ultimately leading to termination of dosing and sacrifice of the surviving 60 mg base/kg/dose group animals (males) on Day 11; and the termination of dosing and sacrifice of (or start of recovery phase for) the surviving 20 mg base/kg/dose group animals (females) on Day 11. There were no CC-90010-related mortalities at doses below 20 mg base/kg/dose.

Based upon the constellation of clinical, laboratory, gross pathologic, and histopathologic findings, the severely toxic dose in 10% of the rats (STD10) was 20 mg base/kg/dose in females and 30 mg base/kg/dose in males. For any clinical trial, the overarching rat STD10 should be considered 20 mg base/kg/dose. This value applies to the 3 days-on/4 days-off CC-90010 dose schedule. Evaluation of recovery animals demonstrated that all test article-related findings were reversible after a period of 4 weeks from the cessation of dosing (with the exception of the testis-related findings which could not be evaluated due to the moribund sacrifice of the 60 mg base/kg/dose group males originally designated to evaluate reversibility).

In the 4-week Beagle dog study, the severely toxic dose was 7.50 mg base/kg/dose. This dose resulted in the moribund sacrifice of animals (4 males and one female) as early as Day 11, ultimately leading to termination of dosing of the surviving 7.50 mg base/kg/dose group males, and the start of recovery phase for the surviving 7.50 mg base/kg/dose group males. There were no CC-90010-related mortalities at doses below 7.50 mg base/kg/dose, but there were CC-90010-related findings at all doses evaluated.

Based upon the constellation of clinical, laboratory, gross pathologic, and histopathologic findings, 3.75 mg base/kg/dose was established as the dog highest non-severely toxic dose (HNSTD). These values apply to the 3-days-on/4-days-off CC-90010 dose schedule. At the lowest dose (1.75 mg base/kg/dose), adverse findings were limited to decreased thymus weights and testicular/epididymal toxicity. Evaluation of recovery animals demonstrated that all test article-related findings were reversible after a period of 4 weeks from the cessation of dosing with the exception of the testis- and epididymis-related findings.

Safety pharmacology evaluations were performed to determine the potential cardiovascular and respiratory effects of CC-90010 in conscious Beagle dogs as part of the GLP 4-week repeat dose toxicity study. There were no CC-90010-related effects on electrocardiograms, heart rate, or respiratory rate. An in vitro human ether-à-go-go-related gene (hERG) study identified an IC50 of 24.3 µM.

Safety pharmacology evaluations, ie, functional observational battery (FOB), were also performed to determine the potential central nervous system effects of CC-90010 as part of the GLP 4-week repeat-dose toxicity rat study. There were no CC-90010-related FOB effects.

In a non-GLP bacterial reverse mutation assay (Ames), CC-90010 was determined to be non-mutagenic.

Overall, CC-90010 exhibits an acceptable safety profile in preclinical species for an oncology clinical candidate, and the toxicology program for CC-90010 adequately supports the conduct of clinical trials in cancer patients.

1.3. Summary of Clinical Data with CC-90010

As of the 02 Dec 2019 clinical data cutoff date, preliminary clinical safety and efficacy data are available for 69 subjects treated in Part A (dose escalation) and 12 subjects treated in Part B (dose expansion) of Study CC-90010-ST-001.

1.3.1. CC-90010-ST-001 Part A – Dose Escalation

A total of 69 subjects with advanced or unresectable solid tumors, and relapsed and/or refractory NHL from 11 dose cohorts have been treated as of the cutoff date. The median duration of treatment was 8 weeks (range: 1 to 96 weeks) with a median of 2 cycles (range: 1 to 24 cycles). The number of subjects treated in each cohort are as follows:

- 7 subjects treated in Dose Level 1 (15 mg, 3-days-on/4-days- off)
- 7 subjects in Dose Level 2 (15 mg, 3-days-on/11-days-off)
- 4 subjects in Dose Level 3A (25 mg, 3 days on/11-days-off)
- 6 subjects in Dose Level 3B (30 mg, 4-days-on/24-days-off)
- 6 subjects in Dose Level 3C (15 mg 2-days-on/5-days-off)
- 7 subjects in Dose Level 4A (40 mg, 3 days on/11-days-off)
- 7 subjects in Dose Level 4B (45 mg, 4-days-on/24-days-off)
- 7 subjects in Dose Level 4C (25 mg 2-days-on/5-days-off)
- 6 subjects in Dose Level 5A (30 mg, 3 days on/11-days-off)
- 6 subjects in Dose Level 5B (55 mg, 4-days-on/24-days-off)
- 6 subjects in Dose Level 5C (35 mg 2-days-on/5-days-off)

Overall, 6 subjects experienced at least one dose-limiting toxicity (DLT) in Part A. The initial Dose Level 1 selected for this study was not well tolerated with one on-target DLT of Grade 4 decreased platelet count associated with Grade 3 skin hemorrhage, as well as several events of thrombocytopenia Grades 1 to 3, and events of hyperglycemia requiring metformin, and asthenia. This led to the opening of Dose Level 2 at a reduced dose intensity of 15 mg/dose, 3-days-on/11-days off schedule, which showed a much better tolerated profile with no DLT. Three alternative dosing schedules were further escalated in parallel: 3-days-on/11-days off (Schedule A), 4-days-on/24-days-off (Schedule B), and 2-days- on/5-days-off every week (Schedule C), on a 28-day cycle. In Dose Level 3B, one subject developed treatment- related

Grade 3 alanine aminotransferase (ALT) elevation that fulfilled the criteria for DLT; no DLTs were observed in Dose Levels 3A and 3C. In Dose Level 4A, one subject had a DLT of Grade 3 ALT elevation and three subjects total had treatment- related dose delays in Cycle 1 which were assessed as DLTs per protocol. This dose was declared as non-tolerated by the Safety Review Committee (SRC) and Dose level 5A was opened at a reduced dose intensity of 30 mg, 3-days-on/11-days-off. Dose level 5A was well tolerated and was declared as the maximum tolerated dose (MTD) for the 3-days-on/11-days-off dosing schedule. Dose Level 4B was generally well tolerated with only one subject having a DLT of Grade 4 thrombocytopenia, which led to the opening of Dose Level 5B. However, Dose Level 5B was declared a non-tolerated dose by the SRC, with one subject developing multiple DLTs of Grade 4 thrombocytopenia, Grade 4 increased blood creatine phosphokinase, Grade 3 worsening diabetes mellitus, Grade 3 diarrhea and Grade 3 stomatitis. Hence, Dose Level 4B was declared as the MTD by the SRC for the 4-days-on/24-days-off schedule. Dose level 4C and 5C on the other hand had 2 subjects each who had dose reductions in Cycle 1 and were both declared as non-tolerated; another subject in Dose Level 5C had a Grade 3 fatigue that met DLT criteria.

Dose escalation has been completed and the NTDs and MTDs were determined for the 3 schedules. The MTDs are 30 mg/dose for Schedule A (180 mg per 28-day cycle), 45 mg/dose for Schedule B (180 mg per 28-day cycle), and 15 mg/dose for Schedule C (120 mg per 28-day cycle). CC-90010 was well tolerated at dose levels below 200 mg dose intensity per 28-day cycle in these heavily pretreated subjects with advanced tumors. Overall, the majority of TEAEs were reversible, and manageable by dose adjustments and/or supportive treatment. There was no clear dose/toxicity relationship within same dose level cohorts with marked individual susceptibility to develop toxicity especially thrombocytopenia and liver toxicity. Schedule C weekly schedule (2-days-on/5-days-off) was less well tolerated at equal dose intensity per 28-day cycle compared to Schedules A and B, with multiple dose reductions mainly due to thrombocytopenia.

As of 02 Dec 2019 clinical data cutoff date, in the Treated population (N = 69), all subjects experienced at least one TEAE and the most frequently reported TEAEs (≥ 10% of subjects) were thrombocytopenia in 35 (50.7%) subjects, diarrhea in 29 (42.0%) subjects, nausea in 28 (40.6%) subjects, asthenia in 27 (39.1%) subjects, dysgeusia in 20 (29.0%) subjects, stomatitis, vomiting, fatigue, and decreased appetite in 17 (24.6%) subjects each, hyperglycemia in 15 (21.7%) subjects, anemia in 11 (15.9%) subjects, abdominal pain in 10 (14.5%) subjects, increased ALT, dermatitis acneiform, and constipation in 9 (13.0%) subjects each, and headache and maculopapular rash in 7 (10.1%) subjects each. Overall, 40 (58.0%) subjects experienced at least one Grade 3 or 4 TEAE (30 subjects with Grade 3 and 10 subjects with Grade 4), and the most frequently reported Grade 3 or 4 TEAEs (in ≥ 2 subjects) were thrombocytopenia in 9 (13.0%) subjects, asthenia and hyperglycemia in 4 (5.8%) subjects each, anemia and increased ALT in 3 (4.3%) subjects each, decreased platelet count, fatigue, decreased performance status (PS), hypokalemia, hyponatremia, arthralgia, back pain, cognitive disorder, and dyspnea in 2 (2.9%) subjects each.

Overall, 60 (87.0%) subjects experienced at least one TEAE assessed as related to the study treatment by the investigator, and the most frequently reported treatment-related TEAEs (≥ 10% of subjects) were thrombocytopenia in 32 (46.4%) subjects, diarrhea and nausea in 27 (39.1%) subjects each, asthenia in 22 (31.9%) subjects, dysgeusia in 18 (26.1%) subjects, stomatitis in 16 (23.2%) subjects, fatigue in 15 (21.7%) subjects, vomiting and decreased appetite in 12 (17.4%) subjects each, dermatitis acneiform in 9 (13.0%) subjects, and

hyperglycemia and anemia in 8 (11.6%) subjects each. Twenty-three (33.3%) subjects experienced at least one treatment-related Grade 3 or 4 TEAE, and the most frequently reported treatment-related Grade 3 or 4 TEAEs (in ≥ 2 subjects) were thrombocytopenia in 9 (13.0%) subjects, anemia in 3 (4.3%) subjects, and decreased platelet count, fatigue, increased ALT, and hyponatremia in 2 (2.9%) subjects each.

As of the cutoff date, 32 (46.4%) subjects experienced at least one serious TEAE, and the most frequently reported serious TEAE (in ≥ 2 subjects) were thrombocytopenia in 2 (2.9%) subjects and decreased platelet count in 1 (1.4%) subject, cognitive disorder and headache in 3 (4.3%) subjects each, and anemia, diarrhea, fatigue, general physical health deterioration, noncardiac chest pain, and arthralgia in 2 (2.9%) subjects each (the clinical team decided to combine the counts for thrombocytopenia and decreased platelet count) . Overall, 8 (11.6%) subjects experienced at least one serious TEAE assessed as related to study treatment, and the most frequently reported treatment-related serious TEAE (in ≥ 2 subjects) were thrombocytopenia in 2 (2.9%) subjects and decreased platelet count in 1 (1.4%) subject, and anemia, diarrhea, and fatigue in 2 (2.9%) subjects each (the clinical team decided to combine the counts for thrombocytopenia and decreased platelet count).

Overall, 7 (10.1%) subjects experienced a TEAE leading to death and all were assessed as not related to study treatment, ie, 2 subjects in the 3-days-on/4-days-off schedule, 2 subjects in the 3-days-on/11-days- off schedule, 1 subject at the 4-days-on/24-days-off schedule, and 2 subjects at the 2-days-on/5-days-off schedule. No death due to drug-related toxicity occurred during the study.

As of the 02 Dec 2019 data cutoff date, 1 (1.4%) subject with Grade II astrocytoma had a confirmed and durable CR: this subject was still ongoing with CR at Cycle 17. This subject was treated with CC-90010 at 25 mg on a 2 days on/5 days off schedule at Cycle 1, then at 45 mg on a 4 days on/24 days off schedule during Cycles 2 and 3, and 30 mg on a 4 days on/24 days off schedule at Cycle 4 and thereafter. One (1.4%) subject with endometrial cancer, treated with CC-90010 at 40 mg on a 3 days on/11 days off schedule, achieved a confirmed PR. A total of 23 (33.3%) subjects had SD (22 confirmed), 10 (14.5%) of whom had SD ≥ 4 months (all confirmed). All subjects who had a durable SD (ie, SD of ≥ 4 months) were classified as having solid tumors.

1.3.2. CC-90010-ST-001 Part B - Dose Expansion

As of the 02 Dec 2019 data cutoff date, 12 subjects were enrolled and treated in Part B of the study: 10 subjects in Cohort 1, 1 subject in Cohort 2, and 1 subject with NUT midline carcinoma (hereinafter, Other). The latter was enrolled before site approval of Protocol Amendment 4, which restricted the enrollment in Part B to subjects with relapsed and/or refractory (R/R) DLBCL and advanced BCC.

Overall, the median duration of treatment was 6.0 weeks (range: 4 to 17 weeks) and the median number of cycles administered was 1.5 (range, 1 to 4 cycles). The median duration of treatment was 4.0 weeks for Cohort 1 (R/R DLBCL), 11.9 weeks for Cohort 2 (advanced BCC), and 11.9 weeks for Other (NUT midline carcinoma).

Overall, 10 (83.3%) subjects experienced at least one TEAE and the most frequently reported TEAEs (≥ 10% of subjects) were anemia in 8 (66.7%) subjects, thrombocytopenia in 7 (58.3%)

EDMS Doc. Number: [redacted]

subjects, diarrhea, nausea, and asthenia in 5 (41.7%) subjects each, neutropenia and hyperglycemia in 4 (33.3%) subjects each, vomiting, pyrexia, decreased appetite, and hypomagnesemia in 3 (25.0%) subjects each, and hypophosphatemia in 2 (16.7%) subjects.

Overall, 9 (75.0%) subjects experienced at least one Grade 3 or 4 TEAE (5 subjects with Grade 3 and 4 subjects with Grade 4), and the most frequently reported Grade 3 or 4 TEAEs (in ≥ 2 subjects) were thrombocytopenia in 6 (50.0%) subjects, anemia in 4 (33.3%) subjects, hyperglycemia and pyrexia in 2 (16.7%) subjects each. In Cohort 1 (R/R DLBCL), 8/10 (80.0%) subjects experienced at least one Grade 3 or 4 TEAE with the most frequently reported Grade 3 or 4 TEAEs being thrombocytopenia (in 6 subjects), anemia (in 4 subjects), and hyperglycemia and pyrexia (in 2 subjects each). In Cohort 2 (advanced BCC), no subject had a Grade 3 or 4 TEAE. In Other, 1/1 (100%) subject had at least one Grade 3 or 4 TEAE (Grade 3 tumor pain).

Overall, 9 (75.0%) subjects experienced at least one TEAE assessed as related to the study treatment by the investigator, and the most frequently reported treatment-related TEAEs (≥ 10% of subjects) were thrombocytopenia in 6 (50.0%) subjects, anemia and diarrhea in 4 (33.3%) subjects each, vomiting, decreased appetite, and hyperglycemia in 3 (25.0%) subjects each, and nausea and asthenia in 2 (16.7%) subjects each. In Cohort 1 (R/R DLBCL), 7/10 (70.0%) subjects experienced at least one treatment-related TEAE with thrombocytopenia being most frequently reported event (in 5 subjects). In the 2 other cohorts (Cohort 2 and Other), all treatment-related TEAEs were reported in 1 subject only. Overall, 5 (41.7%) subjects experienced at least one treatment-related Grade 3 or 4 TEAE, and the most frequently reported treatment-related Grade 3 or 4 TEAEs (in ≥ 2 subjects) were thrombocytopenia in 5 (41.7%) subjects, and anemia and hyperglycemia in 2 (16.7%) subjects each. In Cohort 1 (R/R DLBCL), 5/10 (50.0%) subjects experienced at least one treatment-related Grade 3 or 4 TEAE with thrombocytopenia being most frequently reported event (in 5 subjects). In the 2 other cohorts (Cohort 2 and Other), no treatment-related Grade 3 or 4 TEAEs were reported.

Overall, 7 (58.3%) subjects experienced at least one serious TEAE, and each serious TEAE was reported in 1 subject only. Six out of 10 (60.0%) subjects in Cohort 1 (R/R DLBCL), no subject in Cohort 2 (advanced BCC), and 1/1 (100%) subject in Other experienced at least one serious TEAE. Overall, 2 (16.7%) subjects experienced at least one serious TEAE assessed as related to study treatment, and all serious TEAEs were reported in 1 subject. Two out of 10 (20.0%) subjects experienced at least one treatment-related serious TEAE in Cohort 1. In the 2 other cohorts (Cohort 2 and Other), no treatment-related serious TEAEs were reported. No TEAE leading to death and no death due to drug-related toxicity were reported in Part B of the study.

As of the 02 Dec 2019 data cutoff date, one subject in Cohort 1 with R/R DLBCL achieved a CR as assessed at the end of Cycles 2 and 4. The 9 other subjects with R/R DLBCL (Cohort 1) did not have postbaseline efficacy assessments as of the data cutoff date. However, one of them achieved a PR as assessed at the end of Cycle 2, on 13 Dec 2019 after the data cutoff date. The subject with NUT midline carcinoma in Other achieved a confirmed SD, and the subject with advanced BCC in Cohort 2 had a confirmed disease progression.

1.3.3. Summary of Clinical PK Data from Study CC-90010-ST-001

Final PK data from Part A of Study CC-90010-ST-001 is available for 69 treated subjects and is provided in the Investigator's Brochure.

Following oral administration of CC-90010, geometric mean peak exposure to CC-90010, based on C_{max}, increased with dose on Day 1 (single dose) and after last dose (repeated dosing), in a manner approximately proportional with dose. Across all dose levels and both sampling occasions, C_{max} was observed a few hours after dosing, with median t_{max} ranging from 1 to 2 hours postdose. The terminal elimination half-life, where estimated, was generally similar across the dose schedules, ranging from approximately 40 to 70 hours, after repeated dosing of CC-90010. The CL_{ss}/F and V_{ss}/F ranged from 1.6 to 6.1 L/h and 133.8 to 553.4 L, respectively, across the dose levels, after repeated dosing of CC-90010. There was evidence of accumulation after repeated dosing of CC-90010 for all dose schedules and dose levels, with accumulation ratios of C_{max} and AUC_{0-24} ranging from 1.2 to 2.9, which is expected given the long $t_{½}$ of CC-90010.

Between-subject variability across the dose levels ranged from 22.0% to 91.7% on Day 1 based on C_{max}, and 4.0% to 94.8% based on C_{max} and AUC_{0-last} after the last dose, with the 25 mg (2 days on/5 days off) dose level demonstrating the greatest variability on both occasions. Cerebrospinal fluid samples were collected for one subject, who had quantifiable cerebrospinal fluid concentrations of CC-90010, with an average time matched CSF/plasma ratio of 6.6%.

1.4. Safety Monitoring Plan

Potential toxicities for CC-90010 are being identified based on nonclinical studies with CC-90010 and preliminary data as of the 02 Dec 2019 data cutoff date from the CC-90010-ST-001 clinical study. The safety profiles reported in the literature of two BET inhibitors tested in Phase 1 FIH studies reveal good tolerability with continuous daily dosing for 14 days in each 21-day cycle with thrombocytopenia as major DLT (Abramson, 2015; Herait, 2015) or GI tract toxicity (mainly diarrhea) as DLT (Dombret, 2014; Herait, 2015; Berthon, 2016).

The frequency and caliber of safety assessments proposed for CC-90010-ST-001 are typical of those expected for a Phase 1 study and consistent with findings on toxicological studies of CC-90010 in rats and dogs and clinical data from Parts A, B, and C. In rats and dogs, the primary target tissues of toxicity were the GI tract, bone marrow, lymphoid organs, and testes. The overall pre-clinical and the histopathology data suggest that the GI system may be the key target of CC-90010-mediated toxicity.

Frequent early monitoring of subjects' weight, hydration status, serum electrolytes, the incidence and severity of diarrhea and emesis, as well as episodes of abdominal pain (gastric, intestinal) are critical components of the safety monitoring plan and implementation of aggressive supportive care measures for the early onset (ie, Grade 1) of nausea, vomiting or diarrhea are highly recommended. Based on the morphologic changes, flattening of the intestinal villi, and the mucosal erosions observed in the GI tract of rats and dogs, subjects with malabsorption syndromes, active ulcer/gastritis, or recurring episodes of GI bleeding will be excluded from enrollment. Mucosa coating agents for protection of esophageal/gastric mucosa will be recommended at the discretion of the Investigator as well as monitoring subjects for GI bleeding. Subjects will be encouraged to report episodes of GI discomfort or pain, appetite loss, or blood in stool.

Bone marrow hypocellularity and lymphoid tissue (thymus, spleen, lymph nodes) depletion findings emphasize the importance of frequent blood count monitoring, with platelets and white blood cell (WBC) differential. Subjects will be monitored for possible toxicity through standard

and specialized laboratory tests including complete blood counts, prothrombin time (PT)/ activated partial thromboplastin time (APTT)/international normalized ratio (INR) and serum chemistries.

Transient changes in blood glucose were observed in only a few occasions in the nonclinical toxicology studies with CC-90010. Furthermore, clinical data of a new investigational BETi, OTX015, reported that 1 of 45 subjects with nonleukemic hematologic malignancies experienced Grade 1 or 2 related hyperglycemia and 3 of 45 subjects had Grade 3 related hyperglycemia (Thieblemont 2014; Amorim, 2016). . In Part A of the Study CC-90010-ST-001, 15 (21.7%) subjects experienced hyperglycemia (treatment-related in 8 subjects) and 4 (5.8%) subjects experienced Grade 3 hyperglycemia (treatment-related in 1 subject). In Part B of the study, 4 (33.3%) subjects experienced hyperglycemia (treatment-related in 3 subjects), 1 (8.3%) subject experienced Grade 3 hyperglycemia, assessed as treatment-related, and 1 (8.3%) subject experienced Grade 4 hyperglycemia, assessed as treatment-related.

The standard laboratory panel will include fasting glucose measurements. General guidelines for the management of possible hyperglycemia are provided in Appendix I.

The histopathological findings in testes (rats and dogs)- and epididymides (dogs) will warrant prohibition of semen donation and fathering children for the duration of the clinical study as well as for at least 106 days after the last study dose. There were no histologic lesions in reproductive organs of female animals in the nonclinical studies. The significance of this pre-clinical finding and the potential and relative clinical risk is unknown at this time. Developmental and reproductive toxicology studies have not been conducted with CC-90010. Subjects will be required to follow the pregnancy prevention guidelines as described in Section 6.1.

Subjects will be monitored for possible occurrence of hypotension, headache, flushing, vision changes, hearing changes and priapism and will be advised to report any such conditions to their physician. In addition, subjects will be monitored for occurrence of new or clinically significant worsening of pre-existing insomnia and/or mood disturbances.

Comprehensive studies to evaluate the phototoxicity potential of CC-90010 have not been conducted. As a precautionary measure, it is recommended that subjects avoid prolonged exposure to ultraviolet (UV) light, wear protective clothing and sunglasses, and use UV-blocking topical preparations while taking CC-90010.

Subjects with a history of heart failure, ischemic heart disease, uncontrolled hypertension, serious cardiac arrhythmias, or long QT interval on ECG will be excluded from enrollment. All study subjects will require documentation of adequate left ventricular ejection fraction (> 45%) at baseline.

1.5. Rationale

1.5.1. Study Rationale and Purpose

CC-90010 is a new investigational product (IP) that has a strong biological rationale for the treatment of subjects with solid tumors and NHLs (refer to Section 1.2). The safety and tolerability of CC-90010 in humans, as well as the biologic and clinical activity, will be evaluated in this study. The study will be conducted in three parts: dose escalation (Part A), dose expansion (Part B) and evaluation of food effect (Part C, Spain only).

EDMS Doc. Number: [redacted]

1.5.2. Rationale for the Study Design

In Part A, a Bayesian logistic regression model (BLRM) utilizing escalation with overdose control (EWOC) will guide dose escalations to an estimated MTD for CC-90010 (Babb, 1998; Neuenschwander, 2008). For traditional escalation designs (eg, 3+3, rolling six, accelerated titration) dose escalation rule for the next cohort is based on toxicity rates observed at the current dose only.

The BLRM with EWOC allows for historical data to be utilized in combination with all Cycle 1 DLT information from current and previous cohorts. It uses more information and thus can provide more accurate estimate of MTD and potentially decrease the number of subjects treated at subtherapeutic or intolerable doses (Tourneau, 2012). The use of EWOC will provide rules or restrictions to reduce the probability of dosing beyond the MTD. Additional details of the design are presented in Section 9 and Appendix K. The use of Bayesian response adaptive models for Phase I studies has been advocated by the EMEA guideline on small populations (EMEA CHMP, 2006) and by Rogatko, et al (Rogatko, 2007). One or more dosing regimens and/or disease subsets may be selected for cohort expansion in Part B to obtain additional safety and efficacy information for larger cohorts of subjects.

1.5.3. Rationale for the Food Effect Evaluation (Part C)

In Part C, the impact of concomitant food intake on the PK and safety profile of CC-90010 at the RP2D in subjects with advanced solid tumors will be evaluated. The results from this evaluation will support the recommendation of CC-90010 administration regarding food intake.

The ICH M3 (R2) (Guidance on Nonclinical Safety Studies for the Conduct of Human Clinical Trials and Marketing Authorization for Pharmaceuticals) clearly outlines the nonclinical toxicity/safety studies recommended to support human clinical trials in non-oncology patients (e.g. Healthy Volunteers), in order to promote safe and ethical development of new pharmaceuticals. Although the available CC-90010 nonclinical data support clinical trials in subjects with advanced cancer (as outlined in ICH S9 [Nonclinical Evaluation for Anticancer Pharmaceuticals]), information important to safeguard Healthy Volunteers (e.g. No Observed Adverse Effect Levels [NOAELs] from general toxicity studies, a stand-alone core battery of safety pharmacology studies, genotoxicity data [e.g. Ames]) is not available for CC-90010. This data package makes it likely not possible to calculate a clinical dose that would be low enough to be safe and appropriate for Healthy Volunteers and at the same time would be high enough to generate meaningful food effect data at the dose to be used in subsequent studies (RP2D). Therefore, the food effect evaluation is to be performed in subjects with advanced solid tumors.

The food effect evaluation will be conducted as a randomized crossover substudy of CC-90010-ST-001. This balanced crossover design removes the inter-subject variability from the comparison between treatment (fed vs. fasted conditions), while randomly assigning subjects to one of the sequences controls bias that might otherwise influence the comparison.

Rationale regarding the study design and dose are provided in Section 1.5.4 and sample size considerations for food effect sub-study are provided in Section 9.3 .

EDMS Doc. Number: [redacted]

1.5.4. Rationale for Dose, Schedule, and Regimen Selection

Based on the doses and exposures at which the principal treatment-related effects occurred in the GLP-compliant, 4-week rat and dog studies, both species are considered of similar sensitivity to the toxicities associated with CC-90010 administration. The proposed FIH starting dose (and schedule) was 15 mg CC-90010 base, once daily for 3 consecutive days followed by 4 consecutive days off drug every week (3/7-days schedule). This CC-90010 dose was calculated using the approach described in the ICH S9 Guideline entitled “Nonclinical Evaluation for Anticancer Pharmaceuticals (ICH, 2009) and is summarized in Table 3.

The proposed starting dose in humans was lower than $1/10^{th}$ the STD10 in rats, less than $1/6^{th}$ the HNSTD in dogs, and was considered safe based on multiples of exposure (as measured by AUC) in rats and dogs relative to the predicted human exposure at a dose of 15 mg CC-90010 base. As noted in Table 3, the human exposure at 15 mg base was predicted to range from 736 to 2263 ng·hr/mL; these values were approximately 23- to 72-fold lower than the mean exposure corresponding to the rat STD10 (52800 ng·hr/mL) and approximately 4- to 14-fold lower than the mean exposure corresponding to the dog HNSTD (10000 ng·hr/mL). Based on these toxicokinetic data, the proposed FIH starting dose of 15 mg CC-90010 base was expected to have an acceptable safety profile.

Table 3: Proposed Clinical Starting Dose of CC-90010 Based on the Severely Toxic Dose in 10% of the Rats and the Highest Non-severely Toxic Dose in the One-Month Toxicity Study in Dogs

Species	Rat STD10 or Dog HNSTD (mg base/kg)	HED (mg base/kg)	HED (mg base/kg)	Safety Factor	HED/Safety Factor (mg base)[a]	Proposed Clinical Starting Dose (mg base)[b]
Rat	20	3.2	192	10	19	
Dog	3.75	2.1	126	6	21	15

HED = human equivalent dose; HNSTD = highest non-severely toxic dose; STD10 = severely toxic dose in 10% of the animals.

[a] Based on HED conversion factor for a 60-kg person from the FDA Guidance for Industry entitled “Estimating the Maximum Safe Starting Dose in Initial Clinical Trials for Therapeutics in Adult Healthy Volunteers” (FDA, 2005) and the ICH S9 Guideline entitled “Nonclinical Evaluation for Anticancer Pharmaceuticals” (ICH, 2009).

[b] Using allometry derived plasma clearance (1.1 to 3.5 mL/min/kg) and volume of distribution (0.66 to 1.9 L/kg) estimates and assuming 62% oral bioavailability (based on the average from preclinical species), the predicted C_{max} and AUC_{24h} at the intended human starting dose of 15 mg were approximately 78 to 230 ng/mL and 736 to 2263 ng·h/mL, respectively.

Refer to the Investigator’s Brochure for detailed information.

As of 02 Dec 2019, preliminary safety and efficacy data are available for 69 subjects treated in Part A (dose escalation) and 12 subjects treated in Part B (dose expansion) of Study CC-90010-ST-001 (see Section 1.3). Since the initial starting cohort, 3 different schedules have been evaluated at multiple dose levels; all data were assessed both internally and by the Safety Review Committee (SRC), and a maximum tolerated dose (MTD) has been successfully determined for each. The RP2D determined for Part B is 45 mg 4-days-on/24-days-off (180 mg per 28-day cycle), which was well tolerated. At the initial dose level (DL) 1 (15 mg, 3-days-on/4-days-off schedule, weekly), one DLT occurred (Grade 4 decreased platelet count associated with Grade 3

EDMS Doc. Number: [redacted]

skin hemorrhage), as well as several events of Grade 3, Grade 2, and Grade 1 thrombocytopenia, and events of hyperglycemia requiring metformin, and asthenia. The SRC decided to open DL2 at a reduced monthly dose intensity of 15 mg, 3-days-on/11-days-off schedule, which showed a much better tolerability with no DLT.

Three alternative dosing schedules were subsequently escalated in parallel: 3-days-on/11-days-off (Schedule A), 4-days-on/24-days-off (Schedule B), and 2-days-on/5-days-off every week (Schedule C), in each 28-day cycle. Overall, 6 subjects experienced at least one DLT, ie, one subject at DL1 (15 mg/dose, 3-days-on/4-days-off schedule) (n = 7), one subject from Schedule A (n = 24), 3 subjects from Schedule B (n = 19), and one subject from Schedule C (n = 19). CC-90010 monotherapy was well tolerated at dose levels below 200 mg dose intensity per 28-day cycle when treatment holidays were foreseen in this heavily pretreated population of subjects with advanced solid tumors and relapsed and/or refractory lymphoma. The MTD identified for CC-90010 monotherapy on each schedule were 30 mg 3-days- on/11-days-off for Schedule A (180 mg per 28-day cycle), 45 mg 4-days-on/24- days-off for Schedule B (180 mg per 28-day cycle), and 15 mg 2-days-on/5-days off for Schedule C (120 mg per 28-day cycle).

The selection of the RP2D for Part B, of 45 mg 4-days-on/24-days-off (180 mg per 28-day cycle), is based primarily on PK and PD analysis, and safety and tolerability data. At this dose and schedule, optimal target engagement defined as decrease of blood PD marker C-C Chemokine Receptor Type 1 (CCR1) ≥ 50%, as well as optimal exposure (highest monthly area under the curve [AUC]) were achieved. Additionally, acceptable tolerability profile with the least dose interruptions and dose reductions, as well as most rapid recovery to baseline of platelet counts and preliminary antitumor activity were observed at this dose and schedule. Furthermore, there was no clinically significant neutropenia (Grade 3 or 4) observed.

The selected RP2D of 45 mg 4-days-on/24-days-off (180 mg per 28-day cycle) was well tolerated with only one subject experiencing thrombocytopenia Grade 4, considered a DLT, which was due to low bone marrow reserve. No other subjects at this DL developed any clinically relevant toxicity. The next lower DL of 30 mg 4-days-on /24-days-off (120 mg per 28-day cycle) was very well tolerated with only mild episodes of Grade 1 and Grade 2 asthenia and gastrointestinal toxicity. One subject developed one asymptomatic, transient Grade 3 alanine aminotransferase (ALT) increase, which was considered a DLT. No other subjects at this DL developed any clinically relevant toxicity. The lowest explored monthly intensity in Part A of CC-90010-ST-001 was 90 mg per 28-day cycle (15 mg 3-days-on/11-days-off). This DL was very well tolerated with only mild fatigue and only one subject with one episode of self-limited Grade 2 thrombocytopenia.

Based on preliminary activity observed among the first 14 subjects enrolled in Part B, 1 subject with CR and 1 with PR in the initial R/R DLBCL (Cohort 1), this cohort will expand to enroll up to 20-25 subjects. An additional cohort of approximately 15 evaluable subjects with R/R DLBCL (Cohort 3) will be enrolled under an alternative dosing regimen of 30 mg CC-90010 3-days-on/11-days off in each 28-day cycle, which has the same cumulative dose as the selected RP2D (180 mg per 28-day cycle). The selection of the alternative schedule is based primarily on PK and PD analysis, and safety and tolerability data. One subject with endometrial cancer, treated under 3-days on/11-days off schedule during dose escalation, achieved a confirmed PR. At this dose and schedule, optimal target engagement defined as decrease of blood PD marker CCR1 ≥ 50% (+/- 5% coefficient of variation) was achieved 4 hours post last dose in the first cycle.

Based on the PK model developed from Part A of this study, cumulative 28-day plasma exposure is expected to be similar for both the 30 mg 3 days on/11 days off and 45 mg 4 days on/24 days off dosing regimen, given that the 28-day cumulative dose for both regimens is 180 mg. 30 mg CC-90010 3-days on/11-days off was declared the maximum tolerated dose (MTD) for the 3-days- on/11-days off schedule, with no DLTs reported for the 6 subjects enrolled and treated at this dose and schedule. 30 mg CC-90010 3-days on/11-days off has similar safety and tolerability profile as 45 mg 4-days-on/24-days-off. As of the 02 Dec 2019 data cutoff date, % of subjects in Part A with at least one Treatment-emergent Adverse Events (TEAE) Related to Study Drug was 66.7% at 30 mg CC-90010 3-days- on/11-days off compared to 85.7% at 45 mg 4-days-on/24-days-off. Similarly, 33.3% of subjects at 30 mg CC-90010 3-days-on/11-days off had at least one TEAE Grade 3 or 4 Related to Study Drug compared to 28.6% at 45 mg 4-days-on/24-days-off. Exploring a regimen with a short dosing holiday allowing for a second dosing interval within a 28-day cycle is warranted in an aggressive disease like R/R DLBCL.

For the food effect evaluation (Part C), the RP2D of 45 mg 4-days-on/24-days-off (180 mg per 28-day cycle) will be administered to subjects with advanced solid tumors. The food effect evaluation will be performed on Day 4 (last day of dosing) in Cycles 1 and 2 (Cycle 1 Day 4 and Cycle 2 Day 4) (please refer to Section 3.1.3 for details). Based on the biopharmaceutic profile (data on file), CC-90010 shows Biopharmaceutics Classification System (BCS) Category II like behavior, and administration with food is not expected to cause a more than 50% increase in exposures. The planned evaluation of impact of concomitant food intake is on Day 4; as such the overall increase in cumulative exposures over 4 days of dosing is expected to be modest.

1.5.5. Rationale for Pharmacodynamics and Potential Predictive Biomarkers

An exploratory objective of this study is to identify a dose of CC-90010 that is not only safe but that exhibits pharmacologic activity. A set of genes, the expression of which changes upon ex vivo treatment with CC-90010, has been identified in peripheral blood mononuclear cells (PBMCs) and in whole blood. In the current study, changes in the expression of these genes in whole blood and/or other genes in tumor biopsy may provide confirmation that a dose is pharmacologically active and could help distinguish which dose shows the most compelling pharmacologic activity. Additionally, markers of cell viability and apoptosis such as PARP cleavage in tumor tissues may be assessed, if the data permits.

Predictive biomarkers may someday allow prospective identification of patients who are more likely to benefit clinically from CC-90010 as a single agent or combined with other agents. Although the predictive diagnostic analyses in the current trial are exploratory in nature, they could reveal associations between biomarkers and responses that could provide a basis for future diagnostically driven studies. Refer to Section 6.6 for additional information.

1.5.6. Rationale for Tumor Type Selection in Part B

Different tumor types will be selected for the CC-90010 dose expansion cohorts in Part B depending on the results from Part A of the study, pre-clinical efficacy (for data in PDX models of TNBC and GBM tumors, see Section 1.2.1.3) and supportive literature. As a reversible inhibitor of BET family members, expansion cohorts of subjects with relapsed and/or refractory DLBCL and locally advanced basal cell carcinoma (BCC) will be enrolled in Part B.

DLBCL is the most-common subtype of NHL, with an estimated 27,650 new cases in the US in 2016, accounting for approximately 32.5% of all NHL neoplasms diagnosed annually from 1998-2011 (Al-Hamadani, 2015; Teras, 2016). With the addition of rituximab to the cyclophosphamide, doxorubicin, vincristine and prednisone (CHOP) chemotherapy regimen, more than 40% of patients with DLBCL are cured, and benefit from long-term remission and improved overall survival (Coiffier, 2010). However, those with relapsed or refractory disease have poor outcomes. Gene expression profiling has identified biologically and clinically distinct molecular subtypes of DLBCL, which have prognostic significance and potential therapeutic implications: germinal center B-cell (GCB) subtype and the activated B-cell-like (ABC) subtype (Alizadeh, 2000). The ABC subtype has been shown to have inferior outcomes with R-CHOP therapy, with an estimated 5-year overall survival (OS) of 35% as compared to GCB subtype with OS of 60% (Rosenwald, 2002). Biologically, the ABC subtype is characterized by constitutive activation of the NF-κB which promotes lymphomagenesis, while c-MYC plays a significant role in the pathogenesis in many GCB cases (Abedin, 2016).

Concurrent rearrangements of c-MYC and either BCL-2 or BCL-6 (double-hit lymphoma), or both (triple-hit lymphoma), as well as protein overexpression of c-MYC in combination with overexpression of BCL-2, are associated with chemotherapy resistance and low overall survival rates in DLBCL (Ott 2014; Mottok 2015). Given that approximately 5-15% of cases of DLBCL harbor rearrangements of c-MYC, and protein overexpression of c-MYC is seen in the majority of DLBCL cases (Ott, 2014), novel therapeutic approaches targeting these poor-prognosis populations are urgently needed.

Recent studies have demonstrated that inhibition of the BET family of proteins leads to suppression of c-MYC expression. BRD4, a member of the BET family, facilitates transcription of acetylated chromatin via its capacity to recruit protein complexes, including the mediator complex and the super elongation complex. Through this mechanism, BRD4 contributes to malignant transformation by increasing expression of oncogenes such as c-MYC. JQ1, a small molecule BET inhibitor was able to induce cell death, cell-cycle arrest or senescence in DLBCL cell lines, mediated through downregulation of MYC expression and subsequent suppression of MYC target gene expression (Trabucco, 2014). Furthermore, JQ1 was found to suppress tumor growth of DLBCL cells engrafted in mice and improved the survival of engrafted mice.

In clinical studies of other BET inhibitors, objective responses were observed in 5 out of 64 relapsed or refractory lymphomas treated with CPI-0610, including 2 CRs, 3 PRs and 5 patients with > 6 months SD (Blum, 2018). In preclinical studies, CPI-0610 resulted in downregulation of NF-κB. Three of the responses and one of the prolonged SD were in patients with ABC subtype of DLBCL. Thrombocytopenia, a class effect for all BET inhibitors was dose limiting, however, it was reversible. RG6146 demonstrated an ORR of 11% among relapsed/refractory MYC-expressing DLBCL patients (Caimi, 2017). In a study of OTX-015, another small molecule BET inhibitor, three patients with DLBCL achieved durable objective responses (two CRs at 120 mg once a day, and one PR at 80 mg once a day) (Amorim, 2016). Six additional patients (two with DLBCL, four with indolent lymphomas) had evidence of clinical activity, albeit not meeting objective response criteria. Common toxic effects reported in the study were thrombocytopenia (96%), anaemia (91%), neutropenia (51%), diarrhea (47%), fatigue (27%), and nausea (24%). Grade 3-4 adverse events were infrequent other than thrombocytopenia (58%). These data provide rationale for exploration of CC-90010 in DLBCL.

Basal cell carcinoma is the commonest skin cancer globally and its incidence is increasing (Rubin, 2005; ACS, 2015). It is estimated that currently between two and three million non-melanoma skin cancers occur globally each year and approximately 80% are BCCs (WHO, 2015; ACS, 2015). This is probably an underestimate because in the United States where the registry is better documented than most countries, it is estimated that more than 3.5 million new patients are diagnosed with non-melanoma skin cancer annually (ACS, 2015). Furthermore, the incidence in Europe is increasing by 1 per 100,000 per annum (Rubin, 2005; Lomas, 2012; ACS, 2015).

Most BCCs can be cured by topical therapy, surgery or radiotherapy or a combination thereof (Trakatelli, 2014). However a small proportion progress to, or present with, locally advanced, or in less than 1%, metastatic BCC, which is not amenable to such therapy (Alonso, 2006; Danial, 2013; Sekulic, 2012; Bassett-Seguin, 2015). Advanced BCC often causes significant disfigurement and morbidity, with associated physical and psychological problems since it occurs most commonly in sun-exposed areas, such as the head (Wong, 2003). Treatment of advanced and metastatic cases was difficult prior to availability of Hedgehog (Hh) inhibitors.

Aberrant Hh signaling occurs in 95% of BCCs (Migden, 2015b). Hh signaling pathway is initiated when the extracellular Hh protein binds to the transmembrane receptor Patched (PTCH1) and liberates the smoothened homologue (SMO) transmembrane protein (Ingham, 2001; Rubin, 2006). Signaling by SMO mobilizes the normally latent zinc finger transcription factor GLI2, which transactivates the GLI1 promoter (Huangfu, 2005; Haycraft, 2005; Liu, 2005). GLI1 and GLI2 directly activate transcription of Hh target genes, including several involved in cell growth, such as MYCN and CCND1 (Scales, 2009; Oliver, 2003; Tang, 2014). Additionally GLI1 amplifies Hh signaling by activating transcription of GLI2 in a positive feedback loop (Regl, 2002).

Mutations of PTCH1 and SMO have been identified in basal cell nevus syndrome and sporadic BCCs (Hahn, 1996; Gailani, 1996; Unden, 1997; Xie, 1998). In 80-90% of BCC cases, mutations cause loss of function of PTCH1, which normally inhibits the signaling activity of SMO (Alcedo, 1996; Hahn, 1996; Johnson, 1996; Bassett-Seguin, 2015). Another 10% of BCC cases are due to constitutive activation of SMO (Xie, 1998; Bassett-Seguin, 2015; Reifenberger, 2005). These mutations cause constitutive Hh pathway signaling and the resultant expression of GLI1 in basal cells is associated with development of BCC (Dahmane, 1997; Von Hoff, 2009). Therefore agents to inhibit SMO were developed.

Erivedge™ (vismodegib) directly binds to and inhibits SMO (LoRusso, 2011; Sekulic, 2012) and decreases formation of GLI1 (Von Hoff, 2009). Hence it targets BCCs related to both constitutively activated SMO mutations and PTCH1 mutations. Although vismodegib has a 30.3% independently reviewed response rate for metastatic BCC and a 42.9% response rate for locally advanced (La) BCC subjects for whom surgery or radiotherapy is inappropriate, the median duration of response is only 7.6 months and two-thirds of treated subjects did not respond (ERIVEDGE European Public Assessment Report). A recent safety review, with at least 12 months follow up, showed that 36% of subjects withdrew from vismodegib treatment due to adverse events, plus an additional 10% due to subject request (Bassett-Seguin, 2015). Odomzo™ (sonidegib), another SMO inhibitor, has a 58% independently reviewed response rate for locally advanced BCC and the responses appear somewhat more durable, with 39% of LaBCC having investigator-assessed responses lasting at least six months (Migden, 2015a). However 28% of

subjects were discontinued and 32% of subjects had dose adjustments for adverse reactions (ODOMZO European Public Assessment Report). The durability of responses and tolerance to SMO inhibitors are leaving a substantial number of subjects with unmet medical need.

About 20% of BCC subjects develop resistance (Ridky, 2015). This is usually related to Hh pathway reactivation, through SMO mutations. SMO mutations are present in only 15-33% of untreated BCCs compared to 69-77% of resistant BCCs (Atwood, 2015; Sharpe, 2015). The SMO mutations either interfere with the drug binding pocket, increase basal SMO activity or act through concurrent copy number changes in suppressor of fused protein (SUFU) and GLI2 (Atwood, 2015; Sharpe, 2015). A well tolerated agent which could overcome these resistance pathways by targeting downstream of SMO, would be beneficial. In recent published data, despite strong anti-tumor activity of vismodegib in mouse models of BCC, residual tumor cells persist. Tumors displayed robust Hh pathway inhibition during treatment, indicating that the drug continues to block signaling. As such, residual tumour cells have not acquired drug resistance through de novo mutations, but have adopted an identity that no longer relies on Hh signaling (Biehs, 2018).

BRD4 and other BET bromodomain proteins regulate GLI1 transcription downstream of SMO, with BRD4 directly occupying GLI1 and GLI2 promoters (Tang, 2014). This occupancy can be inhibited by BET inhibitors and the BET inhibitor, JQ1, decreases tumor cell proliferation in vitro and in vivo in Hh-driven tumors, even those resistant to SMO inhibition (Tang, 2014). Hence clinical investigation of a BET inhibitor in locally advanced BCC subjects with de-novo or acquired resistance is warranted.

1.5.7. Rationale for Tumor Type Selection in Part C

The food effect evaluation (Part C) is to be performed in subjects with advanced solid tumors. As mentioned in Section 1.5.3, the available data package makes it likely not possible to calculate a clinical dose that would be low enough to be safe and appropriate for Healthy Volunteers, and at the same time, would be high enough to generate meaningful food effect data at the dose to be used in subsequent studies (RP2D). Therefore, the food effect evaluation is to be performed in subjects with advanced solid tumors. The tumor type selection is supported by data from preclinical in vitro and in vivo models where CC-90010 elicited antitumor activity in a variety of solid tumor types (see Section 1.2.1.2 and Section 1.2.1.3). As of the 10 December 2018 clinical data cutoff date, CC-90010 has also demonstrated preliminary evidence of antitumor activity in Part A of this study. One subject with endometrial cancer treated at 40 mg CC-90010, 3-days-on/11-days-off schedule had a partial response (PR) and 19 (27.5%) subjects had stable disease (SD). After the cutoff date, a subject with progressive Grade II diffuse astrocytoma achieved a PR on treatment with CC-90010 at 45 mg 4-days-on/24-days-off (see Section 1.3).

BET protein deregulation has been seen in several tumors, including but not limited to NUT midline carcinoma, leukemia, hepatocellular carcinoma, GBM, CRC, lung adenocarcinoma, prostate and breast cancers (Filippakopoulos, 2010; Lockwood, 2012; Li, 2015; Pastori, 2014, Zuber, 2011; Mani, 2008; Von Burstin, 2009; Hu, 2015).

BRD4 has been validated as a therapeutic target in many malignant tumors including hepatocellular carcinoma, leukaemia, osteosarcoma, pancreatic cancer and salivary gland carcinoma (SGC) (Filippakopoulos, 2010; Wang, 2017). In vitro studies demonstrated that the BET inhibitor JQ1 had no adverse effects on human normal epithelial cells, but inhibited

proliferation, migration and invasion, and induced apoptosis of salivary adenoid cystic carcinoma cells, and down-regulated the expression of BRD4 and c-MYC and BCL-2, two know target genes of BRD4 (Wang, 2017). Among patients with salivary adenoid cystic carcinoma, MYC overexpression was shown to be associated with a shorter disease-free survival (Fuji, 2017). Hence clinical investigation of a BET inhibitor in subjects with some of these tumors is also warranted.

2. STUDY OBJECTIVES AND ENDPOINTS

Table 4: Study Objectives

**Primary Objectives**
The primary objectives of the study are:<br>• To determine the safety and tolerability of CC-90010.<br>• To define the maximum tolerated dose (MTD) and/or the recommended Phase 2 dose (RP2D) of CC-90010.
**Secondary Objectives**
The secondary objectives are:<br>• To provide information on the preliminary efficacy of CC-90010.<br>• To characterize the pharmacokinetics (PK) of CC-90010.<br>• To evaluate the food effect on the PK and safety profile of CC-90010 when administered at the RP2D of 45 mg 4-days-on/24-days-off (180 mg per 28-day cycle) in a fed condition (high-fat and high-calorie meal) relative to a fasted condition.
**Exploratory Objectives**
The exploratory objectives are:<br>• To evaluate the pharmacodynamic (PD) effects of CC-90010 on gene expression in peripheral blood and if available, in tumor samples.<br>• To explore the relationship between CC-90010 dose, plasma exposure, and selected clinical endpoints (eg, measures of toxicities, preliminary activity, and/or biomarkers).<br>• To explore the relationship between baseline, on-treatment, and/or changes in gene expression in tumor samples (if available) and clinical response.<br>• To characterize the principal metabolites of CC-90010 in plasma provided sufficient data are available.<br>Data from exploratory objectives may not be included in the Clinical Study Report.

Table 5: Study Endpoints

Endpoint	Name	Description	Timeframe
Primary	Safety endpoints	• DLTs evaluated using the NCI CTCAE criteria, Version 4.03<br>• MTD<br>• AEs evaluated using the NCI CTCAE criteria, Version 4.03	Dose escalation and expansion
Secondary	Preliminary efficacy	Clinical benefit rate (CBR) determined by response rates by disease-appropriate response criteria	Dose escalation and expansion
		Objective response rate (ORR), duration of response or stable disease, and progression-free survival (PFS)	Dose escalation and expansion
	Overall survival	From randomization to death due to any cause	Dose escalation and expansion
	PK endpoints	Maximum observed plasma concentration (C_{max}), area under the plasma concentration time-curve (AUC), time to maximum plasma concentration (T_{max}), terminal half-life ($t_{1/2}$), apparent clearance (CL/F), and apparent volume of distribution (V_z/F) of CC-90010	Dose escalation and expansion
	Effect of food on the PK of CC-90010 at the RP2D and schedule	Plasma PK parameters such as $C_{max,}$ $T_{max,}$ $AUC_{0-\infty}$, AUC_{0-Last}, $AUC_{0-24,}$ and $t_{½}$ will be estimated following administration of CC-90010 under fasted and fed conditions.	All planned PK time points for the Part C (food effect evaluation) (see Section 5 and Section 6)
Exploratory	PD endpoints	• Gene and protein expression in peripheral blood cell components<br>• Gene and protein expression in tumor tissue, if available	Dose escalation and expansion
	PK endpoints	• Population PK parameters and clinically relevant covariates<br>• Profile of CC-90010 metabolites in plasma<br>• Exposure-response relationships	

DLT = dose-limiting toxicity; MTD = maximum tolerated dose; NCI CTCAE = National Cancer Institute Common Terminology Criteria for Adverse Events; NTD = non-tolerated dose; PK = pharmacokinetic; PD = pharmacodynamic.

3. OVERALL STUDY DESIGN

3.1. Study Design

Study CC-90010-ST-001 is an open-label, Phase 1a, dose escalation and expansion, FIH clinical study of CC-90010 in subjects with advanced or unresectable solid tumors and relapsed and/or refractory advanced NHLs. The dose escalation part (Part A) of the study will explore escalating oral doses of CC-90010 administered as one dosing schedule at a time or ≥ 2 dosing schedules given in parallel to estimate the MTD of CC-90010 (eg, 2-days- on/5-days- off each week, 3-days-on/4-days-off every other week, 4-days on/24 days off, etc.). A BLRM utilizing EWOC (Babb, 1998; Neuenschwander, 2008) will help guide CC-90010 dose escalation decisions with the final decisions made by a safety review committee (SRC).

The expansion part (Part B) will further evaluate the safety and efficacy of CC-90010 administered at or below the MTD in selected expansion cohorts in order to further define the RP2D. CC-90010 will be evaluated as a single agent in the following cohorts:

- Cohort 1: relapsed and/or refractory DLBCL - approximately 20-25 evaluable subjects at 45 mg CC-90010 4-days-on/24-days-off in each 28-day cycle
- Cohort 2: advanced BCC – enrollment stopped due to recruitment challenges
- Cohort 3: relapsed and/or refractory DLBCL - approximately 15 evaluable subjects at 30 mg CC-90010 3-days-on/11-days-off in each 28-day cycle

The food effect assessment (Part C, Spain only) will evaluate the impact of food on CC-90010 when administered at the RP2D of 45 mg 4-days-on/24-days-off (180 mg per 28-day cycle), by comparison of the PK parameters following fasted and fed (high-fat, high- calorie meal) conditions.

Parts A, B and C will consist of 3 periods: Screening, Treatment, and Follow-up periods (refer to Figure 4).

Figure 4: Overall Study Design

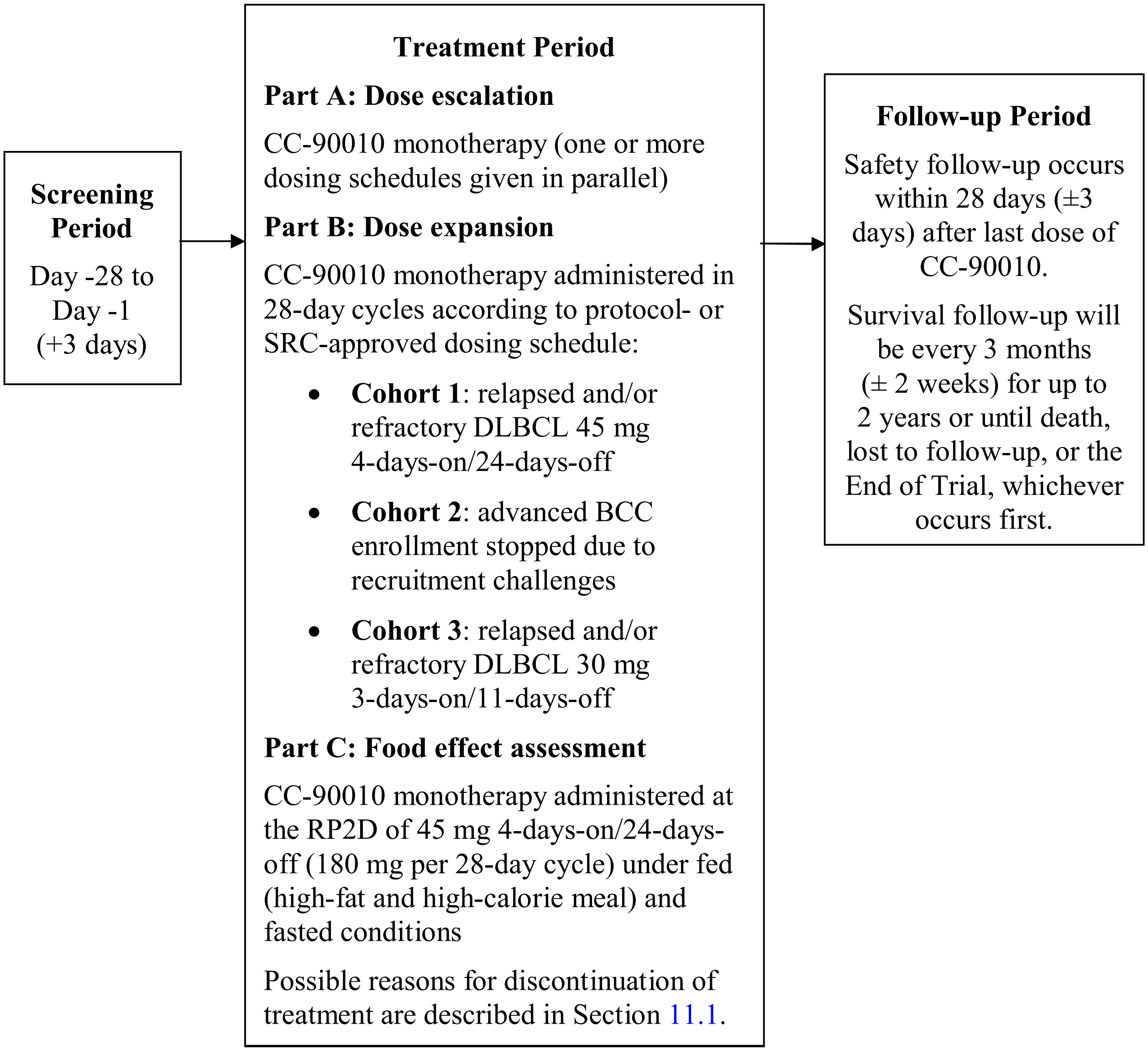

Screening Period

The Screening Period starts 28 days (+3 days) prior to first dose of CC-90010. The informed consent document (ICD) must be signed and dated by the subject and the administering staff prior to the start of any other study-specific procedures. All screening tests and procedures must be completed within the 28 days (+3 days) prior to the first dose of CC-90010.

Treatment Period

During the Treatment Period, CC-90010 was initially administered orally once daily for 3 consecutive days followed by 4 consecutive days off drug every week (3/7-days schedule) in each 28-day cycle in Part A. Alternative dosing schedules (ie, 2-days-on/5-days-off each week,

3-days-on/4-days-off every other week, 4-days- on/24-days-off) administered as one dosing schedule at a time or ≥ 2 dosing schedules given in parallel were evaluated based on the review of available safety, PK, pharmacodynamic (PD), and efficacy data by the SRC. The starting dose and schedule of each alternative dosing schedule will not exceed the dose intensity of a dose cohort that has met the criteria for dose escalation. In Part A, the window for evaluation of dose-limiting toxicity (DLT) will be 28 days (4 weeks) during Cycle 1.

Following completion of dose escalation in Part A, selected expansion cohorts will receive CC-90010 in Part B. The SRC determined the RP2D for Part B to be 45 mg CC-90010 given once daily for 4 consecutive days on followed by 24 consecutive days off (4-days-on/24-days-off) in each 28-day cycle. A cohort of up to approximately 20-25 subjects with relapsed and/or refractory (R/R) DLBCL (Cohort 1)) will be enrolled in Part B expansion. Enrollment in advanced BCC (Cohort 2) will be stopped due to recruitment challenges. An additional cohort of approximately 15 evaluable subjects with R/R DLBCL (Cohort 3) will be enrolled under an alternative dosing regimen of 30 mg CC-90010 3-days- on/11-days off in each 28-day cycle.

The food effect assessment (Part C, Spain only) will evaluate the impact of food on CC-90010 when administered at the RP2D of 45 mg 4-days-on/24-days-off, by comparison of PK parameters following fasted and fed (high-fat, high-calorie meal) conditions in approximately 24 subjects with advanced solid tumors.

Follow-up Period

In the Follow-up Period, all subjects will be followed for 28 days (±3 days) after the last dose of CC-90010 for safety.

Subjects who discontinue treatment for reasons other than disease progression, start of a new anticancer therapy, or withdrawal of consent from the entire study will have disease assessments performed according to the specified tumor assessment schedule until progression and/or initiation of new systemic anticancer therapies.

After the Safety Follow-up visit, all subjects will be followed every subsequent 3 months (± 2 weeks) for survival follow-up for up to 2 years or until death, lost to follow-up, or the End of Trial, whichever occurs first.

3.1.1. Part A Dose Escalation

A minimum of 3 subjects will be enrolled at each dose level. In Part A, the initial CC-90010 dose was 15 mg. The BLRM with EWOC will incorporate available prior safety information and update the model parameters after each new cohort of subjects completes Cycle 1. The decision for the next dose will be made by the SRC based on a calculation of risk assessment using the BLRM, and available safety (ie, DLT and non-DLT safety data), PK, PD, and preliminary efficacy information. Details of the statistical methodology are provided in Section 9 and Appendix K.

At all decision time points, the BLRM permits alterations in the dose increments based on the observed DLTs; however, the dose for the next cohort will not exceed a 100% increase from the prior dose. The MTD is the highest dose that causes DLT in not more than 33% of the subjects treated with CC-90010 in the first Cycle, with at least 6 evaluable subjects treated at this dose. The SRC will make the final decision regarding the CC-90010 dose for each cohort.

EDMS Doc. Number: [redacted]

During dose escalation, a CC-90010 dose can be declared the MTD after meeting the following conditions:

- at least 6 evaluable subjects have been treated at the dose,
- the posterior probability of targeted toxicity (eg, the true DLT rate lying in [0.16, 0.33]) at the dose exceeds 60% or a minimum of 6 subjects have been treated on the study, and
- the dose is recommended according to the BLRM and the SRC approves it.

The SRC will include Investigators (and/or designated representatives), the Sponsor's study physician, safety physician, study statistician, and the study manager. Ad hoc attendees may include the study pharmacokineticist, biomarker scientists and study clinical scientists. Other internal and external experts may be consulted by the SRC, as necessary.

The decision to evaluate additional subjects within a dose cohort, a higher dose cohort, intermediate dose cohorts, smaller dose increments, alternative dosing schedules administered as one dosing schedule at a time or ≥ 2 dosing schedules given in parallel (eg, 2-days- on/ 5-days-off each week, 3-days-on/4-days-off every other week, 4-days- on/24 days off), or declare an MTD will also be determined by the SRC, based on the BLRM assessment and their review of available safety (ie, DLT and non-DLT data), PK, PD, and preliminary efficacy information. The final decision will be made by the SRC.

After the first dose is administered in any cohort during dose escalation, subjects in each cohort are observed for 28 days (Cycle 1, DLT window) before the next dose cohort can begin. No more than one subject per day will be enrolled in a given dose escalation cohort.

In Part A, a subject evaluable for DLT is defined as one that:

- Has received ≥ 80% of the total planned dose amount of CC-90010 during Cycle 1 without experiencing a DLT,

or

- Experienced a DLT after receiving at least one dose of CC-90010.

Subjects non-evaluable for DLT will be replaced.

Intra-subject dose escalation will not be allowed during the DLT assessment period, however, in Cycles ≥ 2, subjects with evidence of clinical benefit (eg, symptom improvement even with concurrent initial increase in lesions) or without evidence of disease progression who are tolerating their assigned dose of CC-90010 may (at the Investigator's discretion and in consultation with the Sponsor's study physician) escalate to the highest dose level shown to be adequately tolerated by at least one cohort of subjects in this study (ie, overdose risk is less than 25% based on the BLRM assessment).

3.1.2. Part B-Cohort Expansion

Following completion of dose escalation (Part A), enrollment in the Part B dose expansion will be limited to the following cohorts:

- Cohort 1: relapsed and/or refractory DLBCL - approximately 20-25 evaluable subjects at 45 mg CC-90010 4-days-on/24-days-off in each 28-day cycle

- Cohort 2: advanced BCC – enrollment stopped due to recruitment challenges
- Cohort 3: relapsed and/or refractory DLBCL - approximately 15 evaluable subjects at 30 mg CC-90010 3-days-on/11-days-off in each 28-day cycle

The SRC determined the RP2D for Part B to be 45 mg CC-90010 given once daily for 4 consecutive days on followed by 24 consecutive days off (4-days-on/24-days-off) in each 28-day cycle. One or more dosing regimens may be selected for cohort expansion. An additional cohort of approximately 15 evaluable subjects with R/R DLBCL (Cohort 3) will be enrolled under an alternative dosing regimen of 30 mg CC-90010 3-days- on/11-days off in each 28-day cycle. The SRC will continue to review safety data regularly throughout the study and make recommendations about study continuation and dose modification, as appropriate.

3.1.3. Part C-Food Effect Assessment

The food effect assessment (Part C, Spain only) will be a randomized 2-sequence, 2-period crossover substudy to assess the impact of concomitant food intake on the PK and safety profile of CC-90010, under fasted and fed conditions. This balanced crossover design removes the inter-subject variability from the comparison between treatment (fed vs fasted), while randomly assigning subjects to one of the sequences controls bias that might otherwise influence the comparison. This design is in accordance with the EMA Guideline on the investigation of drug interactions (EMA CHMP, 2012) and the FDA Guidance on Food-Effect Bioavailability and Fed Bioequivalence Studies (FDA, 2002). Consistent with the RP2D selected for Part B, CC-90010 will be given once daily for 4 consecutive days on followed by 24 consecutive days off (4 days-on/24-days-off) in each 28-day cycle. For specific evaluation of concomitant food intake, on Day 4 of each period (Cycle 1 and Cycle 2), subjects will either receive CC-90010 after an overnight fast lasting at least 10 hours or within 30 minutes of a high-fat, high-calorie breakfast.

Following confirmation of eligibility during screening, subjects will be randomized to one of two treatment sequences administered in 2 separate periods: Treatment A then B, or Treatment B then A, as shown in Figure 5. Period 1 and Period 2 of Part C correspond to Cycle 1 and Cycle 2, respectively. In each period in Part C, 12 subjects will be assigned to Treatment A and 12 subjects will be assigned to Treatment B, leading to a total of 24 subjects for this assessment.

Treatment A and B are as described below:

- Treatment A: Single oral dose of CC-90010 (45 mg) at Hour 0 on Day 4, following a 10 hour overnight fast.
- Treatment B: Single oral dose of CC-90010 (45 mg) at Hour 0 on Day 4, 30 minutes after the start of a high-fat high-calorie breakfast.

Figure 5: Overall Study Design for Part C (Food-Effect Assessment)

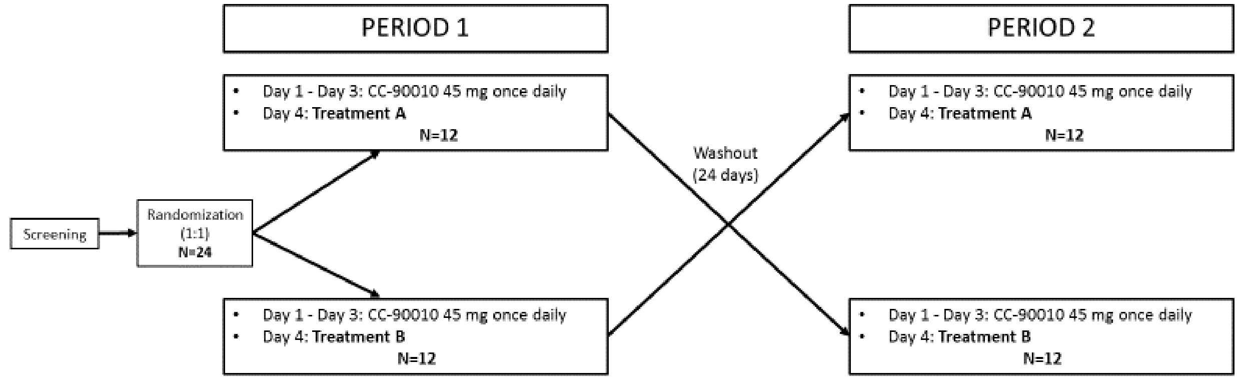

Treatment A: Single oral dose of 45 mg of CC-90010 at Hour 0 on Day 4, following a 10-hour overnight fast.
Treatment B: Single oral dose of 45 mg of CC-90010 at Hour 0 on Day 4, 30 minutes after the start of a high-fat high-calorie breakfast

Within Cycle 1, the 24-days off CC-90010 dosing will serve as a washout period, which is considered sufficient to prevent carryover effect of the treatments. Blood samples will be collected prior to dosing and at several timepoints for up to 18 days (432 hours, corresponding to Day 22 of the cycle) after Day 4 dose in each Period, to measure plasma concentrations of CC-90010. Pharmacokinetic (PK) parameters describing levels of CC-90010 in circulation will be used to assess food effect on bioavailability of CC-90010.

Following Period 2 (Cycle 2), subjects will continue in the treatment period of the study at the RP2D (Figure 4).

For Part C, approximately 24 subjects who complete both treatment Periods 1 and 2 (completers) will be enrolled. Subjects may be replaced at the discretion of the sponsor to ensure 24 completers.

3.1.4. Overview of Assessments

The schedule of assessments is shown in Table 6 and Table 7. The safety variables for this study include adverse events, safety clinical laboratory variables, 12-lead electrocardiograms (ECGs), Eastern Cooperative Oncology Group (ECOG) PS, left ventricular ejection fraction (LVEF) assessments, physical examinations, vital signs, exposure to study treatment, assessment of concomitant medications, and pregnancy testing for females of child bearing potential.

Subjects will be evaluated for efficacy after every 2 cycles through Cycle 6, and then every 3 cycles thereafter. All subjects who discontinue treatment for reasons other than disease progression, start of a new anticancer therapy, or withdrawal of consent from the entire study will be followed until progression and/or initiation of new systemic anticancer therapies.

Blood will be collected at specified timepoints for determining the PK profiles of CC-90010 and for exploratory PD assessments. Paired tumor biopsies for analysis of biomarkers of treatment activity are optional in the dose escalation phase but mandatory during the dose expansion phase.

The study will be conducted in compliance with the International Council on Harmonisation (ICH) of Technical Requirements for Registration of Pharmaceuticals for Human Use/Good Clinical Practice (GCP) and applicable regulatory requirements.

3.2. Study Duration for Subjects

Enrollment is expected to take approximately 38 months to complete (up to 18 months for dose escalation and up to 20 months for expansion). Completion of active treatment and post-treatment follow-up is expected to take an additional 6 to 33 months. The entire study is expected to last approximately 6 years.

3.3. End of Trial

The End of Trial is defined as either the date of the last visit of the last subject to complete the post-treatment follow-up, or the date of receipt of the last data point from the last subject that is required for primary, secondary and/or exploratory analysis, as prespecified in the protocol, whichever is the later date.

EDMS Doc. Number: [redacted]

4. STUDY POPULATION

4.1. Number of Subjects

This is a multicenter, open-label study in which 69 evaluable subjects were enrolled during Part A (dose escalation). During Part B (dose expansion), approximately 20-25 evaluable subjects may be enrolled in Cohort 1. In addition, Cohort 3 will enroll approximately 15 evaluable subjects at 30 mg CC-90010 3-days-on/11-days-off in each 28-day cycle. Enrollment occurred at 3 sites in Spain for Part A. Enrollment in Part B will include additional sites in Europe (approximately 8 sites in total). During Part C (Food Effect assessment), approximately 24 subjects who complete both treatment Periods 1 and 2 (completers) will be enrolled. Subjects may be replaced at the discretion of the sponsor to ensure 24 completers. Enrollment in Part C will occur in the same Spanish sites as in Part A.

4.2. Inclusion Criteria

Subjects must satisfy the criteria below to be enrolled in the study.

1. Men and women ≥ 18 years of age, at the time of signing the informed consent document (ICD).
2. Subject must understand and voluntarily sign an ICD prior to any study-specific assessments/procedures being conducted.
3. Subject is willing and able to adhere to the study visit schedule and other protocol requirements.
4. For subjects enrolling in food-effect assessment (Part C) only:
    a. Subject must agree and be willing to consume a standard high-fat, high-calorie meal.
    b. Subject must be willing to refrain from caffeine or xanthene-containing products (coffee, tea, cola, chocolate, etc.) for 48 hours prior to dosing on Cycle 1 Day 4 and Cycle 2 Day 4 and up to 24 hours post dose.
5. Subjects with histological or cytological confirmation of either:
    a. In Part A, advanced or unresectable solid tumors or advanced relapsed and/or refractory NHL (ie, DLBCL and FL or MZL) including those who have progressed on (or not been able to tolerate due to medical comorbidities or unacceptable toxicity) standard anticancer therapy or for whom no other approved conventional therapy exists.
    b. In Part B dose expansion,
    - Cohorts 1 and 3: relapsed and/or refractory DLBCL following at least 2 prior lines of therapy (e.g. have failed at least one line of standard therapy and have received at least one prior line of salvage therapy) OR have failed at least one prior line of standard therapy and are not eligible for autologous stem cell transplant (ASCT) or have declined ASCT; transformed lymphoma following chemotherapy for lower grade lymphoma and at least two standard treatment regimen for DLBCL.
        – Subjects with two or more lines of systemic therapy must have been treated with and have lack of response after chimeric antigen receptor (CAR) T-cell therapy, if

such therapy is available, OR be ineligible for CAR T-cell therapy at the time of enrollment, OR subject declined CAR T-cell therapy.

- Cohort 2: advanced basal cell carcinoma including those who have progressed on (or not been able to tolerate due to medical comorbidities or unacceptable toxicity) standard anticancer therapy or for whom no other approved conventional therapy exists.

c. In Part C, advanced or unresectable solid tumors including those who have progressed on (or not been able to tolerate due to medical comorbidities or unacceptable toxicity) standard anticancer therapy or for whom no other approved conventional therapy exists.

6. At least one site of measurable disease according to RECIST 1.1 (Eisenhauer, 2009) for subjects with solid tumors; bi-dimensionally measurable disease on cross sectional imaging by CT or MRI, with at least one lesion >1.5 cm in its greatest transverse diameter, as defined by the IWG criteria (Cheson, 2014) for subjects with NHL. For subjects with rare malignancies, not falling into the above categories and who might benefit from a treatment with BET inhibitor, evaluable disease can be considered.
7. Tumor biopsies whenever safe and feasible will be collected in Part A, except for subjects with GBM. Subject consents to mandatory tumor biopsies (Screening and on treatment) in Part B. In exceptional circumstances an exemption waiver may be granted by the Sponsor for this criterion.
8. ECOG PS of 0 to 1.
9. Subjects must have the following laboratory values at screening:
   - Absolute neutrophil count (ANC) ≥ 1.5 x 10^9/L without growth factor support for 7 days (14 days if subject received pegfilgrastim).
   - Hemoglobin (Hgb) ≥10 g/dL (≥ 8 g/dL for NHL subjects)
   - Platelet count (plt) ≥150 x 10^9/L (≥100 x 10^9/L without transfusion for 7 days for NHL subjects)
   - Serum potassium concentration within normal range, or correctable with supplements
   - Serum glutamic oxaloacetic transaminase (SGOT)/aspartate aminotransferase (AST) and serum glutamate pyruvic transaminase (SGPT)/alanine aminotransferase (ALT) ≤ 3.0 x Upper Limit of Normal (ULN) or ≤ 5.0 x ULN if liver metastases are present
   - Serum total bilirubin ≤ 1.5 x ULN or < 2 x ULN if liver metastases are present
   - Serum creatinine ≤ 1.5 x ULN or measured glomerular filtration rate (GFR) ≥ 50 mL/min/1.73 m^2 using an exogenous filtration marker such as iohexol, inulin, 51Cr EDTA or 1^{125} iothalamate, or creatinine clearance of ≥ 50 mL/min using Cockroft-Gault equation.
   - Subjects must have serum albumin > 3.5 g/dL
   - PT (or INR) and APTT within normal range

EDMS Doc. Number: [redacted]

10. Females of childbearing potential (FCBP)[1] must:
    - Either commit to true abstinence[2] from heterosexual contact (which must be reviewed on a monthly basis and source documented) or agree to use, and be able to comply with, at least two effective contraceptive methods (oral, injectable, or implantable hormonal contraceptive; tubal ligation; intra-uterine device; barrier contraceptive with spermicide; or vasectomized partner), one of which must be barrier, from signing the ICD, throughout the study (including dose interruptions), and for up to 46 days following the last dose of CC-90010; and
    - Have two negative pregnancy tests as verified by the Investigator prior to starting CC-90010:
        - a negative serum pregnancy test (sensitivity of at least 25 mIU/mL) at Screening
        - a negative serum or urine pregnancy test (Investigator's discretion) within 72 hours prior to Cycle 1 Day 1 of study treatment (note that the Screening serum pregnancy test can be used as the test prior to Cycle 1 Day 1 study treatment if it is performed within the prior 72 hours).
    - Avoid conceiving for 46 days after the last dose of CC-90010.
    - Agree to ongoing pregnancy testing during the course of the study, and after the end of study treatment. This applies even if the subject practices true abstinence[2] from heterosexual contact.
11. Males must practice true abstinence[2] (which must be reviewed on a monthly basis) or agree to use a condom (a latex condom is recommended) during sexual contact with a pregnant female or a FCBP and will avoid conceiving from signing the ICD, while participating in the study, during dose interruptions, and for at least 106 days following CC-90010 discontinuation, even if he has undergone a successful vasectomy.

4.3. Exclusion Criteria

The presence of any of the following will exclude a subject from enrollment:

1. Subject has received anti-cancer therapy (either approved or investigational) within ≤ 4 weeks or 5 half-lives, whichever is shorter, prior to starting CC-90010.
    - < 42 days for prior nitrosoureas or mitomycin C
2. Subject has received prior CAR T-cell therapy or other T-cell targeting treatment (approved or investigational) ≤ 4 weeks prior to starting CC-90010.

[1] A female of childbearing potential is a sexually mature woman who 1) has achieved menarche at some point 2) has not undergone a hysterectomy (the surgical removal of the uterus) or bilateral oophorectomy (the surgical removal of both ovaries) or 3) has not been naturally postmenopausal (amenorrhea following cancer therapy does not rule out childbearing potential) for at least 24 consecutive months (ie, has had menses at any time during the preceding 24 consecutive months).

[2] True abstinence is acceptable when this is in line with the preferred and usual lifestyle of the subject. [Periodic abstinence (eg, calendar, ovulation, symptothermal, post-ovulation methods) and withdrawal are not acceptable methods of contraception].

EDMS Doc. Number: [redacted]

3. Toxicities resulting from prior systemic cancer therapies must have resolved to ≤ NCI CTCAE Grade 1 prior to starting CC-90010 treatment (with exception of grade 2 peripheral neuropathy and alopecia).
4. Subject has received autologous hematologic stem cell transplant (HSCT) ≤ 3 months prior to starting CC-90010 treatment. Subjects with allogeneic HSCT will not be allowed on this protocol.
   - 6-month exclusionary period for recovery from HSCT-associated toxicity.
5. Subject has undergone major surgery ≤ 4 weeks or minor surgery ≤ 2 weeks prior to starting CC-90010 or who have not recovered from surgery.
6. Subject has completed any radiation treatment < 4 weeks prior to starting CC-90010 (with exception of palliative bone radiotherapy for which 2-week period is required).
7. Subject has persistent diarrhea due to a malabsorptive syndrome (such as celiac sprue or inflammatory bowel disease) ≥ NCI CTCAE Grade 2, despite medical management, or any other significant GI disorder that could affect the absorption of CC-90010.
8. Subjects with symptomatic or uncontrolled ulcers (gastric or duodenal), particularly those with a history of and/or risk of perforation and GI tract hemorrhages.
9. Symptomatic, untreated, or unstable central nervous system (CNS) metastases.
   - Subjects recently treated with whole brain radiation or stereotactic radiosurgery for CNS metastases must have completed therapy at least 4 weeks prior to starting CC-90010 and have a follow-up brain CT or MRI demonstrating either stable or improving metastases 4 or more weeks after completion of radiotherapy (the latter to be obtained as part of the Screening Assessments, refer to Section 6.1).
   - Subjects must be asymptomatic and off steroids or on stable dose of steroids for at least 4 weeks (<4 mg/day dexamethasone or equivalent) or on tapering dose of steroids.
10. Known symptomatic acute or chronic pancreatitis.
11. Impaired cardiac function or clinically significant cardiac diseases, including any of the following:
    - LVEF < 45% as determined by multiple gated acquisition scan (MUGA) or echocardiogram (ECHO).
    - Complete left bundle branch or bifascicular block.
    - Congenital long QT syndrome.
    - Persistent or clinically meaningful ventricular arrhythmias or atrial fibrillation.
    - QTcF ≥ 480 msec on Screening ECG (mean of triplicate recordings).
    - Unstable angina pectoris or myocardial infarction ≤ 6 months prior to starting CC-90010.
    - Other clinically significant heart disease such as congestive heart failure requiring treatment or uncontrolled hypertension (blood pressure ≥ 160/95 mm Hg).

12. Pregnant or nursing females.
13. Known HIV infection.
14. Known chronic active hepatitis B or C virus (HBV, HCV) infection.
    - Subjects who are seropositive due to HBV vaccination are eligible.
    - Subjects who have no active viral infection and are under adequate prophylactics against HBV re-activation are eligible.
    - Subjects with HCC are exempt from the above criteria.
15. Ongoing treatment with chronic, therapeutic dosing of anti-coagulants (eg, warfarin, low molecular weight heparin, Factor Xa inhibitors, thrombin antagonists). Low dose low molecular weight heparin for catheter maintenance and for prophylactics for subjects with prior PE and DVT are permitted.
16. History of concurrent second cancers requiring active, ongoing systemic treatment.
17. Subjects with a history of clinically significant cognitive disorder(s) or active cognitive disorder(s).
18. Evidence of history of bleeding diathesis. Any hemorrhage/bleeding event > CTCAE Grade 2 or haemoptysis > 1 teaspoon within 4 weeks prior to the first dose
19. Subjects with known prior episodes of non-arteritic anterior ischemic optic neuropathy (NAION) should be excluded from the study. CC-90010 should be used with caution in subjects with retinitis pigmentosa.
20. Subject has any significant medical condition (eg, active or uncontrolled infection or renal disease), laboratory abnormality, or psychiatric illness that would prevent the subject from participating (or compromise compliance) in the study or would place the subject at unacceptable risk if he/she were to participate in the study.
21. Subject has any condition that confounds the ability to interpret data from the study.
22. Patients with poor bone marrow reserve as assessed by the Investigator such as in the following conditions:
    - Having received extensive bone radiotherapy
    - Having experienced several episodes of bone marrow aplasia in previous treatments
    - Confirmed histological bone marrow cancer infiltration (with exemption of NHL)
    - Requiring regular hematopoietic support (blood transfusion, erythropoietin, GCSF)

5. TABLE OF EVENTS

For a detailed description of the procedures listed, please refer to Section 6. For details regarding CC-90010 administration, please refer to Section 7. The Table of Events for Part A is provided in Appendix B.

Table 6: Table of Events - Part B (Cohorts 1 and 2) and Part C

		Treatment Period																Follow-up Period[b]	
		Cycle 1								Cycles 2-4					Cycles 5+				
	Screening	WK1				WK2		WK3	WK4	WK1		WK2	WK3	WK4	WK1	WK3	EOT	Safety	Long Term
Events[a]	D-28 to -1	D1	D2	D3	D4/ D5	D8	D9	D15	D22	D1	D4	D8	D15	D22	D1	D15[c]	≤ 28 days	28 days (±3 days)	q3 mo (±2 wks)
**Study Entry (Section 6.1)**																			
Informed consent	X																		
Contraceptive counseling	X	X								X					X		X		
Informed consent for optional exploratory analyses/PK sampling	X																		
Inclusion/ exclusion criteria	X																		
Medical/ oncologic history and therapies	X																		
Demographics	X																		
IRT registration	X	X								X					X		X		
Prior/concomitant medications & procedures	Continuous starting from the time a subject sign the ICD until 28 days after the last dose of CC-90010																		
**Study Drug (Section 7)**																			
Administer oral CC-90010 at RP2D (45 mg, 4-days-on/24-days-off in each 28-day cycle) in Part B and Part C[d] (Section 7.2.1)		X	X	X	X (D4)					X	X				X				

EDMS Doc. Number: [redacted]

Table 6: Table of Events - Part B (Cohorts 1 and 2) and Part C (Continued)

Events[a]	Screening	Treatment Period																Follow-up Period[b]	
		Cycle 1								Cycles 2-4					Cycles 5+				
		WK1				WK2		WK3	WK4	WK1		WK2	WK 3	WK4	WK1	WK3	EOT	Safety	Long Term
	D-28 to -1	D1	D2	D3	D4/ D5	D8	D9	D15	D22	D1	D4	D8	D15	D22	D1	D15[c]	≤ 28 days	28 days (±3 days)	q3 mo (±2 wks)
Food Effect Assessment (Part C only)[e]					X (D4)						X (C2 only)								
Provide/review of diary card		X	X	X	X (D4)					X	X (C2 only part C)				X		X		
IP accountability		X								X					X		X		
**Safety Assessments (Section 6)**																			
Adverse Event Monitoring (Section 6.2.2)	Continuous starting from the time a subject sign the ICD until 28 days after the last dose of CC-90010																		
Height	X																		
Weight	X	X				X		X	X	X		X	X	X	X	X	X		
Vital Signs (Section 6.2.4)	X	X				X		X	X	X		X	X	X	X	X	X		
Physical Examination (Section 6.2.5)	X	X								X					X		X		
ECOG PS (Appendix H)	X	X								X					X		X		
B Symptoms Assessment (only NHL; Section 6.2.6)	X				As clinically indicated												X		
12-lead ECG (single or triplicate; Section 6.2.7)[f]	X	X			X (D4)					X					X		X		
LVEF (ECHO/MUGA; Section 6.2.8)	X	As clinically indicated															X (±7d)		

EDMS Doc. Number: [redacted]

Table 6: Table of Events - Part B (Cohorts 1 and 2) and Part C (Continued)

		Treatment Period																Follow-up Period[b]	
		Cycle 1								Cycles 2-4					Cycles 5+				
	Screening	WK1				WK2		WK3	WK4	WK1		WK2	WK 3	WK4	WK1	WK3	EOT	Safety	Long Term
**Events[a]**	**D-28 to -1**	**D1**	**D2**	**D3**	**D4/ D5**	**D8**	**D9**	**D15**	**D22**	**D1**	**D4**	**D8**	**D15**	**D22**	**D1**	**D15[c]**	**≤ 28 days**	**28 days (±3 days)**	**q3 mo (±2 wks)**
Pregnancy Testing (FCBP only; Section 6.2.9)	X	X								X					X		X		
Hematology laboratory (Section 6.2.10)	X (D-14 to -1)	X				X		X	X	X		X (C2 only)	X	X (C2 only)	X		X		
Chemistry laboratory with LDH & uric acid tests (Section 6.2.10)	X (D-14 to -1)	X				X		X	X	X		X (C2 only)	X	X (C2 only)	X		X		
PT, INR, APTT	X (D-14 to -1)	X				X		X	X	X		X (C2 only)	X	X (C2 only)	X		X		
Hemoglobin A1c	X									X					X		X		
Amylase, lipase Insulin, C-Peptide		X										X (C2 only)			X (q3 cycles, ie, C5, C8, etc)		X		
Urinalysis (Section 6.2.10)	X (D-14 to -1)	X								X					X		X		
**PK & PD Assessments (Sections 6.5 & 6.6)**																			
Blood, PK of CC-90010 **(Part B only)** (Section 6.5.2)		X	X	X	X	X	X (D9 and D11)				X[g] (C2 only)								

EDMS Doc. Number: [redacted]

Table 6: Table of Events - Part B (Cohorts 1 and 2) and Part C (Continued)

		Treatment Period																Follow-up Period[b]	
		Cycle 1								Cycles 2-4					Cycles 5+				
	Screening	WK1				WK2		WK3	WK4	WK1		WK2	WK3	WK4	WK1	WK3	EOT	Safety	Long Term
Events[a]	D-28 to -1	D1	D2	D3	D4/ D5	D8	D9	D15	D22	D1	D4	D8	D15	D22	D1	D15[c]	≤ 28 days	28 days (±3 days)	q3 mo (±2 wks)
Blood, PK of CC-90010[h] (Food effect assessment - **(Part C only)** (Section 6.5.3)		X	X	X	X	X	X (D9 and D11)	X	X		X (D4 and D5 C2 only)	X (D8, D9 and D11 C2 only)	X (C2 only)	X (C2 only)					
Blood (whole), PD (Part B only) (Section 6.6.2.2)	X	X	X	X	X	X	X				X[i] (C2 only)						X		
Tumor Biopsy (Part B only; mandatory)	X[j]										X[j] (C2 only)								
Archival tumor tissue (FFPE) – Part B only[k]	X																		
**Efficacy (Section 6.4)**																			
Solid tumor/NHL assessments: CT/MRI imaging[l]	X													X (D28 ±7d; C2 & C4)	X (D28 ± 7d in C6, then q3 cycles, ie, end of C9, C12, etc)		X		
NHL-specific: bone marrow evaluation if known or suspected bone marrow involvement	X[m]									X, only when confirming CR									
NHL-specific: FDG PET or PET/CT scan (not required if tumor is FDG-negative)	X									Required to document response assessment if FDG-avid at Screening									

EDMS Doc. Number: [redacted]

Table 6: Table of Events - Part B (Cohorts 1 and 2) and Part C (Continued)

		Treatment Period																Follow-up Period[b]	
		Cycle 1								Cycles 2-4					Cycles 5+				
	Screening	WK1				WK2		WK3	WK4	WK1		WK2	WK3	WK4	WK1	WK3	EOT	Safety	Long Term
Events[a]	D-28 to -1	D1	D2	D3	D4/ D5	D8	D9	D15	D22	D1	D4	D8	D15	D22	D1	D15[c]	≤ 28 days	28 days (±3 days)	q3 mo (±2 wks)
**Additional Follow-up (Section 6.3)**																			
AE/SAE follow-up																		X	
Survival follow-up																			X

Abbreviations: AE = adverse event; β - hCG = beta human chorionic gonadotropin; BMMC = bone marrow mononuclear cells; C = cycle; CK = creatine kinase; CT = computed tomography; D = day(s); ECHO = echocardiogram; ECOG = Eastern Cooperative Oncology Group; ECG = electrocardiogram; FCBP = females of child bearing potential; FDG = fluorodeoxyglucose; FFPE = formalin-fixed, paraffin embedded; INR = international normalized ratio; ICD = informed consent document; IRT = interactive response technology; LDH = lactic dehydrogenase; LVEF = left ventricular ejection fraction; mo = months; MRI = magnetic resonance imaging; MUGA = multi-gated acquisition scan; NHL= non-Hodgkin's lymphoma; PET = positron emission tomography; PD = pharmacodynamic; PK = pharmacokinetic; PS = performance score; PT = prothrombin time; PTH = parathyroid hormone; APTT = activated partial thromboplastin time; q = every; RP2D = recommended Phase 2 dose; SAE = serious adverse event; WK(s) = week.

[a] All study visits/procedures will have a ± 3 day window (screening +3 day window only) and all laboratory blood samples should be drawn predose, unless otherwise specified in this table or Section 6.

[b] This Safety follow-up assessment may be by telephone (refer to Section 6.3.1). Long Term survival follow-up for up to 2 years or until death, lost to follow-up, or End of Trial, whichever occurs first. May be conducted by record review (including public records) and/or telephone contact with the subject, family, or treating physician.

[c] For Cycles 7 and higher, subjects will only complete Day 1 assessments. No assessments or visit is required on Day 15 (Week 3) unless clinically indicated.

[d] The RP2D is 45 mg CC-90010 given once daily for 4 consecutive days on followed by 24 consecutive days off (4-days-on/24-days-off) in each 28-day cycle. Refer to Section 7.2.1 and Section 7.2.1.1.

[e] In Part C, on Day 4 of Period 1 and Period 2 (Cycle 1 and Cycle 2 respectively), subjects will receive their dose of CC-90010, either with or without food, depending on the treatment sequence to which subject is randomized.

[f] Screening triplicate ECGs must be performed prior to dosing to fulfill eligibility criteria. Triplicate ECGs will be performed in Cycle 1 Days 1 and 4 (refer to Section 6.2.7). A single ECG will be performed on Day 1 of subsequent cycles and at EOT.

[g] Please refer to Section 6.5.2 for the Part B PK schedule. In Part B, an additional time-matched blood PK sample should be collected during Cycle 2 Day 4 when the tumor biopsy is performed. The recommended time for the tumor biopsy is between 2 to 6 hours after administration of CC-90010 on Cycle 2 Day 4.

[h] Please refer to Section 6.5.3 for the PK assessments in Part C. Blood samples will be collected prior to dosing and for up to 18 days (432 hours) after Day 4 dose in each period to measure plasma concentrations of CC-90010.

[i] Please refer to Section 6.6.2.2 for the PD schedule in Part B. An additional time-matched blood PD sample should be collected during Cycle 2 Day 4 when the tumor biopsy is performed in Part B.

[j] Paired tumor biopsies are mandatory for Part B. Tumor samples will be split for PK and PD analyses. In exceptional circumstances an exemption waiver may be granted by the Sponsor for mandatory paired tumor biopsies in Part B (needs to be discussed with Sponsor for prior authorization). The Screening biopsy should be obtained after all inclusion/exclusion criteria have been fulfilled. In Part B, the on treatment biopsy will be obtained on Cycle 2 Day 4. The recommended time for the tumor biopsy is between 2 to 6 hours after administration of CC-90010 on Cycle 2 Day 4.
In Part C, paired tumor biopsies will not be collected.

[k] Archival tumor tissue collected in Part B only. Should be obtained by the end of Cycle 1. Archival tumor specimens are not required if a fresh tumor biopsy is obtained during Screening

[l] All subjects who discontinue treatment for reasons other than disease progression, start of a new anticancer therapy, or withdrawal of consent from the entire study will be followed according to the specified tumor assessment schedule until progression and/or initiation of new systemic anticancer therapies.

[m] May be omitted if results were normal on the subject's most recent historical bone marrow biopsy. Additionally, this analysis may be omitted if a prior analysis was performed within 90 days before Cycle 1 Day 1. Historical results will be recorded in the eCRF.

Table 7: Table of Events - Part B (Cohort 3)

Events[a]	Screening	Treatment Period																Follow-up Period[b]	
		Cycle 1								Cycles 2-4					Cycles 5+		EOT	Safety	Long Term
		WK1				WK2	WK3		WK4	WK1		WK2	WK3	WK4	WK1	WK3			
	D-28 to -1	D1	D2	D3	D4	D8	D15	D17	D22	D1	D3	D8	D15	D22	D1	D15[c]	≤ 28 days	28 days (±3 days)	q3 mo (±2 wks)
**Study Entry (Section 6.1)**																			
Informed consent	X																		
Contraceptive counseling	X	X								X					X		X		
Informed consent for optional exploratory analyses	X																		
Inclusion/ exclusion criteria	X																		
Medical/ oncologic history and therapies	X																		
Demographics	X																		
IRT registration	X	X								X					X		X		
Prior/concomitant medications & procedures	Continuous starting from the time a subject sign the ICD until 28 days after the last dose of CC-90010																		
**Study Drug (Section 7)**																			
Administer oral CC-90010 at 30 mg, 3-days-on/11-days-off in each 28-day cycle) in Part B cohort 3[d] (Section 7.2.1)		X	X	X			X	X		X	X		X		X	X			

Table 7: Table of Events - Part B (Cohort 3) (Continued)

Events[a]	Screening	Treatment Period																Follow-up Period[b]	
		Cycle 1								Cycles 2-4					Cycles 5+		EOT	Safety	Long Term
		WK1				WK2	WK3		WK4	WK1		WK2	WK3	WK4	WK1	WK3			
	D-28 to -1	D1	D2	D3	D4	D8	D15	D17	D22	D1	D3	D8	D15	D22	D1	D15[c]	≤ 28 days	28 days (±3 days)	q3 mo (±2 wks)
Provide/review of diary card		X	X	X			X	X (D16, D17)		X			X		X	X	X		
IP accountability		X								X					X		X		
**Safety Assessments (Section 6)**																			
Adverse Event Monitoring (Section 6.2.2)	Continuous starting from the time a subject sign the ICD until 28 days after the last dose of CC-90010																		
Height	X																		
Weight	X	X				X	X		X	X		X	X	X	X	X	X		
Vital Signs (Section 6.2.4)	X	X				X	X		X	X		X	X	X	X	X	X		
Physical Examination (Section 6.2.5)	X	X								X					X		X		
ECOG PS (Appendix H)	X	X								X					X		X		
B Symptoms Assessment (only NHL; Section 6.2.6)	X				As clinically indicated												X		
12-lead ECG (single or triplicate; Section 6.2.7)[e]	X	X						X		X					X		X		
LVEF (ECHO/MUGA; Section 6.2.8)	X	As clinically indicated															X (±7d)		
Pregnancy Testing (FCBP only; Section 6.2.9)	X	X								X					X		X		

Table 7: Table of Events - Part B (Cohort 3) (Continued)

		Treatment Period																Follow-up Period[b]	
		Cycle 1								Cycles 2-4					Cycles 5+				
	**Screening**	**WK1**				**WK2**	**WK3**		**WK4**	**WK1**		**WK2**	**WK3**	**WK4**	**WK1**	**WK3**	**EOT**	**Safety**	**Long Term**
**Events[a]**	**D-28 to -1**	**D1**	**D2**	**D3**	**D4**	**D8**	**D15**	**D17**	**D22**	**D1**	**D4**	**D8**	**D15**	**D22**	**D1**	**D15[c]**	**≤ 28 days**	**28 days (±3 days)**	**q3 mo (±2 wks)**
Hematology laboratory (Section 6.2.10)	X (D-14 to -1)	X				X	X		X	X		X (C2 only)	X	X (C2 only)	X		X		
Chemistry laboratory with LDH & uric acid tests (Section 6.2.10)	X (D-14 to -1)	X				X	X		X	X		X (C2 only)	X	X (C2 only)	X		X		
PT, INR, APTT	X (D-14 to -1)	X				X	X		X	X		X (C2 only)	X	X (C2 only)	X		X		
Hemoglobin A1c	X									X					X		X		
Amylase, lipase Insulin, C-Peptide		X										X (C2 only)			X (q3 cycles, ie, C5, C8, etc)		X		
Urinalysis (Section 6.2.10)	X (D-14 to -1)	X								X					X		X		
**PK & PD Assessments (Sections 6.5 & 6.6)**																			
Blood, PK of CC-90010 (Part B) (Section 6.5.2)		X	X	X	X	X		X (D17, D18, D19)	X (D22, D24)		X[f] (D3 C2 only)								

Table 7: Table of Events - Part B (Cohort 3) (Continued)

Events[a]	Screening	Treatment Period																Follow-up Period[b]	
		Cycle 1								Cycles 2-4					Cycles 5+		EOT	Safety	Long Term
		WK1				WK2	WK3		WK4	WK1		WK2	WK3	WK4	WK1	WK3			
	D-28 to -1	D1	D2	D3	D4	D8	D15	D17	D22	D1	D4	D8	D15	D22	D1	D15[c]	≤ 28 days	28 days (±3 days)	q3 mo (±2 wks)
Blood (whole), PD (Part B only) (Section 6.6.2.2)	X	X	X	X	X	X	X	X (D17, D18, D19)			X[g] (D3 C2 only)						X		
Tumor Biopsy (Part B only; mandatory)	X[h]										X[h] (D3 C2 only)								
Archival tumor tissue (FFPE) – Part B only[i]	X																		
**Efficacy (Section 6.4)**																			
NHL assessments: CT/MRI imaging[j]	X													X (D28 ±7d; C2 & C4)	X (D28 ± 7d in C6, then q3 cycles, ie, end of C9, C12, etc)		X		
NHL-specific: bone marrow evaluation if known or suspected bone marrow involvement	X[k]									X, only when confirming CR									
NHL-specific: FDG PET or PET/CT scan (not required if tumor is FDG-negative)	X									Required to document response assessment if FDG-avid at Screening									

Table 7: Table of Events - Part B (Cohort 3) (Continued)

		Treatment Period																Follow-up Period[b]	
		Cycle 1								Cycles 2-4					Cycles 5+				
	Screening	WK1				WK2	WK3		WK4	WK1		WK2	WK3	WK4	WK1	WK3	EOT	Safety	Long Term
Events[a]	D-28 to -1	D1	D2	D3	D4	D8	D15	D17	D22	D1	D4	D8	D15	D22	D1	D15[c]	≤ 28 days	28 days (±3 days)	q3 mo (±2 wks)
**Additional Follow-up (Section 6.3)**																			
AE/SAE follow-up																		X	
Survival follow-up																			X

Abbreviations: AE = adverse event; β - hCG = beta human chorionic gonadotropin; BMMC = bone marrow mononuclear cells; C = cycle; CK = creatine kinase; CT = computed tomography; D = day(s); ECHO = echocardiogram; ECOG = Eastern Cooperative Oncology Group; ECG = electrocardiogram; FCBP = females of child bearing potential; FDG = fluorodeoxyglucose; FFPE = formalin-fixed, paraffin embedded; ICD = informed consent document; INR = international normalized ratio; IRT = interactive response technology; LDH = lactic dehydrogenase; LVEF = left ventricular ejection fraction; mo = months; MRI = magnetic resonance imaging; MUGA = multi-gated acquisition scan; NHL= non-Hodgkin's lymphoma; PET = positron emission tomography; PD = pharmacodynamic; PK = pharmacokinetic; PS = performance score; PT = prothrombin time; PTH = parathyroid hormone; APTT = activated partial thromboplastin time; q = every; RP2D = recommended Phase 2 dose; SAE = serious adverse event; WK(s) = week.

[a] All study visits/procedures will have a ± 3 day window (screening +3 day window only) and all laboratory blood samples should be drawn predose, unless otherwise specified in this table or Section 6.

[b] This Safety follow-up assessment may be by telephone (refer to Section 6.3.1). Long Term survival follow-up for up to 2 years or until death, lost to follow-up, or End of Trial, whichever occurs first. May be conducted by record review (including public records) and/or telephone contact with the subject, family, or treating physician.

[c] For Cycles 7 and higher, subjects will only complete Day 1 assessments. No assessments or visit is required on Day 15 (Week 3) unless clinically indicated.

[d] The dose is 30 mg CC-90010 given once daily for 3 consecutive days on followed by 11 consecutive days off (3-days-on/11-days-off) in each 28-day cycle. Refer to Section 7.2.1 and Section 7.2.1.1.

[e] Screening triplicate ECGs must prior to dosing to fulfill eligibility criteria. Triplicate ECGs will be performed in Cycle 1 Days 1 and 17 (last dose) (refer to Section 6.2.7). A single ECG will be performed on Day 1 of subsequent cycles and at EOT.

[f] Please refer to Section 6.5.2 for the Part B PK schedule. In Part B, an additional time-matched blood PK sample should be collected when the tumor biopsy is performed. The recommended time for the tumor biopsy is between 2 to 6 hours after administration of CC-90010.

[g] Please refer to Section 6.6.2.2 for the PD schedule in Part B. An additional time-matched blood PD sample should be collected when the tumor biopsy is performed.

[h] Paired tumor biopsies are mandatory for Part B. Tumor samples will be split for PK and PD analyses. In exceptional circumstances an exemption waiver may be granted by the Sponsor for mandatory paired tumor biopsies in Part B (needs to be discussed with Sponsor for prior authorization). The Screening biopsy should be obtained after all inclusion/exclusion criteria have been fulfilled. The on treatment biopsy will be obtained on Cycle 2 Day 3. The recommended time for the tumor biopsy is between 2 to 6 hours after administration of CC-90010 on Cycle 2 Day 3.

[i] Should be obtained by the end of Cycle 1. Archival tumor specimens are not required if a fresh tumor biopsy is obtained during Screening

[j] All subjects who discontinue treatment for reasons other than disease progression, start of a new anticancer therapy, or withdrawal of consent from the entire study will be followed according to the specified tumor assessment schedule until progression and/or initiation of new systemic anticancer therapies.

[k] May be omitted if results were normal on the subject's most recent historical bone marrow biopsy. Additionally, this analysis may be omitted if a prior analysis was performed within 90 days before Cycle 1 Day 1. Historical results will be recorded in the eCRF.

6. PROCEDURES

Any questions regarding the protocol should be directed to the Celgene Medical Monitor or designee. The procedures conducted for each subject enrolled in Part B and Part C of the study are outlined in Table 6 and Table 7. The Table of Events for Part A is provided in Table 17, Appendix B.

All study visits will have a ± 3 day window unless otherwise specified below or in the Table of Events (refer to Table 6 and Table 7).

All laboratory blood samples should be drawn predose unless otherwise specified (eg, PK samples).

The study procedures should be recorded in the source document and the electronic case report forms (eCRF). In the event subjects fail Screening, minimal information will be documented on the eCRFs, per database instructions.

6.1. Screening Period

The Screening window starts 28 days (+3 days) prior to first dose of CC-90010 and all screening tests and procedures must be completed within this window. Refer to Table 6 and Table 7, this section, and Section 6.2 for detailed information on procedures performed and the schedule.

Waivers to the protocol will not be granted during the conduct of this trial, under any circumstances.

Safety laboratory analyses will be performed locally. Screening laboratory values must demonstrate subject eligibility, but may be repeated within the screening window, if necessary.

The ICD will be administered at the Screening visit to all subjects by qualified study staff. It must be signed and dated by the subject and the administering staff prior to the start of any other study-specific procedures and its completion documented in source documents and in the eCRF.

The following will be performed at Screening, after informed consent has been obtained:

- Inclusion and exclusion criteria will be assessed at Screening and recorded in the source documents and the eCRF.
- Medical, oncologic, and surgical history, and demographic data (including each subject's date of birth, sex, race, and ethnicity) will be collected during Screening as consistent with local regulations. Oncologic history will include a detailed history of the primary diagnosis and date, therapies, and responses.
- Information on prior and concomitant medications and procedures will be collected (refer to Section 6.2.1).
- Registration in the interactive response technology system (IRT).
- Adverse event monitoring (refer to Section 10).
- Height and weight measured.
- Vital signs assessed (refer to Section 6.2.4).

- Physical examination (source documented only) and ECOG performance status (refer to Appendix H and Section 6.2.5).
  - For subjects with NHL in Part A only, measurements of lymph nodes and documentation of any enlargement of the spleen and/or liver will be recorded in the source document and in the eCRF.
- The B symptom assessment (NHL Subjects only): B symptoms are fever (> 100.5°F or 38°C) for 2 or more weeks without other evidence of infection, night sweats for more than 1 month without evidence of infection, and weight loss greater than 10% within the prior 6 months.
- A 12-lead ECG in triplicate (refer to Section 6.2.7) will be performed prior to dosing to fulfill eligibility criteria.
- Left Ventricular Ejection Fraction (LVEF) assessment (refer to Section 6.2.8).
- Pregnancy testing (refer to Section 6.2.9) for all females of childbearing potential. Appropriate methods of contraception and potential risks of fetal exposure will be discussed with subjects during Screening. Double contraceptive methods (one of which must be a barrier method) for females of childbearing potential (eg, oral, injectable, or implantable hormonal contraceptive; intra-uterine device; barrier contraceptive with spermicide; or vasectomized partner) must be used from the time the ICD is signed, throughout the study (including dose interruptions), and for 46 days after the last dose of the CC-90010. A single contraceptive method for males (a condom) must be used from the time the ICD is signed, throughout the study (including dose interruptions), and for at least 106 days after the last dose of the CC-90010. These will be documented in source documents.
- Clinical laboratory tests (refer to Section 6.2.10) to be completed within the timeframe specified in Table 6 and Table 7.
- Efficacy/tumor assessments (refer to Section 6.4).

6.2. Treatment Period

Visits and assessments are shown in Table 6 and Table 7. Subjects completing 6 cycles of treatment and continuing on study drug are only required to have clinic visits/assessments performed on Day 1 (±3 days) of each subsequent cycle (Cycles 7 and higher) unless more frequent visits are clinically indicated.

6.2.1. Concomitant Medication and Procedures

All concomitant medications and procedures taken or conducted beginning when the subject signs the ICD, throughout the study, and until 28 days after the last dose of CC-90010 will be recorded in the source documents and eCRF.

6.2.2. Adverse Event Monitoring

Adverse events and serious adverse events (SAEs) will be recorded from the time a subject signs the ICD until 28 days after the last dose of CC-90010.

Subjects experiencing AEs will be monitored with relevant clinical assessments and laboratory tests, as determined by the Investigator. Every attempt will be made to document resolution dates for ongoing AEs. The AEs will be recorded on the AE page of the eCRF and in the subject's source documents. Photographs of skin rashes should be obtained whenever possible, anonymized, and stored appropriately for future retrieval.

6.2.3. Weight

The subject's weight will be recorded in the source document and eCRF at the visits listed in Table 6 and Table 7.

6.2.4. Vital Signs

Vital signs include body temperature, blood pressure, pulse rate, and respiration rate and will be recorded at Screening and during the study at various time points for safety monitoring as described in Table 6 and Table 7.

Recorded measurements will be captured in the source document and eCRF.

6.2.5. Physical Examination and ECOG Performance Status

Complete physical examination and Eastern Cooperative Oncology Group Performance Status (ECOG PS; refer to Appendix H) will be performed at the visits listed in Table 6 and Table 7 and results for both will be recorded in the source document. Results for the ECOG PS will also be collected on the eCRF.

Physical examination findings will be classified as either normal or abnormal. If abnormal, a description of the abnormality and clinical importance will be provided in the source documents. Clinically significant changes from baseline will be recorded in the AE section of the eCRF.

For subjects with NHL in Part A only, measurements of lymph nodes and documentation of any enlargement of the spleen and/or liver will be recorded in the source document and on the eCRF.

6.2.6. B Symptom Assessment (NHL Subjects Only)

For subjects with NHL, B symptom assessments will be performed at the visits listed in Table 6 and Table 7, and results recorded in the source documents and on the eCRF.

B symptoms are fever (> 100.5°F or 38°C) for 2 or more weeks without other evidence of infection, night sweats for more than 1 month without evidence of infection, and weight loss greater than 10% within the prior 6 months.

6.2.7. 12-Lead Electrocardiograms

Triplicate standard 12-lead electrocardiograms (ECGs) will be recorded at the visits listed in Table 6 and Table 7 in all subjects. The 12-lead ECG should be collected prior to any blood draws if both are scheduled for the same nominal time. The 12-lead ECGs (12-lead at 25 mm/sec reporting rhythm, ventricular rate, PR interval, QRS complex, QT interval, and QTc interval) will be performed after the subject has been in the supine position for at least 5 minutes.

Triplicate ECGs (3 recordings within 2 ± 1 minute intervals) will be performed at:

- Screening

- Cycle 1
  - Day 1: predose (within 30 minutes prior to dosing), 2 hours (± 10 minutes) and 4 hours (± 10 minutes) postdose
  - Last dose (based on assigned dosing schedule): predose (within 30 minutes prior to dosing) and 2 hours (± 10 minutes) postdose

A single ECG will be performed at

- Day 1 of subsequent cycles: predose (within 30 minutes prior to dosing)
- EOT

Investigators will make immediate clinical decisions based on their interpretation of the ECG results and provide their overall assessment of the ECG in the eCRF. Clinically significant changes from baseline will be recorded in the AE section of the eCRF.

The ECG outputs will also be uploaded to the central ECG laboratory for definitive analysis and interpretation.

6.2.8. Left Ventricular Ejection Fraction

Left ventricular ejection fraction (LVEF), (multiple gated acquisition scan [MUGA], or echocardiogram [ECHO]) will be conducted at Screening in all subjects. Follow-up assessments should be performed as clinically indicated and at the EOT visit (± 7 days) if not performed within the previous 8 weeks, as indicated in Table 6 and Table 7. Follow-up assessments should use the same procedure used at the screening assessment. A clinically significant reduction is defined as either a ≥ 20% absolute reduction in LVEF or drop to below 45% from baseline.

6.2.9. Pregnancy Testing and Contraceptive Counseling

A female of childbearing potential (FCBP) is defined as a sexually mature woman who has:

- Not undergone a hysterectomy or bilateral oophorectomy, and
- Not been naturally postmenopausal (amenorrhea following cancer therapy does not rule out childbearing potential) for at least 24 consecutive months (eg, has had menses at any time in the preceding 24 consecutive months).

The Investigator will classify a female subject as a FCBP according to this definition. Pregnancy testing is not required for non-FCBP subjects but justification must be recorded in the eCRF and the source document. Pregnancy testing will be conducted by the local laboratory.

For an FCBP, pregnancy testing will be conducted at the visits listed in Table 6 and Table 7:

- A serum pregnancy test with sensitivity of at least 25 mIU/mL is to be obtained at Screening and serum or urine pregnancy test (based on Investigator's discretion) within 72 hours prior to Cycle 1 Day 1 of study treatment. The subject may not receive CC-90010 until the Investigator has verified the two screening pregnancy tests to be negative (note that the Screening serum pregnancy test can be used as the test prior to Cycle 1 Day 1 study treatment if it is performed within the prior 72 hours).

- A serum or urine pregnancy test (based on Investigator's discretion and minimum test sensitivity [25 mIU/mL]) should be done within 72 hours prior to Day 1 of every cycle and at the end of treatment (EOT) visit. The subject may not receive CC-90010 until the Investigator has verified the pregnancy test to be negative.
- An FCBP must avoid activities that could lead to conception while receiving CC-90010 and for 46 days after the last dose of CC-90010. Practice of true abstinence from sexual activity will be monitored monthly and source documented.
- A male subject whose partner is an FCBP must avoid activities that could lead to conception while receiving CC-90010 and for at least 106 days after the last dose of CC-90010. Practice of true abstinence from sexual activity will be monitored monthly and source documented.

Results for pregnancy tests will be recorded in the source document and eCRF.

6.2.10. Clinical Laboratory Tests

The following laboratory assessments will be performed at the Screening visit and during the study at the time points described in Table 6 and Table 7. All samples should be drawn predose unless otherwise specified. Laboratory assessments will be recorded in the source document and eCRF and are the following:

- Hematology: complete blood counts (CBC) including hemoglobin, hematocrit, WBC count with absolute differential and platelet count.
- Serum chemistry: albumin, total protein, magnesium, phosphorus, calcium, creatinine, urea/BUN, glucose (fasting ≥ 4 hours), potassium, sodium, chloride, total bilirubin (fractionate if outside normal range), alkaline phosphatase, AST or serum glutamic oxaloacetic transaminase (SGOT), ALT or serum glutamate pyruvic transaminase (SGPT), LDH, and uric acid.
- Special chemistry: hemoglobin A1c, insulin, C-peptide, amylase, lipase.
- Coagulation: PT, INR, and APTT
- Urinalysis: dipstick
  - Microscopy in the event of first appearance of 2+ or greater protein or worsening proteinuria.
- Glomerular filtration rate or creatinine clearance determination at Screening, if applicable, as specified in the inclusion criteria (refer to Section 4.2).

6.2.11. End of Treatment (EOT)

An EOT evaluation (refer to Table 6 and Table 7 for procedures) will be performed for subjects who are withdrawn from treatment for any reason as soon as possible (≤ 28 days) after the decision to permanently discontinue treatment has been made.

6.3. Follow-up Period

6.3.1. Safety Follow-up

All subjects will be followed for 28 days after the last dose of CC-90010 for AE reporting and concomitant medication information. The 28-day (±3 days) safety follow-up contact may be by telephone. In addition, any SAEs made known to the Investigator at any time thereafter that are suspected of being related to CC-90010 will be reported as described in Section 10.1.

6.3.2. Survival Follow-up

After the Safety Follow-up visit, all subjects will be followed every subsequent 3 months (± 2 weeks) for survival follow-up for up to 2 years or until death, lost to follow-up, or the End of Trial, whichever occurs first.

Survival follow-up may be conducted by record review (including public records) and/or telephone contact with the subject, family, or the subject's treating physician.

6.4. Efficacy Assessment

Tumor assessments will be performed at Screening and will include CTs of the chest, abdomen and pelvis, and a brain scan (CT or MRI) for subjects with known or suspected cerebral involvement. Bone scans will be done at screening if there is suspicion of bone metastases or known pre-existing bone metastases. Bone scans will only be repeated during study if needed to confirm PD or CR. Otherwise, bone scans can be spared. After Screening, radiologic tumor assessments will be performed at the end (Day 28 ± 7 days) of Cycles 2, 4, and 6, and then every 3 cycles thereafter, using the same CT/MRI scanning modalities used at Screening. If clinically indicated, tumor assessments may be done outside of these scheduled assessments. An EOT scan does not need to be obtained if the prior scan was within 28 days.

- Additionally for NHL subjects, at Screening [^{18}F]fluorodeoxyglucose (FDG) positron emission tomography (PET) or FDG PET/computed tomography CT scan is required in subjects with FDG-avid tumors. FDG PET or PET/CT imaging for tumor assessments after Screening are required to document response assessment if FDG-avid at Screening. An EOT scan does not need to be obtained if the prior scan was within 28 days.
- For NHL subjects with known or suspected bone marrow involvement, a bone marrow evaluation with flow immunophenotyping will be performed at Screening, and, to confirm a complete response (CR). The Screening evaluation may be omitted if results were normal on the subject's most recent historical bone marrow biopsy. Additionally, this analysis at Screening may be omitted if a prior analysis was performed within 90 days before Cycle 1 Day 1. Historical results will be recorded in the eCRF.

All subjects who discontinue treatment for reasons other than disease progression, start of a new anticancer therapy, or withdrawal of consent from the entire study will be followed according to the specified tumor assessment schedule until progression and/or initiation of new systemic anticancer therapies.

Tumor response at each post-screening assessment will be determined by the Investigator, based on Response Evaluation Criteria in Solid Tumors (RECIST) v1.1 as described in Appendix E for solid tumors and the IWG Response Criteria (Cheson, 2014) and the Deauville Criteria for fluorodeoxyglucose-positron emission tomography (FDG PET) scan interpretation (Itti, 2013; Meignan, 2014) as described in Appendix F for NHL. For subjects with glioblastoma multiforme, assessment will be based on the Response Assessment in Neuro-Oncology criteria (Wen, 2010). For locally advanced basal cell carcinoma (LaBCC) subjects, a conglomeration of radiology of target lesions assessed by RECIST 1.1, digital clinical photography assessed by WHO (bi-dimensional assessment) (Miller, 1981) and punch biopsies to confirm CR or if response confounded by lesion ulceration, cyst, or scarring/fibrosis will be used.

6.5. Pharmacokinetics

6.5.1. CC-90010 Pharmacokinetic Assessments- Part A

For evaluation of PK of CC-90010 in plasma, blood samples were collected from all subjects at the timepoints listed in Table 18 for the 3-days-on/4-days-off every week dosing schedule, Table 19 for the following schedules: 3-days-on/4-days-off every other week and 2-days-on/5-days-off each week, and Table 20 for the 4-days-on/24-days off dosing schedule in Appendix C. The actual time of each sample collection will be recorded in the source documents and on the electronic case report forms (eCRFs).

6.5.2. CC-90010 Pharmacokinetic Assessments- Part B

For evaluation of PK of CC-90010 in plasma, blood samples will be collected from all subjects at the timepoints listed in Table 8 and Table 9. The actual time of each sample collection will be recorded in the source documents and on the electronic case report forms (eCRFs).

Table 8: Pharmacokinetic Blood Sampling Schedule for the 4-days-on/24-days-off Schedule, Part B

Time in Hours Relative to CC-90010 Dose	Collection Window	Cycle 1 Day 1	Cycle 1 Day 4	Other Timepoints
0	Within 30 min prior to dosing	X	X	
0.5	± 5 min	X	X	
1	± 5 min	X	X	
1.5	± 5 min	X	X	
2	± 5 min	X	X	

EDMS Doc. Number: [redacted]

Table 8: Pharmacokinetic Blood Sampling Schedule for the 4-days-on/24-days-off Schedule, Part B (Continued)

Time in Hours Relative to CC-90010 Dose	Collection Window	Cycle 1 Day 1	Cycle 1 Day 4	Other Timepoints
3	± 10 min	X	X	
4	± 10 min	X	X	
24	± 1 hour	X (prior to dosing on Day 2)	X (Day 5)	
48	± 2 hours	X (prior to dosing on Day 3)		
96	± 2 hours		X (Day 8)	
120	± 2 hours		X (Day 9)	
168	± 2 hours		X (Day 11)	
At tumor biopsy on treatment				X[a] (Cycle 2 Day 4)

[a] An additional time-matched blood PK sample should be collected during Cycle 2 Day 4 when the tumor biopsy is performed. The recommended time for the tumor biopsy is between 2 to 6 hours after administration of CC-90010 on Cycle 2 Day 4.

Table 9: Pharmacokinetic Blood Sampling Schedule for the 3-days-on/11-days-off Schedule, Part B

Time in Hours Relative to CC-90010 Dose	Collection Window	Cycle 1 Day 1	Cycle 1 Day 17	Other Timepoints
0	Within 30 min prior to dosing	X	X	
0.5	± 5 min	X	X	
1	± 5 min	X	X	
1.5	± 5 min	X	X	
2	± 5 min	X	X	
3	± 10 min	X	X	
4	± 10 min	X	X	
24	± 1 hour	X (prior to dosing on Day 2)	X (Day 18)	
48	± 2 hours	X (prior to dosing on Day 3)	X (Day 19)	
72	± 2 hours	X (Day 4)		

Table 9: Pharmacokinetic Blood Sampling Schedule for the 3-days-on/11-days-off Schedule, Part B (Continued)

Time in Hours Relative to CC-90010 Dose	Collection Window	Cycle 1 Day 1	Cycle 1 Day 17	Other Timepoints
120	± 2 hours		X (Day 22)	
168	± 2 hours	X (Day 8)	X (Day 24)	
At tumor biopsy on treatment				X[a] (Cycle 2 Day 3)

[a] An additional time-matched blood PK sample should be collected during Cycle 2 Day 3 when the tumor biopsy is performed. The recommended time for the tumor biopsy is between 2 to 6 hours after administration of CC-90010 on Cycle 2 Day 3.

The Sponsor may collect additional unscheduled PK samples in order to follow up the safety of the study treatment or to better understand the progression of the disease or the disease's response to the study treatment.

See the Laboratory Manual for sample collection, handling, and processing instructions.

6.5.2.1. Tumor Tissue for PK Assessment in Part B

An analysis of CC-90010 concentrations in tumor tissue will be performed for all subjects in Part B. The tumor biopsy should be performed between 2 to 6 hours after administration of CC-90010 on Cycle 2 Day 4 for 4-days-on/24-days-off Schedule or Cycle 2 Day 3 for 3-days-on/11-days-off Schedule. Please refer to Section 6.6.3 for tumor biopsies.

6.5.3. CC-90010 Pharmacokinetic Assessments- Part C Food Effect Assessment

For evaluation of PK of CC-90010 in plasma during the food effect evaluation, blood samples will be collected from all subjects at the timepoints listed in. The actual time of each sample collection will be recorded in the source documents and on the electronic case report forms (eCRFs).

Table 10: Pharmacokinetic Blood Sampling Schedule for the 4-days-on/24-days-off Schedule for Food Effect Assessment, Part C

Time in Hours Relative to Most Recent CC-90010 Dose	Collection Window	Cycle 1 Day 1	Cycle 1 Day 4	Cycle 2 Day 4
0	Within 30 min prior to dosing	X	X	X
0.5	± 5 min	X		
1	± 5 min	X	X	X

Table 10: Pharmacokinetic Blood Sampling Schedule for the 4-days-on/24-days-off Schedule for Food Effect Assessment, Part C (Continued)

Time in Hours Relative to Most Recent CC-90010 Dose	Collection Window	Cycle 1 Day 1	Cycle 1 Day 4	Cycle 2 Day 4
1.5	± 5 min	X	X	X
2	± 5 min	X	X	X
3	± 10 min	X	X	X
4	± 10 min	X	X	X
6	± 10 min		X	X
24	± 1 hour	X (Day 2)	X (Day 5)	X (Day 5)
48	± 2 hours	X (Day 3)		
96	± 2 hours		X (Day 8)	X (Day 8)
120	± 2 hours		X (Day 9)	X (Day 9)
168	± 2 hours		X (Day 11)	X (Day 11)
264	± 2 hours		X (Day 15)	X (Day 15)
432	± 2 hours		X (Day 22)	X (Day 22)

Note: Days are relative to the first day of dosing (Day) in Cycle 1 or Cycle 2.

6.6. Biomarkers, Pharmacodynamics, Pharmacogenomics

6.6.1. Archival Tumor Biopsy

Archival tumor, as formalin-fixed, paraffin-embedded (FFPE) blocks or mounted sections will be retrieved for subjects in Part A and Part B, after eligible subjects are enrolled in the IRT system unless single-case exemption is granted by the Sponsor.

Archival tumor specimens are not required if a fresh tumor biopsy is obtained during Screening.

6.6.2. Pharmacodynamic and Predictive Biomarkers- Part A and Part B only

No pharmacodynamic biomarkers in blood will be collected for Part C (food effect assessment).

6.6.2.1. Whole Blood for PD Biomarker Studies- Part A

The schedules for pharmacodynamic biomarkers in blood for Part A are provided in Appendix D.

6.6.2.2. Whole Blood for PD Biomarker Studies- Part B

The schedules for pharmacodynamic biomarkers in blood for Part B are provided below in Table 11 and Table 12:

EDMS Doc. Number: [redacted]

Table 11: Pharmacodynamic Blood Sampling Schedule for the 4-days-on/24-days-off Schedule, Part B

Time in Hours Relative to CC-90010 Dose	Collection Window	Cycle 1 Day 1	Cycle 1 Day 4	Other Timepoints
Screening period	Day -28 to Day -1 (+3 days)			X
0	Within 30 min prior to dosing	X	X	
2	± 5 min	X	X	
4	± 10 min	X	X	
24	± 1 hours	X (prior to dosing on Day 2)	X (Day 5)	
48	± 2 hours	X (prior to dosing on Day 3)		
96	± 2 hours		X (Day 8)	
120	± 2 hours		X (Day 9)	
End of treatment				X
At tumor biopsy on treatment				X[a] (Cycle 2 Day 4)

[a] An additional time-matched PD sample should be collected during Cycle 2 Day 4 when the tumor biopsy is performed. The recommended time for the tumor biopsy is between 2 to 6 hours after administration of CC-90010 on Cycle 2 Day 4.

Table 12: Pharmacodynamic Blood Sampling Schedule for the 3-days-on/11-days-off Schedule, Part B

Time in Hours Relative to CC-90010 Dose	Collection Window	Cycle 1 Day 1	Cycle 1 Day 17	Other Timepoints
Screening Period	Day -28 to Day -1 (+3 days)			X
0	Within 30 min prior to dosing	X	X	
2	± 5 min	X	X	
4	± 10 min	X	X	
24	± 1 hour	X (prior to dosing on Day 2)	X (Day 18)	

Table 12: Pharmacodynamic Blood Sampling Schedule for the 3-days-on/11-days-off Schedule, Part B (Continued)

Time in Hours Relative to CC-90010 Dose	Collection Window	Cycle 1 Day 1	Cycle 1 Day 17	Other Timepoints
48	± 2 hours	X (prior to dosing on Day 3)	X (Day 19)	
72	± 2 hours	X (Day 4)		
120	± 2 hours		X (Day 22)	
168	± 2 hours	X (Day 8)		
336	± 2 hours	X (Day 15)		
End of treatment				X
At tumor biopsy on treatment				X[a] (Cycle 2 Day 3)

[a] An additional time-matched PD sample should be collected during Cycle 2 Day 3 when the tumor biopsy is performed. The recommended time for the tumor biopsy is between 2 to 6 hours after administration of CC-90010 on Cycle 2 Day 3.

The Sponsor may collect additional unscheduled PD samples in order to follow up the safety of the study treatment or to better understand the progression of the disease or the disease's response to the study treatment.

6.6.2.3. Tumor Tissue for PD and Predictive Biomarker Studies

Please refer to Section 6.6.3 for tumor biopsies. Paired tumor biopsies are mandatory in Part B.

- Screening (after all inclusion and exclusion criteria are fulfilled)
- Cycle 2 Day 4 for 4-days-on/24-days-off Schedule (Cohort 1) or Cycle 2 Day 3 for 3-days-on/11-days-off Schedule (Cohort 3)
- Optional, any other time until EOT visit.

6.6.3. Tumor Biopsies

Tumor biopsies will be collected whenever safe and feasible in Part A. Paired tumor biopsies are mandatory in Part B. Tumor tissue samples will be used for PK and PD analyses. No tumor biopsies will be collected for Part C (food effect assessment). In exceptional circumstances an exemption waiver may be granted by the Sponsor for mandatory paired tumor biopsies, but this needs to be discussed with the Sponsor for prior authorization. The biopsy is collected by either tumor excision (preferred) or core needle (at least 3 passages if possible) at Screening and in Cycle 1 on Day 16 or 17 in Part A, and at Screening and Cycle 2 Day 4 for 4-days-on/24-days-off Schedule (Cohort 1) and Cycle 2 Day 3 for 3-days-on/11-days-off Schedule (Cohort 3) in Part B. The recommended time for the tumor biopsy in Part B is between 2 to 6 hours after administration of CC-90010. For subjects with locally advanced or metastatic basal cell

carcinoma, a skin punch biopsy will be the most common form of tumor biopsy. An archival tumor sample must be provided if a fresh biopsy is not collected during Screening in Part A. Fine needle aspiration is not sufficient as a source of tumor biopsy material. Samples should be processed per Laboratory Manual. Optimally, the tumor tissue samples (Screening and on treatment) will be obtained from the same tumor site.

If CC-90010 has been interrupted prior to completing the Cycle 1 Day 16 or 17 dose in Part A, it is recommended that the tumor biopsy be deferred until after at least two consecutive doses have been administered. In Part B, if CC-90010 has been interrupted prior to completing the Cycle 2 Day 4 dose for 4-days-on/24-days-off Schedule (Cohort 1), it is recommended that the tumor biopsy be deferred until after four consecutive doses have been administered. If CC-90010 has been interrupted prior to completing the Cycle 2 Day 3 dose for 3-days-on/11-days-off Schedule (Cohort 3), it is recommended that the tumor biopsy be deferred until after three consecutive doses have been administered.

Additionally, an optional tumor biopsy may be obtained in both Part A and Part B, during later treatment cycles to elucidate effects of long-term treatment or resistance mechanisms, respectively.

See the Laboratory Manual for sample collection, handling, and processing instruction.

7. DESCRIPTION OF STUDY TREATMENTS

7.1. Description of Investigational Products (IP)

7.1.1. CC-90010

CC-90010 is a non-ionizable compound with a molecular weight of 383.5 g/mole. It is a white to pale yellow solid powder.

Celgene Corporation will supply CC-90010 clinical drug product as formulated tablets in high density polyethylene (HDPE) bottles with child-resistant caps, labeled appropriately for investigational use as per the regulations of the relevant country health authorities. The formulated tablets are white to off-white uncoated tablets at dosage strengths of 10 mg and 15 mg of CC-90010.

CC-90010 clinical drug product should be stored as indicated on the package label.

7.2. Treatment Administration and Schedule

7.2.1. CC-90010 Administration

CC-90010 will be administered once daily in the morning on an empty stomach (ie, ≥ 1 hour before breakfast) with at least 240 mL of water after an overnight fast lasting ≥ 4 hours in both Parts A and B. Subjects should abstain from food or other medication intake for ≥ 1 hour after each dose. In the first cohort in Part A, subjects will administer CC-90010 starting on Day 1 for 3 consecutive days followed by 4 consecutive days off drug every week (3/7-days schedule) in each 28-day cycle. Alternate dosing schedules may be implemented based on the review of clinical safety and laboratory data by the SRC.

The SRC determined the RP2D for Part B to be 45 mg CC-90010 given once daily for 4 consecutive days on followed by 24 consecutive days off (4-days-on/24-days-off) in each 28-day cycle. An alternative dosing schedule 30 mg CC-90010 3-days-on/11-days-off in each 28-day cycle will also be tested in Part B.

On study days that require PK assessments (refer to Section 6.5), CC-90010 will be administered in the clinic after any predose assessments are completed. On all other study days, subjects will self-administer their assigned doses at home and record dosing times on the study diary card (refer to Section 7.6).

Study treatment may be discontinued if there is evidence of clinically significant disease progression, unacceptable toxicity or subject/physician decision to withdraw (refer to Section 11). Subjects may continue to receive study drug beyond disease progression at the discretion of the Investigator in consultation with the Sponsor's study physician.

7.2.1.1. Missed Doses of CC-90010

All efforts should be made to administer study treatment on all the scheduled days of each treatment cycle. Any missed doses during that period should not be taken after the last scheduled day of administration but should be returned by the subject for IP accountability.

EDMS Doc. Number: [redacted]

If a subject vomits within 2 hours of taking a dose of CC-90010 and the whole tablet is visible in the vomit, the subject can take a replacement dose within 1 hour of vomiting. However, if the subject vomits more than 2 hours after taking a dose of CC-90010, or the whole tablet is not visible in the vomit, the subject should not take a replacement dose.

If a subject should miss or forget to take the scheduled dose of CC-90010, the subject can take the dose if it has not been more than 6 hours after the scheduled dose and still in fasting mode. If it has been more than 6 hours since the scheduled dose, the dose is skipped and is resumed at the next planned administration date.

7.2.1.2. CC-90010 Administration for Food Effect Study – Part C

Approximately 24 subjects with advanced solid tumors will be assigned randomly to 1 of 2 treatment sequences (see Table 13). The sequences will dictate the order in which each subject receives the following treatments:

- Treatment A: Single oral dose of CC-90010 (45 mg) at Hour 0 on Day 4, following a 10 hour overnight fast.
- Treatment B: Single oral dose of CC-90010 (45 mg) at Hour 0 on Day 4, 30 minutes after the start of a high-fat, high-calorie breakfast.

On Day 4 of each study period, subjects will receive their dose of CC-90010, either with or without food, depending on the treatment sequence to which the subject is randomized.

Table 13: Treatment Sequences for Food Effect Assessment

Overall Sample Size (N = 24)	Treatment Period 1	Washout (24 days)	Treatment Period 2
Sequence 1 (n = 12)	Treatment A		Treatment B
Sequence 2 (n = 12)	Treatment B		Treatment A

N = overall sample size; n = number of subjects randomized to each sequence.
Treatment A: Single oral dose of CC-90010 (45 mg) at Hour 0 on Day 4, following a 10 hour overnight fast.
Treatment B: Single oral dose of CC-90010 (45 mg) at Hour 0 on Day 4, 30 minutes after the start of a high-fat high-calorie breakfast.
Treatment Period 1 and the Washout correspond to Cycle 1. Treatment period 2 correspond to the initial 4 days of Cycle 2

On Day 1 to Day 3 of each period (Cycle 1 and Cycle 2) of the food effect substudy, all subjects will receive 45 mg CC-90010 dose in the morning on an empty stomach (ie, ≥ 1 hour before breakfast) with at least 240 mL of water after an overnight fast lasting ≥ 4 hours. Subjects should abstain from food or other medication intake for ≥ 1 hour after each dose.

Subjects must refrain from caffeine or xanthene-containing products (coffee, tea, cola, chocolate, etc.) for 48 hours prior to dosing on Cycle 1 Day 4 and Cycle 2 Day 4 and up to 24 hours post dose.

For evaluation of food effect on Day 4, all subjects must observe an overnight fast of at least 10 hours prior to Day 4 dosing. Water is allowed as necessary, except from between 1 hour prior to dosing and 1 hour following dosing (excluding any water given with CC-90010).

For the fed treatment (Treatment B in Part C), following the 10-hour overnight fast on Day 4, subjects will begin consuming a breakfast meal 30 ±5 minutes prior to the planned administration of CC-90010. Subjects are required to consume the entire meal within 30 minutes (no less than 20 minutes) from the time the meal is served. The time and date of CC-90010 dose, along with the start and end time of meal consumption, as well as the content of the consumed meal will be recorded. A high-fat (approximately 50 percent of total caloric content of the meal) and high-calorie (approximately 800 to 1000 calories) meal is to be consumed. This test meal should derive approximately 150, 250, and 500-600 calories from protein, carbohydrate, and fat, respectively.[3] The site will utilize appropriate nutritional staff, where available, in design of the meals. The meals will be provided by the site under direction of staff nutritionist or dietician in accordance with the protocol guidelines and any subject specific dietary needs/restrictions. Subjects will then ingest a 45 mg dose of CC-90010 with 240 mL of room temperature water. No additional food will be allowed for at least 4 hours post-dose. Subject may drink water as desired, except for 1 hour before and 1 hour after administration of CC-90010.

For the fasted treatment (Treatment A), following the 10-hour overnight fast, on Day 4, subjects will ingest a 45 mg dose of CC-90010 with 240 mL of room temperature water. No additional food will be allowed for at least 4 hours post-dose. Subject may drink water as desired, except for 1 hour before and 1 hour after administration of CC-90010.

On Days 1 to 4 of subsequent cycles starting with Cycle 3, 45 mg CC-90010 will be administered once daily in the morning on an empty stomach (ie, ≥ 1 hour before breakfast) with at least 240 mL of water after an overnight fast lasting ≥ 4 hours. Subjects should abstain from food or other medication intake for ≥ 1 hour after each dose. Subjects will take 45 mg of CC-90010 once daily for 4 consecutive days on followed by 24 consecutive days off (4-days-on/24-days-off) in each 28-day cycle.

7.2.2. Dose Escalation Process

For the purposes of dose escalation decisions, at least 3 subjects will be enrolled in successive cohorts. In Part A, the first cohort will be treated with the starting dose of 15 mg. Subjects must complete a minimum of 1 cycle of treatment with the minimum safety evaluation and drug exposure or have had a DLT within the first cycle of treatment to be considered evaluable for dose escalation decisions. The recommended dose for the next cohort of subjects will be guided by the BLRM with EWOC principle. Dose escalation decisions will occur when the cohort of subjects has met these criteria. Dose escalation decisions will be made by the SRC. Decisions will be based on a synthesis of all relevant data available from all dose levels evaluated in the ongoing study including safety information, DLTs, all treatment related CTCAE grade ≥ 2 toxicity data during Cycle 1, and PK, data from evaluable subjects. PK data from subjects will be made available on an on-going basis throughout the study and dosing will be adapted accordingly.

The adaptive Bayesian methodology provides an estimate of the dose levels of CC-90010 that do not exceed the MTD and incorporates all DLT information at all dose levels for this estimation.

[3] An example test meal would be two eggs fried in butter, two strips of bacon, two slices of toast with butter, four ounces of hash brown potatoes and eight ounces of whole milk. Substitutions in this test meal can be made as long as the meal provides a similar amount of calories from protein, carbohydrate, and fat and has comparable meal volume and viscosity.

In general, the next recommended dose will have the highest chance that the DLT rate will fall in the target interval (the true DLT rate lying in 16-33%) and will always satisfy the EWOC principle. Per EWOC it should be unlikely (<25% posterior probability) that the DLT rate at the next dose will exceed 0.33. In all cases, the recommended dose for the next cohort will not exceed a 100% increase from the previous dose. Smaller increases in dose may be recommended by the SRC upon consideration of all of the available clinical data.

The procedure for subject accrual in each dose cohort and provisions for dose escalation/de-escalation decisions for the study is as follows:

1. This study will begin by evaluating CC-90010 in Part A in cohorts of at least 3 evaluable subjects at each dose level. Initially, the dosing increments between cohorts will be 100%. When a single subject experiences a DLT or 2 subjects experience grade ≥ 2 treatment-related toxicity, the cohort size may be increased to 6 evaluable subjects for the current and subsequent cohorts. The increase in CC-90010 dose will be ≤ 50% for each subsequent dose escalation cohort.
2. Following completion of Cycle 1 for all evaluable subjects in a cohort, the two-parameter BLRM with EWOC principle will be used to make recommendations to the SRC for the next dose level with the following exceptions:
   - If the first 2 subjects in a cohort experience DLTs, no additional subjects will be enrolled into that cohort until the Bayesian model has been updated with this new information. Likewise, the model will be re-evaluated if 2 subjects in a cohort experience DLTs before the enrollment of any additional subject.
3. After each cohort, the SRC will meet and review data from the BLRM assessment and available safety (ie, DLT and non-DLT data), PK, PD, and preliminary efficacy information. The final dose escalation decisions will be made by the SRC.

After repeating the above steps, a CC-90010 dose can be declared the MTD after meeting the following conditions:

- at least 6 evaluable subjects have been treated at the dose,
- the posterior probability of targeted toxicity (eg, the true DLT rate lying in [0.16, 0.33]) at the dose exceeds 60% or a minimum of 6 subjects have been treated on the study, and
- the dose is recommended according to the BLRM and the SRC approves it.

At the discretion of the SRC to better understand the safety, tolerability and PK of CC-90010, additional cohorts of subjects may be enrolled at prior dose levels or to intermediate dose levels before or while proceeding with further dose escalation.

7.2.3. Dose Escalation Decisions

Provisional dose levels to be assigned to separate cohorts of subjects are described in Section 9.9.1. Dose decisions during escalation are however not limited to these doses. Based on the recommendation of the BLRM regarding the highest dose that may not be exceeded at any decision point during escalation and the maximum increase in dose allowed by the protocol, intermediate doses may be administered to subsequent new cohorts of subjects.

EDMS Doc. Number: [redacted]

The decision to evaluate additional subjects within a dose cohort, a higher dose cohort, intermediate dose cohorts, smaller dose increments, alternate dosing schedules, or declare an MTD will also be determined by the SRC, based on their review of clinical and laboratory safety data.

7.2.4. Definition of a Subject Evaluable for DLT

All subjects who receive at least one dose of CC-90010 will be evaluable for safety.

After the first dose is administered in any cohort during dose escalation, subjects in each cohort are observed for 28 days (Cycle 1, DLT window) before the next dose cohort can begin. No more than one subject per day will be enrolled in a given dose escalation cohort.

A subject evaluable for DLT is defined as one that:

- Has received ≥ 80% of the total planned dose amount of CC-90010 during Cycle 1 without experiencing a DLT,

or

- Experienced a DLT after receiving at least one dose of CC-90010.

Subjects non-evaluable for DLT will be replaced. Additional subjects within any dose cohort may be enrolled at the discretion of the SRC. Intra-subject dose escalation will not be allowed during the DLT assessment period.

7.2.5. Definition of Maximum Tolerated Dose (MTD)

The MTD is the highest dose that causes DLTs in not more than 33% of the subjects treated with CC-90010 in the first cycle with at least 6 evaluable subjects treated at this dose. The estimation of MTD is described in Section 7.2.2.

A variable dose cohort (eg, less frequent dosing) may be evaluated to accurately determine the MTD at the discretion of the SRC.

7.2.6. Definition of Dose-Limiting Toxicity (DLT)

During dose escalation, the DLT assessment period is Cycle 1 (28 days). National Cancer Institute (NCI) Common Terminology Criteria for Adverse Events (CTCAE), Version 4.03 are used as a guide for the grading of severity of adverse events. A DLT is defined as any of the following toxicities occurring within the DLT assessment unless the event can clearly be determined to be unrelated to CC-90010. Dose-limiting toxicities are described below:

- Hyperglycemia meeting any of the following criteria:
  - Grade 2 fasting hyperglycemia (> 160 mg/dL) lasting > 14 days despite optimal medical treatment
  - Grade 3 or greater fasting hyperglycemia (> 250 mg/dL) lasting > 4 days despite optimal medical treatment
  - Grade 4 hyperglycemia lasting ≥12 hours despite adequate treatment

  - Hyperglycemia associated with diabetic ketoacidosis or nonketotic hyperosmolar coma regardless of glucose level
  - Hyperglycemia that necessitates dose reduction despite dose interruption and resolution to ≤ grade 1 hyperglycemia within two weeks as described in Appendix I.
- Any Grade 4 non-hematologic toxicity of any duration
- Any non-hematologic toxicity Grade ≥ 3 EXCEPT for:
  - Grade 3 diarrhea, nausea, or vomiting of ≤ 3 days duration (with optimal medical management).
  - Grade 3 rash of the acneiform, pustular or maculopapular type which resolves to Grade ≤ 2 within 7 days of study drug interruption and does not recur at the same level with resumption of study drug at the same dose (with optimal medical management).
  - Grade 3 fatigue which resolves to Grade ≤ 2 within 7 days of study drug interruption and does not recur at the same level with resumption of study drug at the same dose (with optimal medical management).
- Hematological toxicities as follows:
  - Febrile neutropenia
  - Grade 4 neutropenia lasting > 7 days
  - Grade 4 thrombocytopenia lasting > 7 days, Grade ≥ 3 thrombocytopenia with clinically significant bleeding
- Any AE, Grade ≥ 2 not specified or exempt above, unless clearly determined to be unrelated to the drug, necessitating dose-level reduction during Cycle 1.

Isolated laboratory changes without associated clinical signs or symptoms (eg, hypomagnesemia, hypermagnesemia, hypoalbuminemia, hypophosphatemia, lymphocyte count increased or decreased) may not be included in this definition. These findings will be discussed and reviewed by the SRC.

7.2.7. Criteria for Dose Escalation in the Next Cohort of Subjects

During Part A, the dose escalation criteria are described in Section 7.2.2.

7.2.8. Definition of Stopping Criteria

During Part A, the dose escalation stopping rules are in Section 7.2.2.

7.2.9. Permitted Study Drug Adjustments

Dose reductions are permitted in any cycle, including Cycle 1.

- Dose reductions that occur in Cycle 1 during dose escalation will constitute DLT as outlined in Section 7.2.6, but subjects will be allowed to continue on CC-90010 at a reduced dose.

- When a dose adjustment is indicated, CC-90010 dose and/or the frequency will be adjusted. Two actual dose reductions are allowed.
- Once the dose has been reduced, it can be escalated when toxicity becomes Grade ≤ 1 after consultation and in agreement with the Sponsor´s study physician. If toxicity recurs at the higher dose, the dose will be reduced a second time, but no re-escalation is then permitted. If any subject continues to experience unacceptable toxicity after two dose reductions (one for the starting dose), CC-90010 will be discontinued permanently.

Intra-subject dose escalation will not be allowed during the DLT assessment period. Refer to Section 7.2.9.2 for additional information on possible dose increases.

7.2.9.1. Criteria for Dose Reduction

Any AE meeting the definition of DLT will require dose frequency adjustment and/or dose reduction or dose interruption if no recovery. Doses may be delayed or treatment interruptions introduced if any treatment related Grade ≥ 2 toxicities are not resolved to Grade ≤ 1 by the time of the next dose. Such cases should be discussed with the Sponsor´s study physician to determine the optimal duration of the dosing delay.

Treatment related Grade ≥ 3 toxicity or chronic Grade 2 toxicity may warrant dose reduction of CC-90010 (eg, from 15 mg to 10 mg). Such cases should be discussed with the Sponsor's study physician before dosing changes are made.

7.2.9.2. Criteria for Dose Increase

In Part A (escalation phase), intra-subject dose escalation beyond the doses initially assigned to a subject is not permitted in Cycle 1. Those continuing to take CC-90010 beyond Cycle 2 may, following approval by the SRC, have the dose increased providing the alternative dose has been shown to be well tolerated by at least one cohort of subjects in this study (ie, overdose risk is less than 25% based on the BLRM assessment).

In Part B (expansion phase), no dose escalation beyond the MTD is allowed.

7.2.9.3. Treatment Interruption for Adverse Events

In cases of Grade 2 toxicities such as nausea, fatigue, electrolyte alterations, hyperglycemia, AST or ALT elevation with normal or grade 1 bilirubin, etc, treatment interruption is not required. If warranted, treatment may be interrupted up to 4 weeks until toxicity (excluding alopecia) reaches either Grade ≤ 1 or baseline levels. Treatment interruption beyond 4 weeks may be allowed however, if in the opinion of the Investigator, it is clinically beneficial for the subject; this should be discussed with Sponsor's study physician. Treatment may restart either at the same, or a reduced dose, at the Investigator's discretion or as described in Section 7.2. Any such treatment interruptions must be discussed with the Sponsor's study physician.

In the DLT assessment period of the dose escalation phase, a treatment interruption resulting in <80% of CC-90010 doses in Part A for reasons other than DLT will make a subject non-evaluable for DLT and necessitate replacement of that subject in the dosing cohort. Any such treatment interruptions must be discussed with the Sponsor's study physician.

7.2.10. Management of Select Adverse Events

7.2.10.1. Neutropenia, Thrombocytopenia, and Anemia

Hematopoietic growth factors or other hematologic support, such as erythropoietin, darbepoetin, granulocyte-colony stimulating factor (G-CSF), granulocyte-macrophage colony stimulating factor (GM-CSF), RBC- or platelet- transfusions are allowed in the study with therapeutic intent. Therapeutic use of G-CSF is allowed at any time for subjects experiencing Grade 3/4 neutropenia or any grade febrile neutropenia. Prophylactic use of granulocyte (or granulocyte-macrophage) growth factors is not allowed during Cycle 1.

In Part A (dose escalation) (n=69), the most frequently reported treatment-emergent adverse events (TEAEs) (≥ 10% of subjects) were thrombocytopenia in 21 (30.4%) subjects or decreased platelet count in 13 (18.8%) subjects. There were 34 (49.3%) subjects who experienced at least one Grade 3 or 4 TEAE, the most common being thrombocytopenia in 7 (10.1%) subjects or decreased platelet count occurring in 3 (4.3%) subjects (see Section 1.3). Grade 3 and 4 thrombocytopenia was associated with low platelet counts at baseline. Similarly, thrombocytopenia was the DLT most frequently reported by Amorim et al, 2016. Both incidence and severity increased with dose and schedule. Of 17 patients with Grade 4 thrombocytopenia, 11 received platelet transfusions and four had Grade 1–2 bleeding (three patients had epistaxis and one patient had rectal haemorrhage). The thrombocytopenia nadir (Grade 4) occurred after a median of 18 days (Interquartile range [IQR] 15–26) from starting OTX015 treatment. Severe thrombocytopenia (Grade 3–4) was more frequent in patients with low platelet counts at baseline (Amorim, 2016).

Subjects with Grade ≥ 2 thrombocytopenia should not be dosed until recovery of thrombocytopenia to Grade ≤ 1. Once the thrombocytopenia has resolved to Grade ≤ 1, treatment can be resumed (discussion with the Sponsor's study physician is highly recommended).

Subjects with Grade 3 or 4 neutropenia should be monitored frequently with laboratory tests until resolution to Grade ≤ 1. Antimicrobial, antifungal, and antiviral prophylaxis should be considered.

7.2.10.2. Pain

Tumor pain or treatment-induced pain can be controlled with opioid and opioid-related analgesics, such as codeine, meperidine, propoxyphene or morphine, administered at the clinician's discretion, and as dictated by medical need. The risk of bleeding, especially in the setting of thrombocytopenia, should be considered prior to use of non-steroidal anti-inflammatory drugs (NSAIDs) and aspirin.

7.2.10.3. Gastrointestinal Effects

Mucosa coating agents for protection of esophageal/gastric mucosa are recommended at the discretion of the Investigator as well as monitoring subjects for GI bleeding. Subjects will be encouraged to report all episodes of GI discomfort or pain, appetite loss, or blood in stool.

It is recommended that subjects experiencing diarrhea be managed according to the guideline provided in Appendix J. Antidiarrheal medication, such as loperamide, should be initiated at the earliest onset of Grade 1-2 diarrhea. Antidiarrheal medication may be administered as

prophylaxis and for treatment of diarrhea. Dehydration and electrolyte disturbances should be rapidly corrected. General measures to improve diarrhea, such as a low-fiber diet and increase liquid consumption, should be considered.

7.2.10.4. Hyperglycemia

Transient changes in blood glucose were observed in only a few occasions in the nonclinical toxicology studies with CC-90010. Furthermore, preliminary clinical data of a new investigational BETi, OTX015, reported 7 of 37 patients with non-leukemic hematologic malignancies experienced Grade 1-2 hyperglycemia and 3 patients experienced Grade 3 hyperglycemia (Thieblemont, 2014). In Part A of the Study CC-90010-ST-001, 15 (21.7%) subjects experienced hyperglycemia (treatment-related in 8 subjects) and 4 (5.8%) subjects experienced Grade 3 hyperglycemia (treatment-related in 1 subject). In Part B of the study, 4 (33.3%) subjects experienced hyperglycemia (treatment-related in 3 subjects), 1 (8.3%) subject experienced Grade 3 hyperglycemia, assessed as treatment-related, and 1 (8.3%) subject experienced Grade 4 hyperglycemia, assessed as treatment-related. (see Section 1.3). General guidelines for the management of possible hyperglycemia are provided in Appendix I.

7.2.11. Definition of Overdose

Overdose, as defined for this protocol, refers to CC-90010 dosing only. On a per dose basis, an overdose is defined as the following amount over the protocol-specified doses of CC-90010 assigned to a given subject, regardless of any associated adverse events or sequelae:

- PO any amount over the protocol-specified dose

On a schedule or frequency basis, an overdose is defined as anything more frequent than the protocol required schedule or frequency.

Complete data about drug administration, including any overdose, regardless of whether the overdose was accidental or intentional, should be reported in the case report form. Refer to Section 10 for the reporting of adverse events associated with overdose.

7.3. Method of Treatment Assignment

Eligible subjects will be enrolled sequentially in Part A (dose escalation). Enrollment in Part B (dose expansion) will be stratified by disease cohort and dosing schedule, as applicable.

An Interactive Response Technology (IRT) system will be used to track subject assignments to the dose levels in Part A and tumor cohorts in Part B.

7.4. Packaging and Labeling

The labels for CC-90010 will include sponsor name, address and telephone number, the protocol number, IP name, dosage form and strength (where applicable), amount of CC-90010 per container, lot number, expiry date (where applicable), medication identification/kit number, storage conditions, and required caution statements and/or regulatory statements as applicable. Additional information may be included on the label as applicable per local regulations.

7.5. Investigational Product Accountability and Disposal

Celgene (or designee) will review with the Investigator and relevant site personnel the procedures for documenting receipt of CC-90010, as well as the procedures for counting, reconciling CC-90010, and documenting this process. Celgene (or designee) will also review with the Investigator and relevant site personnel the process for CC-90010 return, disposal, and/or destruction including responsibilities for the site vs. Celgene (or designee).

7.6. Investigational Product Compliance

Only the pharmacist or the Investigator's designee will dispense CC-90010. A record of the number of tablets of CC-90010 dispensed to and taken by each subject must be maintained. The pharmacist or the Investigator's designee will document the doses dispensed/administered in the appropriate study records.

Subjects will use diary cards to record their daily self-administration of CC-90010 at home. The person completing the diary card will sign/initial and date the cards in accordance with good documentation practice. These will be reviewed by study staff each time the subject visits the clinic. Entries will be clarified, as necessary, so that appropriate information can be captured on the eCRFs. Study site personnel will perform a CC-90010 administration compliance check and record this information on the subject's source documentation and on the appropriate eCRF.

8. CONCOMITANT MEDICATIONS AND PROCEDURES

All medications (excluding prior cancer therapy for the tumor under evaluation) taken beginning when the subject signs the ICD and all concomitant therapy during the study until 28 days after treatment discontinuation, together with dose, dose frequency and reasons for therapy use will be documented in the source documents and on the concomitant medication eCRF.

All prior cancer therapy for the tumor under evaluation, including chemotherapy, biologic, immunologic, irradiation, and surgery, will be documented on dedicated prior cancer treatment eCRFs.

The Investigator will instruct subjects to notify the study staff about any new medications taken after signing the ICD. All medications and significant non-drug therapies (herbal medicines, physical therapy, etc.) and any changes in dosing with existing medications will be documented on the eCRFs.

8.1. Permitted Concomitant Medications and Procedures

Subject to the precautions described in Section 8.2, the use of any concomitant medication/therapies deemed necessary for the care of the subject should be used. Repeat PK evaluations may be conducted if changes are made to concomitant medications suspected of affecting drug absorption or metabolism. The following are permitted concomitant medications and procedures:

- Subjects with ≥ Grade 1 diarrhea should promptly initiate treatment with eg, diphenyoxylate/atropine (Lomotil), or loperamide (Imodium) or an alternative over-the-counter remedy for diarrhea as per institutional practice. Premedication with antidiarrheal medication for subsequent doses of CC-90010 may be appropriate and should be discussed with medical monitor.
- Anti-emetics will be withheld until subjects have experienced CTCAE ≥ Grade 1 nausea or vomiting. Subjects may then receive prophylactic anti-emetics at the discretion of the Investigator.
- Subjects may receive prophylactic mucosa protective agents at the discretion of the Investigator.
- Therapeutic use of granulocyte growth factors is allowed at any time for subjects experiencing febrile neutropenia or Grade 3/4 neutropenia. Routine prophylaxis with granulocyte colony stimulating factor or granulocyte-macrophage colony stimulating factor is allowed at Investigator discretion starting with Cycle 2 and beyond.
- Subjects receiving stable doses of recombinant erythropoietin or darbepoetin alfa for at least 4 weeks prior to starting the CC-90010 may continue their pretreatment doses throughout the study. Subjects may initiate de novo treatment with erythropoietin stimulating agents (ESAs) beginning in Cycle 2 for hypoproliferative anemias secondary to prior chemotherapy exposure provided there is no clinical suspicion of a concurrent cause for the anemia (eg, CC-90010-induced).
- Parenteral flu vaccination is permitted.

- Routine infectious disease prophylaxis is not required. However, antibiotic, antiviral, antipneumocystis, antifungal, or other prophylaxis may be implemented during the study at the discretion of the Investigator.
- Treatment with bisphosphonates (eg, pamidronate, zolendronate) or other agents (eg, denosumab) is permitted to prevent or delay progression of bone metastases. Maintenance of a stable dosing regimen throughout the study is recommended.
- Focal palliative radiotherapy for treatment of cancer-related symptoms (eg, localized bone pain) is allowed during study treatment at the discretion of the investigator.
- Subjects may receive physiologic replacement doses of glucocorticoids (up to the equivalent of 10 mg daily prednisone) as maintenance therapy for adrenal insufficiency.
- Maintenance hormonal therapies are allowed in subjects with a history of breast or prostate cancer.
- For subjects with recurrent GBM, anti-convulsive medication may be used during the study at the discretion of the investigator.
- For subjects at risk of brain edema secondary to brain metastasis and radiotherapy, a steroid dose (up to the equivalent of 4 mg/day dexamethasone can be used) at the discretion of the Investigator.
- As a precautionary measure, it is recommended that subjects avoid prolonged exposure to UV light, wear protective clothing and sunglasses, and use UV-blocking topical preparations while taking CC-90010.

8.2. Prohibited Concomitant Medications and Procedures

Other investigational therapies must not be used while the subject is on the study.

Anticancer therapy (chemotherapy, biologic or investigational therapy, and surgery) other than the study treatments must not be given to subjects while the subject is on the study. If such treatment is required the subject must be discontinued from the study. Treatment with immunosuppressive agents is not allowed while the subject is on the study. If such treatment is required the subject must be discontinued from the study.

Treatment with chronic, therapeutic dosing of anti-coagulants (eg, warfarin, low molecular weight heparin, Factor Xa inhibitors, thrombin antagonists) is not allowed. Short-term, prophylactic dosing of anticoagulants may be considered in subjects if medically indicated (eg, hospitalized subjects, post-operatively) under careful consideration by the Investigator.

Routine prophylaxis with granulocyte-colony stimulating factor (G-CSF), granulocyte macrophage-colony stimulating factor (GM-CSF) is not allowed during Cycle 1.

Live vaccines may not be administered to subjects in this trial. Killed inactivated vaccines, such as an injectable annual influenza vaccine, are permitted.

CC-90010 is an inhibitor of P-gp and has the potential to interact with drugs that are substrates of P-gp. Drugs that are known to be strong P-gp substrates and have narrow therapeutic index (ie, digoxin and apixaban) should be avoided. If use of one of these drugs becomes necessary, the

risks and benefits should be discussed with the Sponsor's study physicians prior to its concomitant use with CC-90010. To address the potential PDE5 or PDE6 inhibition-specific toxicities, co-administration of protease inhibitors, grapefruit and grapefruit juice, anti-hypertensives, alpha blockers, nitrates, guanylate cyclase stimulators, and PDE5 inhibitors should be carefully considered

CC-90010 is primarily metabolized by CYP3A4/5. Drugs that are known to be strong inducers or inhibitors of CYP3A4/5 should be avoided. If use of one of these drugs is necessary, the risks and benefits should be discussed with the Sponsor's study physician prior to its concomitant use with CC-90010.

Examples of these drugs are (not inclusive):

- CYP3A4/5 inhibitors: atazanavir, clarithromycin, indinavir, itraconazole, ketoconazole, nefazodone, nelfinavir, ritonavir, saquinavir, and telithromycin
- CYP3A4/5 inducers: rifampin and carbamazepine

9. STATISTICAL CONSIDERATIONS

9.1. Overview

Data summaries/statistical analyses will be performed by study part (Part A B and C), dose schedule, dose level (Part A), tumor cohort (Part B) and fasting status (Part C) as applicable.

9.2. Study Population Definitions

The study population definitions are as follows:

- Enrolled Population – All subjects who are assigned an enrollment number and meet inclusion/exclusion criteria.
- Treated Population – All subjects who enroll and receive at least one dose of CC-90010.
- Efficacy Evaluable (EE) Population – All subjects who enroll in the study, meet eligibility criteria, complete at least one cycle of CC-90010 (taking at least 80% of assigned doses), and have baseline and at least one valid post-baseline tumor assessment.
- Pharmacokinetic (PK) Evaluable Population – all subjects who enroll and receive at least one dose of CC-90010 and have at least one measurable concentration of CC-90010.
- Biomarker Evaluable (BE) Population – all subjects who enroll, receive at least one dose of CC-90010, and have at least one biomarker assessment, excluding disqualified assessments.
- PK completer population (Part C only): all subjects who have adequate PK data to allow calculation of AUC from time 0-hour (Day 1 of Cycle) through the 432-hour (Day 22 of Cycle) time point from both Periods 1 and 2.

9.3. Sample Size and Power Considerations

During Part A of the study, an adaptive BLR model (with 2 parameters) guided by the EWOC principle will be for dose escalation as described in Section 7.2.2. No formal statistical power calculations to determine sample size were performed for this study. The actual number of subjects will depend on the number of dose levels/cohorts that are tested. However, the anticipated number of subjects in Part A will be approximately 85.

After the MTD is determined from Part A, Part B expansion phase will further evaluate the safety and efficacy of CC-90010 as a single agent administered at or below the MTD in selected expansion cohorts:

- Cohort 1: relapsed and/or refractory DLBCL - approximately 20-25 evaluable subjects at 45 mg CC-90010 4-days-on/24-days-off in each 28-day cycle
- Cohort 2: advanced BCC – enrollment stopped due to recruitment challenges
- Cohort 3: relapsed and/or refractory DLBCL - approximately 15 evaluable subjects at 30 mg CC-90010 3-days-on/11-days-off in each 28-day cycle

For Part B, sample sizes are not determined based on power calculation but rather on clinical, empirical and practical considerations traditionally used for exploratory studies of this kind. During the Part B dose expansion, at least 15 efficacy evaluable subjects will initially be accrued in Cohort 1. Cohort 1 will be expanded to approximately 20-25 subjects if a responder or SD of 4 months or longer is observed.

For the cohort 1 and 3, if the criteria of declaring futility at the end of study is Pr (ORR < 26%) >80%, N=25 (cohort 1) and N=15 (cohort 3) will provide 73% and 65% chances, respectively, to declare futility given the true ORR = 14% (based on posterior probability of beta-binomial distribution with prior beta (0.35, 1).

A total of 24 subjects in the food effect study in Part C will provide approximately 15% precision level for the estimated geometric mean ratios of $AUC_{0-\infty}$ (area under the concentration-time curve from 0 extrapolated to infinity) between Treatment A (Fasting) and B (Fed) assuming intra-subject coefficient of variance (CV) is 0.3, that is, if there is no food effect on $AUC_{0-\infty}$, the observed geometric mean ratios of $AUC_{0-\infty}$ will be somewhere between 0.86 and 1.16. The precision represents the half-width of the 90% confidence interval of the geometric mean ratios on natural-scale, as shown in the Table below. Based on the preliminary PK results from Part A of the present study CC-90010-ST-001, the intra-subject CV is approximately 0.25 – 0.3. The precision in the comparison of PK parameters are calculated for different estimates of intra-subject CV in .

Table 14: Precision based on Estimates of Intra-subject Coefficient of Variance

Number of Subjects	24				
Intra-subject CV	0.1	0.2	0.25	0.3	0.4
Precision	5%	10.3%	13%	15.7%	21%

CV: Coefficient of Variance.

9.4. Background and Demographic Characteristics

In Part A dose escalation, the baseline characteristics of subjects will be summarized by dose cohort for the enrolled population. In Part B dose expansion, the baseline characteristics of subjects will be summarized by cohort/tumor type. The age, weight, height and other continuous demographic and baseline variables will be summarized using descriptive statistics. Performance status, gender, race and other categorical variables will be summarized with frequency tabulations. Medical history data will be summarized using frequency tabulations by system organ class and preferred term.

9.5. Subject Disposition

Subject disposition (analysis population allocation, on-going, discontinued, along with primary reason) from treatment and study will be summarized using frequency and percent. A summary of subjects enrolled by site will be provided. Protocol violations will be summarized using frequency tabulations. Supportive corresponding subject listings will also be provided.

9.6. Efficacy Analysis

Efficacy analyses will be based on the treated population and include summaries of clinical benefit rate (CBR), objective response rate (ORR), duration of response or stable disease, progression-free survival (PFS), and OS by dose cohort and dosing schedule (Part A) or tumor type and dosing schedule (Part B). Tumor response (CR, PR, SD, PD, or inevaluable) will be assessed by investigators according to Response Evaluation Criteria in Solid Tumors (RECIST), version 1.1 and IWG criteria. For subjects with glioblastoma multiforme, assessment will be based on the Response Assessment in Neuro-Oncology criteria (Wen, 2010). For LaBCC subjects, a conglomeration of radiology of target lesions assessed by RECIST 1.1, digital clinical photography assessed by WHO (bi-dimensional assessment) (Miller, 1981) and punch biopsies to confirm CR or if response confounded by lesion ulceration, cyst, or scarring/fibrosis will be used.

The CBR is defined as tumor responses (as assessed by the Investigators) of complete response (CR), partial response (PR) and stable disease (SD) (SD of ≥ 4 months duration). The ORR is defined as the percent of subjects whose best response is CR or PR. When SD is the best response, it must be documented radiographically at least once after study entry after a minimal interval of 7 weeks (ie, coincident with the first post baseline response assessment time point minus assessment window). If the minimal time for a best response of SD is not met, the subject's best response will depend on the outcome of subsequent assessments. For example, a subject who exhibits SD at first assessment (where the first assessment does not meet minimal duration criteria for SD) and PD at the second assessment, would be classified as having a best response of PD. A subject lost to follow-up after the first SD assessment would be considered non-evaluable, if the minimal duration criteria for SD were not met.

Two-sided 95% Clopper-Pearson exact confidence intervals will be provided for ORR and CBR estimates. Similar analyses will be performed to include those subjects with confirmed responses as well as for the Efficacy Evaluable population.

For subjects with best response of CR or PR, duration of response is measured from the time when criteria for CR/PR are first met (whichever is first recorded) until the first date at which progressive disease is objectively documented. For subjects with best response of SD, duration of SD is measured from the first dose date until the criteria for progression are met. If progression is not documented prior to CC-90010 discontinuation, duration of overall response, and duration of SD will be censored at the date of the last adequate tumor assessment.

Duration of response/SD based on investigators' assessments will be summarized by descriptive statistics (mean, standard deviation, median, minimum and maximum) for the treated population. Except for medians, which will be calculated based on both observed and censored values using the Kaplan-Meier method, all other statistics (mean, standard deviation, minimum and maximum) will be calculated based on observed values only.

Progression-Free Survival (PFS) is defined as the time from the first dose of CC-90010 to the first occurrence of disease progression or death from any cause. Subjects who neither progress nor die at a data cut-off date will be censored at the date of their last adequate tumor assessment. The PFS will be summarized using descriptive statistics (mean, standard deviation, median, minimum and maximum) for the treated population. Except for the median, which will be calculated based on both observed and censored values using the Kaplan-Meier method, all other

statistics (mean, standard deviation, minimum and maximum) will be calculated based on observed values only.

Overall Survival (OS) is measured as the time from the first dose of CC-90010 to death due to any cause and will be analyzed in a manner similar to that described for PFS.

9.7. Safety Analysis

Adverse events, including treatment-emergent adverse events (TEAEs), laboratory assessments, vital signs, ECG results, ECOG performance status, LVEF assessments, physical examinations, vital signs, exposure to study treatment, assessment of concomitant medications, and pregnancy testing for females of childbearing potential will be summarized for the treated population (by dose cohort in Part A dose escalation and cohort/tumor type in Part B).

Adverse events observed will be classified using the Medical Dictionary for Regulatory Activities (MedDRA), Version 17.1 or higher, system organ class (SOC) and preferred term (PT). In the by-subject analysis, a subject having the same AE more than once will be counted only once. All adverse events will also be summarized by SOC, PT, and NCI CTCAE grade (Version 4.0 or higher). Adverse events leading to discontinuation of study treatment, those classified as Grade 3 or 4, study drug-related AEs, and SAEs (including deaths) will be tabulated separately. By-subject listings of all AEs, TEAEs, SAEs (including deaths), and their attribution will be provided.

Clinical laboratory results will be summarized descriptively by dose cohort (Part A) or cohort/tumor type (Part B) and visit, which will also include a display of change from baseline. Shift tables demonstrating the changes (low/normal/high) from baseline to worst post-baseline laboratory value will be displayed in cross-tabulations by dose cohort (Part A) or cohort/tumor type (Part B). Similar shift tables demonstrating the change of NCI CTCAE grades from baseline to the worst post-baseline severity grade during the treatment period will also be presented by dose cohort (Part A) or tumor type (Part B) for applicable analytes. Listings of abnormal clinical laboratory data according to NCI CTCAE severity grades (if applicable), abnormal flags (low or high) and clinical significance of the latter will be provided.

Graphical displays (eg, "spaghetti" plots or box plots) will be provided for key laboratory analytes.

Descriptive statistics for vital signs, both observed values and changes from baseline, will be summarized by dose cohort (Part A) or cohort/tumor type (Part B) and visit. Shift tables demonstrating the changes from baseline to the worst post-baseline value will be displayed in cross-tabulations by dose cohort (Part A) or tumor type (Part B). Vital sign measurements will be listed by subject and by visit.

ECG parameters and changes from baseline will be summarized by dose cohort (Part A) or cohort/tumor type (Part B) and visit using descriptive statistics. Post-baseline abnormal QTc (both QTcF and QTcB) values will be summarized using frequency tabulations for the following 5 categories:

- QTc > 450 msec
- QTc > 480 msec

- QTc > 500 msec
- QTc increase from baseline > 30 msec
- QTc increase from baseline > 60 msec.

Shift from baseline to worst post-baseline qualitative assessment of abnormality (ie, 'Normal', 'Abnormal, not clinically significant', and 'Abnormal, clinically significant' or 'Normal' and 'Abnormal') will be displayed in cross–tabulations by dose cohort (Part A) or cohort/tumor type (Part B). A listing of ECG parameters by subject, by visit will be provided.

9.8. Interim Analysis

No formal interim analysis is planned. Data will be reviewed on an ongoing basis.

9.9. Other Topics

9.9.1. Statistical Method for Dose Escalation

An adaptive BLRM guided by the escalation with EWOC principle will be used to make dose recommendations and estimate the MTD during the escalation phase of the study (refer to Appendix K for additional details).

The DLT relationship in the escalation part of the study will be described by the following Bayesian logistic regression model:

$$logit(p_i) = \log(\alpha) + \beta \log\left(\frac{d_i}{d^*}\right) + \gamma_1 x_{i1} + \gamma_2 x_{i2} + \gamma_3 x_{i3}$$

We use total dose within one cycle as d_i, $d^* = 314$. Schedule 1(3 days on / 4 days off) is used as referent, $\gamma_1, \gamma_2, \gamma_{13}$ represent Schedule 2 (3 days on/11 days off), 3 (2 days on /5 days off), and 4 (4 days on/24 days off).

Prior Specifications

The parameters of the prior distributions of model parameters are selected based on the method to construct weakly informative prior as described in (Neuenschwander, 2015) and are provided in Table 15. This results in wide confidence intervals for the probabilities of a DLT at each dose. The probability of DLT for the first dose is assumed to be low. Table 15 demonstrates the parameters for prior distribution.

Table 15: Prior Parameters for Bivariate Normal Distribution of Model Parameters

Parameters	Mean	Standard Deviation	Correlation
$\log(\alpha), \log(\beta)$	(-0.693, 0)	(2, 1)	0
γ_1	0.68	0.643031	NA
γ_2	0.30	0.84764	NA
γ_3	1.09	0.4053196	NA

Dose Recommendation

The provisional CC-90010 dose levels (total doses within on cycle) are: 180 mg in Schedule 1, 90 mg, 150 mg, 240 mg in Schedule 2, 120 mg, 200 mg, 280 mg in Schedule 3, 120 mg, 180 mg, 220 mg in Schedule 4. It is however possible for some doses to be skipped or additional dose levels to be added during the course of the study, based on the emerging safety information. After each cohort the posterior distributions for the probabilities of a DLT rates at different dose levels are obtained. The results of this analysis are summarized in terms of the estimated probabilities that the true rate of DLT at each dose-level will have of lying in each of the following intervals:

- [0, 0.16) under-dosing
- [0.16, 0.33) targeted toxicity
- [0.33, 1.00] excessive toxicity.

Following the principle of escalation with EWOC, after each cohort of subjects the recommended dose is the one with the highest posterior probability of the DLT rate falling in the target interval [16%, 33%) among the doses fulfilling EWOC, ie, it is unlikely (<25% posterior probability) that the DLT rate at the dose falls in the excessive toxicity interval.

Note that the dose that maximizes the posterior probability of targeted toxicity is the best estimate of the MTD, but it may not be an admissible dose according to the overdose criterion if the amount of data is insufficient. If vague prior information is used for the probabilities of DLT, in the early stages of the study this escalation procedure will reflect a conservative strategy.

The dose recommended by the adaptive Bayesian logistic model may be regarded as guidance and information to be integrated with a clinical assessment of the toxicity profiles observed at the time of the analysis in determining the next dose level to be investigated.

Details of the dose escalation and determination of the MTD are provided in Section 7.2.

9.9.2. Assessment of Pharmacokinetics (Part A and Part B)

Plasma PK parameters such as AUC_{0-24}, C_{max}, T_{max}, $t_{1/2}$, CL/F, and V_z/F of CC-90010 will be calculated by the noncompartmental analysis method from the plasma concentration-time profiles of CC-90010. Additional PK parameters including CSF to plasma concentration ratio of CC-90010, may be calculated, if data permits.

Summary statistics including number of subjects (N), mean, standard deviation (SDev), coefficient of variation (CV%), geometric mean, geometric CV%, median, minimum, and maximum will be provided for CC-90010 concentration by nominal time point, study day, and dose cohort. Mean and individual plots of plasma concentrations will be presented in both original and semi-logarithmic scales. Summary statistics will also be provided for CC-90010 PK parameters by study day and dose cohort and be presented in tabular form.

A population PK analysis for CC-90010 may be conducted to explore the inter-individual variability of plasma drug exposure and the contributing factors (covariates). The relationship between CC-90010 dose, plasma exposures, and selected clinical endpoints (eg, measures of toxicities, effectiveness, and/or biomarkers) will be explored. The population PK model, in

EDMS Doc. Number: [redacted]

combination with the knowledge on exposure-response, may be used to assist in identification of the dosing regimen for Part B or Phase 2 studies.

9.9.3. Assessment of Pharmacokinetics in Food Effect Evaluation (Part C)

To characterize the PK of CC-90010 under fasted and fed conditions in the food effect evaluation, an analysis of variance will be performed on the natural log-transformed AUC_{0-24}, AUC_{0-Last}, $AUC_{0-\infty}$ (data permitting), and C_{max} of CC-90010 using MIXED procedure in SAS®. The MIXED model will contain terms for sequence, period, and treatment as fixed effects, and subject nested within sequence as a random effect. The geometric mean ratios (fed/fasted) and their 90% CIs will be provided. For T_{max}, a nonparametric analysis will be used to produce a median difference between treatments.

Results will be presented in tabular and graphic forms, as appropriate.

9.9.4. Assessment of Pharmacodynamics

Descriptive statistics (N, mean, SD, median, min, and max) will be provided for baseline, post-baseline values, and changes from baseline or percent change from baseline of each biomarker by dose cohort (Part A) or cohort/tumor type (Part B) and visit. Subjects' biomarker results over time will be plotted. Comparison of biomarker levels before and during treatment will be performed by Wilcoxon signed rank test. If sufficient and valid results from biomarker assays can be obtained, the relationship between percent changes in biomarker levels and clinical endpoints including ORR and CBR will be explored.

10. ADVERSE EVENTS

10.1. Monitoring, Recording and Reporting of Adverse Events

An AE is any noxious, unintended, or untoward medical occurrence that may appear or worsen in a subject during the course of a study. It may be a new intercurrent illness, a worsening concomitant illness, an injury, or any concomitant impairment of the subject's health, including laboratory test values (as specified by the criteria in Section 10.3), regardless of etiology. Any worsening (ie, any clinically significant adverse change in the frequency or intensity of a pre-existing condition) should be considered an AE. A diagnosis or syndrome should be recorded on the AE page of the CRF rather than the individual signs or symptoms of the diagnosis or syndrome.

Abuse, withdrawal, sensitivity or toxicity to an investigational product should be reported as an AE. Overdose, accidental or intentional, whether or not it is associated with an AE should be reported on the overdose CRF (refer to Section 7.2.11 for the definition of overdose.) Any sequela of an accidental or intentional overdose of an investigational product should be reported as an AE on the AE CRF. If the sequela of an overdose is an SAE, then the sequela must be reported on an SAE report form and on the AE CRF. The overdose resulting in the SAE should be identified as the cause of the event on the SAE report form and CRF but should not be reported as an SAE itself.

In the event of overdose, the subject should be monitored as appropriate and should receive supportive measures as necessary. There is no known specific antidote for CC-90010 overdose. Actual treatment should depend on the severity of the clinical situation and the judgment and experience of the treating physician.

All subjects will be monitored for AEs during the study. Assessments may include monitoring of any or all of the following parameters: the subject's clinical symptoms, laboratory, pathological, radiological or surgical findings, physical examination findings, or findings from other tests and/or procedures.

All AEs will be recorded by the Investigator from the time the subject signs informed consent until 28 days after the last dose of CC-90010 as well as those SAEs made known to the Investigator at any time thereafter that are suspected of being related to CC-90010. AEs and SAEs will be recorded on the AE page of the CRF and in the subject's source documents. All SAEs must be reported to Celgene Drug Safety within 24 hours of the Investigator's knowledge of the event by facsimile, or other appropriate method, using the SAE Report Form, or approved equivalent form.

10.2. Evaluation of Adverse Events

A qualified Investigator will evaluate all adverse events as to:

10.2.1. Seriousness

An SAE is any AE occurring at any dose that:

- Results in death;

- Is life-threatening (ie, in the opinion of the Investigator, the subject is at immediate risk of death from the AE);
- Requires inpatient hospitalization or prolongation of existing hospitalization (hospitalization is defined as an inpatient admission, regardless of length of stay);
- Results in persistent or significant disability/incapacity (a substantial disruption of the subject's ability to conduct normal life functions);
- Is a congenital anomaly/birth defect;
- Constitutes an important medical event.

Important medical events are defined as those occurrences that may not be immediately life-threatening or result in death, hospitalization, or disability, but may jeopardize the subject or require medical or surgical intervention to prevent one of the other outcomes listed above. Medical and scientific judgment should be exercised in deciding whether such an AE should be considered serious.

Events **not considered** to be SAEs are hospitalizations for:

- a standard procedure for protocol therapy administration. However, hospitalization or prolonged hospitalization for a complication of therapy administration will be reported as an SAE.
- routine treatment or monitoring of the studied indication not associated with any deterioration in condition.
- the administration of blood or platelet transfusion as routine treatment of studied indication. However, hospitalization or prolonged hospitalization for a complication of such transfusion remains a reportable SAE.
- a procedure for protocol/disease-related investigations (eg, surgery, scans, endoscopy, sampling for laboratory tests, bone marrow sampling). However, hospitalization or prolonged hospitalization for a complication of such procedures remains a reportable SAE.
- hospitalization or prolongation of hospitalization for technical, practical, or social reasons, in absence of an AE.
- a procedure that is planned (ie, planned prior to start of treatment on study); must be documented in the source document and the CRF. Hospitalization or prolonged hospitalization for a complication remains a reportable SAE.
- an elective treatment of or an elective procedure for a pre-existing condition, unrelated to the studied indication, that has not worsened from baseline.
- emergency outpatient treatment or observation that does not result in admission, unless fulfilling other seriousness criteria above.

If an AE is considered serious, both the AE page/screen of the CRF and the SAE Report Form must be completed.

For each SAE, the Investigator will provide information on severity, start and stop dates, relationship to the IP, action taken regarding the IP, and outcome.

10.2.2. Severity/Intensity

For both AEs and SAEs, the Investigator must assess the severity/ intensity of the event.

The severity/intensity of AEs will be graded based upon the subject's symptoms according to the current active minor version of the Common Terminology Criteria for Adverse Events (CTCAE, Version 4.03); http://ctep.cancer.gov/protocolDevelopment/electronic_applications/ctc.htm#ctc_40

AEs that are not defined in the CTCAE should be evaluated for severity/intensity according to the following scale:

- Grade 1 = Mild – transient or mild discomfort; no limitation in activity; no medical intervention/therapy required
- Grade 2 = Moderate – mild to moderate limitation in activity, some assistance may be needed; no or minimal medical intervention/therapy required
- Grade 3 = Severe – marked limitation in activity, some assistance usually required; medical intervention/therapy required, hospitalization is possible
- Grade 4 = Life-threatening – extreme limitation in activity, significant assistance required; significant medical intervention/therapy required, hospitalization or hospice care probable
- Grade 5 = Death - the event results in death

The term "severe" is often used to describe the intensity of a specific event (as in mild, moderate or severe myocardial infarction); the event itself, however, may be of relatively minor medical significance (such as severe headache). This criterion is *not* the same as "serious" which is based on subject/event *outcome* or *action* criteria associated with events that pose a threat to a subject's life or functioning.

Seriousness, not severity, serves as a guide for defining regulatory obligations.

10.2.3. Causality

The Investigator must determine the relationship between the administration of the CC-90010 and the occurrence of an AE/SAE as Not Suspected or Suspected as defined below:

Not suspected: a causal relationship of the adverse event to CC-90010 administration is **unlikely or remote**, or other medications, therapeutic interventions, or underlying conditions provide a sufficient explanation for the observed event.

Suspected: there is a **reasonable possibility** that the administration of CC-90010 caused the adverse event. 'Reasonable possibility' means there is evidence to suggest a causal relationship between the IP and the adverse event.

Causality should be assessed and provided for every AE/SAE based on currently available information. Causality is to be reassessed and provided as additional information becomes available.

If an event is assessed as suspected of being related to a comparator, ancillary or additional CC-90010 that has not been manufactured or provided by Celgene, please provide the name of the manufacturer when reporting the event.

10.2.4. Duration

For both AEs and SAEs, the Investigator will provide a record of the start and stop dates of the event.

10.2.5. Action Taken

The Investigator will report the action taken with IP as a result of an AE or SAE, as applicable (eg, discontinuation, interruption, or dose reduction of IP, as appropriate) and report if concomitant and/or additional treatments were given for the event.

10.2.6. Outcome

The Investigator will report the outcome of the event for both AEs and SAEs.

All SAEs that have not resolved upon discontinuation of the subject's participation in the study must be followed until recovered (returned to baseline), recovered with sequelae, or death (due to the SAE).

10.3. Abnormal Laboratory Values

An abnormal laboratory value is considered to be an AE if the abnormality:

- results in discontinuation from the study;
- requires treatment, modification/ interruption of CC-90010 dose, or any other therapeutic intervention; or
- is judged to be of significant clinical importance, eg, one that indicates a new disease process and/or organ toxicity, or is an exacerbation or worsening of an existing condition.

Regardless of severity grade, only laboratory abnormalities that fulfill a seriousness criterion need to be documented as a serious adverse event.

If a laboratory abnormality is one component of a diagnosis or syndrome, then only the diagnosis or syndrome should be recorded on the AE page/screen of the CRF. If the abnormality was not a part of a diagnosis or syndrome, then the laboratory abnormality should be recorded as the AE. If possible, the laboratory abnormality should be recorded as a medical term and not simply as an abnormal laboratory result (eg, record thrombocytopenia rather than decreased platelets).

10.4. Pregnancy

All pregnancies or suspected pregnancies occurring in either a female subject of childbearing potential or partner of childbearing potential of a male subject are immediately reportable events.

The exposure of any pregnant female (eg, caregiver, pharmacist, study coordinator or monitor) to CC-90010 is also an immediately reportable event.

10.4.1. Females of Childbearing Potential:

Pregnancies and suspected pregnancies (including elevated β-hCG or positive pregnancy test in a female subject of childbearing potential regardless of disease state) occurring while the subject is on CC-90010, or within 46 days of the subject's last dose of CC-90010, are considered immediately reportable events. Investigational product is to be discontinued immediately. The pregnancy, suspected pregnancy, or positive pregnancy test must be reported to Celgene Drug Safety immediately by email, phone or facsimile, or other appropriate method, using the Pregnancy Initial Report Form, or approved equivalent form.

The female subject should be referred to an obstetrician-gynecologist, preferably one experienced in reproductive toxicity for further evaluation and counseling. The Investigator will follow the female subject until completion of the pregnancy, and must notify Celgene Drug Safety immediately about the outcome of the pregnancy (either normal or abnormal outcome) using the Pregnancy Follow-up Report Form, or approved equivalent form.

If the outcome of the pregnancy was abnormal (eg, spontaneous abortion), the Investigator should report the abnormal outcome as an AE. If the abnormal outcome meets any of the serious criteria, it must be reported as an SAE to Celgene Drug Safety by facsimile, or other appropriate method, within 24 hours of the Investigator's knowledge of the event using the SAE Report Form, or approved equivalent form.

All neonatal deaths that occur within 28 days of birth should be reported, without regard to causality, as SAEs. In addition, any infant death after 28 days that the Investigator suspects is related to the in utero exposure to CC-90010 should also be reported to Celgene Drug Safety by facsimile, or other appropriate method, within 24 hours of the Investigator's knowledge of the event using the SAE Report Form, or approved equivalent form.

10.4.2. Male Subjects

If a female partner of a male subject taking CC-90010 becomes pregnant, the male subject taking CC-90010 should notify the Investigator, and the pregnant female partner should be advised to call their healthcare provider immediately. This includes pregnancies occurring within 106 days of the male subject's last dose of CC-90010. Where applicable, the CC-90010 may need to be discontinued in the male subject, but may be resumed later at the discretion of the Investigator and medical monitor.

10.5. Reporting of Serious Adverse Events

Any AE that meets any criterion for an SAE requires the completion of an SAE Report Form in addition to being recorded on the AE page/screen of the CRF. All SAEs must be reported to Celgene Drug Safety within 24 hours of the Investigator's knowledge of the event by facsimile, or other appropriate method (eg, via email), using the SAE Report Form, or approved equivalent form. This instruction pertains to initial SAE reports as well as any follow-up reports.

The Investigator is required to ensure that the data on these forms is accurate and consistent. This requirement applies to all SAEs (regardless of relationship to CC-90010) that occur during

the study (from the time the subject signs informed consent until 28 days after the last dose of CC-90010) or any SAE made known to the Investigator at any time thereafter that are suspected of being related to CC-90010. Serious adverse events occurring prior to treatment (after signing the ICD) will be captured.

The SAE report should provide a detailed description of the SAE and include a concise summary of hospital records and other relevant documents. If a subject died and an autopsy has been performed, copies of the autopsy report and death certificate are to be sent to Celgene Drug Safety as soon as these become available. Any follow-up data should be detailed in a subsequent SAE Report Form, or approved equivalent form, and sent to Celgene Drug Safety.

Where required by local legislation, the Investigator is responsible for informing the Institutional Review Board/Ethics Committee (IRB/EC) of the SAE and providing them with all relevant initial and follow-up information about the event. The Investigator must keep copies of all SAE information on file including correspondence with Celgene and the IRB/EC.

10.5.1. Safety Queries

Queries pertaining to SAEs will be communicated from Celgene Drug Safety to the site via facsimile or electronic mail. The response time is expected to be no more than five (5) business days. Urgent queries (eg, missing causality assessment) may be handled by phone.

10.6. Expedited Reporting of Adverse Events

For the purpose of regulatory reporting, Celgene Drug Safety will determine the expectedness of events suspected of being related to CC-90010 based on the Investigator's Brochure accordingly.

In the United States, all suspected unexpected serious adverse reactions (SUSARs) will be reported in an expedited manner in accordance with 21 CFR 312.32.]

For countries within the European Economic Area (EEA), Celgene or its authorized representative will report in an expedited manner to Regulatory Authorities and Ethics Committees concerned, suspected unexpected serious adverse reactions (SUSARs) in accordance with Directive 2001/20/EC and the Detailed Guidance on collection, verification and presentation of adverse reaction reports arising from clinical trials on investigational products for human use (ENTR/CT3) and also in accordance with country-specific requirements.

Adverse events such as disease progression, death related to disease progression (in the absence of serious CC-90010-related events) and serious events due to the relapse of the studied indication will not be subject to expedited reporting by the Sponsor to regulatory authorities.

Celgene or its authorized representative shall notify the Investigator of the following information:

- Any AE suspected of being related to the use of CC-90010 in this study or in other studies that is both serious and unexpected (eg, SUSAR);
- Any finding from tests in laboratory animals that suggests a significant risk for human subjects including reports of mutagenicity, teratogenicity, or carcinogenicity.

Where required by local legislation, the Investigator shall notify his/her IRB/EC promptly of these new serious and unexpected AE(s) or significant risks to subjects.

The Investigator must keep copies of all pertinent safety information on file including correspondence with Celgene and the IRB/EC (refer to Section 14.3 for record retention information).

Celgene Drug Safety Contact Information:

For Celgene Drug Safety contact information, please refer to the Serious Adverse Event Report Form Completion Guidelines or to the Pregnancy Report Form Completion Guidelines.

11. DISCONTINUATIONS

11.1. Treatment Discontinuation

The following events are considered sufficient reasons for discontinuing a subject from the investigational product:

- Adverse Event
- Withdrawal by subject
- Lack of efficacy
- Physician decision
- Protocol violation
- Progressive disease
- Death
- Lost to follow-up
- Other (to be specified on the CRF)

The reason for discontinuation of treatment should be recorded in the CRF and in the source documents.

The decision to discontinue a subject from treatment remains the responsibility of the treating physician, which will not be delayed or refused by the Sponsor. However, prior to discontinuing a subject, the Investigator may contact the Medical Monitor and forward appropriate supporting documents for review and discussion.

11.2. Study Discontinuation

The following events are considered sufficient reasons for discontinuing a subject from the study:

- Screen failure
- Adverse event
- Withdrawal by subject
- Lack of efficacy
- Physician decision
- Protocol violation
- Progressive disease
- Death
- Lost to follow-up
- Other (to be specified on the CRF)

The reason for study discontinuation should be recorded in the CRF and in the source documents.

12. EMERGENCY PROCEDURES

12.1. Emergency Contact

In emergency situations, the Investigator should contact the responsible Sponsor's study physician/Medical Monitor or designee by telephone at the number(s) listed on the Emergency Contact Information page of the protocol (after title page).

In the unlikely event that the Sponsor's study physician/Medical Monitor or designee cannot be reached, please contact the global Emergency Call Center by telephone at the number listed on the Emergency Contact Information page of the protocol (after title page). This global Emergency Call Center is available 24 hours a day and 7 days a week. The representatives are responsible for obtaining your call-back information and contacting the on-call Celgene/contract research organization Medical Monitor, who will then contact you promptly.

Note: The back-up 24-hour global emergency contact call center should only be used if you are not able to reach the Sponsor's study physician/or Medical Monitor or designee for emergency calls.

12.2. Emergency Identification of Investigational Products

This is an open-label study; therefore, CC-90010 will be identified on the package labeling.

Subjects enrolled in this study will be issued an identification card showing the name of this study and an emergency contact number. This can be used by health care professionals seeking emergency information about a subject's participation in the study.

13. REGULATORY CONSIDERATIONS

13.1. Good Clinical Practice

The procedures set out in this study protocol pertaining to the conduct, evaluation, and documentation of this study are designed to ensure that Celgene, its authorized representative, and Investigator abide by Good Clinical Practice (GCP), as described in International Conference on Harmonisation (ICH) Guideline E6 and in accordance with the general ethical principles outlined in the Declaration of Helsinki. The study will receive approval from an IRB/EC prior to commencement. The Investigator will conduct all aspects of this study in accordance with applicable national, state, and local laws of the pertinent regulatory authorities.

13.2. Investigator Responsibilities

Investigator responsibilities are set out in the ICH Guideline for Good Clinical Practice and in the local regulations. Celgene staff or an authorized representative will evaluate and approve all Investigators who in turn will select their staff.

The Investigator should ensure that all persons assisting with the study are adequately informed about the protocol, amendments, study treatments, as well as study-related duties and functions, including obligations of confidentiality of Celgene information. The Investigator should maintain a list of Sub-investigators and other appropriately qualified persons to whom he or she has delegated significant study-related duties.

The Investigator is responsible for keeping a record of all subjects who sign an informed consent document (ICD) and are screened for entry into the study. Subjects who fail screening must have the reason(s) recorded in the subject's source documents.

The Investigator, or a designated member of the Investigator's staff, must be available during monitoring visits to review data, resolve queries and allow direct access to subject records (eg, medical records, office charts, hospital charts, and study-related charts) for source data verification. The Investigator must ensure timely and accurate completion of CRFs and queries.

The information contained in the protocol and amendments (with the exception of the information provided by Celgene on public registry websites) is considered Celgene confidential information. Only information that is previously disclosed by Celgene on a public registry website may be freely disclosed by the Investigator or its institution, or as outlined in the Clinical Trial Agreement. Celgene protocol, amendment and IB information is not to be made publicly available (for example on the Investigator's or their institution's website) without express written approval from Celgene. Information proposed for posting on the Investigator's or their institution's website must be submitted to Celgene for review and approval, providing at least 5 business days for review.

At the time results of this study are made available to the public, Celgene will provide Investigators with a summary of the results that is written for the lay person. The Investigator is responsible for sharing these results with the subject and/or their caregiver as agreed by the subject.

13.3. Subject Information and Informed Consent

The Investigator must obtain informed consent of a subject and/or a subject's legal representative prior to any study related procedures.

Documentation that informed consent occurred prior to the study subject's entry into the study and of the informed consent process should be recorded in the study subject's source documents including the date. The original ICD signed and dated by the study subject and by the person consenting the study subject prior to the study subject's entry into the study, must be maintained in the Investigator's study files and a copy given to the study subject. In addition, if a protocol is amended and it impacts on the content of the informed consent, the ICD must be revised. Study subjects participating in the study when the amended protocol is implemented must be re-consented with the revised version of the ICD. The revised ICD signed and dated by the study subject and by the person consenting the study subject must be maintained in the Investigator's study files and a copy given to the study subject.

13.4. Confidentiality

Celgene affirms the subject's right to protection against invasion of privacy and to be in compliance with ICH and other local regulations (whichever is most stringent). Celgene requires the Investigator to permit Celgene's representatives and, when necessary, representatives from regulatory authorities, to review and/or copy any medical records relevant to the study in accordance with local laws.

Should direct access to medical records require a waiver or authorization separate from the subject's signed ICD, it is the responsibility of the Investigator to obtain such permission in writing from the appropriate individual.

13.5. Protocol Amendments

Any amendment to this protocol must be approved by the Celgene Clinical Research Physician/Medical Monitor. Amendments will be submitted to the IRB/EC for written approval. Written approval must be obtained before implementation of the amended version occurs. The written signed approval from the IRB/EC should specifically reference the Investigator name, protocol number, study title and amendment number(s) that is applicable. Amendments that are administrative in nature do not require IRB/IEC approval but will be submitted to the IRB/IEC for information purposes.

13.6. Institutional Review Board/Independent Ethics Committee Review and Approval

Before the start of the study, the study protocol, ICD, and any other appropriate documents will be submitted to the IRB/EC with a cover letter or a form listing the documents submitted, their dates of issue, and the site (or region or area of jurisdiction, as applicable) for which approval is sought. If applicable, the documents will also be submitted to the authorities in accordance with local legal requirements.

IP can only be supplied to an Investigator by Celgene or its authorized representative after documentation on all ethical and legal requirements for starting the study has been received by

Celgene or its authorized representative. This documentation must also include a list of the members of the IRB/EC and their occupation and qualifications. If the IRB/EC will not disclose the names, occupations and qualifications of the committee members, it should be asked to issue a statement confirming that the composition of the committee is in accordance with GCP. For example, the IRB General Assurance Number may be accepted as a substitute for this list. Formal approval by the IRB/EC should mention the protocol title, number, amendment number (if applicable), study site (or region or area of jurisdiction, as applicable), and any other documents reviewed. It must mention the date on which the decision was made and must be officially signed by a committee member. Before the first subject is enrolled in the study, all ethical and legal requirements must be met.

The IRB/EC and, if applicable, the authorities, must be informed of all subsequent protocol amendments in accordance with local legal requirements. Amendments must be evaluated to determine whether formal approval must be sought and whether the ICD should also be revised.

The Investigator must keep a record of all communication with the IRB/EC and, if applicable, between a Coordinating Investigator and the IRB/EC. This statement also applies to any communication between the Investigator (or Coordinating Investigator, if applicable) and regulatory authorities.

Any advertisements used to recruit subjects for the study must be reviewed by Celgene and the IRB/EC prior to use.

13.7. Ongoing Information for Institutional Review Board/ Ethics Committee

If required by legislation or the IRB/EC, the Investigator must submit to the IRB/EC:

- Information on serious or unexpected adverse events as soon as possible;
- Periodic reports on the progress of the study;
- Deviations from the protocol or anything that may involve added risk to subjects.

13.8. Termination of the Study

Celgene reserves the right to terminate this study prematurely at any time for reasonable medical or administrative reasons. Any premature discontinuation will be appropriately documented according to local requirements (eg, IRB/EC, regulatory authorities, etc).

In addition, the Investigator or Celgene has the right to discontinue a single site at any time during the study for medical or administrative reasons such as:

- Unsatisfactory enrollment;
- GCP noncompliance;
- Inaccurate or incomplete data collection;
- Falsification of records;
- Failure to adhere to the study protocol.

14. DATA HANDLING AND RECORDKEEPING

14.1. Data/Documents

The Investigator must ensure that the records and documents pertaining to the conduct of the study and the distribution of the investigational product are complete, accurate, filed and retained. Examples of source documents include: hospital records; clinic and office charts; laboratory notes; memoranda; subject's diaries or evaluation checklists; dispensing records; recorded data from automated instruments; copies or transcriptions certified after verification as being accurate copies; microfiche; x-ray film and reports; and records kept at the pharmacy, and the laboratories, as well as copies of CRFs or CD-ROM.

14.2. Data Management

Data will be collected via CRF and entered into the clinical database per Celgene SOPs. This data will be electronically verified through use of programmed edit checks specified by the clinical team. Discrepancies in the data will be brought to the attention of the clinical team, and investigational site personnel, if necessary. Resolutions to these issues will be reflected in the database. An audit trail within the system will track all changes made to the data.

14.3. Record Retention

Essential documents must be retained by the Investigator according to the period of time outlined in the clinical trial agreement. The Investigator must retain these documents for the time period described above or according to local laws or requirements, whichever is longer. Essential documents include, but are not limited to, the following:

- Signed ICDs for all subjects;
- Subject identification code list, screening log (if applicable), and enrollment log;
- Record of all communications between the Investigator and the IRB/EC;
- Composition of the IRB/EC;
- Record of all communications between the Investigator, Celgene, and their authorized representative(s);
- List of Sub-investigators and other appropriately qualified persons to whom the Investigator has delegated significant study-related duties, together with their roles in the study, curriculum vitae, and their signatures;
- Copies of CRFs (if paper) and of documentation of corrections for all subjects;
- CC-90010 accountability records;
- Record of any body fluids or tissue samples retained;
- All other source documents (subject records, hospital records, laboratory records, etc.);
- All other documents as listed in Section 8 of the ICH consolidated guideline on GCP (Essential Documents for the Conduct of a Clinical Trial).

The Investigator must notify Celgene if he/she wishes to assign the essential documents to someone else, remove them to another location or is unable to retain them for a specified period. The Investigator must obtain approval in writing from Celgene prior to destruction of any records. If the Investigator is unable to meet this obligation, the Investigator must ask Celgene for permission to make alternative arrangements. Details of these arrangements should be documented.

All study documents should be made available if required by relevant health authorities. Investigator or institution should take measures to prevent accidental or premature destruction of these documents.

EDMS Doc. Number: [redacted]

15. QUALITY CONTROL AND QUALITY ASSURANCE

All aspects of the study will be carefully monitored by Celgene or its authorized representative for compliance with applicable government regulations with respect to current GCP and SOPs.

15.1. Study Monitoring and Source Data Verification

Celgene ensures that appropriate monitoring procedures are performed before, during and after the study. All aspects of the study are reviewed with the Investigator and the staff at a study initiation visit and/or at an Investigators' Meeting. Prior to enrolling subjects into the study, a Celgene representative will review the protocol, CRFs, procedures for obtaining informed consent, record keeping, and reporting of AEs/SAEs with the Investigator. Monitoring will include on-site visits with the Investigator and his/her staff as well as any appropriate communications by mail, email, fax, or telephone. During monitoring visits, the facilities, investigational product storage area, CRFs, subject's source documents, and all other study documentation will be inspected/reviewed by the Celgene representative in accordance with the Study Monitoring Plan.

Accuracy will be checked by performing source data verification that is a direct comparison of the entries made onto the CRFs against the appropriate source documentation. Any resulting discrepancies will be reviewed with the Investigator and/or his/her staff. Any necessary corrections will be made directly to the CRFs or via queries by the Investigator and/or his/her staff. Monitoring procedures require that informed consents, adherence to inclusion/exclusion criteria and documentation of SAEs and their proper recording be verified. Additional monitoring activities may be outlined in a study-specific monitoring plan.

15.2. Audits and Inspections

In addition to the routine monitoring procedures, a Good Clinical Practice Quality Assurance unit exists within Celgene. Representatives of this unit will conduct audits of clinical research activities in accordance with Celgene SOPs to evaluate compliance with Good Clinical Practice guidelines and regulations.

The Investigator is required to permit direct access to the facilities where the study took place, source documents, CRFs and applicable supporting records of study subject participation for audits and inspections by IRB/ECs, regulatory authorities (eg, FDA, EMA, Health Canada) and company authorized representatives. The Investigator should make every effort to be available for the audits and/or inspections. If the Investigator is contacted by any regulatory authority regarding an inspection, he/she should contact Celgene immediately.

16. PUBLICATIONS

As described in Section 13.2, all protocol- and amendment-related information, with the exception of the information provided by Celgene on public registry websites, is considered Celgene confidential information and is not to be used in any publications. Celgene protocol-related information proposed for use in a publication must be submitted to Celgene for review and approval, and should not be utilized in a publication without express written approval from Celgene, or as described in the Clinical Trial Agreement.

Celgene will ensure Celgene-sponsored studies are considered for publication in the scientific literature in a peer-reviewed journal, irrespective of the results. At a minimum, this applies to results from all Phase 3 clinical studies, and any other study results of significant medical importance. This also includes results relating to investigational medicines whose development programs have been discontinued.

Study results may also be presented at one or more medical congresses, and may be used for scientific exchange and teaching purposes. Additionally, this study and its results may be submitted for inclusion in all appropriate health authority study registries, as well as publication on health authority study registry websites, as required by local health authority regulations.

Eligibility for external authorship, as well as selection of first authorship, will be based on several considerations, including, but not limited to, contribution to protocol development, study recruitment, data quality, participation in data analysis, participation in study steering committee (when applicable) and contribution to abstract, presentation and/or publication development.

17. REFERENCES

Abedin S, Boddy C, Munshi H. BET inhibitors in the treatment of hematologic malignancies: current insights and future prospects. OncoTargets and Therapy 2016 Sep 28;9: 5943-5953.

Abramson J, Blum K, Flinn I, Gutierrez M, Goy A, Maris M, et al. BET inhibitor CPI-0610 is well tolerated and induces responses in diffuse large B-cell lymphoma and follicular lymphoma: preliminary analysis of an ongoing Phase 1 study [abstract]. ASH 2015; Abstract 1491.

Alcedo J, Ayzenzon M, Von Ohlen T, Noll M, Hooper J. The Drosophila smoothened Gene encodes a seven-pass membrane protein, a putative receptor for the Hedgehog signal. Cell 1996;86:221-232.

Alonso V, Revert A, Monteagudo C, Martin JM, Insa A, Pinazo I, et al. Basal cell carcinoma with distant multiple metastases to the vertebral column. JEADV 2006;20:735-767.

Amakye D, Jagani Z, Dorsch M. Unraveling the therapeutic potential of the Hedgehog pathway in cancer. Nature Med 2013;19(11):1410-1422.

American Cancer Society: Skin Cancer: Basal and Squamous Cell. 2015.

Amorim S, Stathis A, Gleeson M, Iyengar S, Magarotto V, Leleu X, et al. Bromodomain inhibitor OTX015 in patients with lymphoma or multiple myeloma: a dose-escalation, open label, pharmacokinetic, phase 1 study. Lancet Haematol. 2016;3(4):e196-204.

Arrowsmith C, Bountra C, Fish P, Lee K, Schapira M. Epigenetic protein families: a new frontier for drug discovery. Nat Rev Drug Discov 2012;11:384-400.

Atwood S, Sarin K, Oro A, Tang J. Smoothened variants explain the majority of drug resistance in basal cell carcinoma. Cancer Cell 2015;27:342-353.

Babb J, Rogatko A, Zacks S. Cancer phase I clinical trials: efficient dose escalation with overdose control. Stat Med 1998;17(10):1103-20.

Bandopadhayay P, Bergthold G, Nguyen B, Schubert S, Gholamin S, Tang Y, et al. BET bromodomain inhibition of MYC-amplified medulloblastoma. Clin Cancer Res 2014;20(4):912-25.

Basset-Seguin N, Hauschild A, Grob JJ, Kuntsfeld R, Dreno B, Mortier L, et al. Vismodegib in patients with advanced basal cell carcinoma (STEVIE): a pre-planned interim analysis of an international, open-label trial. Lancet Oncol 2015;16:729-36.

Benson 3rd AB, Ajani JA, Catalano RB, Engelking C, Kornblau SM, Martenson Jr JA, et al. Recommended guidelines for the treatment of cancer treatment-induced diarrhea. J Clin Oncol 2004;22:2918-2926.

Berthon C, Raffoux E, Thomas X, Vey N, Gomez-Roca C, Yee K, et al. Bromodomain inhibitor OTX015 in patients with acute leukaemia: a dose-escalation, phase 1 study. Lancet Haematol. 2016;3(4):e186-95.

Biehs B, Dijkgraaf G, Piskol R, Alicke B, Boumahdi S, Peale F. A cell identity switch allows residual BCC to survive Hedgehog pathway inhibition. Nature 2018 Oct;562(7727):429-433.

Campbell L, Polyak K. Breast tumor heterogeneity: cancer stem cells or clonal evolution? Cell Cycle 2007;6(19):2332-2338.

Castellanos J, Merchant N, Nagathihalli N. Emerging targets in pancreatic cancer: epithelial-mesenchymal transition and cancer stem cells. OncoTargets Ther 2013;6:1261-7.

Chaidos A, Caputo V, Karadimitris A. Inhibition of bromodomain and extra-terminal proteins (BET) as a potential therapeutic approach in haematological malignancies: emerging preclinical and clinical evidence. Ther Adv Hematol 2015;6(3):128-41.

Cheson BD, Fisher RI, Barrington SF, Cavalli F, Schwartz L, Zucca E, et al. Recommendations for Initial Evaluation, Staging, and Response Assessment of Hodgkin and Non-Hodgkin Lymphoma: The Lugano Classification. J Clin Oncol 2014;32(27):3059-3067.

Cheson BD, Pfistner B, Juweid M, Gascoyne R, Specht L, Horning S, et al. Revised response criteria for malignant lymphoma. J Clin Oncol 2007;25(5):579-586.

Chi P, Allis C, Wang G. Covalent histone modifications-miswritten, misinterpreted and mis-erased in human cancers. Nat Rev Cancer 2010;10(7):457-69.

Coiffier B, Thieblemont C, Van Den Neste E, Lepeu G, Plantier I, Castaigne S, et al. Long-term outcome of patients in the LNH-98.5 trial, the first randomized study comparing rituximab-CHOP to standard CHOP chemotherapy in DLBCL patients: a study by the Groupe d'Etudes des Lymphomes de l'Adulte. Blood. 2010 Sep 23;116(12):2040-5.

Danial C, Lingala B, Balise R, Oro AE, Reddy S, Colevas A, Chang ALS. Markedly improved overall survival in 10 consecutive patients with metastatic basal cell carcinoma. BJD 2013;169:673-676.

Dahmane N, Lee J, Robins P, Heller P, Ruiz A. Activation of the transcription factor Gli1 and the sonic Hedgehog signalling pathway in skin tumours. Nature 1997;389:876-880.

Dawood S, Austin L, Cristofanilli M. Cancer stem cells: implications for cancer therapy. Oncology J 2014;28(2):1101-7.

Dawson M, Kouzarides T, Huntly B. Targeting epigenetic readers in cancer. N Engl J Med 2012;367:647-57.

Delmore JE, Issa G, Lemieux M, Rahl P, Shi J, Jacobs H, et al. BET bromodomain inhibition as a therapeutic strategy to target c-Myc. Cell. 2011 Sep 16;146(6):904-17.

Dombret H, Preudhomme C, Berthon C, Raffoux E, Thomas X, Vey N, et al. A phase I study of the BET-bromodomain inhibitor OTX015 in patients with advanced acute leukemia [abstract]. ASH 2014; Abstract 117.

European Medicines Agency (EMA), Committee for Medicinal Products for Human Use (CHMP): Guideline on clinical trials in small populations. July 2006.

European Medicines Agency (EMA), Committee for Medicinal Products for Human Use (CHMP): Guideline on the investigation of drug interactions. June 2012.

Eisenhauer EA, Therasse P, Bogaerts J, Schwartz LH, Sargent D, Ford R, et al. New response evaluation criteria in solid tumours: revised RECIST guideline (Version 1.1). Eur J Cancer 2009;45(2):228-247.

Eppert K, Takenaka K, Lechman E, Waldron L, Nilsson B, van Galen P, et al. Stem cell gene expression programs influence clinical outcome in human leukemia. Nature Med 2011;17(9):1086-1094.

Epstein E. Basal cell carcinomas: attack of the hedgehog. Nature Rev 2008;8:743-754.

Erivedge (vismodegib) [European Public Assessment Report]. Grenzach-Wyhlen, Germany: Roche Pharma AG; 2015. Available from: http://www.ema.europa.eu/ema/index.jsp?curl=pages/medicines/human/medicines/002602/human_med_001659.jsp&mid=WC0b01ac058001d124

Fang D, Nguyen T, Leishear K, Finko R, Kulp A, Hoyz S, et al. A tumorigenic subpopulation with stem cell properties in melanomas. Cancer Res 2005;65(20):9328-9337.

Food and Drug Administration (FDA). Guidance for industry: food effect bioavailability and fed bioequivalence studies. Dec 2002.

Filippakopoulos P, Qi J, Picaud S, Shen Y, Smith W, Fedorov O, et al. Selective inhibition of BET bromodomains. Nature 2010;468:1067-1073.

Filippakopoulos P, Knapp S. Targeting bromodomains: epigenetic readers of lysine acetylation. Nature Reviews 2014;13:337-356.

French C. NUT midline carcinoma. Cancer Genetics and Cytogenetics 2010;203:16-20.

Gailani M, Stahle-Backdahl M, Leffell D, Glynn M, Zaphiropoulus P, Pressman C, et al. The role of the human homologue of Drosophila patched in sporadic basal cell carcinomas. Nature genetics 1996;14:78-81.

Goldberg PA, Roussel MG, Inzucchi SE. Clinical results of an updated insulin infusion protocol in critically ill patients. Diabetes Spectrum 2005;18:188-91.

Hahn H, Wicking C, Zaphiropoulos P, Gailani M, Shanley S, Chidambaram A, et al. Mutations of the Human Homolog of Drosophila patched in the Nevoid Basal Cell Carcinoma Syndrome. Cell 1996;85:841-851.

Haycraft C, Banizs B, Aydin-Son Y, Zhang Q, Michaud E, Yoder B. Gli2 and Gli3 Localize to Cilia and Require the Intraflagellar Transport Protein Polaris for Processing and Function. PloS Genetics 2005 ;1(4) :048-0488.

Herait P, Dombret H, Thieblemont C, Facon T, Stathis A, Cunningham D, et al. BET-bromodomain (BRD) inhibitor OTX015: final results of the dose-finding part of a Phase 1 study in hematologic malignancies. Annals of Oncology 2015;26(Supplement 2):ii10-ii11.

Hu Y, Zhou J, Ye F, Xiong H, Peng L, Zheng Z, et al. BRD4 inhibitor inhibits colorectal cancer growth and metastasis. Int J Mol Sci 2015;16:1928-48.

Huangfu D, Anderson K. Cilia and Hedgehog responsiveness in the mouse. PNAS 2005;102(32):11325-11330.

Huber M, Kraut N, Beug H. Molecular requirements for epithelial-mesenchymal transition during tumor progression. Curr Opin Cell Biol 2005;17:548-58.

ICH Harmonised Tripartite Guideline S9: Nonclinical evaluation for anticancer pharmaceuticals. Current Step 4 version dated 29 October 2009.

Infante P, Alfonsi R, Botta B, Mori M, Marcotullio L. Targeting GLI factors to inhibit the Hedgehog pathway. Trends in Pharma Sci 2015;36(8):547-558.

Ingham P, McMahon A. Hedgehog signaling in animal development: paradigms and principles. Genes & Dev 2001;15:3059-3087.

Itti E, Meignan M, Berriolo-Riedinger A, Biggi A, Cashen AF, Vera P, et al. An international confirmatory study of the prognostic value of early PET/CT in diffuse large B-cell lymphoma: comparison between Deauville criteria and DeltaSUVmax. Eur J Nucl Med Mol Imaging. 2013 Sep;40(9):1312-20.

Ivy SP, Siu LL, Garrett-Mayer E, Rubinstein L. Approaches to Phase 1 clinical trial design focused on safety, efficiency, and selected patient populations: a report from the clinical trial design task force of the National Cancer Institute Investigational Drug Steering Committee. Clin Cancer Res 2010;16(6):1726-36.

Jacobson R, Ladurner A, King D, Tjian R. Structure and function of a human TAFII250 double bromodomain module. Science 2000;288:1422-5.

Jenuwein T, Allis C. Translating the histone code. Science 2001;293:1074-80.

Johnson R, Rothman A, Xie J, Goodrich L, Bare J, Bonifas J, et al. Human Homolog of patched, a candidate gene for the basal cell nevus syndrome. Science 1996;272:1668-1671.

Jung K, Das A, Chai J, Kim S, Morya N, Park K, et al. Rna sequencing reveals mechanisms underlying BET inhibitor JQ1-medidated modulation of the LPS-induced activation of BV-2 microglial cells. J Neuroinflammation 2015;12(36):1-18.

Junwei S, Vakoc C. The mechanisms behind the therapeutic activity of BET bromodomain inhibition. Molecular Cell 2014;54:728-736.

Lathia J, Mack S, Mulkearns-Hubert E, Valentim C, Rich J. Cancer stem cells in glioblastoma. Genes & Devel 2015;29:1203-1217.

Li X, Lewis M, Huang J, Gutierrez C, Osborne K, Wu M, et al. Intrinsic resistance of tumorigenic breast cancer cells to chemotherapy. J Natl Cancer Inst 2008;100:672-679.

Li G, Guo W, Zhang Y, Seng J, Zhang H, Ma X, et al. Suppression of BRD4 inhibits human hepatocellular carcinoma by repressing MYC and enhancing BIM expression. Oncotarget 2015;7(3):2462-2474.

Liu A, Wang B, Niswander L. Mouse intraflagellar transport proteins regulate both the activator and repressor functions of Gli transcription factors. Development 2005;132:3103-3111.

Lockwood WW, Zejnullahu K, Bradner JE, Varmus H. Sensitivity of human lung adenocarcinoma cell lines to targeted inhibition of BET epigenetic signaling proteins. PNAS 2012;109(47):19408-19413.

Lomas A, Leonardi-Bee J, Bath-Hextall F. A systematic review of worldwide incidence of nonmelanoma skin cancer. BJD 2012; 166: 1069-1080.

Long J, Bin L, Jezabel R, Pastori C, Volmar C, Wahlestedt C, et al. The BET bromodomain inhibitor I-BET151 acts downstream of smoothened protein to abrogate the growth of hedgehog protein-driven cancers. J Biol Chem 2014;289(51):35494-35502.

LoRusso P, Rudin C, Reddy J, Tibes R, Weiss G, Borad M, et al. Phase I trial of Hedgehog pathway inhibitor Vismodgeib (GDC-0449) in patients with refractory, locally advanced or metastatic solid tumors. Cancer Res 2011;17(8):2502-2511.

Lovén J, Hoke H, Lin C, Lau A, Orlando D, Vakoc C, et al. Selective inhibition of tumor oncogenes by disruption of super-enhancers. Cell 2013;153:320-34.

Ma S, Chan K-W, Hu L, Lee T, Wo J, Ng I, et al. Identification and characterization of tumorigenic liver cancer stem/progenitor cells. Gastroenterology 2007;132:2542-2556.

Mani S, Guo W, Liao M-J, Eaton E, Ayyanan A, Zhou A, et al. The epithelial-mesenchymal transition generates cells with properties of stem cells. Cell 2008;133:704-15.

Mazur P, Herner A, Mello S, Wirth M, Hausmann S, Sanchez-Rivera F, et al. Combined inhibition of BET family proteins and histone deacetylases as a potential epigenetics-based therapy for pancreatic ductal adenocarcinoma. Nat Med 2015;21(10):1163-71.

Meignan M, Barrington S, Itti E, Gallamini A, Haioun C, Polliack A. Report on the 4th International Workshop on Positron Emission Tomography in Lymphoma held in Menton, France, 3-5 October 2012. Leuk Lymphoma. 2014 Jan;55(1):31-7.

Mertz JA, Conery AR, Bryant BM, Sandy P, Balasubramanian S, Mele D, et al. Targeting MYC dependence in cancer by inhibiting BET bromodomains. PNAS 2011;108(40):16669-74.

Miller AB, Hoogstraten B, Staquet M, Winkler A. Reporting results of cancer treatment. Cancer 1981;47:207-214.

Migden M, Guminski A, Gutzmer R, Dirix L, Lewis K, Combemale P, Her R, et al. Treatment with two different doses of sonidegib in patients with locally advanced or metastatic basal cell carcinoma (BOLT): a multicentre, randomized, double-blind phase 2 trial. Lancet Oncol 2015a;16:716-728.

Migden M, Gutzmer R, Yi T, Gogov S, Lear J, Dummer R. A 12-month update of BOLT: a phase 2 randomized, double-blind study of sonidegib (LDE225) in patients with locally advanced or metastatic basal cell carcinoma. J Am Acad Dermatol 2015b.

Mottok A, Gascoyne R. Bromodomain Inhibition in Diffuse Large B-cell Lymphoma-Giving MYC a Brake. Clin Cancer Res; 2015 Jan 1; 21(1):4-6

Neuenschwander B, Branson M, Gsponer T. Critical aspects of the Bayesian approach to phase I cancer trials. Stat Med 2008;27(13);2420-39.

Neuenschwander B, Matano A, Tang Z, Wandel S, Roychoudhury S, Bailey S. A Bayesian Industry Approach to Phase I Combination Trials in Oncology. In: Statistical Methods in Drug Combination Studies, Boca Raton, FL: Chapman & Hall/CRC Press. Edited by Zhao, W. and Yang, H. 2015.

Nilsson J, Cleveland J. Myc pathways provoking cell suicide and cancer. Oncogene 2003;22:9007-21.

Nolan-Stevaux O, Lau J, Truitt M, Chu G, Hebrok M. GLI1 is regulated through Smoothened-independent mechanisms in neoplastic pancreatic ducts and mediates PDAC cell survival and transformation. Genes & Dev 2009;23:24-36.

Odomzo (sonidegib) [European Public Assessment Report]. Nuremberg, Germany: Novartis Pharma GmbH; 2015. Available from: http://www.ema.europa.eu/ema/index.jsp?curl=pages/medicines/human/medicines/002839/human_med_001897.jsp&mid=WC0b01ac058001d124

Oken M, Creech R, Tormey D, Horton J, Davis T, McFadden E, et al. Toxicity and response criteria of the Eastern Cooperative Group. Am J Clin Oncol 1982;5:649-655.

Oliver T, Grasfeder L, Carroll A, Kaiser C, Gillingham C, Lin S, et al. Transcriptional profiling of the Sonic hedgehog response: A critical role for N-myc in proliferation of neuronal precursors. PNAS 2003;100(12);7331-7336.

Ott G, Rosenwald A, Campo E. Understanding MYC driven aggressive B-cell lymphomas: pathogenesis and classification. Blood. 2013 Dec 5;122(24):3884-91.

Ott G. Impact of MYC on malignant behavior. Hematology Am Soc Hematol Educ Program. 2014 Dec 5;2014(1):100-6.

Pastori C, Daniel M, Penas C, Volmar C-H, Johnstone A, Brothers S, et al. BET bromodomain proteins are required for glioblastoma cell proliferation. Epigenetics 2014;9(4):611-20.

Puissant A, Frumm S, Alexe G, Bassil C, Qi J, Chanthery Y, et al. Targeting MYCN in neuroblastoma by BET bromodomain inhibition. Cancer Discov 2013;3(3):308-23.

Regl G, Neill G, Eichberger T, Kasper M, Ikram M, Koller J, et al. Human Gli2 and Gli1 are part of a positive feedback mechanism in Basal cell carcinoma. Oncogene 2002;21:5529-5539.

Reifenberger J, Wolter M, Knobbe C, Kohler B, Schonicke A, Scharwachter C, et al. Somatic mutations in the PTCH, SMOH, SUFUH and TP53 genes in sporadic basal cell carcinomas. BJD 2005;152:43-51.

Ridky T, Cotsarelis G. Vismodegib resistance in basal cell carcinoma: Not a smooth fit. Cancer Cell 2015;27:315-316.

Rogatko A, Schoeneck D, Jonas W, Tighiouart M, Khuri F, Porter A. Translation of innovative designs into Phase I trials. J Clin Onc 2007;25(31):4982-4986.

Rubin A, Chen E, Ratner D. Current Concepts Basal-Cell Carcinoma. N Engl J Med 2005; 353:2262-2269.

Satoh K, Hamada S, Shimosegawa T. Involvement of epithelial to mesenchymal transition in the development of pancreatic ductal adenocarcinoma. J Gastroenterol 2015;50:140-6.

Scales S, de Sauvage F. Mechanism of Hedgehog pathway activation in cancer and implications for therapy. Trends in Pharmacological Sciences 2009;30(6):303-312

Sekulic A, Migden M, Oro A, Dirix L, Lewis K, Hainsworth J, et al. Efficacy and Safety of Vismodegib in Advanced Basal-cell Carcinoma. N Engl J Med 2012;366;2171-9.

Sharpe H, Pau G, Yauch R, de Sauvage F. Genomic Analysis of Smoothened inhibitor resistance in basal cell carcinoma. Cancer Cell 2015;27:327-341.

Sheridan C, Kishimoto H, Ruchs R, Mehrotra S, Bhat-Nakshatri P, Turner C, et al. CD44+/CD24- breast cancer cells exhibit enhanced invasive properties: an early step necessary for metastasis. Breast Cancer Res 2006;8(5):R59.

Shi J, Wang Y, Zeng L, Wu Y, Deng J, Zhang Q, et al. Disrupting the interaction of BRD4 with diacetylated Twist suppresses tumorigenesis in basal-like breast cancer. Cancer Cell 2014;25: 210-225.

Shimamura T, Chen Z, Soucheray M, Carretero J, Kikuchi E, Tchaicha J, et al. Efficacy of BET bromodomain inhibition in Kras-mutant non-small cell lung cancer. Clin Cancer Res 2013;19(22):6183-92.

Tang C, Ang B, Pervaiz S. Cancer stem cell: target for anti-cancer therapy. FASEB J 2007;21:3777-3785.

Tang Y, Gholamin S, Schubert S, Willardson MI, Lee A, Bandopadhayay P, et al. Epigenetic targeting of Hedgehog pathway transcriptional output through BET bromodomain inhibition. Nature 2014;20(7):732-740.

Teglund S, Toftgard R. Hedgehog beyond medulloblastoma and basal cell carcinoma. Biochim Biophys Acta 2010;1805:181-208.

Thieblemont C, Stathis A, Inghirami G, Karlin L, Morschhauser F, Gleeson M, et al. A Phase 1 study of the BET-bromodomain inhibitor OTX015 in patients with non-leukemic hematologic malignancies [abstract]. ASH 2014; Abstract 4417.

Thiery J. Epithelial-mesenchymal transitions in tumour progression. Nat Rev Cancer 2002;2(6):442-54.

Thiery J. Epithelial-mesenchymal transitions in development and pathologies. Curr Opin Cell Biol 2003;15:740-6.

Tourneau CL, Gan HK, Razak ARA, Paoletti X. Efficiency of new dose escalation designs in dose-finding Phase I trials of molecularly targeted agents. PloS ONE 2012;7(12):e51039.

Tourneau CL, Lee JJ, Siu LL. Dose Escalation Methods in Phase I cancer clinical trials. J Natl Cancer Inst 2009;101(10):708-720.

Trakatelli M, et al. Guideline on the Treatment of Basal Cell Carcinoma. European Dermatology Forum. 2014.

Turina M, Christ-Crain M, Polk HC. Diabetes and hyperglycemia: strict glycemic control. Crit Care Med 2006;34(9 Suppl):S291-300.

Unden A, Zhaphiropoulos, Bruce K, Toftgard R, Stahle-Backdahl M. Human patched (PTCH) mRNA is Overexpressed consistently in tumor cells of both Familial and Sporadic Basal Cell Carcinoma. Cancer Research 1997;57:2336-2340.

U.S. Department of Health and Human Services, Food and Drug Administration (FDA), Center for Drug Evaluation and Research (CDER). Guidance for Industry: Estimating the maximum safe starting dose in initial clinical trials for therapeutics in adult healthy volunteers. July 2005.

Varnat F, Duquet A, Malerba M, Zbinden M, Mas C, Gervaz P, Altaba A. Human colon cancer epithelial cells harbour active HEDGEHOG-GLI signalling that is essential for tumour growth, recurrence, metastasis and stem cell survival and expansion. EMBO Mol Med 2009;1:338-51.

Von Burstin J, Eser S, Paul M, Seidler B, Brandl M, Messe M, et al. E-Cadherin regulates metastasis of pancreatic cancer in vivo and is suppressed by a SNAIL/HDAC1/HDAC2 repressor complex. http://www.ncbi.nlm.nih.gov/pubmed/19362090 Gastroenterology 2009;137:361-371.

Von Hoff D, LoRusso P, Rudin C, Reddy J, Yauch R, Tibes R, et al. Inhibition of the Hedgehog Pathway in Advanced Basal-cell carcinoma. New Engl J Med 2009;361:1164-1172.

Wang L, Wu X, Wang R, Yang C, Zhi L, Wang C, et al. BRD4 inhibition suppresses cell growth, migration and invasion of salivary adenoid cystic carcinoma. Biol Res. 2017;50(19)

Wen P, Macdonald D, Reardon D, Cloughesy T, Sorensen G, Galanis E, et al. Updated Response Assessment Criteria for High-grade Gliomas: Response Assessment in Neuro-Oncology Working Group. J Clin Oncol 2010;28(11):1963-1972.

Wong C, Strange R, Lear J. Basal cell carcinoma. BJM 2003;327:794-798.

World Health Organization. Ultraviolet radiation and the INTERSUN Programme. 2015.

Wyce A, Degenhardt Y, Bai Y, Le B, Korenchuk S, Crouthame M, McHugh C, Vessella R, Creasy C, Tummino P, Barbash O. Inhibition of BET bromodomain proteins as a therapeutic approach in prostate cancer. Oncotarget 2013a;4(12):2419-29.

Wyce A, Ganji G, Smitheman K, Chung C, Korenchuk S, Barbash B, et al. BET inhibition silences expression of MYCN and BCL2 and induces cytotoxicity in neuroblastoma tumor models. PloS One 2013b;8(8):e72967.

Whyte W, Orlando D, Hnisz D, Abraham B, Lin C, Kagey M, et al. Master transcription factors and mediator establish super-enhancers at key cell identity genes. Cell 2013;153:307-319.

Wu T, Donohoe M. The converging roles of BRD4 and gene transcription in pluripotency and oncogenesis. RNA Dis 2016 ;2(3) :1-7.

Xie J, Murone M, Luoh S, Ryan A, Gu Q, Zhang C, et al. Activating Smoothened mutations in sporadic basal-cell carcinoma. Nature 1998;391:90-92.

Zhu Y, Luo M, Brooks M, Clouthier S, Wicha M. Biological and clinical significance of cancer stem cell plasticity. Clinical and Translational Medicine 2014;32:1-11.

Zuber J, Shi J, Wang E, Rappaport A, Herrmann H, Sison E, et al. RNAi screen identifies Brd4 as a therapeutic target in acute myeloid leukaemia. Nature 2011;478:524-8.

18. APPENDICES

Appendix A: Table of Abbreviations

Table 16: Table of Abbreviations

Abbreviation or Specialist Term	Explanation
ABC	Activated B-cell-like
ADA	Anti-drug antibodies
ADCC	Antibody-dependent cellular cytotoxicity
ADL	Activity of daily life
AE	Adverse event
ALL	Acute lymphoid leukemia
ALT	Alanine aminotransferase (SGPT)
AML	Acute myeloid leukemia
ANC	Absolute neutrophil count
Ara-C	Cytarabine
AST	Aspartate aminotransferase (SGOT)
AUC	Area under the curve
AUC_{last}	Area under the plasma concentration-time curve from time zero to the time of the last quantifiable concentration
AUC_{0-24}	Area under the plasma concentration-time curve calculated from time zero to 24 hours
β-hCG	β-subunit of human chorionic gonadotropin
BCC	Basal cell carcinoma
BET	Bromodomain and extra-terminal
BID	Twice a day
BLBC	Basal-like breast cancer
BLRM	Bayesian logistic regression model
BM	Bone marrow
BMI	Body mass index
BRD	Bromodomain
BRD4	Bromodomain-containing protein-4
BSA	Body surface area
BUN	Blood urea nitrogen

Table 16: Table of Abbreviations (Continued)

Abbreviation or Specialist Term	Explanation
C	Cycle
CBC	Complete blood count
CD	Cluster of differentiation
CEBPα	CCAAT/enhancer binding protein alpha
CI	Confidence interval
c-Kit	Mast/stem cell growth factor receptor
CL	Clearance
CL_{ss}/F	Apparent total clearance after multiple dosing
C_{max}	Maximum plasma concentration of drug
CNS	Central nervous system
CBR	Clinical benefit rate
CR	Complete remission
CRc	Cytogenetic complete remission
Cri	Complete remission with incomplete neutrophil recovery
CRp	Complete remission with incomplete platelet recovery
CRP	C-reactive protein
CRR	Complete remission rate
CRO	Contract research organization
CRF	Case report form
CRP	Clinical Research Physician
CRS	Clinical Research Scientist
CRT	Calreticulin
CSF	Cerebrospinal fluid
CSC	Cancer stem cells
CT	Computed tomography
CTCAE	Common Terminology Criteria for Adverse Events
CV%	Coefficient of variation
DAT	Direct antiglobulin test
DCR	Disease control rate
DIC	Disseminated intravascular coagulation

Table 16: Table of Abbreviations (Continued)

Abbreviation or Specialist Term	Explanation
DLT	Dose-limiting toxicity
DMC	Data Monitoring Committee
DOR	Duration of response
EC	Ethics Committee
ECG	Electrocardiogram
ECHO	Echocardiogram
ECOG PS	Eastern Cooperative Oncology Group Performance Status
eCRF	Electronic case report form
EEA	European Economic Area
ELISA	Enzyme-linked immunoassay
EMT	Epithelial-mesenchymal transition
EOI	End of infusion
EOT	End of treatment
ESR	Erythrocyte sedimentation rate
EWOC	Escalation with overdose control
FACS	Fluorescence-activated cell sorting
FCBP	Females of child bearing potential
FDA	Food and Drug Administration
FISH	Fluorescence in situ hybridization
FLT3	Fms-related tyrosine kinase 3
FLT3-ITD	Fms-related tyrosine kinase 3-internal tandem duplication
FOXP3	Forkhead box P3
GCP	Good Clinical Practice
GBM	Glioblastoma multiforme
GCB	Germinal center B-cell-like
GI	Gastrointestinal
GL1	Glioma-associated oncogene homolog 1
GVHD	Graft-versus-host disease
HBV	Hepatitis B virus
HCV	Hepatitis C virus

Table 16: Table of Abbreviations (Continued)

Abbreviation or Specialist Term	Explanation
HGB	Hemoglobin
Hh	Hedgehog
HIV	Human immunodeficiency virus
HLA	Human leukocyte antigen
HNSTD	Highest non-severely toxic dose
HSCT	Hematopoietic stem cell transplant
huCD	Human cluster of differentiation
IC_{50}	Half-maximal inhibitory concentration
ICD	Informed consent document
ICF	Informed consent form
ICH	International Council on Harmonisation
ICSH	International Council for Standardization in Hematology
IFN	Interferon
IgE	Immunoglobulin E subclass
IgG	Immunoglobulin G subclass
IL	Interleukin
IL-1β	Interleukin-1 beta
IND	Investigational New Drug
INR	International normalized ratio
IP	Investigational Product
IPSS-R	Revised International Prognostic Index Scoring System
IRB	Institutional Review Board
IRR	Infusion related reaction
IRT	Interactive Response Technology
IV	Intravenous
IVIG	Intravenous immunoglobulin
IWG	International working group
KC-GRO	Keratinocyte-derived cytokine-growth-regulated oncogene
LDH	Lactate dehydrogenase
LSC	Leukemia stem cell

Table 16: Table of Abbreviations (Continued)

Abbreviation or Specialist Term	Explanation
LVEF	Left ventricular ejection fraction
mCR	Molecular complete remission
MCP-1	Monocyte chemoattractant protein-1
MDR	Multi-drug resistance
MDS	Myelodysplastic syndrome
MedDRA	Medical Dictionary for Regulatory Activities
MGMT	O^6-methylguanine–DNA methyltransferase
MIP-1α	Macrophage inflammatory protein-1 alpha
MM	Multiple myeloma
MRI	Magnetic resonance imaging
MTD	Maximum tolerated dose
MUGA	Multi-gated acquisition
N	Number
NCI	National Cancer Institute
NHL	Non-Hodgkin's lymphoma
NOD-SCID	Non-obese diabetic, severe-combine immunodeficiency
NOAEL	No observed adverse effect level
NOEL	No observed effect level
NPM1	Nucleophosmin 1
NSG	Non-obese diabetic, severe-combine immunodeficiency gamma
NTD	Non-tolerated dose
NUT	Nuclear protein in testis
O_2	Oxygen
ORR	Objective response rate
OS	Overall survival
PBMC	Peripheral blood mononuclear cells
PCR	Polymerase chain reaction
PD	Pharmacodynamic
PDX	Patient-derived xenograft
PFS	Progression-free survival

Table 16: Table of Abbreviations (Continued)

Abbreviation or Specialist Term	Explanation
PK	Pharmacokinetics
PLT	Platelet
PMBL	Primary mediastinal B-cell lymphoma
PR	Partial remission
PT	Prothrombin time
PTT	Partial thromboplastin time
Q2W	Every two weeks
QD	Once a day
QW	Once weekly
QWx2	Once a week for two weeks
qRT-PCR	Quantitative reverse transcription polymerase chain reaction
QWx4	Once a week for four weeks
RAEB	Refractory anemia with excess blasts
RBC	Red blood cell count
RFS	Relapse free survival
RP2D	Recommended Phase 2 dose
R/R	Relapsed and/or refractory
SAE	Serious adverse event
SAP	Statistical analysis plan
SC	Steering committee
SD	Standard deviation
SE	Standard error
SGOT	Serum glutamic oxaloacetic transaminase
SGPT	Serum glutamic pyruvic transaminase
SIRPα	Signal-regulatory protein alpha
SMO	Smoothened receptor
SOP	Standard operating procedure
SRC	Safety review committee
SUSAR	Suspected unexpected serious adverse reaction
$t_{1/2}$	Half-life

EDMS Doc. Number: [redacted]

Table 16: Table of Abbreviations (Continued)

Abbreviation or Specialist Term	Explanation
t_{max}	Time to peak plasma concentration
TGF	Tumor growth factor
TGI	Tumor growth inhibition
TLS	Tumor lysis syndrome
TNBC	Triple-negative breast cancer
TNFα	Tumor necrosis factor alpha
TMZ	Temozolomide
ULN	Upper limit of normal
US	United States
USP	United States Pharmacopeia
V_{ss}/F	Apparent volume of distribution after multiple dosing
WBC	White blood cell count
WHO	World Health Organization
Wks	Weeks

Appendix B: Table of Events- Part A only

Table 17: Table of Events - Part A

Events[a]	Screening	Treatment Period																	Follow-up Period[b]	
		Cycle 1									Cycles 2-4					Cycles 5+		EOT	Safety	Long Term
		WK1				WK2		WK3		WK4	WK1		WK2	WK3	WK4	WK1	WK3			
	D-28 to -1	D1	D2	D3	D4/ D5	D8	D9	D15	D16- D19	D22	D1	D4	D8	D15	D22	D1	D15[c]	≤ 28 days	28 days (±3 days)	q3 mo (±2 wks)
**Study Entry (Section 6.1)**																				
Informed consent	X																			
Contraceptive counseling	X	X									X					X		X		
Informed consent for optional exploratory analyses/PK sampling	X																			
Inclusion/ exclusion criteria	X																			
Medical/ oncologic history and therapies	X																			
Demographics	X																			
IRT registration	X	X									X					X		X		
Prior/concomitant medications & procedures	Continuous starting from the time a subject sign the ICD until 28 days after the last dose of CC-90010																			
**Study Drug (Section 7)**																				
Administer oral CC-90010 per assigned dosing schedule (Section 7.2.1)[d]		X	X	X	X	X	X	X	X	X	X	X	X	X	X	X	X			
Provide/review of diary card		X	X	X	X	X	X	X	X	X	X	X	X	X	X	X	X	X		
IP accountability		X									X					X		X		
**Safety Assessments (Section 6)**																				
Adverse Event Monitoring (Section 6.2.2)	Continuous starting from the time a subject sign the ICD until 28 days after the last dose of CC-90010																			

EDMS Doc. Number: [redacted]

Table 17: Table of Events - Part A (Continued)

		**Treatment Period**																	**Follow-up Period**[b]	
		**Cycle 1**									**Cycles 2-4**					**Cycles 5+**				
	**Screening**	**WK1**				**WK2**		**WK3**		**WK4**	**WK1**		**WK2**	**WK3**	**WK4**	**WK1**	**WK3**	**EOT**	**Safety**	**Long Term**
**Events**[a]	**D-28 to -1**	**D1**	**D2**	**D3**	**D4/ D5**	**D8**	**D9**	**D15**	**D16-D19**	**D22**	**D1**	**D4**	**D8**	**D15**	**D22**	**D1**	**D15**[c]	**≤ 28 days**	**28 days (±3 days)**	**q3 mo (±2 wks)**
Height	X																			
Weight	X	X				X		X		X	X		X	X	X	X	X	X		
Vital Signs (Section 6.2.4)	X	X				X		X		X	X		X	X	X	X	X	X		
Physical Examination (Section 6.2.5)	X	X									X					X		X		
ECOG PS (Appendix H)	X	X									X					X		X		
B Symptoms Assessment (only NHL)	X				As clinically indicated													X		
12-lead ECG (single or triplicate; Section 6.2.7)[e]	X (≥72 hours prior to D1)	X	X (Last dose based on assigned dose schedule)								X					X		X		
LVEF (ECHO/MUGA; Section 6.2.8)	X	As clinically indicated																X (±7d)		
Pregnancy Testing (FCBP only) (Section 6.2.9)	X	X									X					X		X		
Hematology laboratory (Section 6.2.10)	X (D-14 to -1)	X				X		X		X	X		X (C2 only)	X	X (C2 only)	X		X		
Chemistry laboratory with LDH & uric acid tests (Section 6.2.10)	X (D-14 to -1)	X				X		X		X	X		X (C2 only)	X	X (C2 only)	X		X		
PT, INR, APTT, Factor VII	X (D-14 to -1)	X				X		X		X	X		X (C2 only)	X	X (C2 only)	X		X		
Hemoglobin A1c	X									X						X		X		

Table 17: Table of Events - Part A (Continued)

		Treatment Period																	Follow-up Period[b]	
		Cycle 1									Cycles 2-4					Cycles 5+				
	Screening	WK1				WK2		WK3		WK4	WK1		WK2	WK3	WK4	WK1	WK3	EOT	Safety	Long Term
Events[a]	D-28 to -1	D1	D2	D3	D4/ D5	D8	D9	D15	D16-D19	D22	D1	D4	D8	D15	D22	D1	D15[c]	≤ 28 days	28 days (±3 days)	q3 mo (±2 wks)
Amylase, lipase Insulin, C-Peptide		X											X (C2 only)			X (q3 cycles, ie, C5, C8, etc)		X		
Urinalysis (Section 6.2.10)	X (D-14 to -1)	X									X					X		X		
**PK & PD Assessments (Sections 6.5 & 6.6)**																				
Blood, PK of CC-90010 per assigned dosing schedule[f]		X	X	X	X	X	X	X	X	X										
CSF, PK[g] (Part A only)								X (D17)												
Blood (whole), PD per assigned dosing schedule[h]	X	X	X	X	X	X	X	X	X	X								X		
Tumor Biopsy[i] (Part A only)	X							X (D16 or D17)												
Archival tumor tissue (FFPE)[j]	X																			
**Efficacy (Section 6.4)**																				
Solid tumor/NHL assessments: CT/MRI imaging[k]	X														X (D28 ±7d; C2 & C4)	X (D28 ± 7d in C6, then q3 cycles, ie, end of C9, C12, etc)		X		

Table 17: Table of Events - Part A (Continued)

Events[a]	Screening	Treatment Period																	Follow-up Period[b]	
		Cycle 1									Cycles 2-4					Cycles 5+		EOT	Safety	Long Term
		WK1				WK2		WK3		WK4	WK1		WK2	WK3	WK4	WK1	WK3			
	D-28 to -1	D1	D2	D3	D4/ D5	D8	D9	D15	D16-D19	D22	D1	D4	D8	D15	D22	D1	D15[c]	≤ 28 days	28 days (±3 days)	q3 mo (±2 wks)
NHL-specific: bone marrow evaluation if known or suspected bone marrow involvement	X[l]										X, only when confirming CR									
NHL-specific: FDG PET or PET/CT scan (not required if tumor is FDG-negative)	X										Required to document response assessment if FDG-avid at Screening									
**Additional Follow-up (Section 6.3)**																				
Follow-up anticancer therapies (Part A only)																			X	X
AE/SAE follow-up																			X	
Survival follow-up																				X

Abbreviations: AE = adverse event; β - hCG = beta human chorionic gonadotropin; BMMC = bone marrow mononuclear cells; C = cycle; CK = creatine kinase; CSF = cerebrospinal fluid; CT = computed tomography; D = day(s); ECHO = echocardiogram; ECOG Eastern Cooperative Oncology Group; ECG = electrocardiogram; FCBP = females of child bearing potential; FDG = fluorodeoxyglucose; FFPE = formalin-fixed, paraffin embedded; INR = international normalized ratio; IRT = interactive response technology; LDH = lactic dehydrogenase; LVEF = left ventricular ejection fraction; mo = months; MRI = magnetic resonance imaging; MUGA = multi-gated acquisition scan; NHL= non-Hodgkin's lymphoma; PET = positron emission tomography; PD = pharmacodynamic; PK = pharmacokinetic; PS = performance score; PT = prothrombin time; PTH = parathyroid hormone; APTT = activated partial thromboplastin time; q = every; SAE = serious adverse event; WK(s) = week.

[a] All study visits/procedures will have a ± 3 day window and all laboratory blood samples should be drawn predose, unless otherwise specified in this table or Section 6.

[b] This Safety follow-up assessment may be by telephone (refer to Section 6.3.1). Long Term survival follow-up for up to 2 years or until death, lost to follow-up, or End of Trial, whichever occurs first. May be conducted by record review (including public records) and/or telephone contact with the subject, family, or treating physician.

[c] For Cycles 7 and higher, subjects will only complete Day 1 assessments. No assessments or visit is required on Day 15 (Week 3) unless clinically indicated.

[d] Not all CC-90010 dosing days are shown. Dose schedule is initially 3 consecutive days on CC-90010 and 4 consecutive days off each week (3/7-days schedule) in Part A. Alternative dosing schedules may be implemented based on SRC decisions. Refer to Section 7.2.1.

[e] Screening triplicate ECGs must be performed ≥ 72 hours prior to dosing on Day 1 so that the central read results are available for review. Triplicate ECGs will be performed in Cycle 1 (refer to Section 6.2.7). A single ECG will be performed on Day 1 of subsequent cycles and at EOT.

[f] Please refer to Section 6.5 and Appendix C for the PK schedule per assigned dosing schedule.

[g] Optional for subjects with a primary or metastatic CNS lesion and a shunt or reservoir in place or via lumbar tap. The recommended time for CSF collections is 4 hours (± 1 hour) after dosing on Day 17 (or on day of last dose of CC-90010 in Cycle 1). However, other times for CSF collection will be allowed as long as the CSF collection is on a PK day and is consistent with one of the scheduled blood PK collection times between 1 to 8 hours postdose. A time-matched blood sample must be collected along with each CSF sample.

[h] Please refer to Section 6.6.2 and Appendix D for the PD schedule per assigned dosing schedule. An additional time-matched blood PD sample should be collected during Cycle 2 Day 4 when the tumor biopsy is performed in Part B.

[i] Paired tumor biopsies are highly recommended for Part A, except for subjects with GBM. The Screening biopsy should be obtained after all inclusion/exclusion criteria have been fulfilled. In Part A only, the biopsy may be obtained on Cycle 1 Day 16 or 17 provided that 2 consecutive CC-90010 doses have been administered.
An optional tumor biopsy may also be obtained in both Part A during later treatment cycles. Refer to Section 6.6.3.

[j] Should be obtained by the end of Cycle 1. Not required if subject provides a Screening fresh tumor biopsy in Part A.

[k] All subjects who discontinue treatment for reasons other than disease progression, start of a new anticancer therapy, or withdrawal of consent from the entire study will be followed according to the specified tumor assessment schedule until progression and/or initiation of new systemic anticancer therapies.

[l] May be omitted if results were normal on the subject's most recent historical bone marrow biopsy. Additionally, this analysis may be omitted if a prior analysis was performed within 90 days before Cycle 1 Day 1. Historical results will be recorded in the eCRF.

Appendix C: Pharmacokinetic Blood Sampling Schedules for Part A, Cycle 1

Table 18: Pharmacokinetic Blood Sampling Schedule for the 3-days-on/4-days-off every week Schedule, Part A, Cycle 1

Time in Hours Relative to CC-90010 Dose	Collection Window	Days 1 and 17
0	Within 30 min prior to dosing	X
0.5	± 5 min	X
1	± 5 min	X
1.5	± 5 min	X
2	± 5 min	X
3	± 10 min	X
4	± 10 min	X
6	± 10 min	X
8	± 10 min	X
24	± 1 hour	X (prior to Day 2 and Day 18 dosing)

Table 19: Pharmacokinetic Blood Sampling Schedule for the 3-days-on/4-days-off every other week and 2-days-on/5-days-off each week Schedule, Part A, Cycle 1

Time in Hours Relative to CC-90010 Dose	Collection Window	Day 1	Last dose in Cycle 1
0	Within 30 min prior to dosing	X	X
0.5	± 5 min	X	X
1	± 5 min	X	X
1.5	± 5 min	X	X
2	± 5 min	X	X
4	± 10 min	X	X
6	± 10 min	X	X
8	± 10 min	X	X
24	± 1 hour	X (prior to dosing on Day 2)	X

EDMS Doc. Number: [redacted]

Table 19: Pharmacokinetic Blood Sampling Schedule for the 3-days-on/4-days-off every other week and 2-days-on/5-days-off each week Schedule, Part A, Cycle 1 (Continued)

Time in Hours Relative to CC-90010 Dose	Collection Window	Day 1	Last dose in Cycle 1
48	± 2 hours	X (prior to dosing on Day 3)	X
72	± 2 hours	X (Day 4)	
120	± 2 hours		X
168	± 2 hours	X (Day 8)	

Table 20: Pharmacokinetic Blood Sampling Schedule for the 4-days-on/24-days-off Schedule, Part A, Cycle 1

Time in Hours Relative to CC-90010 Dose	Collection Window	Day 1	Day 4
0	Within 30 min prior to dosing	X	X
0.5	± 5 min	X	X
1	± 5 min	X	X
1.5	± 5 min	X	X
2	± 5 min	X	X
4	± 10 min	X	X
6	± 10 min	X	X
8	± 10 min	X	X
24	± 1 hour	X (prior to dosing on Day 2)	X (Day 5)
48	± 2 hours	X (prior to dosing on Day 3)	
96	± 2 hours		X (Day 8)
120	± 2 hours		X (Day 9)

An exploratory analysis of CC-90010 metabolites in plasma may be performed utilizing the plasma samples collected for PK evaluation (no additional blood draws).

An exploratory analysis of CC-90010 concentrations in CSF may be performed for subjects who have a primary or metastatic CNS lesion with a shunt or reservoir in place or via a lumbar puncture and who provide consent for the optional collection. The recommended time for CSF collections is 4 hours (± 1 hour) after dosing on Day 17 (or the last day of CC-90010 dosing in

Cycle 1 if alternate dosing schedules are implemented). However, other times for CSF collection will be allowed as long as the time for CSF collections is between 1 to 10 hours postdose (refer to Table 17). A time-matched blood sample must be collected along with each CSF sample.

Appendix D: Pharmacodynamic Blood Sampling Schedules for Part A, Cycle 1

For the 3-days-on/4-days-off every week Schedule, Part A, Cycle 1

- Cycle 1 Day 1: pre-dose (≤ 3 hours), and 2, 4, 6, (each ± 15 minutes) and 24 hours (± 1 hour) after the CC-90010 dose

For the 3-days-on/4-days-off every other week and 2-days-on/5-days-off each week Schedule, Part A, Cycle 1

Table 21: Pharmacodynamic Blood Sampling Schedule for the 3-days-on/4-days-off every other week and 2-days-on/5-days-off each week Schedule, Part A, Cycle 1

Time in Hours Relative to CC-90010 Dose	Collection Window	Day 1	Last dose in Cycle 1
0	Within 30 min prior to dosing	X	X
2	± 5 min	X	X
4	± 10 min	X	X
6	± 10 min	X	X
24	± 1 hour	X (prior to dosing on Day 2)	X
48	± 2 hours	X (prior to dosing on Day 3)	X
72	± 2 hours	X (Day 4)	
120	± 2 hours		X
168	± 2 hours	X (Day 8)	

Table 22: Pharmacodynamic Blood Sampling for the 4-days-on/24-days-off Schedule, Part A, Cycle 1

Time in Hours Relative to CC-90010 Dose	Collection Window	Day 1	Day 4
0	Within 30 min prior to dosing	X	X
2	± 5 min	X	X
4	± 10 min	X	X
6	± 10 min	X	X
24	± 1 hours	X (prior to dosing on Day 2)	X (Day 5)

Table 22: Pharmacodynamic Blood Sampling for the 4-days-on/24-days-off Schedule, Part A, Cycle 1 (Continued)

Time in Hours Relative to CC-90010 Dose	Collection Window	Day 1	Day 4
48	± 2 hours	X (prior to dosing on Day 3)	
96	± 2 hours		X (Day 8)
120	± 2 hours		X (Day 9)

The Sponsor may collect additional unscheduled PD samples in order to follow up the safety of the study treatment or to better understand the progression of the disease or the disease's response to the study treatment.

Appendix E: RECIST Version 1.1

The following information is extracted/summarized from Eisenhauer, 2009, New Response Evaluation Criteria in Solid Tumors: Revised RECIST Guideline (Version 1.1). Please refer to the primary reference for further information.

Definitions

At screening, tumor lesions/lymph nodes will be categorized as measurable or non-measurable.

Measurable Disease

Tumor Lesions. Must be accurately measured in at least one dimension (longest diameter in the plane of measurement is to be recorded) with a minimum size of:

- 10 mm by CT scan (CT scan slice thickness no greater than 5 mm)
- 10 mm caliper measurement by clinical exam (lesions which cannot be accurately measured with calipers should be recorded as non-measurable)
- 20 mm by chest X-ray

Malignant Lymph Nodes. To be considered pathologically enlarged and measurable, a lymph node must be ≥ 15 mm in short axis when assessed by CT scan (CT scan slice thickness recommended to be no greater than 5 mm). At baseline and in follow-up, only the short axis will be measured and followed.

Non-measurable Disease

All other lesions, including small lesions (longest diameter <10 mm or pathological lymph nodes with ≥ 10 to < 15 mm short axis) as well as truly non-measurable lesions. Lesions considered truly non-measurable include: leptomeningeal disease, ascites, pleural or pericardial effusion, inflammatory breast disease, lymphangitic involvement of skin or lung, abdominal masses/abdominal organomegaly identified by physical exam that is not measurable by reproducible imaging techniques.

Tumor Response Evaluation

Target lesions

When more than one measurable tumor lesion is present at baseline all lesions up to a maximum of 5 lesions total (and a maximum of 2 lesions per organ) representative of all involved organs should be identified as target lesions and will be recorded and measured at baseline. Target lesions should be selected on the basis of their size (lesions with the longest diameter), be representative of all involved organs, but in addition should be those that lend themselves to reproducible repeated measurements. Note that pathological nodes must meet the measurable criterion of a short axis of ≥ 15 mm by CT scan and only the short axis of these nodes will contribute to the baseline sum. All other pathological nodes (those with short axis ≥ 10 mm but < 15 mm) should be considered non-target lesions. Nodes that have a short axis < 10 mm are considered non-pathological and should not be recorded or followed. At baseline, the sum of the target lesions (longest diameter of tumor lesions plus short axis of lymph nodes: overall maximum of 5) is to be recorded.

After baseline, a value should be provided on the eCRF for all identified target lesions for each assessment, even if very small. If extremely small and faint lesions cannot be accurately measured but are deemed to be present, a default value of 5 mm may be used. If lesions are too small to measure and indeed are believed to be absent, a default value of 0 mm may be used.

Non-target lesions

All non-measurable lesions (or sites of disease) plus any measurable lesions over and above those listed as target lesions are considered non-target lesions. Measurements are not required but these lesions should be noted at baseline and should be followed as “present,” “absent,” or “unequivocal progression.”

Response Criteria

Target and non-target lesions are evaluated for response separately, and then the tumor burden as a whole is evaluated as the overall response.

Target Lesion Response

Target lesions will be assessed as follows:

- Complete Response (CR). Disappearance of all target lesions. Any pathological lymph nodes (whether target or non-target) must have reduction in short axis to < 10 mm.
- Partial Response (PR). At least a 30% decrease in the sum of diameters of target lesions, taking as reference the baseline sum diameters.
- Progressive Disease (PD). At least a 20% increase in the sum of diameters of target lesions, taking as reference the smallest sum on study (this includes the baseline sum if that is the smallest on study). In addition to the relative increase of 20%, the sum must also demonstrate an absolute increase of at least 5 mm. (Note: the appearance of one or more new lesions is also considered progression).
- Stable Disease (SD). Neither sufficient shrinkage to qualify for PR nor sufficient increase to qualify for PD, taking as reference the smallest sum of diameters while on study.

Non-target Lesion Response

Non-target lesions will be assessed as follows:

- Complete Response (CR). Disappearance of all non-target lesions and normalisation of tumor marker level. All lymph nodes must be non-pathological in size (< 10 mm short axis).
- Non-CR/Non-PD. Persistence of one or more non-target lesion(s) and/or maintenance of tumor marker level above the normal limits.
- Progressive Disease (PD). Unequivocal progression (see comments below) of existing non-target lesions. (Note: the appearance of one or more new lesions is also considered progression).

When the Subject Also Has Measurable Disease: In this setting, to achieve “unequivocal progression” on the basis of the non-target disease, there must be an overall level of substantial

worsening in non-target disease such that, even in presence of SD or PR in target disease, the overall tumor burden has increased sufficiently to merit discontinuation of therapy. A modest "increase" in the size of one or more non-target lesions is usually not sufficient to quality for unequivocal progression status. The designation of overall progression solely on the basis of change in non-target disease in the face of SD or PR of target disease will therefore be extremely rare.

When the Subject Has Only Non-measurable Disease: This circumstance arises in some Phase 3 trials when it is not a criterion of study entry to have measurable disease. The same general concepts apply here as noted above; however, in this instance there is no measurable disease assessment to factor into the interpretation of an increase in non-measurable disease burden. Because worsening in non-target disease cannot be easily quantified (by definition: if all lesions are truly non-measurable) a useful test that can be applied when assessing subjects for unequivocal progression is to consider if the increase in overall disease burden based on the change in non-measurable disease is comparable in magnitude to the increase that would be required to declare PD for measurable disease: ie, an increase in tumor burden representing an additional 73% increase in "volume" (which is equivalent to a 20% increase diameter in a measurable lesion). Examples include an increase in a pleural effusion from "trace" to "large," an increase in lymphangitic disease from localized to widespread, or may be described in protocols as "sufficient to require a change in therapy." If "unequivocal progression" is seen, the subject should be considered to have had overall PD at that point. While it would be ideal to have objective criteria to apply to non-measurable disease, the very nature of that disease makes it impossible to do so: therefore, the increase must be substantial.

Overall Response

Overall response should be assessed according to Table 23 for subjects with target lesions, and Table 24 for subjects with only non-target lesions.

Table 23: Time Point Response: Subjects With Target (± Non-target) Disease

Target Lesions Response	Non-target Lesion Response	New Lesions	Overall Response
CR	CR	No	CR
CR	Non-CR/ non-PD	No	PR
CR	Not evaluated	No	PR
PR	Non-PD or not all evaluated	No	PR
SD	Non-PD or not all evaluated	No	SD
Not all evaluated	Non-PD	No	NE
PD	Any	Yes or No	PD
Any	PD	Yes or No	PD
Any	Any	Yes	PD

CR = complete response, NE = inevaluable , PD = progressive disease , PR = partial response, SD = stable disease.

Table 24: Time Point Response: Subjects With Non-target Disease Only

Non target Lesions Response	New Lesions	Overall Response
CR	No	CR
Non-CR/ non-PD	No	Non-CR/ non-PD[a]
Not all evaluated	No	NE
Unequivocal PD	Yes or No	PD
Any	Yes	PD

CR = complete response, NE = inevaluable , PD = progressive disease, PR = partial response, SD = stable disease, .
[a] "Non-CR/non-PD" is preferred over "stable disease" for non-target disease since SD is increasingly used as endpoint for assessment of efficacy in some trials so to assign this category when no lesions can be measured is not advised.

Symptomatic Deterioration

Subjects with a global deterioration of health status requiring discontinuation of treatment without objective evidence of disease progression at that time should be reported as 'symptomatic deterioration'. Every effort should be made to document objective progression even after discontinuation of treatment. Symptomatic deterioration is not a descriptor of an objective response: it is a reason for stopping study therapy. The objective response status of such subjects is to be determined by evaluation of target and non-target disease.

Appendix F: The Lugano Classification Criteria for the Evaluation of Non-Hodgkin Lymphoma and the Deauville 5-Point Scale

The Lugano Classification

The guidelines for Recommendations for Initial Evaluation, Staging, and Response Assessment of Hodgkin and Non-Hodgkin Lymphoma: The Lugano Classification are outlined in a report (Cheson, 2014).

Table 25: Criteria for Involvement of Site

Tissue Site	Clinical	FDG Avidity	Test	Positive Finding
Lymph nodes	Palpable	FDG-avid histologies<br>Nonavid disease	PET-CT<br>CT	Increase FDG uptake<br>Unexplained node enlargement
Spleen	Palpable	FDG-avid histologies<br>Nonavid disease	PET-CT<br>CT	Diffuse uptake, solitary mass, miliary lesions, nodules > 13 cm in vertical length, mass, nodules
Liver	Palpable	FDG-avid histologies<br>Nonavid disease	PET-CT<br>CT	Diffuse uptake, mass<br>Nodules
CNS	Signs, symptoms		CT<br>MRI<br>CSF assessment	Mass lesion(s)<br>Leptomeningeal infiltration, mass lesions<br>Cytology, flow cytometry
Other (eg, skin, lung, GI tract, bone, bone marrow)	Site dependent		PET-CT[a], biopsy	Lymphoma involvement

CNS = central nervous system; CSF = cerebrospinal fluid; CT = computed tomography; FDG = fluorodeoxyglucose; GI = gastrointestinal; MRI = magnetic resonance imaging; PET = positron emission tomography.

[a] PET-CT is adequate for determination of bone marrow involvement and can be considered highly suggestive for involvement of other extralymphatic sites. Biopsy confirmation of those sites can be considered if necessary.

Table 26: Revised Criteria for Response Assessment

Response and site	PET-CT based response	CT-based response
**Complete**	**Complete metabolic response**	**Complete radiologic response (all of the following)**
Lymph nodes and extralymphatic sites	Score 1, 2, or 3[a] with or without a residual mass on 5PS[b]<br>It is recognized that in Waldeyer's ring or extranodal sites with high physiologic uptake or with activation within spleen or marrow (eg, with chemotherapy or myeloid colony- stimulating factors), uptake may be greater than normal mediastinum and/or liver. In this circumstance, complete metabolic response may be inferred if uptake at sites of initial involvement is no greater than surrounding normal tissue even if the tissue has high physiologic uptake	Target nodes/nodal masses must regress to ≤ 1.5 cm in LDi<br>No extralymphatic sites of disease
Nonmeasured lesion	Not applicable	Absent
Organ enlargement	Not applicable	Regress to normal
New lesions	None	None
Bone marrow	No evidence of FDG-avid disease in marrow	Normal by morphology; if indeterminate, IHC negative
**Partial**	**Partial metabolic response**	**Partial remission (all of the following)**
Lymph nodes and extralymphatic sites	Score 4 or 5[b] with reduced uptake compared with baseline and residual mass(es) of any size<br>At interim, these findings suggest responding disease<br>At end of treatment, these findings indicate residual disease	≥ 50% decrease in sum of perpendicular diameters (SPD) of up to 6 target measurable nodes and extranodal sites<br>When a lesion is too small to measure on CT, assign 5 mm × 5 mm as the default value<br>When no longer visible, 0 × 0 mm<br>For a node > 5 mm × 5 mm, but smaller than normal, use actual measurement for calculation
Nonmeasured lesion	Not applicable	Absent/normal, regressed, but no increase
Organ enlargement	Not applicable	Spleen must have regressed > 50% in length beyond normal
New lesions	None	None
Bone marrow	Residual uptake higher than uptake in normal marrow but reduced compared with baseline (diffuse uptake compatible with reactive changes from chemotherapy allowed). If there are persistent focal changes in the marrow in the context of a nodal response, consideration should be given to further evaluation with MRI or biopsy or an interval scan	Not applicable

Table 26: Revised Criteria for Response Assessment (Continued)

Response and site	PET-CT based response	CT-based response
**No response or stable disease**	**No metabolic response**	**Stable disease**
Target nodes/nodal masses, extranodal lesions	Score 4 or 5[b] with no significant change in FDG uptake from baseline at interim or end of treatment	< 50% decrease from baseline in SPD of up to 6 dominant measurable nodes and extranodal sites; no criteria for progressive disease are met
Nonmeasured lesion	Not applicable	No increase consistent with progression
Organ enlargement	Not applicable	No increase consistent with progression
New lesions	None	None
Bone marrow	No change from baseline	Not applicable
**Progressive disease**	**Progressive metabolic response**	**Progressive disease requires at least 1 of the following**
Individual target nodes/nodal masses<br>Extranodal lesions	Score 4 or 5[b] with an increase in intensity of uptake from baseline and/or<br>New FDG-avid foci consistent with lymphoma at interim or end-of-treatment assessment	PPD progression:<br>An individual node/lesion must be abnormal with:<br>LDi > 1.5 cm and<br>Increase by ≥ 50% from PPD nadir and<br>An increase in LDi or SDi from nadir<br>0.5 cm for lesions ≤ 2 cm<br>1.0 cm for lesions > 2 cm<br>In the setting of splenomegaly, the splenic length must increase by > 50% of the extent of its prior increase beyond baseline (eg, a 15 cm spleen must increase to > 16 cm). If no prior splenomegaly, must increase by at least 2 cm from baseline<br>New or recurrent splenomegaly
Nonmeasured lesions	None	New or clear progression of preexisting nonmeasured lesions
New lesions	New FDG-avid foci consistent with lymphoma rather than another etiology (eg, infection, inflammation). If uncertain regarding etiology of new lesions, biopsy or interval scan may be considered	Regrowth of previously resolved lesions<br>A new node > 1.5 cm in any axis<br>A new extranodal site > 1.0 cm in any axis, if < 1.0 cm in any axis, its presence must be unequivocal and must be attributable to lymphoma<br>Assessable disease of any size unequivocally attributable to lymphoma
Bone marrow	New or recurrent FDG-avid foci	New or recurrent involvement

5PS = 5-point scale; CT = computed tomography; FDG = fluorodeoxyglucose; GI = gastrointestinal; IHC = immunohistochemistry; LDi = longest transverse diameter of a lesion; MRI = magnetic resonance imaging; PET = positron emission tomography; PPD = cross product of the LDi and perpendicular diameter; SDi = shortest axis perpendicular to the LDi; SPD = sum of the product of the perpendicular diameters for multiple lesions.

EDMS Doc. Number: [redacted]

[a] A score of 3 in many Subjects indicates a good prognosis with standard treatment, especially if at the time of an interim scan. However, in trials involving PET where de-escalation is investigated, it may be preferable to consider a score of 3 as inadequate response (to avoid undertreatment).

Measured dominant lesions: Up to six of the largest dominant nodes, nodal masses, and extranodal lesions selected to be clearly measurable in two diameters. Nodes should preferably be from disparate regions of the body and should include, where applicable, mediastinal and retroperitoneal areas. Non-nodal lesions include those in solid organs (eg, liver, spleen, kidneys, and lungs), GI involvement, cutaneous lesions, or those noted on palpation. Nonmeasured lesions: Any disease not selected as measured, dominant disease and truly assessable disease should be considered not measured. These sites include any nodes, nodal masses, and extranodal sites not selected as dominant or measurable or that do not meet the requirements for measurability but are still considered abnormal, as well as truly assessable disease, which is any site of suspected disease that would be difficult to follow quantitatively with measurement, including pleural effusions, ascites, bone lesions, leptomeningeal disease, abdominal masses, and other lesions that cannot be confirmed and followed by imaging. In Waldeyer's ring or in extranodal sites (eg, GI tract, liver, bone marrow), FDG uptake may be greater than in the mediastinum with complete metabolic response, but should be no higher than surrounding normal physiologic uptake (eg, with marrow activation as a result of chemotherapy or myeloid growth factors).

[b] PET 5PS: 1, no uptake above background; 2, uptake ≤ mediastinum; 3, uptake > mediastinum but ≤ liver; 4, uptake moderately > liver; 5, uptake markedly higher than liver and/or new lesions; X, new areas of uptake unlikely to be related to lymphoma.

Source: Cheson, 2014.

The Deauville five-point scale

The Deauville five-point scale (5PS) is an internationally recommended scale for clinical routine and clinical trials using FDG-PET/CT in the initial staging and assessment of treatment response in Hodgkin's lymphoma (HL) and certain types of non-Hodgkin's lymphomas (NHL).

Usage

It is a simple tool based on visual interpretation of FDG-uptake. It takes advantage of two reference points of the individual patient, which have demonstrated relatively constant uptake on serial imaging. The two reference organs are the mediastinum (aka blood pool) and the liver.

The scale ranges from 1 to 5, where 1 is best and 5 is the worst. Each FDG-avid (or previously FDG-avid) lesion is rated independently.

1. no uptake or no residual uptake (when used interim)
2. slight uptake, but below blood pool (mediastinum)
3. uptake above mediastinal, but below or equal to uptake in the liver
4. uptake slightly to moderately higher than liver
5. markedly increased uptake or any new lesion (on response evaluation)

Assessment of treatment response

- complete response (CR): scores 1, 2 or 3 together with the absence of FDG-avid bone marrow lesion(s) are interpreted as complete metabolic response (CR), irrespective of a persistent mass on CT
- partial response (PR): a Deauville score of 4 or 5, provided:
  - uptake is decreased compared with baseline and
  - absence of structural progression development on CT
- stable disease (SD), also called no metabolic response: a Deauville score of 4 or 5 without significant change in FDG uptake from baseline.

- progressive disease (PD): a Deauville score of 4 to 5 with increasing intensity compared to baseline or any interim scan and/or any new FDG-avid focus consistent with malignant lymphoma.

Appendix G: Response Assessment for Neuro-Oncology (RANO) Working Group

The Response Assessment Criteria for High-Grade Gliomas: Response Assessment in Neuro-Oncology Working Group (Wen, 2010) can be accessed online at:

https://ascopubs.org/doi/pdf/10.1200/JCO.2009.26.3541
(click on "manual download for full text PDF of manuscript)

Appendix H: Performance Status Criteria

Table 27: Eastern Cooperative Oncology Group (ECOG) Performance Status

Score	Description
**0**	Fully active, able to carry on all pre-disease performance without restriction
**1**	Restricted in physically strenuous activity but ambulatory and able to carry out work of a light or sedentary nature, eg, light housework, office work.
**2**	Ambulatory and capable of all self-care but unable to carry out any work activities. Up and about more than 50% of waking hours.
**3**	Capable of only limited self-care, confined to bed or chair more than 50% of waking hours.
**4**	Completely disabled. Cannot carry on any self-care. Totally confined to bed or chair
**5**	Dead

Source: Oken, 1982.

EDMS Doc. Number: [redacted]

Appendix I: General Guidelines for Managing Hyperglycemia

Fasting glucose is defined as a level monitored ≥ 4 hours from the last meal for assessment of dose-limiting toxicity and clinical management decisions. Subjects should be instructed on how to recognize hypo- and hyperglycemia. Any subject who experiences hyperglycemia or symptoms associated with hyperglycemia should be managed per standard of care with CC-90010 interruptions/reductions as described in Section 7.2.9. Additional guidelines are described below.

- In the event of persistent fasting hyperglycemia (> 126 mg/dL or 14 mmol/L), or at any time considered appropriate by the Investigator, it is recommended that treatment with an oral anti-diabetic agent (OAD) be initiated.
- In the event of Grade ≥ 3 fasting hyperglycemia, monitoring in the clinic should occur until the hyperglycemia resolves to Grade ≤ 2.
- In the event of persistent Grade 3 fasting hyperglycemia (> 250 mg/dL or 27.8 mmol/L), insulin therapy should be considered either in conjunction with an OAD or alone. Long-acting insulin should only be used when the subject is hospitalized. Monitoring of glucose should continue for at least 6 hours following administration of insulin (fast-or long-acting) due to possible rebound effects. The medical monitor should be notified.
- In the event of a Grade 4 fasting blood glucose (>50 mg/dL or 27.8 mmol/L), CC-90010 will be withheld (refer to Section 7.2.9) while insulin therapy is initiated. The medical monitor should be notified. Treatment interruptions of > 4 weeks will necessitate removal of the subject from this study.
- At the discretion of the Investigator, daily home monitoring via fingerstick testing (while fasting in the AM) may be initiated. Subjects will be provided a glucometer and will be trained how to perform fingerstick testing and document results in a diary card which will be reviewed during each clinic visit. They will also be instructed how to contact study staff immediately in the event of a high fasting glucose result (> 160 mg/dL or 8.9 mmol/L). Prompt assessment in the clinic is necessary for Grade 3 or higher measurements. The opinion of an endocrinologist regarding adequate management of the subject may be advisable in such cases.

Glucophage, and other biguanide therapy, should be temporarily suspended when planned radiological tumor assessments (eg, CT scan) involves iodinated contrast. Goldberg, 2005; and Turina, 2006 are suggested resources for hyperglycemia management.

EDMS Doc. Number: [redacted]

Appendix J: Recommendations for Management of Treatment-Induced Diarrhea

The following published guidelines (Benson, 2004) were modified in order to be consistent with the study protocol.

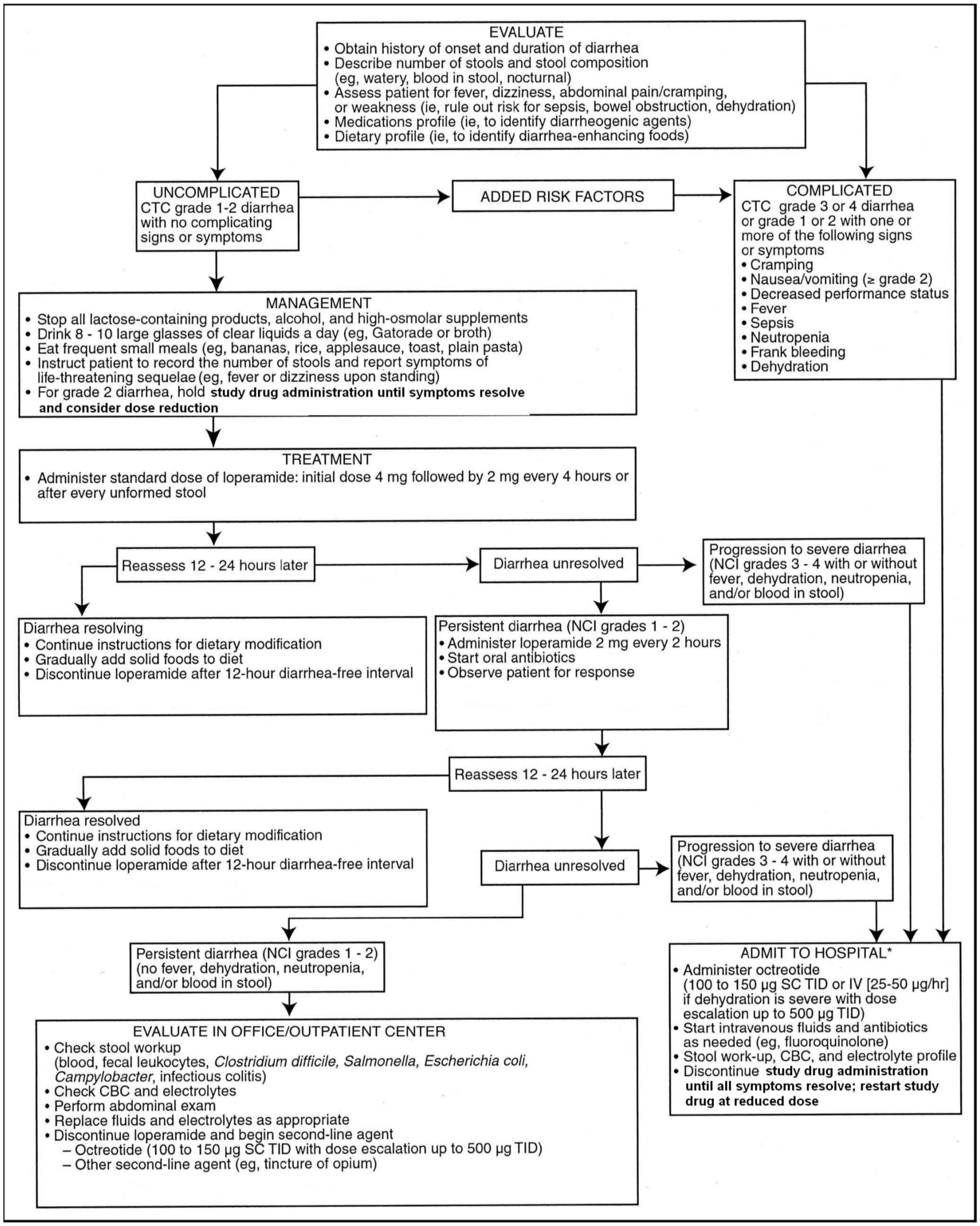

Appendix K: Characteristics of the Bayesian Logistic Regression Model

Introduction

An adaptive Bayesian logistic regression model (Neuenschwander, 1998) for dose escalation with overdose control (Babb, 1988) will be used to guide dose escalation in this study. The BLRM and prior specification are described in Section 9.1.

The purpose of this appendix is to present performance metrics (operating characteristics) that illustrate the precision of the design in estimating the MTD under various dose-toxicity relationships and different dose schedules through computer simulation.

Specifications and results of simulation study

This section presents the operating characteristics that illustrate the precision of the design in estimating the MTD under various assumed true dose-toxicity relationships.

Simulations are performed for the BLRM under a total of 5 scenarios of true dose-DLT relationship (refer to Table 28 and Figure 6):

1. Dose-DLT relationship is a steep curve and MTD is reached at early dose level (SE).
2. Dose-DLT relationship is a steep curve and MTD is reached at middle dose level (SM).
3. Dose-DLT relationship is a steep curve and MTD is reached at late dose level (SL).
4. Dose-DLT relationship is a flat curve and MTD is reached at middle dose level (FM).
5. Dose-DLT relationship is a flat curve and MTD is reached at late dose level (FL).

These five dose-toxicity curves have been constructed under 3 alternative dosing schedules (eg, 2-days- on/5-days- off each week, 3 days on/4-days-off every other week, 4-days- on/24-days-off). The originally planned primary schedule (orally once daily for 3- days –on/4- days-off every week) has only one dose level tested and did not move forward so the simulation was not provided for the this dosing schedule although the data was used in model fitting.

The DLT relationship in the escalation part of the study will be described by the following Bayesian logistic regression model:

$$logit(p_i) = \log(\alpha) + \beta \log\left(\frac{d_i}{d^*}\right) + \gamma_1 x_{i1} + \gamma_2 x_{i2} + \gamma_3 x_{i3}$$

where α and β specify the shape of logistic curve that represents the overall dose-toxicity relationship, $\gamma_1, \gamma_2, \gamma_3$ specify the four different dosing schedules.

In simulation, the dose escalation/de-escalation occurs in each dosing schedule simultaneously. MTD is declared in each dosing schedule separately according to the rule defined in Section 7.2.2.

Table 28: P(DLT) for Five Simulated Scenarios with Numbers in Grey Indicating Doses with True P(DLT) within the Target Toxicity Interval [16%, 33%] under 3 different dosing schedules.

a. Schedule 2

	45 mg	90 mg	150 mg	240 mg	330 mg
SE	0.1807	0.3109	0.4332	0.5539	0.633
SM	0.0135	0.0688	0.2033	0.4439	0.6336
SL	0.0002	0.0026	0.0174	0.0946	0.2581
FM	0.0506	0.113	0.1948	0.3039	0.3944
FL	0.0084	0.0321	0.0828	0.1852	0.2981

b. Schedule 3

	60 mg	120 mg	200 mg	280 mg	360 mg
SE	0.1696	0.2947	0.4145	0.5349	0.6151
SM	0.0123	0.0629	0.1883	0.4206	0.6113
SL	0.0002	0.0022	0.0146	0.0806	0.2259
FM	0.0464	0.1042	0.181	0.285	0.3729
FL	0.0076	0.0291	0.0755	0.1705	0.2775

c. Schedule 4

	60 mg	120 mg	180 mg	220 mg	260 mg
SE	0.1915	0.3263	0.4508	0.5714	0.6494
SM	0.0148	0.0749	0.2185	0.4667	0.6546
SL	0.0002	0.003	0.0205	0.11	0.2915
FM	0.0535	0.1188	0.2039	0.3161	0.4081
FL	0.0093	0.0352	0.0904	0.2001	0.3185

Note: the highlighted dose levels represent MTD (DLT probability is between 16% and 33%).

EDMS Doc. Number: [redacted]

Figure 6: Dose Toxicity Curves for Simulation

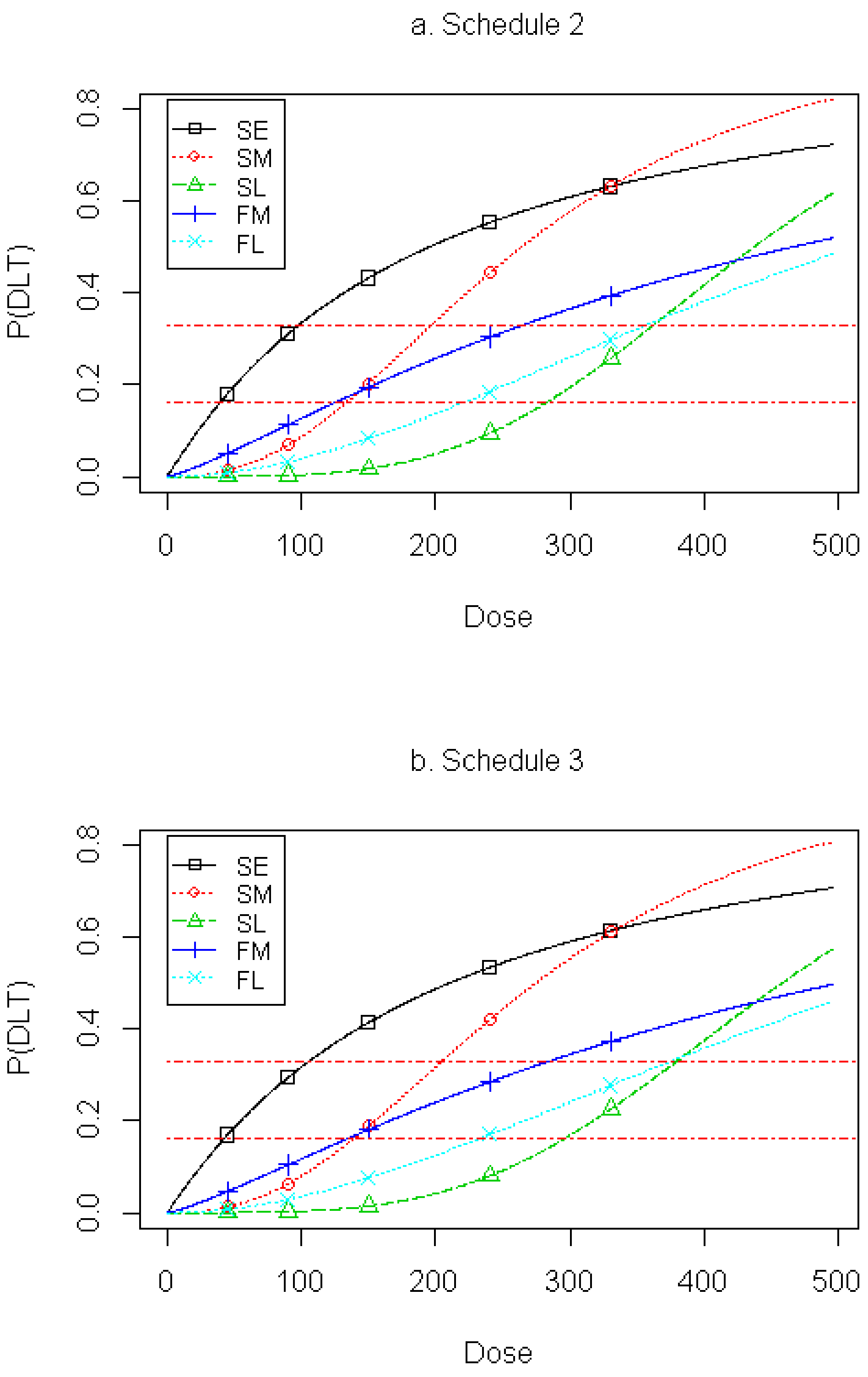

Figure 6: Dose Toxicity Curves for Simulation (Continued)

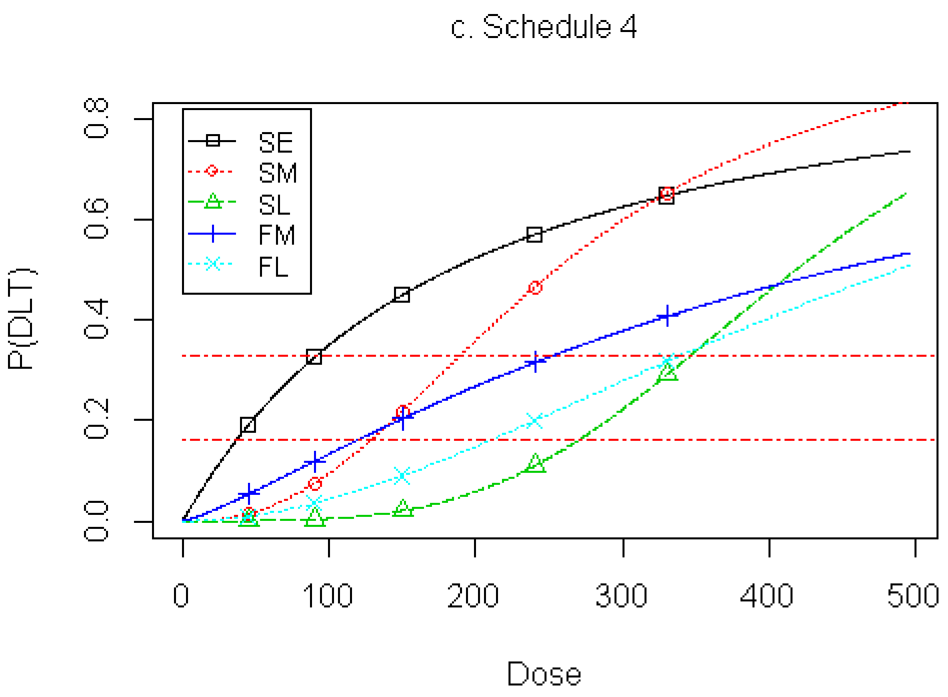

Operating characteristics are reviewed to investigate overall performance of the BLRM under each true scenario. Table 29 summarizes the results from the simulations performed.

Table 29: Summary Metrics of Simulation for BLRM under 5 dose-toxicity curves and different dosing schedule.

Scenario/ Method	Mean Number of Subjects	Proportion of subjects with DLT	Schedule 2: Dose levels with various DLT probability 0.16-0.33	≥0.33	<0.16	Schedule 3: Dose levels with various DLT probability 0.16-0.33	≥0.33	<0.16	Schedule 4: Dose levels with various DLT probability 0.16-0.33	≥0.33	<0.16
SE, BLRM	30.79	0.27	0.74	0.04	0.22	0.74	0.06	0.2	0.53	0	0.47
SM, BLRM	48.15	0.18	0.81	0.07	0.12	0.63	0.08	0.29	0.4	0.04	0.56
SL, BLRM	64.23	0.11	0.48	0	0.52	0.55	0	0.45	0.66	0	0.34
FM, BLRM	48.49	0.17	0.74	0.04	0.21	0.63	0.1	0.27	0.45	0.04	0.51
FL, BLRM	58.13	0.13	0.75	0	0.25	0.76	0	0.24	0.8	0	0.2

Overall the BLRM model with specified prior is performing reasonably. It is able to identify the correct MTD with high percentage ranging from 40% to 81%.

Discussion

The Bayesian Logistic Regression Model enables us to incorporate the historical information, as well as to update the recommended dose based on all safety data in the study.

By reviewing the metrics presented in the table, it can be seen that the model is not sensitive to different scenarios of truth. In general, this model is conservative due to the overdose control criteria. In all scenarios, the probability of recommending a dose that is excessively toxic with true P(DLT) ≥ 33% is much smaller than that of recommending a dose with true P(DLT) between 16% and 33% as MTD. On-study recommendations based on the model are consistent with the clinical decision making process, and should be considered in conjunction with other available clinical information by the Celgene Clinical Trial Team and study investigators in deciding the dose levels to be tested in order to determine the MTD.

Celgene Signing Page

**This is a representation of an electronic record that was signed electronically in Livelink.
This page is the manifestation of the electronic signature(s) used in compliance with
the organizations electronic signature policies and procedures.**

UserName: [redacted]
Title: [redacted]
Date: Thursday, 16 April 2020, 10:21 AM Eastern Daylight Time
Meaning: Approved, no changes necessary.
==
