# Supplementary material for: BET inhibitor trotabresib in heavily pretreated patients with solid tumors and diffuse large B-cell lymphomas
Source: Nat Commun. 2023 Mar 13;14:1359. doi: 10.1038/s41467-023-36976-1 (PMC10011554; doi:10.1038/s41467-023-36976-1)
Supplement: Supplementary file 2 — Reporting Summary [file 41467_2023_36976_MOESM2_ESM.pdf]

nature portfolio

Corresponding author(s): Victor Moreno

Last updated by author(s): 23 February 2023

Reporting Summary

Nature Portfolio wishes to improve the reproducibility of the work that we publish. This form provides structure for consistency and transparency in reporting. For further information on Nature Portfolio policies, see our Editorial Policies and the Editorial Policy Checklist.

Statistics

For all statistical analyses, confirm that the following items are present in the figure legend, table legend, main text, or Methods section.

n/a	Confirmed	
☐	☒	The exact sample size (*n*) for each experimental group/condition, given as a discrete number and unit of measurement
☐	☒	A statement on whether measurements were taken from distinct samples or whether the same sample was measured repeatedly
☒	☐	The statistical test(s) used AND whether they are one- or two-sided<br>*Only common tests should be described solely by name; describe more complex techniques in the Methods section.*
☒	☐	A description of all covariates tested
☒	☐	A description of any assumptions or corrections, such as tests of normality and adjustment for multiple comparisons
☐	☒	A full description of the statistical parameters including central tendency (e.g. means) or other basic estimates (e.g. regression coefficient) AND variation (e.g. standard deviation) or associated estimates of uncertainty (e.g. confidence intervals)
☒	☐	For null hypothesis testing, the test statistic (e.g. *F*, *t*, *r*) with confidence intervals, effect sizes, degrees of freedom and *P* value noted<br>*Give P values as exact values whenever suitable.*
☒	☐	For Bayesian analysis, information on the choice of priors and Markov chain Monte Carlo settings
☒	☐	For hierarchical and complex designs, identification of the appropriate level for tests and full reporting of outcomes
☒	☐	Estimates of effect sizes (e.g. Cohen's *d*, Pearson's *r*), indicating how they were calculated

Our web collection on statistics for biologists contains articles on many of the points above.

Software and code

Policy information about availability of computer code

Data collection	No software was used
Data analysis	No software was used

For manuscripts utilizing custom algorithms or software that are central to the research but not yet described in published literature, software must be made available to editors and reviewers. We strongly encourage code deposition in a community repository (e.g. GitHub). See the Nature Portfolio guidelines for submitting code & software for further information.

Data

Policy information about availability of data

All manuscripts must include a data availability statement. This statement should provide the following information, where applicable:

- Accession codes, unique identifiers, or web links for publicly available datasets
- A description of any restrictions on data availability
- For clinical datasets or third party data, please ensure that the statement adheres to our policy

Anonymized participant-level data cannot be shared due to an increased risk of patient reidentification. Bristol Myers Squibb will consider requests to share anonymized clinical trial data from interventional trials in patients that have completed on or after January 1, 2008. In addition, primary results from these trials must have been published in peer-reviewed journals and the medicines or indications approved in the U.S., EU, and other designated markets. Requests to access clinical trial data will be considered case by case and may be submitted to Celgene, a Bristol Myers Squibb company, using the enquiry form at https://vivli.org/

ourmember/bristol-myers-squibb/. Requests for clinical data are initially reviewed by internal Bristol Myers Squibb personnel to ensure alignment with the scope of the data sharing policy and to check current or expected availability of the data sets and are then evaluated by an Independent Review Committee to ensure that qualifying requests have a consistent, complete, and fair assessment. Sharing is also subject to protection of patient privacy and respect for the patient's informed consent. and must include a description of the research proposal. Information from eligible trials that may be considered for disclosure upon request includes deidentified study-level clinical data, Clinical Study Reports, Statistical Analysis Plans, and Protocols. Source data are provided with this paper. The study protocol is available as Supplementary Note 1 in the Supplementary Information file. The remaining data are available within the Article, Supplementary Information or Source Data file.

Human research participants

Policy information about studies involving human research participants and Sex and Gender in Research.

Reporting on sex and gender	Patients enrolled in this study have been listed as male or female where applicable in the manuscript. Data for patients' gender were not collected, and no analyses based on sex were performed.
Population characteristics	Median patient ages varied from 57 to 67 years across treatment group (range 21 to 80 years). A total of 51% of patients were male and 49% of patients were female. Eastern Cooperative Oncology Group performance status was 0 in 49% of patients and 1 in 51% of patients. Patients had a variety of tumor types, including glioblastoma (9%), basal cell carcinoma (1%), NUT carcinoma (1%), other advanced solid tumors (70%), and diffuse large B-cell lymphoma (19%). The median number of prior systemic anticancer therapies varied from 1 to 4 across treatment groups (range 1 to 9). Six of 23 (26%) patients with DLBCL had received a prior stem cell transplant. Median time from initial diagnosis to the first dose of study drug varied from 28.5 to 222.8 months across treatment groups (range 2.2 to 444.6).
Recruitment	Patients meeting the study eligibility criteria were identified from the general population receiving treatment at each participating site by the investigators, and referred for participation in the trial. Eligible patients were aged ≥18 years with an ECOG PS of 0 or 1.<br><br>Part A included patients with histologically or cytologically confirmed advanced or unresectable solid tumors or R/R advanced NHL that had progressed on standard anticancer therapy or for which no conventional therapy was available.<br><br>Part B included patients with histologically or cytologically confirmed R/R DLBCL that had progressed following two or more previous lines of therapy, including autologous stem cell transplant, or that had progressed after at least one previous line of therapy and where the patient was not eligible for or had declined autologous stem cell transplant. Patients with transformed lymphoma following chemotherapy for follicular lymphoma who had received at least two standard treatment regimens for DLBCL were also eligible. Patients must have had lack of response after chimeric antigen receptor (CAR) T-cell therapy (if available), been ineligible for CAR T-cell therapy, or declined CAR T-cell therapy. Part B also included a cohort of patients with histologically or cytologically confirmed advanced BCC, nuclear protein in testis (NUT) midline carcinoma, advanced salivary gland carcinoma, or advanced endometrial carcinoma and disease progression on, or inability to tolerate, standard therapy, or for whom no standard therapy exists. Patient enrollment in this cohort was subsequently restricted to patients with advanced BCC only in a protocol amendment.<br><br>Part C included patients with histologically or cytologically confirmed advanced solid tumors and disease progression on, or inability to tolerate, standard therapy, or for whom no standard therapy exists.<br>Patients with solid tumors had at least one site of measurable disease; patients with NHL had bidimensionally measurable disease on cross-sectional imaging, with at least one lesion >1.5 cm in diameter.
Ethics oversight	The study was conducted in accordance with the Declaration of Helsinki and in adherence to Good Clinical Practice. The protocol was reviewed and approved by the Institutional Review Board or Independent Ethics Committee of each site before initiation of the study, and all patients provided written informed consent. The study protocol was approved by the Institution Review Board or Independent Ethics Committee of Institut Gustave Roussy, Villejuif, France; Institut Bergonie Centre Regional de Lutte Contre Le Cancer de Bordeaux Et Sud Ouest, Bordeaux, France; Vall d'Hebron University Hospital, Vall d'Hebron Institute of Oncology (VHIO), Barcelona, Spain; Hospital Universitario Fundación Jimenez Diaz, Madrid, Spain; Hospital Universitario 12 de Octubre, Madrid, Spain; Istituto Nazionale Tumori, Fondazione 'G. Pascale, IRCCS, Naples, Italy; Instituto Clinico Humanitas, Rozzano, Milano, Italy; Instituto Scientifico Romagnolo per lo Studio e la Cura dei Tumori, Meldola, Italy; National Cancer Center Hospital East, Tokyo, Japan; Aichi Cancer Center Hospital, Nagoya, Japan; and The Cancer Institute Hospital of Japanese Foundation for Cancer Research, Tokyo, Japan.

Note that full information on the approval of the study protocol must also be provided in the manuscript.

Field-specific reporting

Please select the one below that is the best fit for your research. If you are not sure, read the appropriate sections before making your selection.

☒ Life sciences ☐ Behavioural & social sciences ☐ Ecological, evolutionary & environmental sciences

For a reference copy of the document with all sections, see nature.com/documents/nr-reporting-summary-flat.pdf

Life sciences study design

All studies must disclose on these points even when the disclosure is negative.

Sample size	Dose escalation decisions in part A were guided by a Bayesian logistic regression model with overdose control (Babb J, et al. Stat Med 1998;17:1103-20 and Neuenschwander B, et al. Stat Med 2008;27:2420-39). The number of patients treated at each dose level and schedule was based on empirical considerations, with a maximum of 6 patients typically treated in each group so as to minimize the number of patients treated at subtherapeutic doses while providing adequate data to inform on potential dose-limiting toxicities prior to evaluation of the next dose level. For the part B R/R DLBCL expansion cohort, enrollment was based on the probability of making a no-go decision when the true efficacy was truly below the target level. For instance, if the no-go criteria is Pr (ORR <26%) >80%, enrollment of 25 patients would provide a 73% chance to make a no-go decision when the true ORR is 14% (based on posterior probability of beta-binomial distribution with prior beta [0.35, 1]). Planned enrollment for the part C food effect cohort was also driven by empirical experience, with a minimum sample size of 24 patients believed to produce sufficient precision for PK parameter assessment based on preliminary PK results from part A.
Data exclusions	No data were excluded from the analysis
Replication	Each participating patient was followed from the time of enrollment until study discontinuation or death. No replication was possible.
Randomization	This study was a phase I first-in-human trial. All patients received open-label treatment with trotabresib; assigned study dose and treatment group was allocated sequentially and was not controlled for covariates.
Blinding	This study was a phase I first-in-human trial. All patients received open-label treatment with trotabresib.

Reporting for specific materials, systems and methods

We require information from authors about some types of materials, experimental systems and methods used in many studies. Here, indicate whether each material, system or method listed is relevant to your study. If you are not sure if a list item applies to your research, read the appropriate section before selecting a response.

Materials & experimental systems

n/a	Involved in the study
☒	☐ Antibodies
☒	☐ Eukaryotic cell lines
☒	☐ Palaeontology and archaeology
☒	☐ Animals and other organisms
☐	☒ Clinical data
☒	☐ Dual use research of concern

Methods

n/a	Involved in the study
☒	☐ ChIP-seq
☒	☐ Flow cytometry
☒	☐ MRI-based neuroimaging

Clinical data

Policy information about clinical studies

All manuscripts should comply with the ICMJE guidelines for publication of clinical research and a completed CONSORT checklist must be included with all submissions.

Clinical trial registration	NCT03220347
Study protocol	Data requests may be submitted to Celgene, a Bristol Myers Squibb company, at https://vivli.org/ourmember/bristol-myers-squibb/. Bristol Myers Squibb will consider requests to share clinical trial data from our in-scope Phase II-IV interventional trials in patients that have completed on or after January 1, 2008. In addition, primary results from these trials must have been published in peer-reviewed journals and the medicines or indications approved in the U.S., EU, and other designated markets. Sharing is also subject to protection of patient privacy and respect for the patient's informed consent. and must include a description of the research proposal. Information from eligible trials that may be considered for disclosure upon request includes the protocol.
Data collection	Patients were enrolled at 11 cancer centers in France, Italy, Japan, and Spain (Institut Gustave Roussy, Villejuif, France; Institut Bergonie Centre Regional de Lutte Contre Le Cancer de Bordeaux Et Sud Ouest, Bordeaux, France; Vall d'Hebron University Hospital, Vall d'Hebron Institute of Oncology (VHIO), Barcelona, Spain; Hospital Universitario Fundación Jimenez Diaz, Madrid, Spain; Hospital Universitario 12 de Octubre, Madrid, Spain; Istituto Nazionale Tumori, Fondazione 'G. Pascale, IRCCS, Naples, Italy; Instituto Clinico Humanitas, Rozzano, Milano, Italy; Instituto Scientifico Romagnolo per lo Studio e la Cura dei Tumori, Meldola, Italy; National Cancer Center Hospital East, Tokyo, Japan; Aichi Cancer Center Hospital, Nagoya, Japan; and The Cancer Institute Hospital of Japanese Foundation for Cancer Research, Tokyo, Japan) between July 24, 2017 and the time of manuscript preparation, at which point patient enrollment was ongoing. Data were collected between July 24, 2017 and June 16, 2022.
Outcomes	The primary endpoints of the study were the safety and tolerability of trotabresib, as well as the MTD and/or RP2D of trotabresib. Safety was assessed using the National Cancer Institute Common Terminology Criteria for Adverse Events (NCI CTCAE) v4.03.<br><br>Secondary objectives were the preliminary efficacy of trotabresib in terms of clinical benefit rate (CR + PR + SD of ≥4 months'

duration), ORR (CR + PR), duration of response or SD, PFS, OS, and the PK of trotabresib. In addition, part C assessed the effects of food on the PK of trotabresib as a secondary objective.

Response was assessed using Response Evaluation Criteria in Solid Tumors (RECIST) v1.1 in patients with solid tumors, International Working Group criteria in patients with DLBCL, and Response Assessment in Neuro-Oncology (RANO) criteria in patients with CNS tumors (astrocytoma and glioblastoma). For patients with BCC, response was assessed using a combination of radiological assessment of target lesions per RECIST v1.1, digital clinical photography assessed per World Health Organization (WHO) criteria, and punch biopsies to confirm CR. Two-sided 95% Clopper–Pearson exact CIs were calculated for ORR and CBR estimates, and median PFS and OS with 95% CIs were calculated using the Kaplan–Meier method.
